# Role and mechanism of *NCAPD3* in promoting malignant behaviors in gastric cancer

**DOI:** 10.3389/fphar.2024.1341039

**Published:** 2024-04-22

**Authors:** Su-Yun Zhang, Qiong Luo, Li-Rong Xiao, Fan Yang, Jian Zhu, Xiang-Qi Chen, Sheng Yang

**Affiliations:** ^1^ Departments of Oncology Medicine, Fujian Medical University Union Hospital, Fuzhou, Fujian, China; ^2^ Departments of Respiratory and Critical Care Medicine, Fujian Medical University Union Hospital, Fuzhou, Fujian, China; ^3^ Department of General Surgery, Shengjing Hospital of China Medical University, Shenyang, Liaoning, China; ^4^ Fujian Key Laboratory of Translational Research in Cancer and Neurodegenerative Diseases, Fuzhou, Fujian, China

**Keywords:** gastric cancer, *NCAPD3*, proliferation, apoptosis, molecular mechanism

## Abstract

**Background:**

Gastric cancer (GC) is one of the major malignancies threatening human lives and health. Non-SMC condensin II complex subunit D3 (*NCAPD3*) plays a crucial role in the occurrence of many diseases. However, its role in GC remains unexplored.

**Materials and Methods:**

The Cancer Genome Atlas (TCGA) database, clinical samples, and cell lines were used to analyze *NCAPD3* expression in GC. *NCAPD3* was overexpressed and inhibited by lentiviral vectors and the CRISPR/Cas9 system, respectively. The biological functions of *NCAPD3* were investigated *in vitro* and *in vivo*. Gene microarray, Gene set enrichment analysis (GSEA) and ingenuity pathway analysis (IPA) were performed to establish the potential mechanisms.

**Results:**

*NCAPD3* was highly expressed in GC and was associated with a poor prognosis. *NCAPD3* upregulation significantly promoted the malignant biological behaviors of gastric cancer cell, while *NCAPD3* inhibition exerted a opposite effect. *NCAPD3* loss can directly inhibit CCND1 and ESR1 expression to downregulate the expression of downstream targets CDK6 and IRS1 and inhibit the proliferation of gastric cancer cells. Moreover, *NCAPD3* loss activates IRF7 and DDIT3 to regulate apoptosis in gastric cancer cells.

**Conclusion:**

Our study revealed that *NCAPD3* silencing attenuates malignant phenotypes of GC and that it is a potential target for GC treatment.

## 1 Introduction

Currently, gastric cancer is one of the major malignancies threatening human lives and health (Smyth et al., 2020; Sun et al., 2020). The global cancer statistics report (GLOBOCAN) showed that there were 1.04 million new cases of gastric cancer globally in 2018, making it the fifth most common malignancy, and 0.78 million deaths caused by this disease, making it the third leading cause of cancer death (Bray et al., 2018). Gastric cancer (GC) is a multi-step process that is affected by *Helicobacter pylori* infection, host susceptibility, and other environmental factors. Gastric cancer is also a multifactorial process caused by the accumulation of a large number of genetic and epigenetic changes in oncogenes and tumor suppressor genes, which results in dysregulation in many signaling pathways, disruption of cell cycle, and disturbance of the equilibrium between proliferation and death (Berger et al., 2016). Therefore, there are still many challenges in gastric cancer prevention and treatment, and how to improve gastric cancer diagnosis and treatment is still a global focus and hotspot. Tumor molecular biology studies have shown that tumorigenesis and tumor progression is an extremely complex biological behavior involving the participation of many genes, multifactorial interactions, and multi-stage development (Bass et al., 2014). Therefore, in-depth understanding of the molecular biology mechanisms of gastric cancer occurrence and progression is vital in the search for more effective prevention and treatment measures.

Non-SMC condensin II complex subunit D3 (*NCAPD3*) is a subunit of condensin II and the *NCAPD3* gene is located in chromosome 13q25. Condensin II is a pentameric complex consisting of XCAPD3, CAP-G2, CAP-H2, SMC2, and SMC4 (Zhang et al., 2014). Condensin complexes are divided into condensin I and condensin II. *NCAPD3* and condensin II were first discovered in human HeLa cells and named by Ono et al. (2004). In 2008, Maeshima et al. found that condensins play a critical role in chromosome condensation and separation during mitosis in eukaryotic cells (Maeshima and Eltsov, 2008). In 2011, Abe et al. found that cyclin-dependent kinase-mediated *NCAPD3* phosphorylation in the prophase of mitosis can result in chromosome condensation (Abe et al., 2011). In 2015, Bakhreha et al. found that inducing a CAP-D3^T1403A^ mutation in the *NCAPD3* ortholog in chicken DT40 cells can cause shortening of the mitotic chromosome axis, leading to disruption of cell division during the prophase (Bakhrebah et al., 2015). Although little is known about the expression and role of *NCAPD3* in human tumors, aberrant *NCAPD3* expression and its potential effects have been observed in tumor tissues. In 2008, Lapointe pointed out that the postoperative recurrence rate is lower in prostate cancer patients with low postoperative *NCAPD3* expression. Hence, *NCAPD3* can be used as a prognostic predictor of prostate cancer after surgery (Lapointe et al., 2008). In 2016, Dawkins et al. found that low *NCAPD3* expression in pancreatic cancer is intimately associated with good prognosis (Dawkins et al., 2016). However, there have been no studies on the correlation between *NCAPD3* and gastric cancer.

In the present study, deep mining of gastric cancer and paracancerous tissue gene sequences in The Cancer Genome Atlas (TCGA) was performed, and RNA sequence data of gastric cancer and paracancerous tissues in the TCGA database were analyzed. The results showed that *NCAPD3* is significantly upregulated in gastric cancer tissues. Then gastric cancer and normal gastric mucosal tissues were randomly selected from 67 gastric cancer patients who underwent radical subtotal gastrectomy or total gastrectomy in Fujian Medical University Union Hospital. These tissues were used for immunohistochemical staining. The results showed that *NCPADC3* is highly expressed in gastric cancer tissues and is intimately associated with poor prognosis. These findings demonstrated the potential importance and clinical value of *NCAPD3* in gastric cancer prevention and treatment.

Subsequently, *in vitro* and *in vivo* experiments were conducted on *NCAPD3* to investigate the effects of *NCAPD3* on gastric cancer cell proliferation, invasion, migration, and apoptosis through overexpression and knockout/knockdown experiments. To understand the potential molecular mechanisms of *NCAPD3* knockdown on malignant cytological behavior in gastric cancer, advanced molecular biology techniques and gene chips were employed to measure the effects of *NCAPD3* knockdown on gene expression and its related functional pathways, and the potential biological mechanisms of *NCAPD3* were examined after obtaining the gene expression spectrum. Next, gene set enrichment analysis (GSEA) was employed to further elucidate the effects of *NCAPD3* knockdown on canonical pathways, cellular components, and immune, oncogene, and transcription factor gene sets to examine the role of *NCAPD3* knockdown in gastric cancer occurrence and progression at different levels (Bustin et al., 2009; Liu et al., 2017; Zhang et al., 2021). In summary, this *NCAPD3* research is expected to provide a new target for gastric cancer treatment and inspire new therapeutic strategies for in-depth basic research and new drug development in clinical practice for gastric cancer.

## 2 Materials and Methods

### 2.1 TCGA data download

Gastric cancer (stomach adenocarcinoma, STAD) RNAseq database and clinical information were downloaded from TCGA (https://cancergenome.nih.gov/). As of 30 June 2018, there were 443 samples with useable gastric cancer data in the TCGA database, of which 416 were mRNA chip or RNAseq data samples, and 32 pairs were RNAseq v2 paired sample data with pathological information. Biological coefficient of variation (BCV) was observed for quality control, and 26 paired samples with stable data were selected. Expression spectrum analysis was performed based on this paired sample RNAseq data.

### 2.2 Experimental materials

AGS, SGC7901, MGC803, and BGC823 gastric cancer cell lines were purchased from Shanghai Genechem. RPMI 1640 culture medium, PBS, and fetal bovine serum were purchased from Hyclone (United States), and Opti-MEM culture medium was purchased from Gibco (United States). *NCAPD3* overexpression lentivirus (LV-*NCAPD3*), blank control vector lentivirus (LV-NC1), shRNAs targeting the *NCAPD3* gene (shRNA-*NCAPD3*-1/2/3), single guide RNAs (sgRNAs) targeting human *NCAPD3* (sgRNA-*NCAPD3*-1/2/3) and their negative control (shRNA-NC and sgRNA-NC) were purchased from Shanghai Genechem. Supplementary Table S1 shows the sequences of shRNAs and sgRNAs. Knockout and Mutation Detection Kit were purchased from Shanghai Genesci Medical Technology. The TRIzol reagent was purchased from Shanghai Pufei Biotechnology. The reverse transcription kit was purchased from Promega (United States). PCR primers were synthesized by Shanghai Genechem. The MTT assay kit was purchased from Genview (United States). The AnnexinV-APC apoptosis assay kit was purchased from eBioscience (United States). Transwell chambers with matrigel were purchased from Corning (United States). *NCAPD3*, TNFAIP3, FADD, IRS1, SMAD3, CD44, and MAP1LC3B antibodies were purchased from Abcam (United Kingdom). The CDK6 antibody was purchased from CST (United States). The FLAG antibody was purchased from Sigma-Aldrich (Germany). The GAPDH antibody was purchased from Santa-Cruz Biotechnology (United States). The marker (catalogue number 26619) was purchased from Thermo (United States).

### 2.3 Patients and tissue specimens

All clinical samples, including 67 pairs of gastric cancer and paracancerous tissues, were obtained from the tissue bank of Fujian Medical University Union Hospital. The application of archived cancer samples was approved by the Ethics Committee of Fujian Medical University Union Hospital (No. 2021WSJK042). In this study, no subjects received preoperative radiotherapy or chemotherapy. All resected specimens were stored at −80 °C for long-term storage. Written informed consents were obtained from all of patients or their guardians.

### 2.4 Gastric cancer cell culture

RPMI-1640/DMEM containing 10% fetal bovine serum and 1% penicillin and streptomycin solution was used for culture of the four gastric cancer cell lines (AGS, MGC803, BGC823, and SGC7901) in a 37 °C, 5% CO_2_ incubator.

### 2.5 Cell transfection

The lentivirus plasmid containing full-length *NCAPD3* was used for *NCAPD3* overexpression, and the empty plasmid was used as a negative control. Additionally, shRNA plasmids targeting *NCAPD3* were used for *NCAPD3* knockdown, and shRNA plasmids containing non-specific scrambled shRNA sequences were used as a negative control (shNCs). Lentiviruses were used to transfect knockdown plasmids into GC cells. The culture medium was changed 6 h after lentivirus vectors were transfected into GC cells. Fetal bovine serum (FBS, 10%) was added to the new culture medium, and total RNA and total protein were extracted after 48 h of culture.

### 2.6 NCAPD3 gene knockout using the CRISPR/Cas9 system

The CRISPR/Cas9 system contains the LV-cas9-puro and LV-sgRNA-EGFP recombinant lentiviruses vectors. LV-cas9-puro carries a puromycin resistance gene and LV-sgRNA-EGFP contains an enhanced green fluorescent protein (EGFP) tag. The three single guide RNAs (sgRNAs) targeting human *NCAPD3* were designed and synthesized by Shanghai Genechem. Sequencing was used to validate the sequences of the synthesized sgRNAs. The LV-cas9-puro and LV-sgRNA-EGFP vectors were constructed by Shanghai Genechem.

First, LV-cas9-puro lentiviruses were used to transfect AGS cells. Three days after transfection, a suitable amount of puromycin was used for 3 days of selection to obtain AGS cells with stable Cas9 expression. Following that, the three LV-sgRNA-EGFP lentiviruses were used to transfect Cas9-AGS cells. After 3 days of transfection, an inverted microscope was used to look for green fluorescent protein (GFP), and the percentage of green, fluorescent cells was calculated.

### 2.7 CruiserTM enzymatic cleavage experiment

The Knockout and Mutation Detection Kit was used to detect gene knockout 5 days after LV-sgRNA-EGFP lentivirus infection according to the manufacturer’s instructions. In brief, the genomic DNA extraction kit was used to extract genomic DNA for PCR amplification according to the manufacturer’s instructions. The PCR conditions used were as follows: pre-denaturation at 94 °C for 90 s, followed by 40 cycles of denaturation at 94 °C for 30 s, annealing at 60 °C for 30 s, and extension at 72 °C for 60 s, followed by final extension at 72 °C for 5 min. The PCR products were cooled to <40 °C. Then, 1 μL Cruiser™ was added to 3 μL PCR product, and the mixture was incubated at 45 °C for 20 min for enzymatic cleavage. Finally, 2% agarose gel electrophoresis was used to observe enzymatic cleavage. Supplementary Table S2 shows the PCR primer sequences.

### 2.8 MTT assay

Gastric cancer cells in the logarithmic growth phase were harvested and seeded at 1,500 cells/well in a 96-well plate. Triplicates were set up for every group, and the final volume of culture medium in each well was 100 µL. The cells were cultured under normal conditions. After 24, 48, 72, 96, and 120 h, 20 µL MTT (5 mg/mL) was added, and the 96-well plates were cultured normally for 4 h in an incubator. After that, the culture medium was carefully aspirated and 100 µL DMSO was added. The plates were incubated with shaking for 2–5 min before a microplate reader was used to read the optical density (OD) of each well at 490 nm.

### 2.9 Cell apoptosis assay

Pre-cooled PBS was used to wash the cells before trypsin was used for digestion and cells were collected. Following that, 4 °C pre-cooled D-Hanks solution was used to wash the cells, and then 1 × binding buffer was added. Cells were collected by centrifugation before 200 µL 1 × binding buffer was used to resuspend the cell pellet. Next, 10 µL AnnexinV-APC was added for staining. After incubating at room temperature in the dark for 10–15 min, a flow cytometer was used to measure changes in apoptosis rate in the various groups. This procedure was carried out in triplicate for each sample.

### 2.10 Transwell invasion assay

After cells had undergone trypsin digestion, serum-free culture medium was used for washing. Following that, serum-free culture medium was used to resuspend cells and enumeration was carried out. A cell suspension of 10 × 10^4^ cells/200 μL was added to every Transwell chamber, and 650 μL of 30% FBS complete culture medium was added to the lower chamber, with triplicate wells for each group. The cells were incubated in a 37 °C incubator for 20 h. After 20 h of culture, 1 mL of 4% formaldehyde was added to every well, and room temperature fixation was carried out for 10 min. The cells were stained, the fixing solution was discarded, and 1× PBS was used to wash the cells once. Subsequently, 1 mL of 0.5% crystal violet solution was added to every well. At 30 min after staining, 1× PBS was used to wash the wells thrice. The plate was dried and observed. A cotton bud was used to gently remove cells that had not migrated in the Transwell chambers, and the chambers were observed under a 200 × microscope. Three random fields were selected per well for enumeration, and ImageJ was used for enumeration. Each expriment was done in triplicate.

### 2.11 Scratch assay

Cells in the logarithmic growth phase from the various experimental groups were digested with trypsin before complete culture medium was used to resuspend the cells (plating density was determined based on cell size to achieve >90% confluency on the following day). Next, cells were cultured in 96-well plates in a 37 °C and 5% CO_2_ incubator, with quintuplicate wells per group and 100 μL/well. On the following day, a wound making tool was used to gently scratch the center of the lower part of wells in the 96-well plates. PBS was used to gently wash the plates 2–3 times, and 0.5% FBS culture medium was added. Photographs were taken, and Celigo was used to analyze the migration area at 0 h and 8 h after scratching.

### 2.12 Real-time fluorescence quantitative PCR

Trizol reagent was used to extract total RNA from the various groups, followed by reverse transcription to cDNA. Next, fluorescence quantitative PCR was carried out. The primer sequences for real-time PCR analysis are listed in Supplementary Table S3. The reaction conditions were pre-denaturation at 95 °C for 30 s followed by 40 cycles of 95 °C for 5 s and 60 °C for 30 s. Triplicate wells were set up for every sample, and the 2^−ΔΔCT^ method was used to analyze RT-PCR data. Relative mRNA expression changes were calculated (Bustin et al., 2009).

### 2.13 Western blotting (WB)

Cells in the logarithmic growth phase were collected and washed twice with PBS. The RIPA lysis buffer and protease inhibitor (or phosphatase inhibitor) mixture was used to extract total protein after transfection. The BCA assay kit was used for protein quantitation. Following that, SDS-PAGE was used to separate proteins (20–30 µg), and proteins were transferred to a PVDF membrane. The membrane was blocked with TBST buffer containing 5% skimmed milk at room temperature for 1 h. Next, the membrane was incubated with primary antibodies at 4 °C overnight. The membrane was washed four times with TBST followed by incubation with secondary antibody for 1.5 h. The membrane was washed four times with TBST, and the ECL reagent was used for luminescence, followed by development and imaging. Briefly, excess ECL solution was removed and membrane was put inside plastic wrap inside X-ray film cassette. Next, we expose membrane to the X-ray film in cassette in dark room for 1–2 min. We developed with the help of Carestream medical film processor using fixer and developer and then measure the band intensity using ImageJ.

### 2.14 Co-immunoprecipitation (Co-IP)

Co-IP was performed using the protein A/G plus-agarose immunoprecipitation kit (Santa Cruz Biotechnology, United States), according to the manufacturer’s instructions (Liu et al., 2017). Briefly, the GC cells were lysed by RIPA lysis buffer, followed by total protein extraction. The concentration of proteins was detected using BCA assay kit according to previous studies (Liu et al., 2017; Zhang et al., 2021). Then cell lysate was incubated overnight incubation at 4 °C with IP antibody, followed by incubation with 20 μL protein A/G PLUS-Agarose for 1 h to form an immune complex. The complexes were washed twice with RIPA lysis buffer and resuspended in 6× loading buffer, denatured for 10 min. The suspensions were further analyzed by Western blotting.

### 2.15 Immunohistochemistry

Selected gastric cancer tissue samples and their paired paracancerous tissue samples, which were fixed with 4% formaldehyde embedded in paraffin blocks, were made into 3 μm thick continuous sections. The sections were adhered to poly-L-lysine-coated glass slides and dried in a 70 °C oven for 4 h. *NCAPD3* monoclonal antibody was purchased from Abcam PLC (United Kingdom). SP immunohistochemistry assay kit and DAB substrate were purchased from Fuzhou Maixin Biotech. Staining was performed according to the manufacturer’s instructions, and PBS was used instead of the primary antibody for the negative control. The positive control was provided by the company. Xylene, absolute ethanol, and PBS were analytical grade.

Immunohistochemistry staining of *NCAPD3* was scored by two independent experienced pathologists. For each sample, the score of staining intensity was assigned as follows: 0, negative staining; 1, weak staining (light yellow); 2, moderate staining (yellow brown) and 3, strong staining (brown) (Luo et al., 2020). And the percentage of stained cells was scored as 0 (<5% stained cells); 1 (5%–10% stained cells); 2 (11%–50% stained cells); 3 (51%–80% stained cells) and 4 (>80% stained cells). The final score was defined as staining score multiplied by proportion score (Guo et al., 2021). Final scores of 0–4 and 6–12 were considered to be low and high expression, respectively (Hou et al., 2014).

### 2.16 Animal experiments

Twenty five-week-old male nude mice were randomized into two groups: the negative control group in which untreated MGC803 gastric cancer cells were inoculated, and the *NCAPD3*-knockdown group in which MGC803 gastric cancer cells that were transfected with *NCAPD3*-shRNA lentivirus vector were inoculated. In each mouse, 4.0 × 10^6^ cells were inoculated subcutaneously below the axilla. Tumor volume (volume = π/6×L×W×W, L: length, W: short axis, unit: mm^3^) was calculated 30 days after inoculation for one to two times a week. On day 38, nude mice were euthanized by cervical dislocation after intraperitoneal injection of 2% pentobarbital sodium anesthesia, and the tumors were harvested and weighed. In order to minimize the bias from individual differences, the maximum and minimum mice in each group were removed from data analysis. All of the experimental protocols were approved by the Institutional Animal Care and Use Committee of Fujian Medical University (No. 2021-8CAARM125), and the animal experiments were conducted in Animal Center of Fujian Medical University.

### 2.17 Gene expression spectrum analysis

In order to obtain the *NCAPD3*-regulated gene expression spectrum, AGS cells were transfected with sh*NCAPD3* lentiviruses and control vector to construct AGS-KD and AGS-NC cells. Next, qPCR was used to validate the efficiency of RNA interference. The Trizol reagent was used to extract total RNA for an RNA quality test. The quality standards were: NanoDrop 2000 (Thermo Fisher Scientific, Waltham, MA, United States), 1.7 < A260/A280 < 2.2; Agilent 2100 Bioanalyzer (Agilent Technologies, Santa Clara, CA, United States), RIN ≥7.0, 28S/18S > 0.7. The 3′ IVT Plus Kit (Affymetrix, Santa Clara, CA, United States), a reverse transcription kit, was used for labeling of RNA that passed the quality test according to the manufacturer’s instructions. The labeled RNAs were fragmented and hybridized. The Affymetrix GeneChip PrimeView human gene expression array was used for testing. The selection criteria for significantly differentially expressed genes were |Fold Change| ≥ 2.0 and FDR <0.05. The experiment was completed in Shanghai Genechem. GeneChip Scanner 3000 (Affymetrix) was used for data analysis.

### 2.18 GSEA

In order to examine changes in overall biological processes and pathways after *NCAPD3* knockdown, GSEA was carried out on expression data. First, the probe group with coefficient of variation >25% in the *NCAPD3* knockdown group and control group were removed to obtain the filtered expression matrix. The filtered overall expression matrix was inputted into the GSEA. The MSigDB database was used for enrichment analysis of the background gene set, which is a combination of various gene sets, such as canonical pathway, cellular component, immunologic signatures, oncogenic signature, and transcription factor. The GSEA parameter settings were as follows: the permutation type was gene set, and “control vs. knockdown” was used for enrichment analysis. Therefore, the normalized enrichment score (NES) < 0 represents the degree of pathway enrichment of genes that were ranked in front of changes in the knockdown group. Default parameters were used for the other settings. FDR <0.05 was considered to indicate significant enrichment.

### 2.19 IPA analysis

The Ingenuity Pathway Analysis (IPA: Ingenuity Systems; www.ingenuity.com; Redwood City, CA, United States) database was used for bioinformatics analysis of differentially expressed genes. Canonical pathway analysis was carried out by comparison of differentially expressed genes and pathways containing these genes, and comparison significance (*p* < 0.05) was calculated to determine which pathways contained differentially expressed genes. Following that, the upstream and downstream regulatory factors of differentially expressed genes were compared. A Z-score ≥2 means that the pathway is significantly activated, whereas a Z-score ≤ −2 means that the pathway is significantly inhibited. The activation Z-score algorithm was used to analyze upstream regulatory factors to predict activation or inhibition of upstream regulatory factors. Disease and functional analysis was carried out according to IPA internal algorithm and standards. A Z-score ≥2 means that the disease or function is significantly activated, whereas a Z-score ≤ −2 means that the disease or function is significantly inhibited. Network map analysis was used to present the relationship between the disease and the differentially expressed gene. The Consistency Score is a measurement of the consistency and dense connection of causality between upstream regulatory factors and disease or function. The higher the Consistency Score, the more accurate the regulatory effect results. Therefore, IPA results were used to interpret and visualize interactions between upstream and downstream factors and global signal transduction.

### 2.20 Statistical methods

Excel 2016 was used for data processing, SPSS 22.0 was used for statistical analysis, GraphPad prism 6 was used for plotting of statistical graphs, and ImageJ was used for measurement of the migration area. Statistical description of quantitative data (mean ± standard deviation) was carried out. One-way ANOVA or *t*-test was used for comparison of inter-group differences. Qualitative data were described using ratios, and Chi-square test or Fisher’s exact probability test were used to compare differences. A difference with *p* < 0.05 was considered to be statistically significant.

## 3 Results

### 3.1 *NCAPD3* expression is increased in gastric cancer and is closely related to prognosis

**FIGURE 1:**
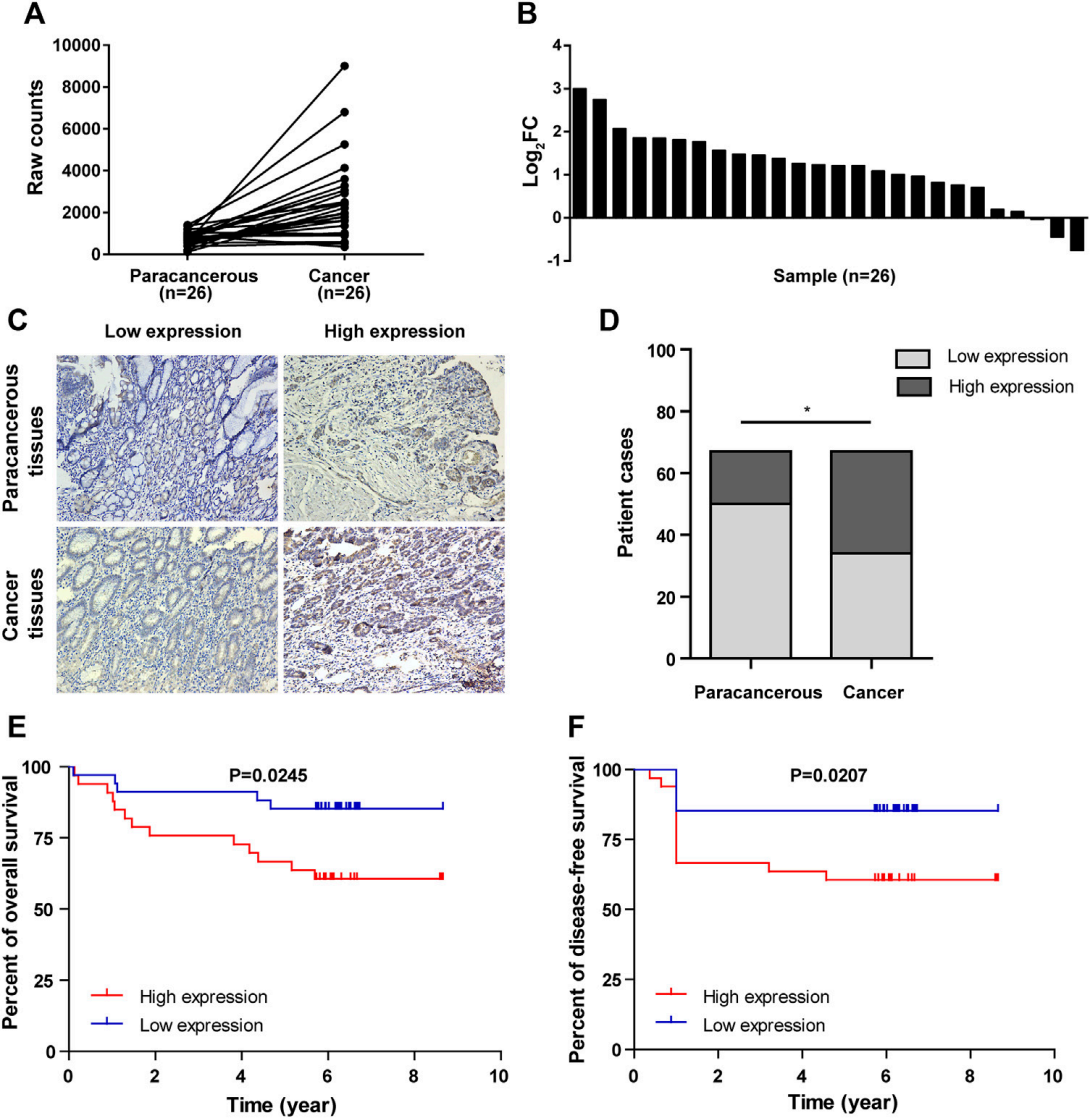
*NCAPD3* expression in gastric cancer tissues. **(A)** Line chart of differential expression of *NCAPD3* in 26 pairs of sequencing samples (gastric cancer and matched paracancerous samples) from TCGA. **(B)** Bar chart of differential expression of *NCAPD3* in 26 pairs of sequencing samples (gastric cancer and matched paracancerous samples) from TCGA. FC (fold change): ratio of expression level in cancerous samples to paracancerous samples. **(C)** Relative protein expression of *NCAPD3* in gastric cancer tissues (n = 67) compared with paracancerous normal tissues (n = 67) with immunohistochemistry. **(D)** Chart of positive immunohistochemical rates and the associated statistics. A total of 49% (33/67) of gastric cancer tissues were positive for *NCAPD3* expression, while 25% (17/67) of normal tissues were positive for *NCAPD3*. *p < 0.05. **(E)** Kaplan-Meier overall survival (OS) according to *NCAPD3* expression in 67 gastric cancer patients. **(F)** Kaplan-Meier Disease-free survival (DFS) by *NCAPD3* expression in 67 gastric cancer patients.

Deep mining of gastric cancer and paracancerous tissue gene sequences in The Cancer Genome Atlas (TCGA) was performed, and RNA sequence data of gastric cancer and paracancerous tissues in the TCGA database were analyzed. The result showed that *NCAPD3* expression level in gastric cancer tissues is significantly higher than paired paracancerous tissues, with a log2-fold change of 2.242 and FDR <0.01 (Figure 1A). In 26 pairs of sequencing samples, the expression level of *NACPAD3* in 17 pairs of gastric cancer tissues was significantly higher than paired paracancerous tissues (Figure 1B).

We then detected *NCAPD3* protein expression in 67 pairs of gastric cancer tissues and paracancerous tissues with immunohistochemistry. Representative IHC images posited that *NCAPD3* protein was primarily expressed in the cytoplasm of gastric cancer cells (Figure 1C). The analysis results of Chi-square test revealed that The *NCAPD3*-positive expression rate in 67 gastric cancer patients was significantly higher than in paracancerous normal gastric mucosal tissues (*p* = 0.004) (Figure 1D). According to the relevant *NCAPD3* expression in tumor tissues, 67 gastric cancer patients were classified into two groups: low-expression group (n = 34) and high-expression group (n = 33), the correlation between *NCAPD3* expression and clinicopathological characteristics in gastric cancer patients were shown in Table 1. Expression of *NCAPD3* was identified to be correlated with invasion depth (*p* = 0.009), lymph node metastases (*p* = 0.029) and pathological TNM stage (*p* < 0.001) but not with other clinicopathological characteristics in patients with gastric cancer. As for prognosis, the patients with *NCAPD3*-high expression had a significantly worse overall survival (OS) and a high risk of relapse than the *NCAPD3*-low patients. The overall survival rate for the *NCAPD3*-low patients was 85.3 percent, as compared with 60.6 percent for the patients with high *NCAPD3* expression (*p* = 0.0245); the five-year disease-free survival (DFS) rate was 85.3 percent, as compared with 63.6 percent (*p* = 0.0207 by the log-rank test), respectively (Figures 1E, F).

**TABLE 1:** Association of *NCAPD3* with clinicopathological characteristics from 67 gastric cancer patients.

Characteristic	Total N (%)	*NCAPD3* expression level, N (%)		χ^2^	*p*-value
		Low expression	High expression		
Age (years)					
≥60	39 (58.2)	19 (55.9)	20 (60.6)	0.021	0.109
<60	28 (41.8)	15 (44.1)	13 (39.4)		
Gender					
Male	42 (62.7)	24 (70.6)	18 (54.5)	1.220	0.269
Female	25 (37.3)	10 (29.4)	15 (45.5)		
pT status					
T_1_	18 (26.9)	14 (41.2)	4 (26.9)	9.058	0.029
T_2_	10 (14.9)	6 (17.6)	4 (14.9)		
T_3_	20 (29.8)	7 (20.6)	13 (29.9)		
T_4_	19 (28.4)	7 (20.6)	12 (28.4)		
pN status					
N_0_	17 (25.4)	13 (38.2)	4 (12.1)	4.731	0.029
N_1-4_	50 (74.6)	21 (61.8)	29 (87.9)		
pTNM stage					
Ⅰ+Ⅱ	35 (52.2)	25 (73.5)	10 (30.3)	10.689	<0.001
Ⅲ	32 (47.8)	9 (26.5)	23 (69.7)		
Pathobiologic categories					
Signet ring cell carcinoma	3 (4.7)	3 (8.8)	0	3.048	0.239
Adenocarcinoma	64 (95.3)	31 (91.2)	33 (100)		
Degree of differentiation					
Moderate/high	38 (56.7)	16 (47.1)	22 (66.7)	1.885	0.170
Undifferentiated/low	29 (43.3)	18 (52.9)	11 (33.3)		

### 3.2 *NCAPD3* overexpression promotes the malignant phenotype of GC cells

**FIGURE 2:**
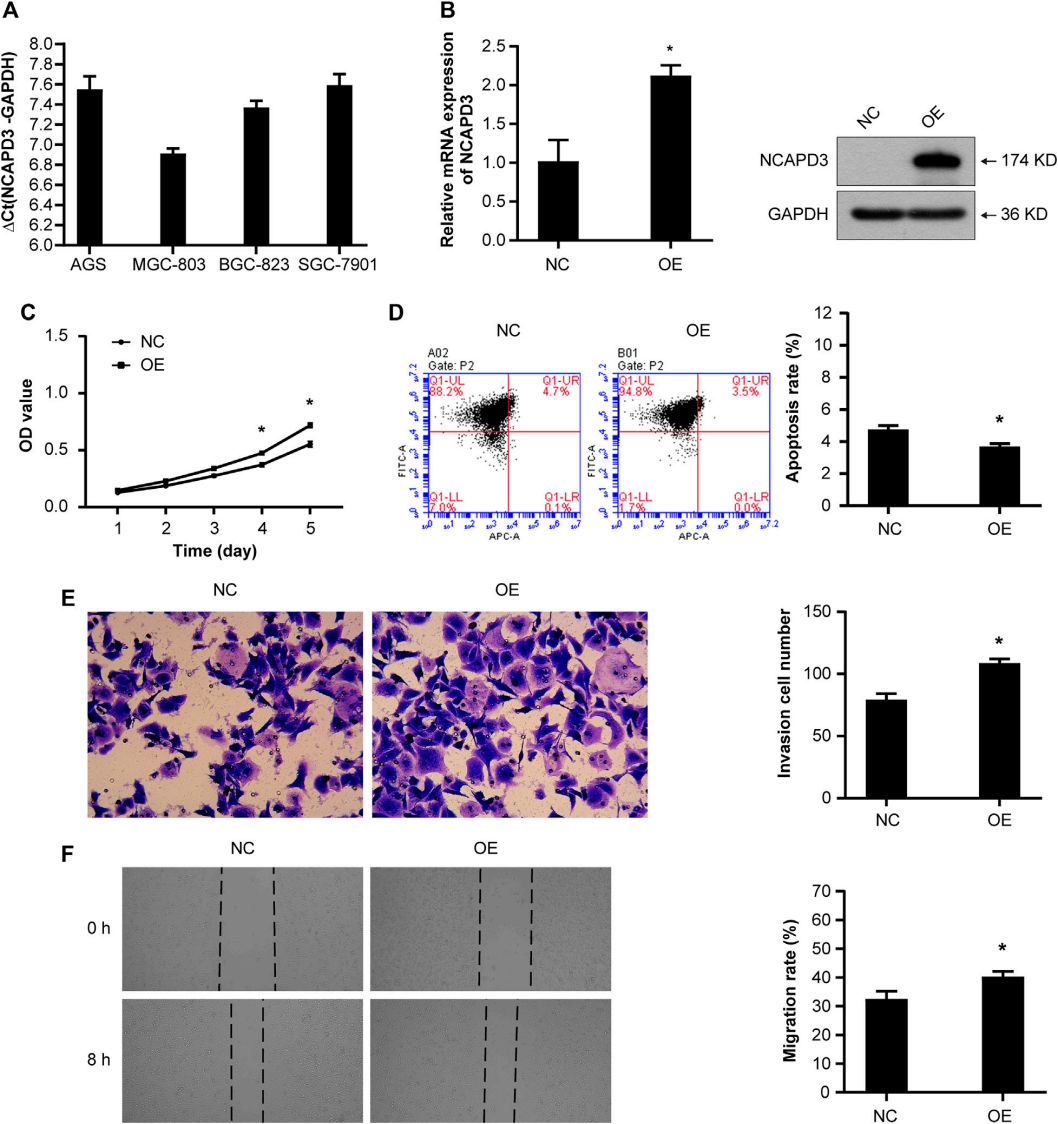
*NCAPD3* overexpression promotes the malignant phenotype of GC cells. **(A)** *NCAPD3* expression in four gastric cancer cell lines (AGS, BGC823, MGC803, and SGC7901) determined by RT-qPCR. The larger the ΔCt_(NCAPD3-GAPDH)_, the lower the abundance of mRNA expression in cells: ΔCt≤12, high abundance; 12<ΔCt <16, medium abundance; ΔCt ≥16, low abundance. **(B)** mRNA and protein expression following *NCAPD3* overexpression in AGS cells examined by RT-qPCR and Western blot analysis. *NCAPD3* protein detected here was exogenous. **(C)** The proliferation of AGS cells was measured by MTT assay and presented as OD490 nm absorbance. **(D)** AnnexinV-APC apoptosis assay was applied to measure the effects of *NCAPD3* overexpression on apoptosis in AGS cells. **(E)** The invasive potentials of AGS cells were determined by Transwell assays. **(F)** Scratch assays were performed in AGS cells to measure the effects of *NCAPD3* overexpression on migration. NC: negative control. OE: *NCAPD3* overexpression. *p < 0.05.

To examine the function of *NCAPD3* in gastric cancer, first, we measured *NCAPD3* expression in four cancer-derived GC cell lines (AGS, BGC823, MGC803, and SGC7901) by RT-qPCR and found that the expressions of NCAPD3 mRNA in these four gastric cancer were all high-abundence (Supplementary Material 1). Additionally, AGS cells had a lower level of *NCAPD3* mRNA relative to the other cells, while MGC803 cells expressed *NCAPD3* at a much higher level (Figure 2A). Therefore, AGS and MGC803 cells were selected for the subsequent studies.

Then we established the stable AGS *NCAPD3* overexpression cell line by lentiviral transduction. As shown in Figure 2B, *NCAPD3* mRNA and protein expressions were significantly increased in cells transfected with *NCAPD3* virus (oe*NCAPD3*) compared with negative control virus (NC). Proliferative and apoptosis abilities were evaluated using MTT assays and flow cytometry assays, respectively. Indeed, the overexpression of *NCAPD3* enhanced the viability and reduced the apoptosis of GC cells (Figures 2C, D). The potentials of migration and invasion were also promoted by overexpression of NCAPDS in AGS cells. These effects were validated by Transwell invasion assays and scratch assays (Figures 2E, F).

### 3.3 Silencing *NCAPD3* inhibits GC cell malignant biological behaviors

**FIGURE 3:**
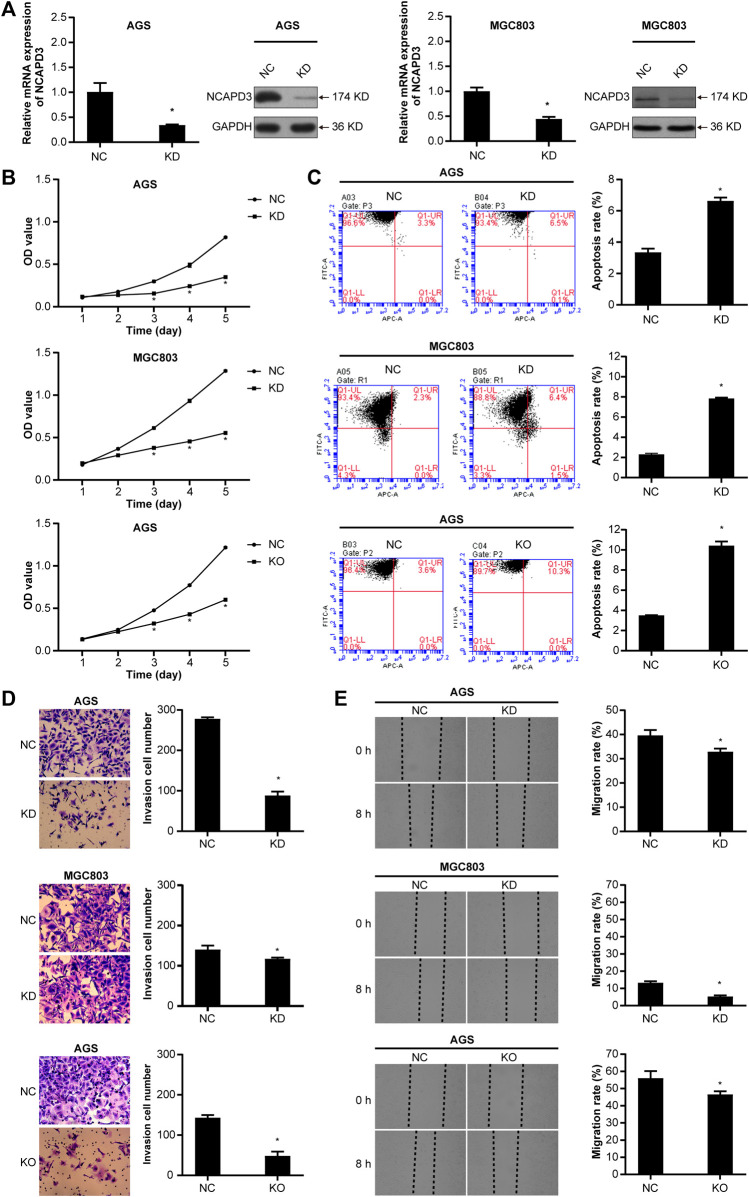
Silencing *NCAPD3* inhibits gastric cancer cell malignant biological. **(A)** RT-PCR and WB were performed to assess knockdown results. **(B)** MTT was used to measure the effects of *NCAPD3* knockdown or knockout on gastric cancer cell proliferation. **(C)** AnnexinV-APC apoptosis assay was conducted to measure the effects of *NCAPD3* knockdown or knockout on apoptosis in gastric cancer cells. **(D)** Transwell invasion assay was applied to assess the effects of *NCAPD3* knockdown or knockout gastric cancer cell invasion. **(E)** The migratory potentials of *NCAPD3*-knockdown/knockout AGS and MGC803 cells were determined by Scratch assays. NC: negative control. KD: *NCAPD3* knockdown. KO: *NCAPD3* knockout. *p < 0.05.

To further determine the oncogenic properties of *NCAPD3* in GC, we used specific short hairpin RNAs (shRNAs) to transfect AGS and MGC803 cells. As demonstrated by RT-qPCR and Western blotting assays, *NCAPD3* expression was successfully suppressed in both sh*NCAPD3*-treated AGS and MGC 803 cells (Figure 3A). Then the Effects of *NCAPD3* knockdown on malignant biological behaviors of gastric cancer cells were detected by MTT, flow cytometry assays, Transwell invasion assays and scratch assays. As shown in Figures 3B–E, *NCAPD3* silencing significantly inhibited proliferation, enhanced apoptosis, and impaired the invasion and migration abilities of GC cells. The results were further verified by the CRISPR/Cas9 technology, which was used to construct AGS cell with stable *NCAPD3* knockout (Supplementary Figure S1).

### 3.4 Effects of *NCAPD3* knockdown on MGC803 gastric cancer subcutaneous xenografts in nude mice

**FIGURE 4:**
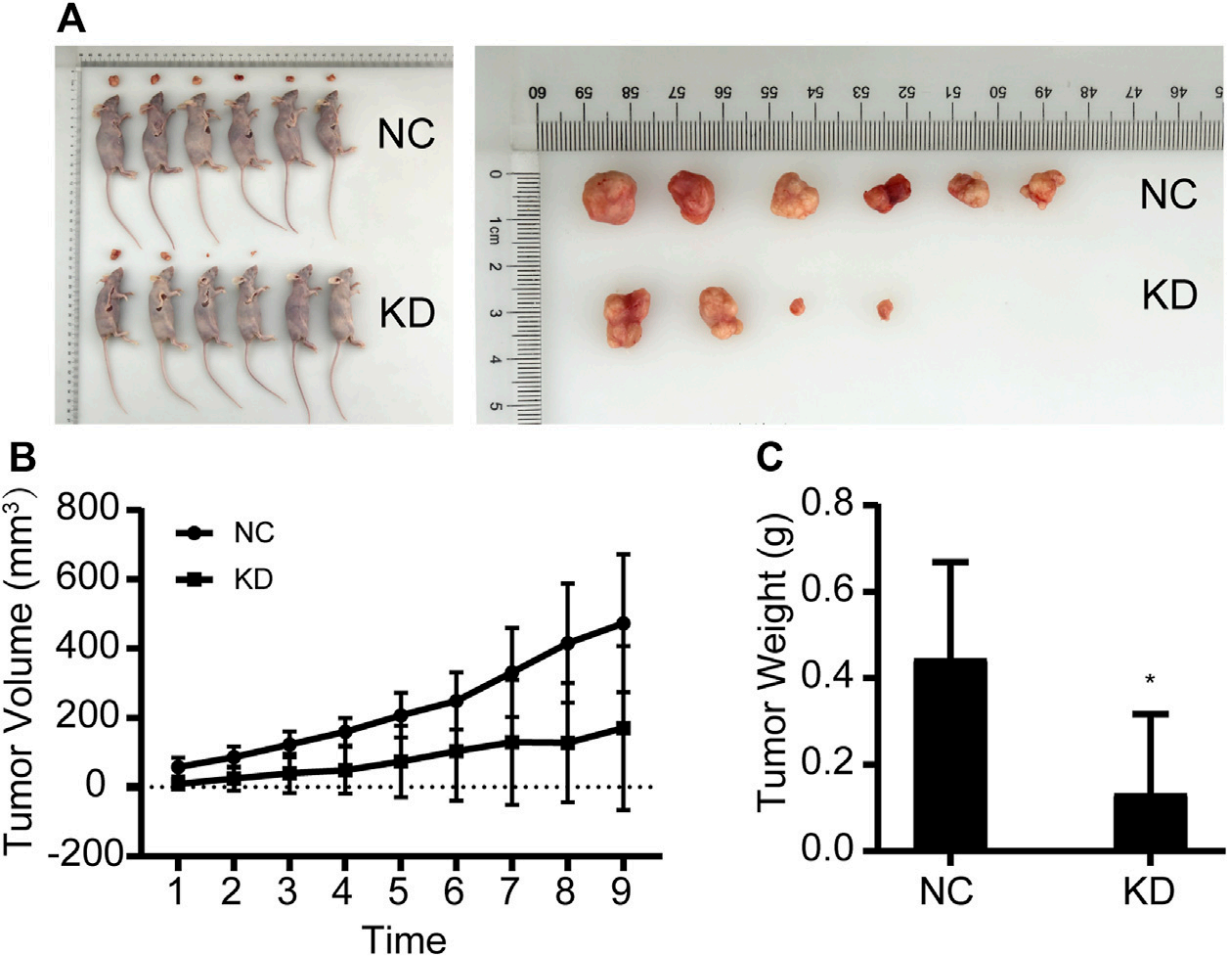
Effects of *NCAPD3* knockdown on BGC823 gastric cancer subcutaneous xenografts in nude mice. **(A)** Macroscopic appearance of representative tumor specimens at autopsy. **(B)** Tumor growth curves for negative control (NC) group and *NCAPD3* knockdown (KD) group mice. **(C)** Effect of *NCAPD3* knockdown on tumor weight. Data are mean ± SD of 6 animals per group. NC: negative control. KD: *NCAPD3* knockdown. *p < 0.05.

MGC803 gastric cancer cells that were transfected with the empty lentivirus vector (negative control) and *NCAPD3*-shRNA lentivirus vector were inoculated subcutaneously in nude mice, and tumorigenicity was observed (Figure 4). The growth curve of subcutaneous tumor xenografts was plotted based on xenograft volume at different time points. Results showed that the growth speed of the *NCAPD3* knockdown group was significantly lower than the negative control group (*p* < 0.05).

Tumors were harvested and weighed after nude mice were euthanized. Results showed that the tumor weight in the *NCAPD3* knockdown group was significantly lower than the negative control group (*p* < 0.05). The tumor weight inhibition rate was 70.21%.

### 3.5 Gene expression spectrum analysis of *NCAPD3* silencing

**FIGURE 5:**
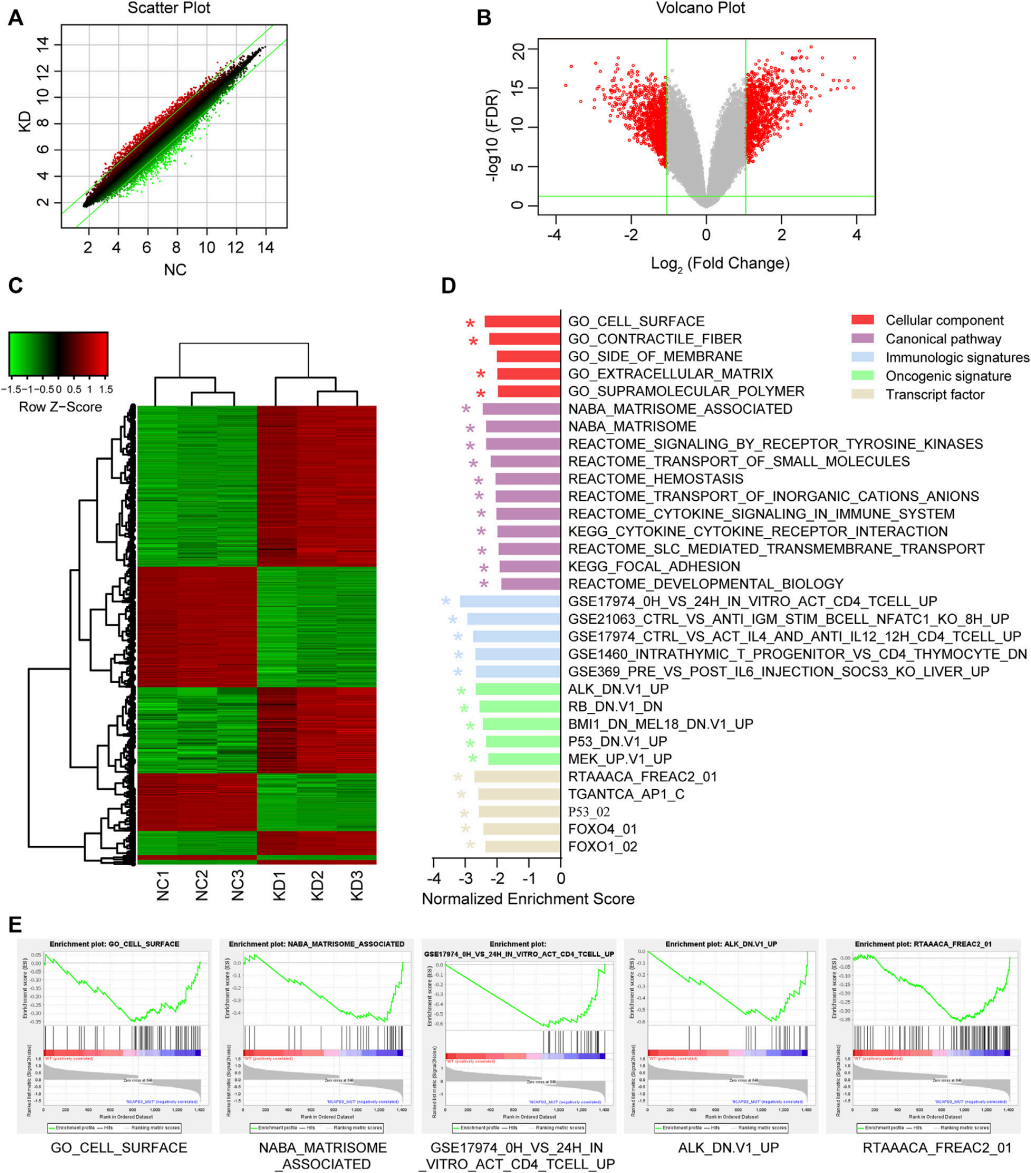
Differential expression analysis and GSEA enrichment results of differentially expressed genes (DEGs) before and after NCAPD3 knockdown. **(A)** Scatter plot. The green line represents the reference line for differential expression, the red dots represent relatively upregulated groups in KD, and the green dots represent upregulated groups in NC. **(B)** Volcano plot. Red represents significant differentially expressed genes, and green represents non-significant differentially expressed genes. **(C)** Cluster analysis heat map. Red represents upregulated gene expression, green represents downregulated gene expression, and black represents no significant change in gene expression. **(D)** The GSEA enrichment results shown in a bar chart. **(E)** Five GESA enrichment plots of typical pathways in the canonical pathway, cellular component, immunologic signatures, oncogenic signature, and transcription factor. NC: negative control. KD: *NCAPD3* knockdown. * FDR <0.05.

In order to study the molecular mechanisms of malignant biological behavior in gastric cancer regulated by *NCAPD3*, GeneChip PrimeView human gene expression array was used to detect differentially expressed genes (DEGs) before and after *NCAPD3* knockdown. The results showed that, after *NCAPD3* expression was inhibited, there were significant differences in the expression levels of 1,411 genes in AGS cells, among which 562 genes were upregulated and 849 genes were downregulated after *NCAPD3* silencing (Figures 5A, B). Hierarchical clustering was used to analyze the expression of differentially expressed genes. Results showed that there were significant changes in gene expression upregulation and downregulation (Figure 5C).

In order to examine changes in overall biological processes and pathways after *NCAPD3* knockdown, GSEA analysis was performed on the cellular component, canonical pathway, immunologic signatures, oncogenic signature, and transcription factor (Figures 5D, E). Compared with the non-knockdown group, five significant enrichment results were obtained in the cellular component GSEA after *NCAPD3* knockdown, among which cell surface sets were significantly enriched. A total of 11 significant signaling pathways were obtained from the canonical pathway analysis, such as signaling by receptor tyrosine kinases, cytokine signaling in the immune system, and cytokine-cytokine receptor interaction. Immunologic signatures enrichment analysis showed that 119 immune gene sets were significantly enriched in the *NCAPD3* knockdown group, showing that *NCAPD3* significantly activates immune-related gene sets; this result is consistent with the canonical pathway enrichment analysis results. Twenty-six significantly enriched oncogene sets were obtained from oncogenic signature enrichment analysis, and the top five significantly enriched gene sets include ALK, RB, BMI1, P53, and MEK. Thirteen significant enrichment results were obtained from transcription factor analysis. Figure 5D presents the top five significantly enriched transcription factor gene sets, such as P53, FOXO4, and FOXO1. These results showed that *NCAPD3* knockdown affects many signaling pathways and biological processes. Furthermore, *NCAPD3* can affect the expression of oncogenes and transcription factors to affect disease occurrence and development.

### 3.6 IPA analysis of differentially expressed genes relative to classical pathways, upstream regulators, disease and function, and regulatory effect

**FIGURE 6:**
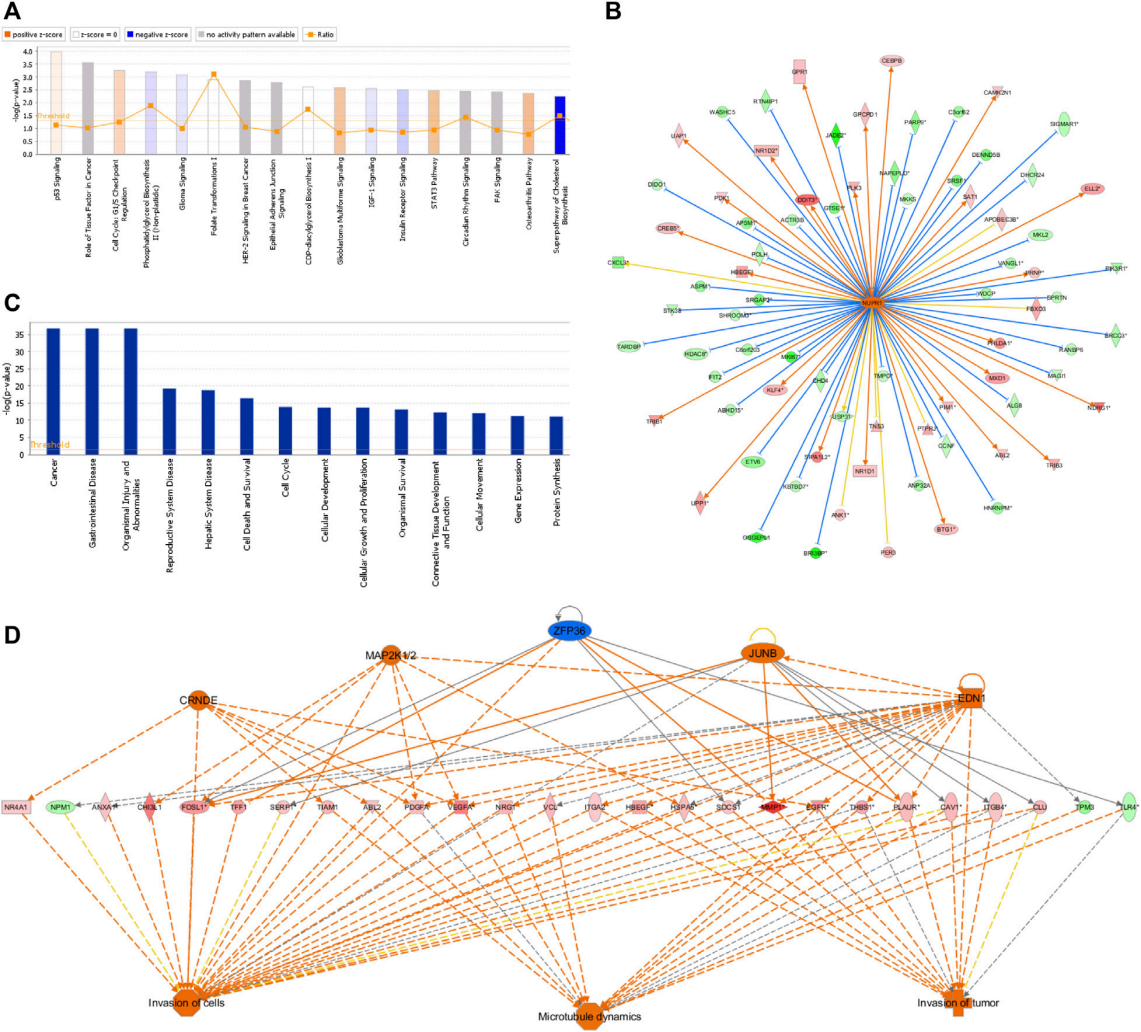
IPA analysis results. **(A)** Canonical pathway enrichment analysis statistical chart. Orange represents pathway activation and blue represents pathway inhibition. **(B)** Network map of upstream regulatory factors of NUPR1. An orange line means that the upstream regulatory factor and gene were consistently activated, a blue line means that the upstream regulatory factor and gene were consistently inhibited, and a yellow line means that the expression trend of the upstream regulatory factor and gene were not consistent. **(C)** Disease and functional enrichment statistical graph. **(D)** Interactions between genes and regulatory factors and functions.

The IPA platform was used to analyze differentially expressed genes in the negative control (NC) group and *NCAPD3* knockdown (KD) group. The canonical pathway analysis results showed that there were 10 signaling pathways that showed differences after *NCAPD3* knockdown in the KD group. Pathways that were mainly activated were the p53 signaling, G1/S cell cycle checkpoint, and STAT3 pathways. Pathways that were inhibited mainly included the phosphatidylglycerol biosynthesis II (non-plastidic), IGF-1 signaling, and superpathway of cholesterol biosynthesis, among which the cholesterol biosynthesis pathway was significantly inhibited, with a Z-score of −2.449 (Figure 6A).

In order to determine the major upstream regulatory factors and explain differential gene expression between the two groups, upstream regulatory factor analysis was carried out. This analysis determined the number of known targets for each regulatory factor, and their direction of change was compared with that found in past papers. One hundred forty-five upstream regulators (including transcription factors, small RNAs, cytokines, kinases, and chemical molecules and drugs) were identified as activators, and 45 upstream regulators were predicted as suppressors. The prediction results for top 10 activated or inhibited upstream regulatory factors were listed (Table 2), among which the transcription factor NUPR1 was predicted to be significantly activated (Z-score = 2.252). Then an interaction network between the NUPR1 upstream regulatory factor and its downstream factors was constructed (Figure 6B). Among these genes, *ELL2*, *NDRG1*, *HBEGF*, *SAT1*, *NR1D1*, *PDK1*, and *DDIT3* were elevated, whereas *PARP9*, *NAPEPLD*, *MKKS*, *PIK3R1*, *MAGI1*, *HDAC8*, *ASPM* were decreased (Figure 6B).

**TABLE 2:** Predicted upstream regulators for all differentially expressed genes between *NCAPD3*-knockdown and negative control AGS cells (top 10).

Upstream regulator	Molecule type	Predicted state	Activation z-score	*p*-value
NUPR1	transcription regulator	Activated	7.02	8.66E-15
ATF4	transcription regulator	Activated	5.409	4.2E-13
TNF	cytokine	Activated	4.612	5.14E-10
Ca2+	chemical - endogenous mammalian	Activated	4.225	0.000156
lipopolysaccharide	chemical drug	Activated	4.14	0.00000545
MMP3	peptidase	Inhibited	−3.441	0.00000136
PD98059	chemical - kinase inhibitor	Inhibited	−3.287	1.98E-11
TAL1	transcription regulator	Inhibited	−3.185	0.0809
MGEA5	enzyme	Inhibited	−3.153	0.000181
HDL-cholesterol	complex	Inhibited	−2.879	0.00000108

In the disease and functional analysis, the first analysis showed significantly enriched differentially expressed genes in disease and function, which were mainly enriched in gastrointestinal disease, cell death and survival, cell cycle, cellular development, cellular growth and proliferation, and cellular movement (Figure 6C). This shows that differentially expressed genes that are regulated by inhibiting *NCAPD3* expression mainly participate in cell growth, development, and survival. Regulatory effect analysis was used to investigate differentially expressed genes participating in different cellular functions. Results showed that the regulatory factors CRNDE, EDN1, JUNB, and MAP2K1/2, ZFP36 mainly regulate differentially expressed genes (*VEGFA*, *EGFR*, *PLAUR*) to regulate invasion of cells, invasion of tumor, and microtubule dynamics. For example, the regulatory factor CRNDE regulates the *EGFR* gene to regulate invasion of cells, invasion of tumor, and microtubule dynamics. The EDI regulatory factor regulates invasion of cells, invasion of tumor, and microtubule dynamics through the *PLAUR* gene (Figure 6D).

### 3.7 Molecular mechanism study on inhibition of tumor cell proliferation and promotion of tumor cell apoptosis due to *NCAPD3* deletion

**FIGURE 7:**
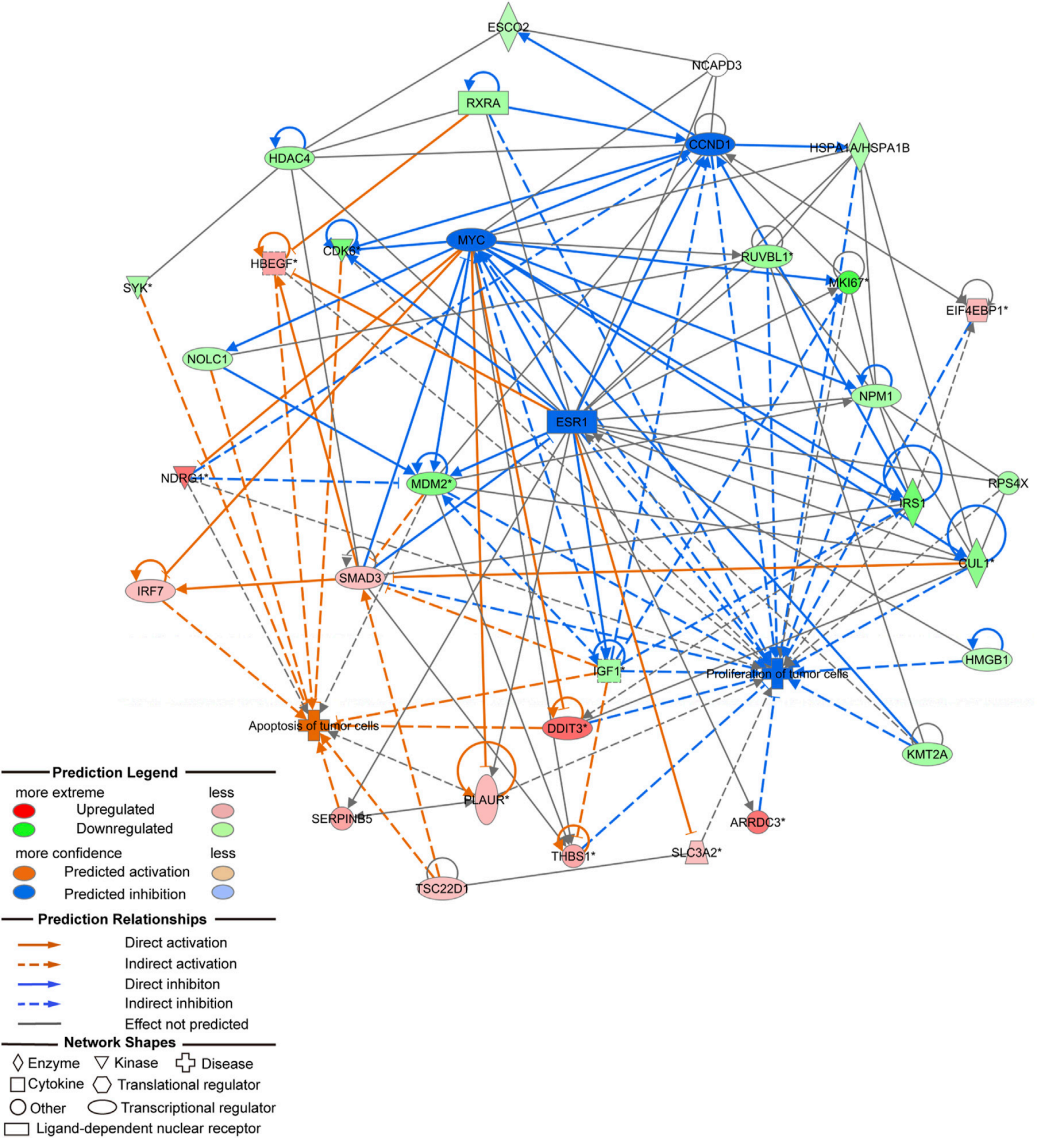
Molecular regulatory network map of NCAPD3 knockdown on inhibition of tumor cell proliferation and promotion of tumor cell apoptosis.

*In vivo* and *in vitro* experiments showed that *NCAPD3* loss can significantly inhibit gastric cancer cell proliferation, invasion, and migration, and promote apoptosis. In order to further study the effector molecular mechanisms, the *NCAPD3* knockdown differentially expressed gene data in this study were combined with literature data, and IPA was employed to construct a molecular regulatory network map of tumor cell proliferation and apoptosis. In the molecular regulatory network map in which *NCAPD3* knockdown inhibits tumor cell proliferation and promotes tumor cell apoptosis, genes related to tumor cell proliferation pathways showed overall decrease or downregulation, among which *CCND1*, *MYC*, *ESR1* were significantly decreased and their downstream genes *CDK6* and *IRS1* were significantly downregulated. Therefore, *NCAPD3* knockdown may inhibit CCND1, MYC, and ESR1 activity to downregulate CDK6 and IRS1 expression, thereby inhibiting the proliferation of gastric cancer cells. Additionally, genes associated with tumor cell apoptosis pathways showed overall activation, among which *IRF7*, *DDIT3*, and *HBEGF* were significantly activated. Therefore, *NCAPD3* knockdown may activate IRF7, DDIT3, and HBEGF expression to promote gastric cancer cell apoptosis (Figure 7).

**FIGURE 8:**
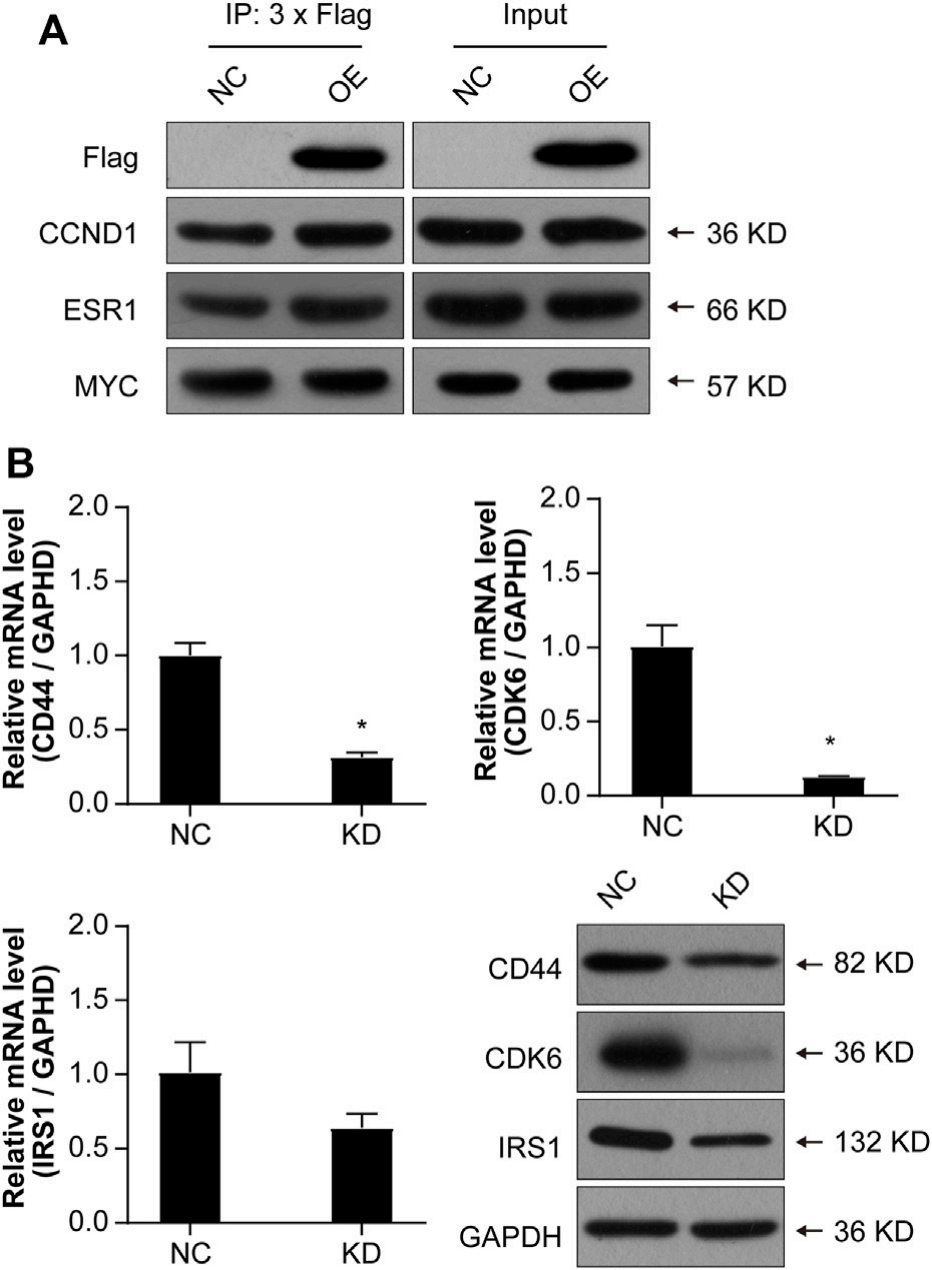
Validations of the possible interactions and downstream target genes. **(A)** Co-IP was used to validate interactions. FLAG is a protein tag used to indicate NCAPD3 protein. **(B)** Validations of three target genes by qPCR and WB. NC: negative control. OE: NCAPD3 overexpression. KD: NCAPD3 knockdown. *p < 0.05.

In order to further validate whether *NCAPD3* directly targets CCND1, ESR1, and MYC to regulate gastric cancer cell proliferation, Co-IP experiments were conducted to examine if *NCAPD3* directly interacts with CCND1, ESR1, and MYC. The results showed that *NCAPD3 NCAPD3* protein may not directly interact with MYC but may directly interact with CCND1 and ESR1 (Figure 8A).

Based on the interaction network analysis, 29 differentially expressed genes (DEGs) of interest were selected for qPCR validation. The validation results showed that the gene chip variation trends of 22 genes were consistent with PCR variation trends (Table 3). From these 22 genes, three (CD44, CDK6, and IRS1) were selected for further WB validation (Figure 8B). The protein expression levels of CD44, CDK6, and IRS1 were downregulated by 32.30%, 84.91%, and 40.50%, respectively. Based on the above experimental and bioinformatics analysis results, the target gene *NCAPD3* may upregulate CD44, CDK6, and IRS1 in AGS gastric cancer cells to carry out its effects. In summary, the *NCAPD3* protein may target CCND1 or ESR1 to downregulate downstream factors such as CDK6 and IRSI to inhibit gastric cancer cell proliferation.

**TABLE 3:** The qPCR validation of 29 differentially expressed genes (DEGs).

DEGs	Change (Genechip)	Change (Q-PCR)
PRKACB	down	down, *p* < 0.05
RRAS	up	No significant change, *p* > 0.05
TNFRSF1A	up	No significant change, *p* > 0.05
UBE2N	down	down, *p* < 0.05
PIK3R1	down	No significant change, *p* > 0.05
CREBBP	down	down, *p* < 0.05
TNFAIP3	up	up, *p* < 0.05
BMPR2	down	down, *p* < 0.05
IL33	up	up, *p* < 0.05
FADD	down	down, *p* < 0.05
TLR4	down	down, *p* < 0.05
PIPK1	down	down, *p* < 0.05
IRS1	down	down, *p* < 0.05
GSK3B	down	up, *p* < 0.05
FRS2	down	up, *p* < 0.05
IRAK4	down	down, *p* < 0.05
ATM	up	up, *p* < 0.05
SMAD3	down	up, *p* < 0.05
CDK6	down	down, *p* < 0.05
MDM2	down	up, *p* < 0.05
CDKN2D	up	up, *p* < 0.05
HDAC4	down	down, *p* < 0.05
PA2G4	down	down, *p* < 0.05
HDAC8	down	down, *p* < 0.05
PAK1IP1	down	down, *p* < 0.05
TCF4	down	down, *p* < 0.05
CD44	down	down, *p* < 0.05
MAP1LC3B	up	up, *p* < 0.05

## 4 Discussion

Gastric cancer occurrence and progression is an extremely complex process involving oncogenes, tumor suppressor genes, cell cycle regulatory factors, and signal regulatory factors. In cancer, somatic cell changes in signaling pathways occur at different frequencies and combinations in different organs and tissues, showing complex interactions and pathway interference (Ajani et al., 2016). Even though conventional gastric cancer treatments such as surgery, chemotherapy, and radiotherapy have contributed to improvements in cancer treatment, disease recurrence is common in most gastric cancer patients (Hayakawa et al., 2015). The reason for this is because the pathogenesis of gastric cancer is still unclear. Therefore, in-depth studies on gastric cancer pathogenesis and discovery of new precision medicine targets are especially important.

*NCAPD3* plays a crucial role in chromosome structural changes and separation during mitosis in eukaryotic cells. Previous studies found that NCAPD2/3 is intimately associated with the occurrence of many diseases (Seipold et al., 2009; Zhang et al., 2014). Thadani et al. found that condensin II complex dynamics regulate the cell cycle (Thadani et al., 2018). Yin et al. (2017) found that high expression of the *NCAPD3* homologous complex, NCAPH, promotes proliferation in colon cancer cells. Kim et al. (2020) reported that knocking out the *NCAPD3* homologue, *NCAPH*, inhibits the proliferation, colony formation, invasion, and migration of non-small cell lung cancer cells, showing that NCAPH participates in regulating non-small cell lung cancer occurrence and progression. NCAPG is a *NCAPD3* homologous complex and also a subunit of the condensin complex. Studies showed that NCAPG participates in many tumors, including prostate cancer (Goto et al., 2017), high-grade glioma in children (Liang et al., 2016), renal cell carcinoma (Yamada et al., 2018), multiple myeloma (Cohen et al., 2014), and melanoma (Ryu et al., 2007). Studies found that NCAPG is crucial for liver cancer occurrence and progression (Zhang et al., 2018). NCAPG can activate the PI3K/AKT/FOXO4 pathway to promote liver cancer proliferation and inhibit apoptosis (Gong et al., 2019). Zhang et al. found that *NCAPG* overexpression inhibits cardia adenocarcinoma apoptosis and promotes epithelial-mesenchymal transition (Zhang et al., 2021). However, the correlation between *NCAPD3* and gastric cancer occurrence and progression is still not clear. Therefore, this study aimed to evaluate the role of *NCAPD3* in gastric cancer cells. The results indicated that *NCAPD3* is highly expressed in gastric cancer cells and clinical tissue specimens and is intimately associated with prognosis, suggesting that high *NCAPD3* expression may be the key to gastric cancer occurrence and progression. In addition, cell experiments demonstrated that *NCAPD3* overexpression promotes gastric cancer cell proliferation, invasion, and migration and inhibit apoptosis. Conversely, inhibiting *NCAPD3* expression attenuates gastric cancer cell proliferation, invasion, and migration and promote apoptosis. Furthermore, *in vivo* animal experiments showed that *in vivo* tumor growth is inhibited after *NCAPD3* knockdown. This shows that *NCAPD3* may affect cell proliferation, invasion, migration, and apoptosis to affect gastric cancer occurrence and progression. In summary, this study showed that *NCAPD3* may be a crucial factor in gastric cancer occurrence and progression.

In order to examine changes in overall biological processes and related signaling pathways after *NCAPD3* knockdown, GSEA was carried out. The results showed that cytokine signaling in the immune system and cytokine-cytokine receptor interaction were significantly enriched after *NCAPD3* knockdown, indicating that *NCAPD3* knockdown may affect immune-related signaling pathways. This finding was proven by the immunologic signature enrichment analysis, the results of which showed that 119 immune gene sets were significantly enriched in the *NCAPD3* knockdown group. The tumor microenvironment is beneficial for cancer cell growth and expansion. Many types of cells participate in the tumor microenvironment, such as inflammatory cells, fibroblasts, neurons, and vascular endothelial cells. These matrix cells secrete various factors to directly activate cancer cell growth signals or remodel surrounding regions, thereby promoting tumor growth. Endothelial cells not only provide nutrients to tumors but also secrete chemokines or cytokines to interact with cancer stem cells and immune cells (Oya et al., 2020). Tumor-associated immune cells have tumor suppression or tumor-promoting functions. In immune cell populations, tumor-associated macrophages, such as M1 and M2 macrophages and myeloid-derived suppressor cells, have been reported to secrete soluble factors or regulate immune responses to directly or indirectly promote gastric cancer occurrence (Oya et al., 2020). Patients whose tumors show high T cell infiltration, particularly cytotoxic CD8^+^ T cells and memory T cells, have longer disease-free survival and overall survival, whereas patients with high neutrophil infiltration in tumors have poor prognosis (Zeng et al., 2019). When one or more cells start to show uncontrollable growth, the cancer will develop. This may be the result of changes in highly regulated processes in normal cell division. These changes may be caused by germline or somatic mutations controlling normal cell proliferation, resulting in an oncogene. GSEA analysis revealed that 26 oncogene sets and 13 transcription factor gene sets were significantly enriched after *NCAPD3* knockdown, such as p53 and FOXO1/4. *TP53* (p53) is the most common gene mutated in human cancers and *TP53* mutations are present in approximately 50% of invasive tumors. Traditionally, p53 is regarded as a gene that induces cell cycle arrest, apoptosis, or senescence or participates in DNA repair to inhibit oncogenesis and cancer progression. Although these tumor suppressor mechanisms have been confirmed in different models, recent data show that p53 can also regulate metabolism, regulate reactive oxygen species (ROS) levels, change non-coding RNA expression, and increase autophagy or ferroptosis to inhibit oncogenesis. Due to its high mutation frequency and crucial role in oncogenesis/cancer progression, p53 is a priority target in antineoplastic treatment (Duffy et al., 2017). Regulation of the FOXO transcription factor mainly occurs at the post-transcriptional and post-translational levels, which are mediated by non-coding RNAs through interactions with other protein chaperones and cofactors (including phosphorylation, acetylation, methylation, and ubiquitination). FOXO regulates factors essential for cell proliferation, death, senescence, angiogenesis, migration, and metastasis and plays a role in tumorigenesis and tumor progression (Jiramongkol and Lam, 2020). These findings showed that *NCAPD3* affects the expression of oncogenes and transcription factors to regulate gastric cancer cell proliferation, invasion, migration, and apoptosis.

In order to further study the potential molecular mechanisms by which *NCAPD3* regulates behavioral changes in gastric cancer cells, in this study, the human genome expression chip was used to detect differentially expressed genes and related signaling pathways before and after *NCAPD3* knockdown. IPA canonical pathway analysis showed that the cholesterol biosynthesis pathway is significantly inhibited after *NCAPD3* knockdown. There is controversy over the role of cholesterol in cancer progression and potential treatments targeting cholesterol homeostasis in oncology (Kuzu et al., 2016). One study reported changes and mutations in genes in the cholesterol homeostasis pathway in cancer cells (Murai, 2015). The expression of cholesterol synthesis genes is upregulated, LDL receptor-mediated cholesterol influx is increased, and cholesterol transport is decreased, which increases cellular cholesterol levels, thereby facilitating cancer cell proliferation (Llaverias et al., 2011; Smith and Land, 2012; Krycer and Brown, 2013). In sarcoma, acute myeloid leukemia, and melanoma, increased activity in the cholesterol synthesis pathway is related to decreased patient survival (Riganti and Massaia, 2013; Sui et al., 2015; Brown et al., 2016). However, there are still very few studies in this area, and further studies are required to comprehensively analyze the consequences of these changes and how they regulate cancer progression. This study employed IPA bioinformatics analysis and found that the cholesterol biosynthesis pathway was significantly inhibited after *NCAPD3* silencing. Therefore, *NCAPD3* downregulation may inhibit cholesterol synthesis, thereby affecting gastric cancer cell proliferation.

Regulatory effect analysis revealed that the biological functions of differentially expressed genes were mainly concentrated in cell invasion and tumor invasion. The expression levels of regulatory factors related to cell and tumor invasion (CRNDE, EDN1, JUNB, MAP2K1/2) were significantly activated, among which the upstream regulatory factors CRNDE and EDN1 activated the expression of the downstream target EGFR to promote cell and tumor invasion, and JUNB activated PLAUR expression to promote cell and tumor invasion. EGFR is a tyrosine kinase receptor, and binding with RGF promotes cell survival and proliferation (Prenzel et al., 2001). In addition, EGFR signaling aids *in vitro* cell differentiation, invasion, and migration (El-Rehim et al., 2004; Arteaga and Engelman, 2014). Dysregulated EGFR signaling has been observed in many cancers, including breast cancer, colon cancer, and lung cancer (Nautiyal et al., 2012; Matalkah et al., 2016). EGFR can promote breast cancer invasion and migration (Zhao et al., 2018). PLAUR is highly expressed in gastric cancer tissues and promotes gastric cancer invasion (Sandoval-Bórquez et al., 2017). Therefore, the present study suggests that *NCAPD3*-knockdown-induced differentially expressed genes (DEGs) such as CRNDE, EDN1, and JUNB can regulate cell and tumor invasion through EGFR and PLAUR.

In the molecular regulatory network map, in which *NCAPD3* knockdown inhibited tumor cell proliferation and promoted tumor cell apoptosis, genes related to tumor cell proliferation pathways showed overall inhibition or downregulation, among which *CCND1*, *MYC*, and *ESR1* were significantly inhibited and their downstream genes *CDK6* and *IRSI* were significantly downregulated. Therefore, *NCAPD3* knockdown may inhibit CCND1, MYC, and ESR1 expression to downregulate CDK6 and IRSI expression, thereby inhibiting the proliferation of gastric cancer cells. Co-IP was carried out to analyze whether *NCAPD3* directly targets CCND1, MYC, and ESR1. The results showed that *NCAPD3* directly interacted with CCND1 and ESR1 but did not directly interact with MYC. ESR1, estrogen receptor gene, is a ligand-activated transcription factor composed of a DNA binding and a transcriptional activation domain, including the N-terminal ligand independent activation function (AF)-1 and C-terminal ligand-dependent AF-2 domains. The ligand-binding domain (LBD) and DNA binding and hinge domain in the protein core are also located at the C-terminal. Ligand-receptor binding helps co-regulate the recruitment of proteins, including co-stimulation and co-inhibitory factors to regulate many physiological processes, such as tumorigenesis and tumor progression (Martin et al., 2017). ESR1 has been reported to regulate proliferation in liver cancer (Wang et al., 2021), bladder cancer (Ge et al., 2019), progenitor Leydig cells (Oh et al., 2017), and chondrocytes (Liu et al., 2019). This shows that ESR1 plays important roles in cell proliferation. CCND1 is a member of the cell cycle protein family and regulates the cell cycle by activating CDK4/CDK6 (Marschall et al., 2016). Early studies showed that CCND1 and CDK6 are activated in tumor cells and their expressions are upregulated. Therefore, CCND1 and CDK6/CDK4 are potential therapeutic targets for tumors (Malumbres and Barbacid, 2009; Lang et al., 2015). Cyclin-dependent kinase 6 (CDK6) is a member of the threonine-serine kinase subfamily that participates in controlling the cell cycle, thereby controlling cell proliferation (Turner et al., 2019). Targeting CDK6 is a potential pathway for inducing cell cycle arrest and inhibiting tumor cell proliferation (Rader et al., 2013). CDK6 is an important factor that regulates the cell cycle, and overexpression or activation of CDK6 will accelerate the cell cycle and promote cell proliferation, thereby resulting in transformation and promoting gastric cancer occurrence and progression (Ilyin et al., 2003). Increased CDK6 protein expression can be detected in many types of cancer, and reducing CDK6 expression inhibits the growth and proliferation of tumors *in vivo* and *in vitro* (Thammasit et al., 2015). Insulin receptor substrate (IRS1) is an important intracellular signaling protein and an important signaling factor for cell surface receptor activation. IRS1 regulates upstream signals and downstream effectors to regulate cell growth, metabolism, and activation (Jellema et al., 2003). A study reported that IRS1 can promote tumor proliferation (Dearth et al., 2007). IRS1 is a crucial regulatory factor of PI3K in malignant cells and affects tumor cell proliferation (Houghton et al., 2010). A study reported that IRS1 regulates PI3K activity to inhibit gastric cancer occurrence (Baba et al., 2007). Therefore, *NCAPD3* knockdown may inhibit CCND1 and ESR1 expression to downregulate CDK6 and IRSI expression, thereby inhibiting the proliferation of gastric cancer cells.

In addition, molecular network regulatory maps showed that apoptosis pathway-related genes (e.g., *IRF7* and *DDIT3*) in tumor cells showed overall activation after *NCAPD3* inhibition. This is consistent with the result that inhibiting *NCAPD3* expression promotes apoptosis in gastric cancer cells. IRF7 is an interferon regulatory factor that is intimately associated with apoptosis. Liu et al. (2017) found that IRF7 activates NF-κB/GSDMD signals in mouse adipose tissues to promote inflammasome-induced apoptosis. Zhang et al. (2017) reported that the HTLV-1 oncoprotein Tax interacts with MAVS, STING, and RIP1 to inhibit the innate interferon response, resulting in TBK1-mediated inhibition of IRF3/IRF7 phosphorylation, and also to inhibit apoptosis and autophagy in target cells. DDIT3 is also known as C/EBP homologous protein (CHOP) and is a mark of endoplasmic reticulum stress. DDIT3 can form heterodimers with other proteins in the C/EBP family. During endoplasmic reticulum stress, DDIT3 acts as a transcription factor to downregulate the expression of antiapoptotic factors BCL-2 and BCL-Xl, and it acts as a transcription activation factor to upregulate the expression of proapoptotic genes such as *BIM* (Tsukano et al., 2010; Hu et al., 2019). Therefore, *NCAPD3* inhibition may regulate gastric cancer cell apoptosis by activating IRF7 and DDIT3.

## 5 Conclusion

In summary, *NCAPD3* is upregulated in gastric cancer. *NCAPD3* promotes gastric cancer cell proliferation, invasion, and migration and inhibits apoptosis to accelerate gastric cancer progression. Inhibiting *NCAPD3* expression can attenuate the malignant biological behaviors of gastric cancer cells. *NCAPD3* loss can directly inhibit CCND1 and ESR1 expression to downregulate the expression of downstream targets CDK6 and IRS1 and inhibit the proliferation of gastric cancer cells. Moreover, *NCAPD3* loss activates IRF7 and DDIT3 to regulate apoptosis in gastric cancer cells. In addition, *NCAPD3* significantly affects many canonical pathways and immune and transcription factor gene sets in gastric cancer occurrence and progression. Overall, this study shows that *NCAPD3* may be a potential target for gastric cancer treatment.

## Data Availability

The datasets presented in this study can be found in online repositories. The names of the repository/repositories and accession number(s) can be found in the article/Supplementary Material. The expression data presented in the study is publicly available. This data can be found here: Gene Expression Omnibus, accession number GSE261264.
