# Supplementary figures and images for: Role and mechanism of NCAPD3 in promoting malignant behaviors in gastric cancer (part 1 of 2)
Source: Front Pharmacol. 2024 Apr 22;15:1341039. doi: 10.3389/fphar.2024.1341039 (PMC11070777; doi:10.3389/fphar.2024.1341039)

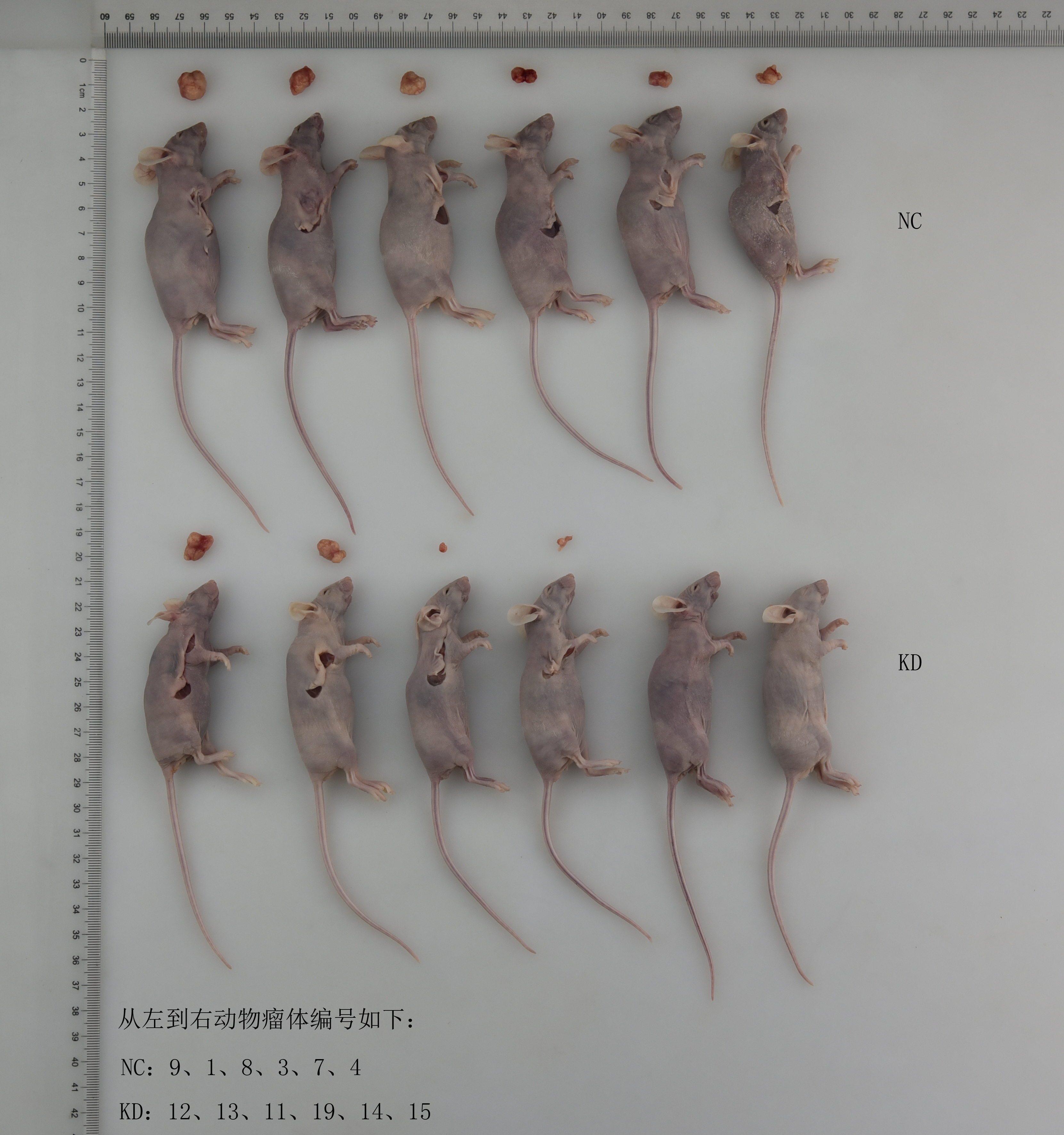

Supplement: Supplementary file 2 [file DataSheet11.ZIP › mice and tumor_6 mice per group.JPG]

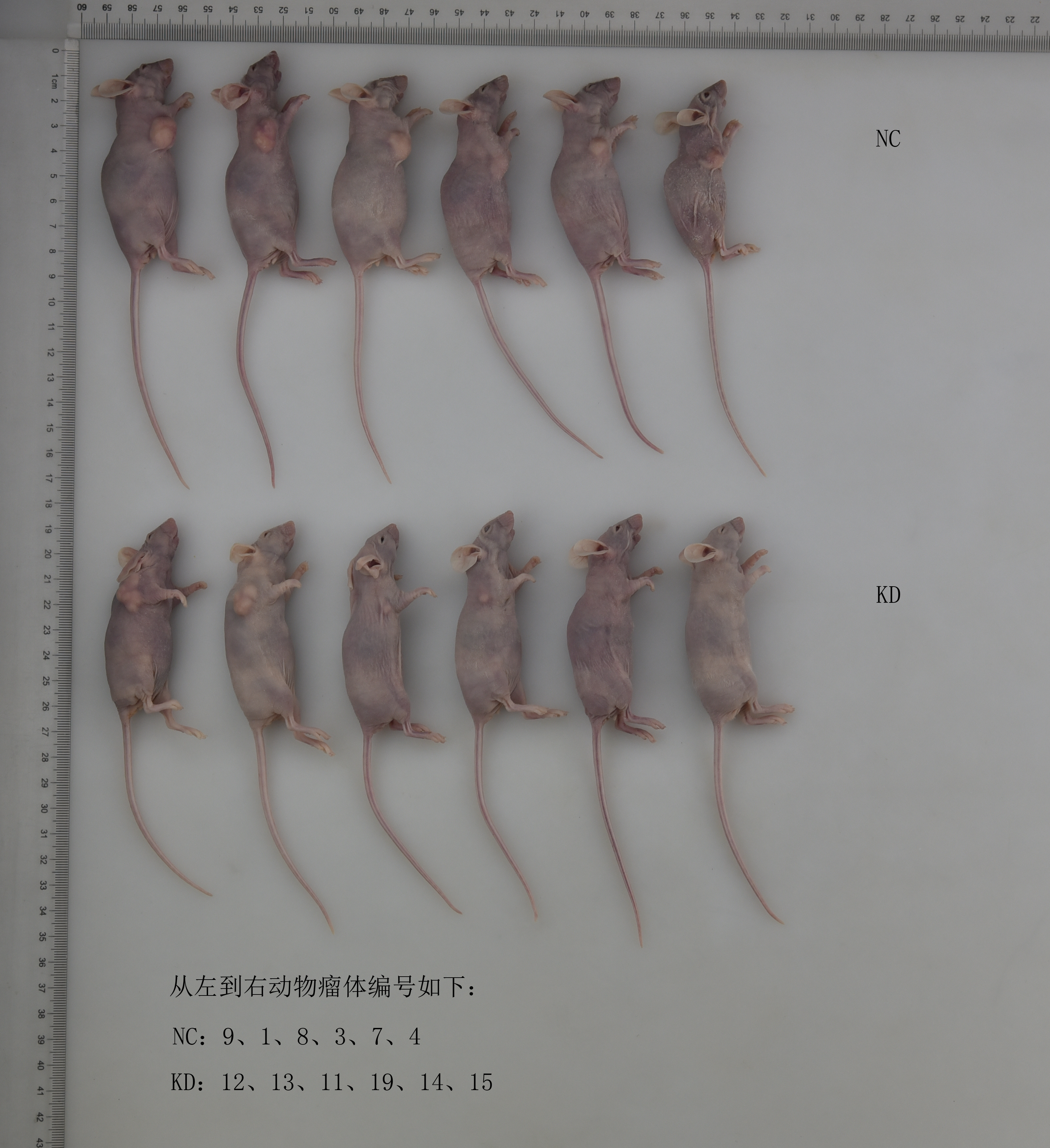

Supplement: Supplementary file 2 [file DataSheet11.ZIP › mice_6 mice per group.JPG]

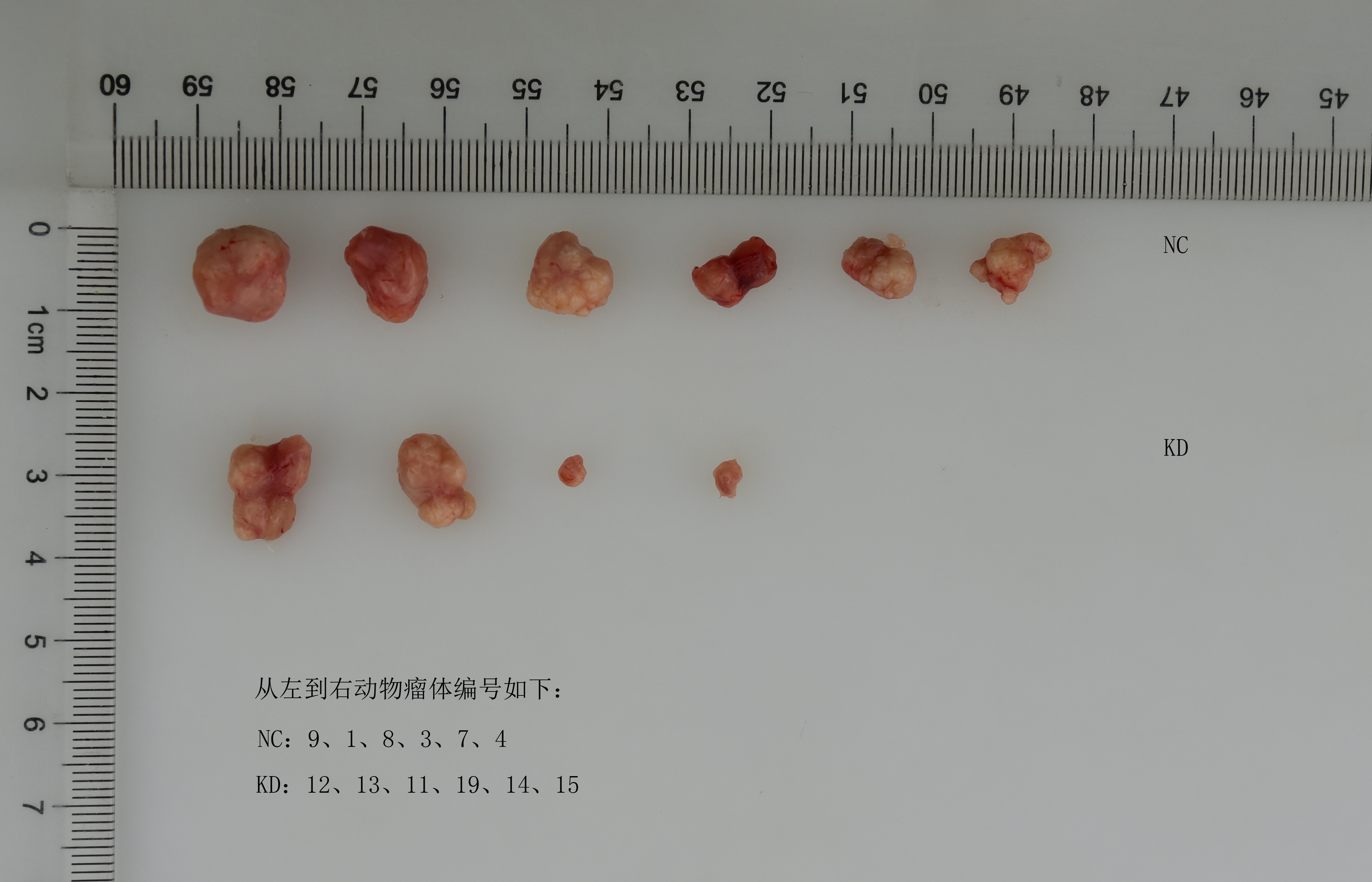

Supplement: Supplementary file 2 [file DataSheet11.ZIP › tumor_6 mice per group.JPG]

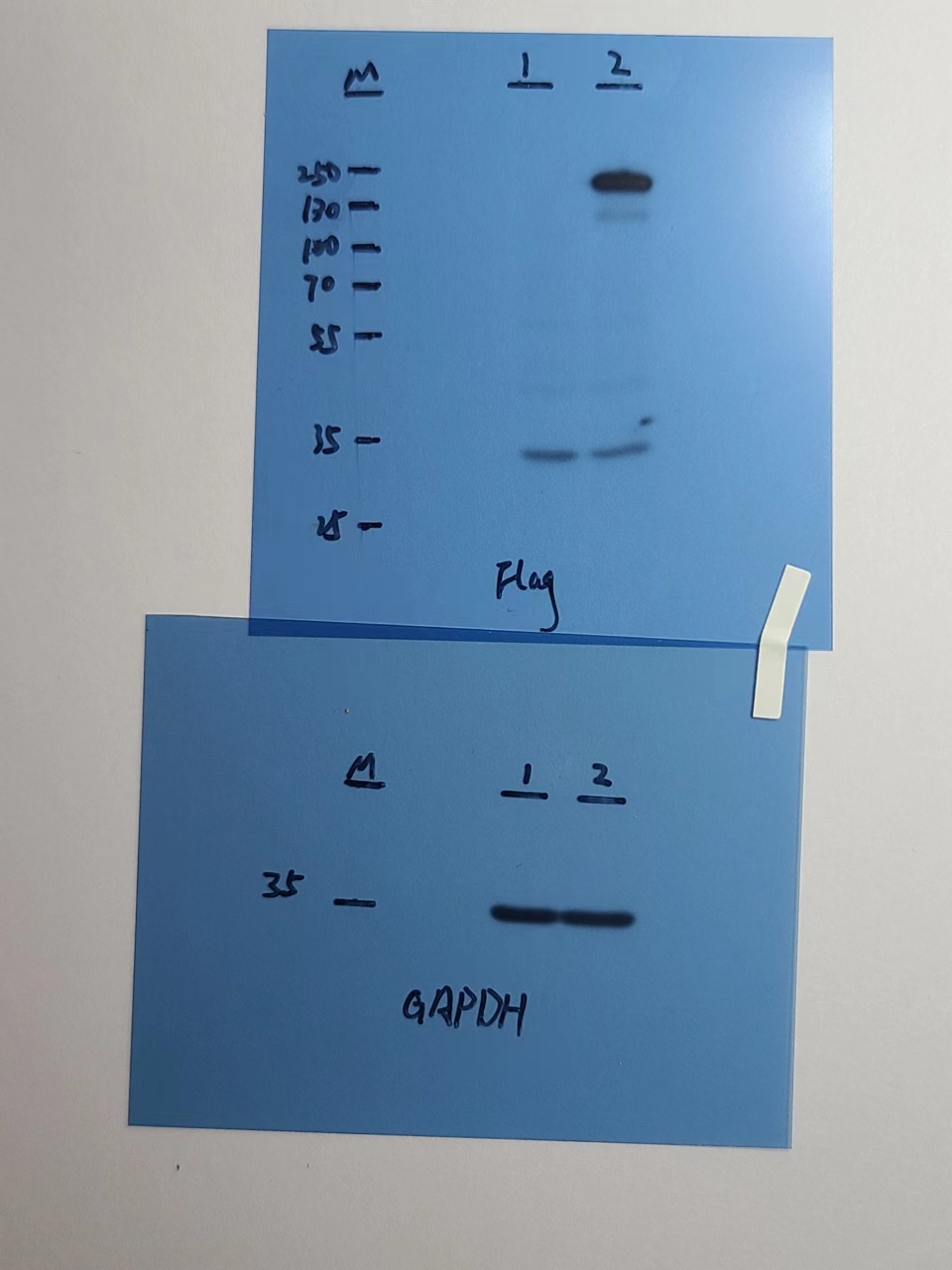

Supplement: Supplementary file 4 [file DataSheet9.ZIP › Figure 2-B2-AGS.jpg]

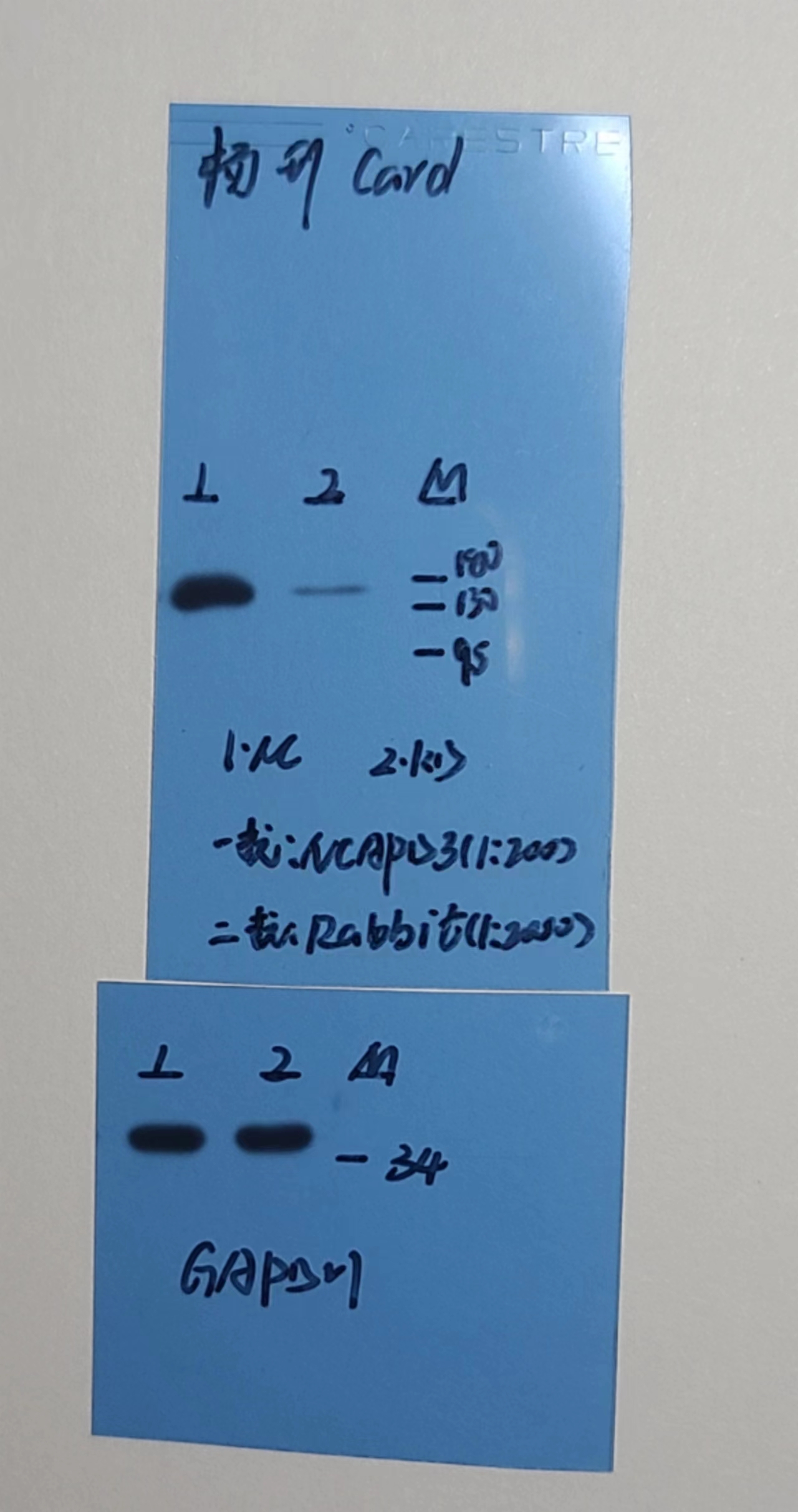

Supplement: Supplementary file 4 [file DataSheet9.ZIP › Figure 3-A2-AGS.jpg]

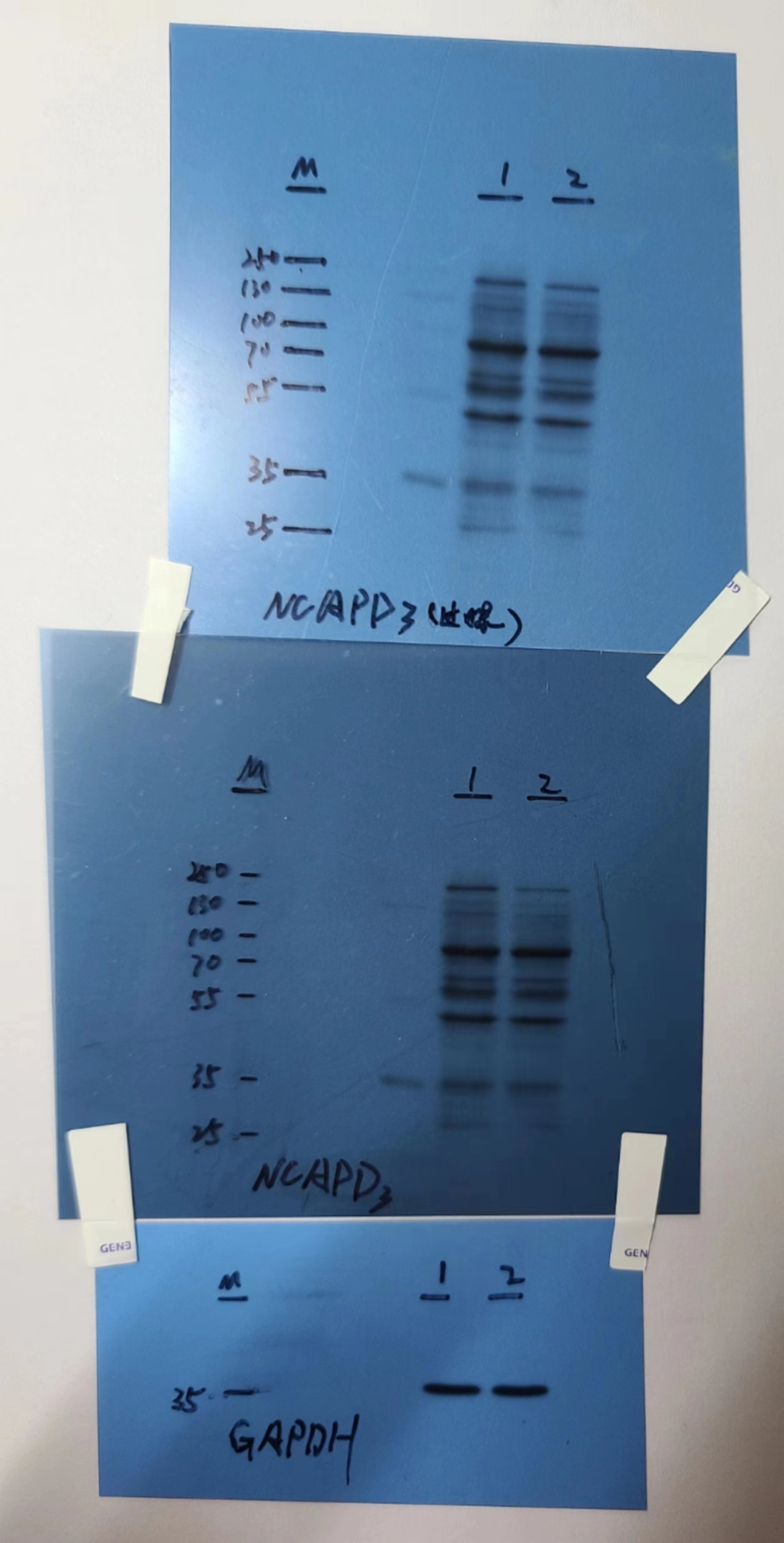

Supplement: Supplementary file 4 [file DataSheet9.ZIP › Figure 3-A4-MGC803.jpg]

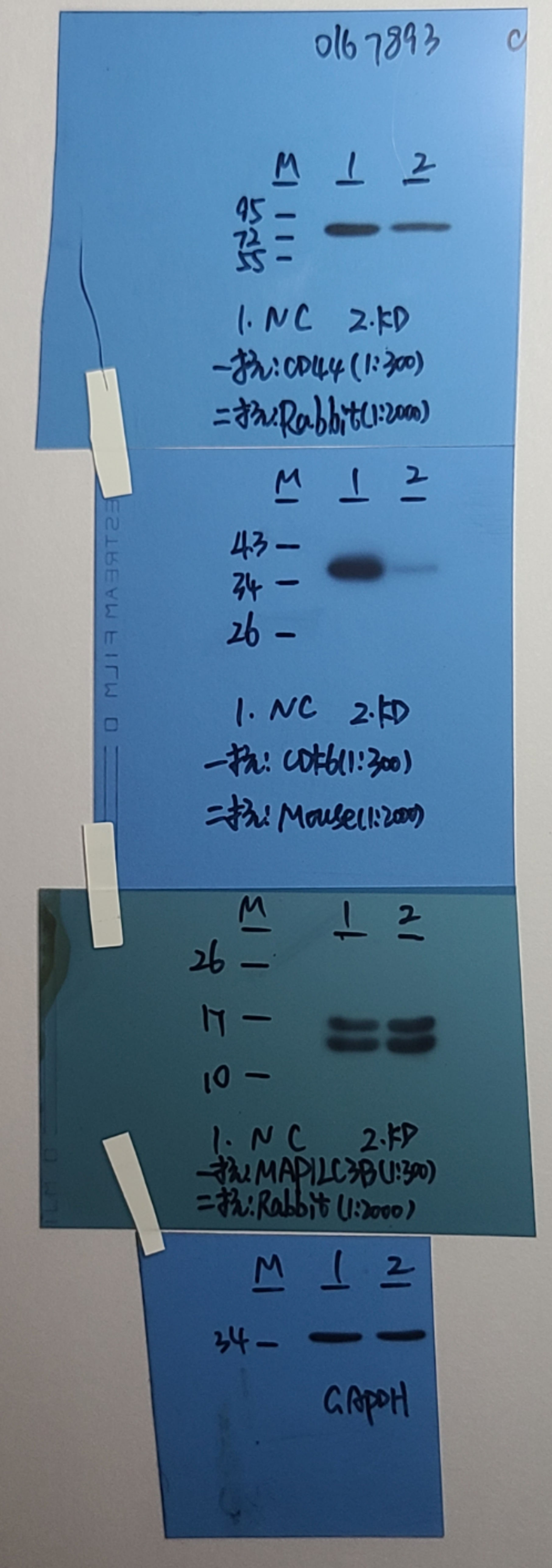

Supplement: Supplementary file 4 [file DataSheet9.ZIP › Figure 8-B-CD44 CDK6.jpg]

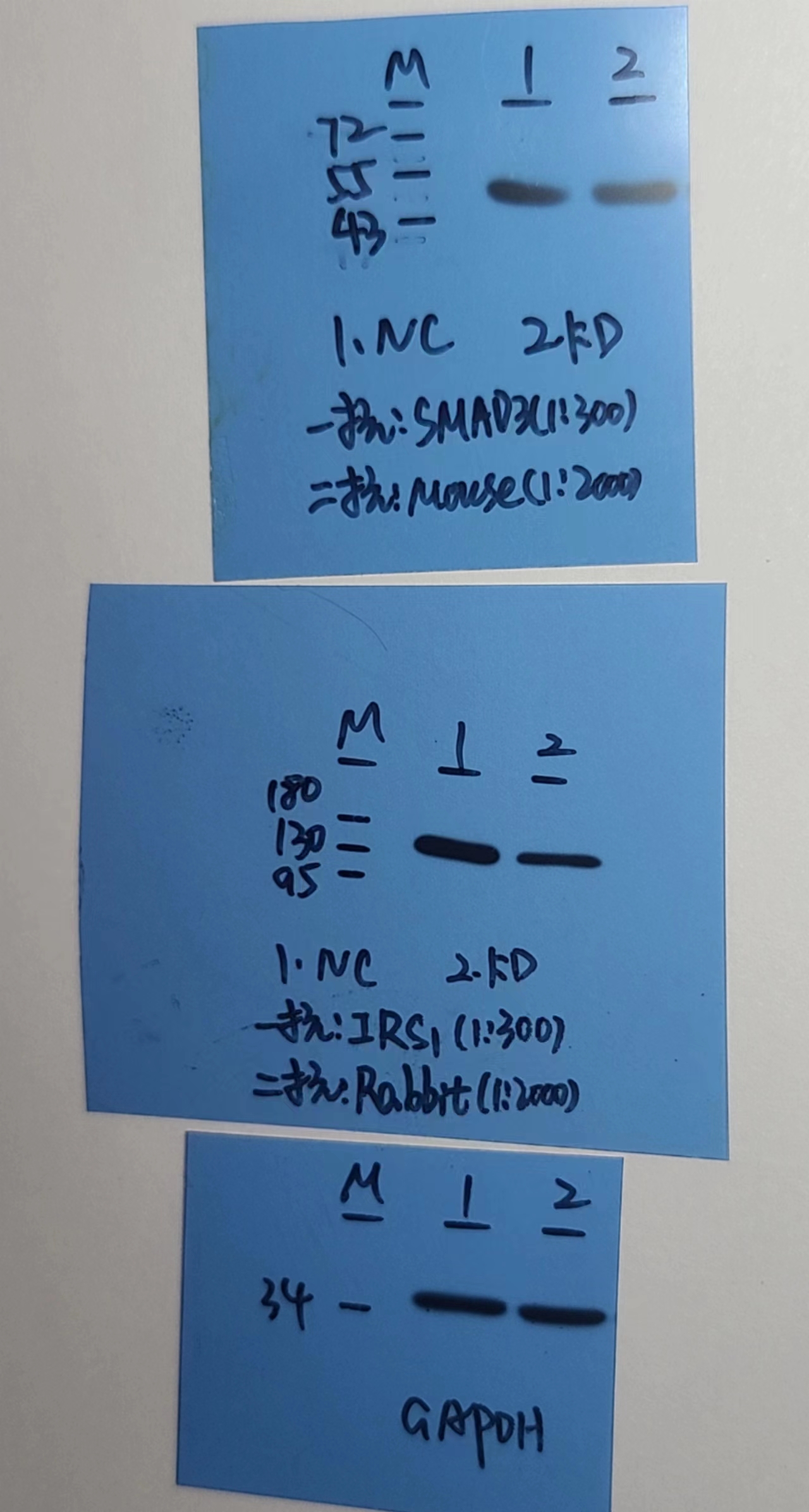

Supplement: Supplementary file 4 [file DataSheet9.ZIP › Figure 8-B-IRS1.jpg]

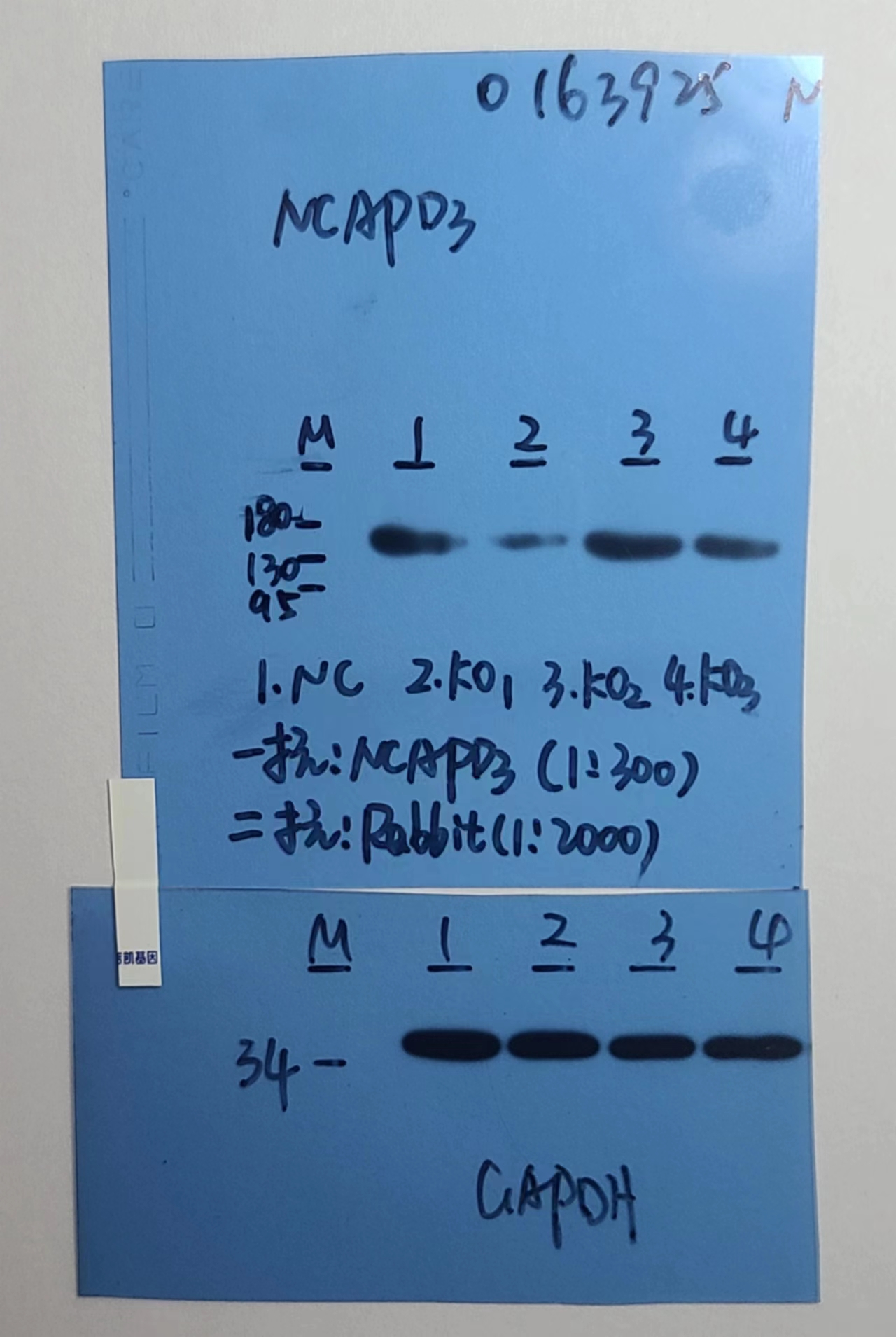

Supplement: Supplementary file 4 [file DataSheet9.ZIP › WB of KO.jpg]

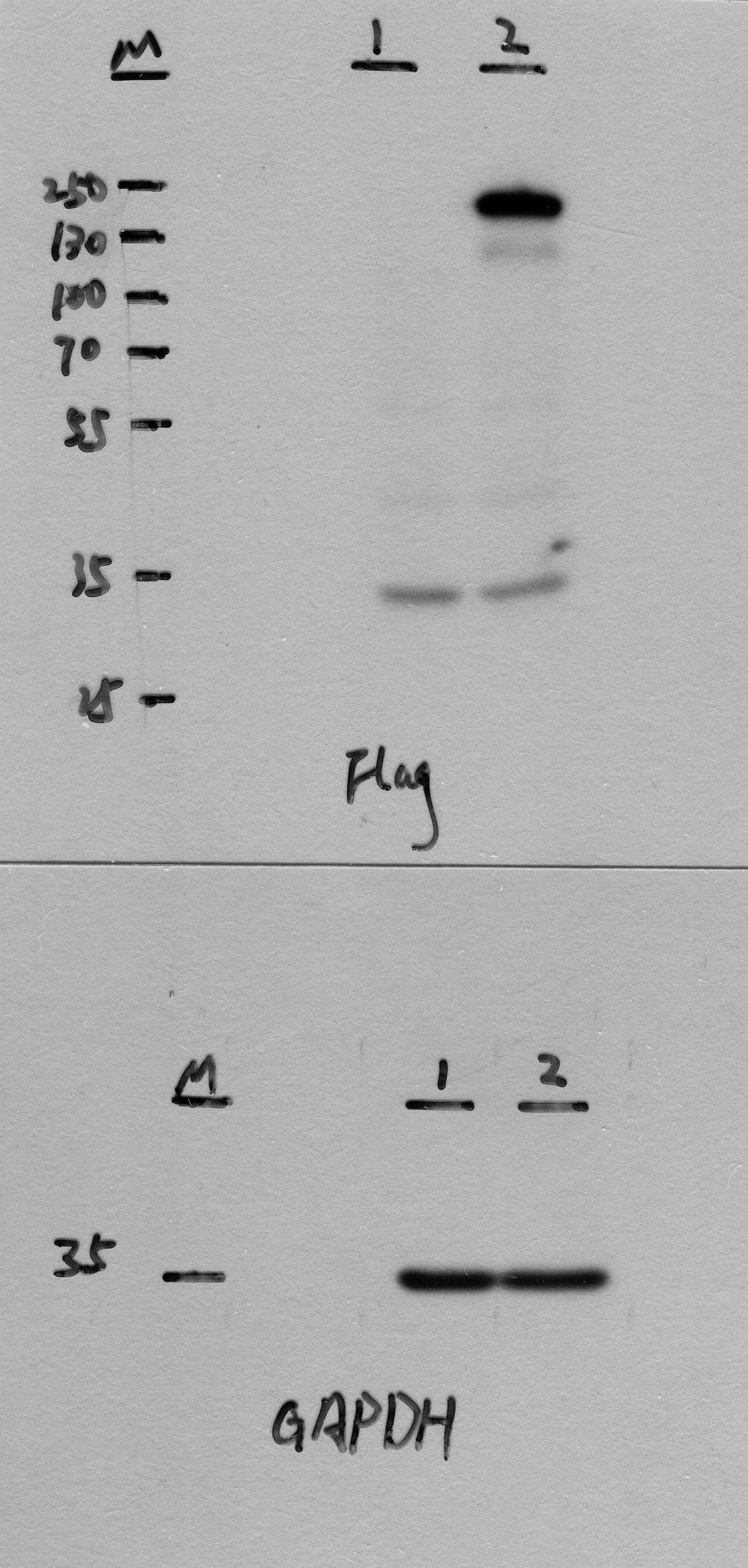

Supplement: Supplementary file 7 [file DataSheet1.ZIP › Figure 2-B2-AGS.tif]

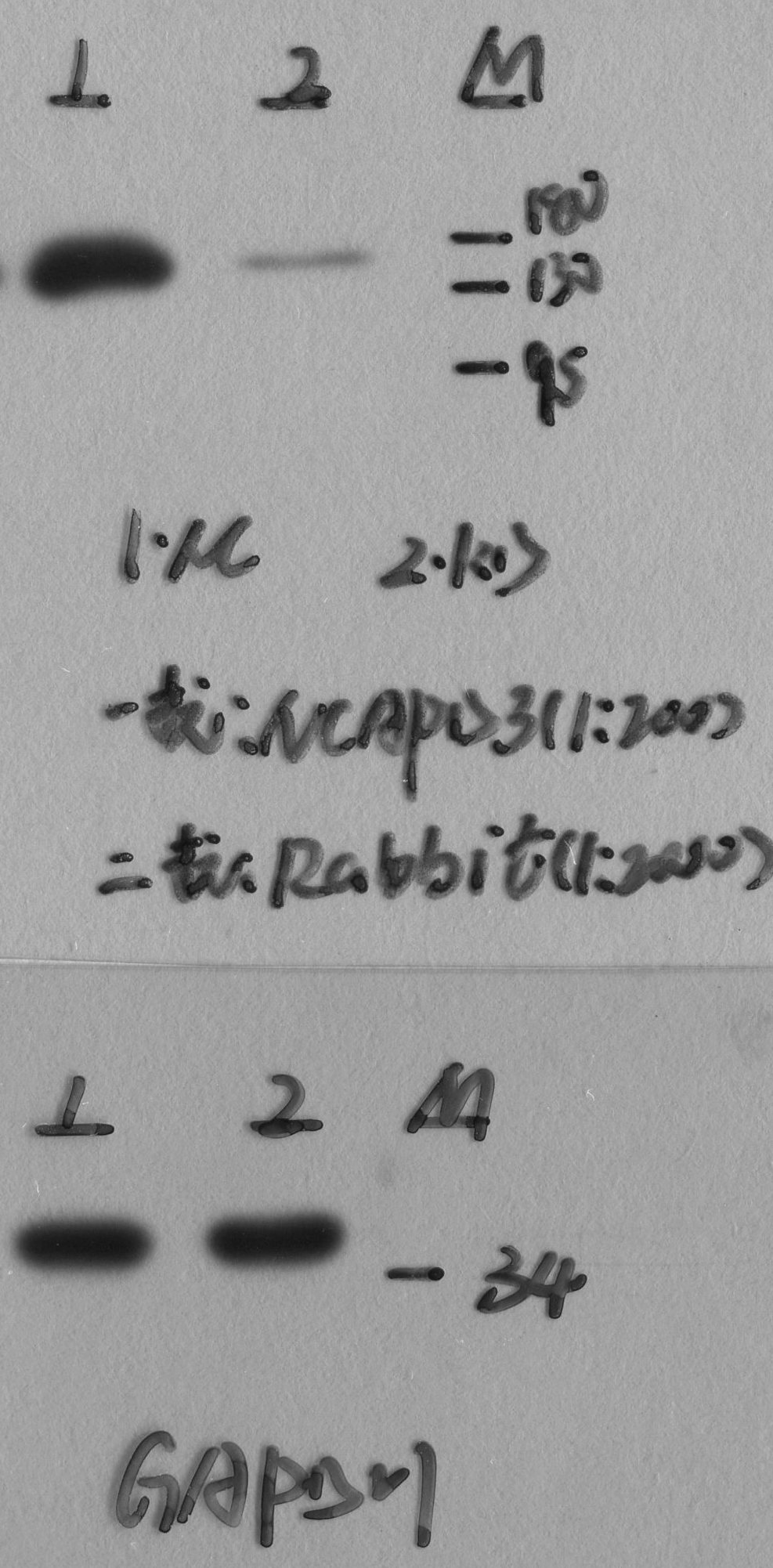

Supplement: Supplementary file 7 [file DataSheet1.ZIP › Figure 3-A2-AGS.tif]

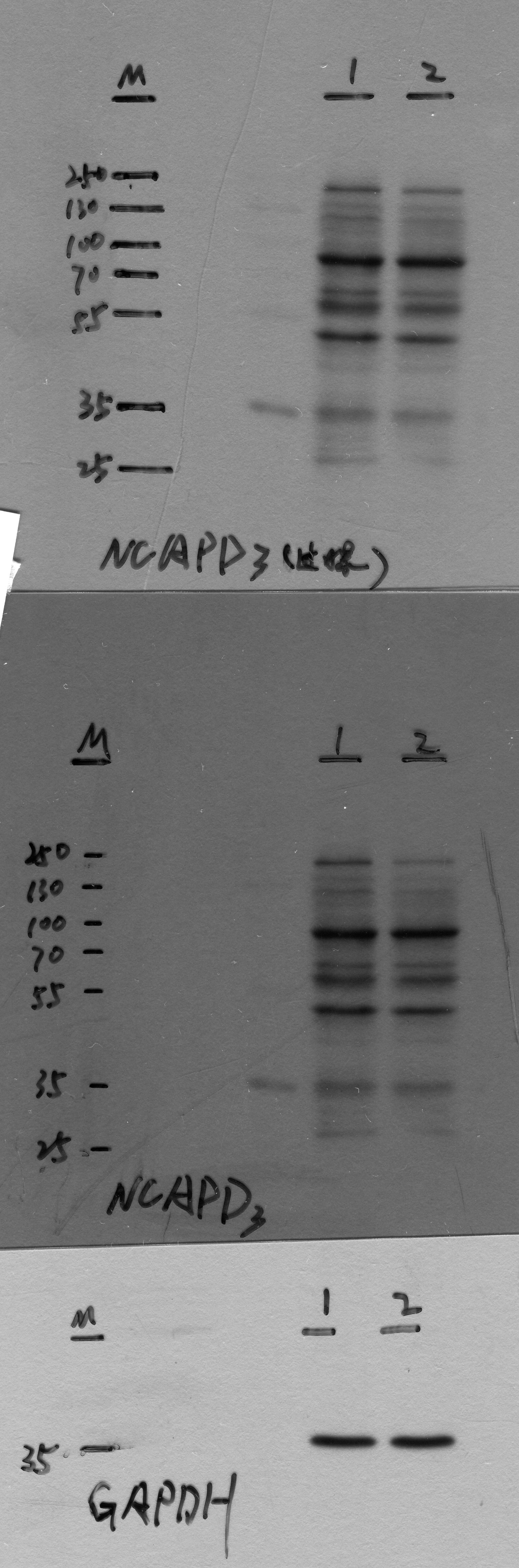

Supplement: Supplementary file 7 [file DataSheet1.ZIP › Figure 3-A4-MGC803.tif]

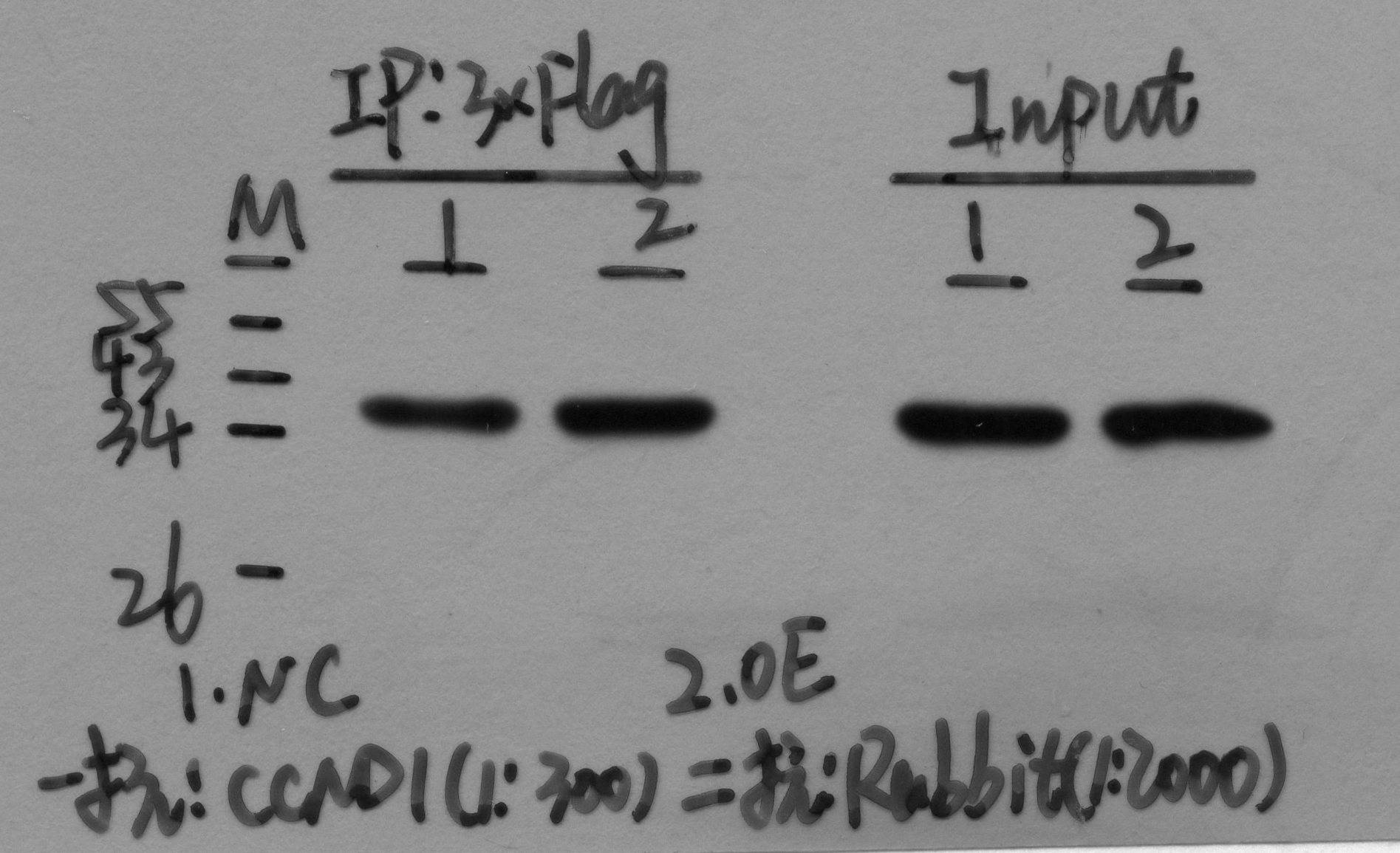

Supplement: Supplementary file 7 [file DataSheet1.ZIP › Figure 8-A-CCND1.tif]

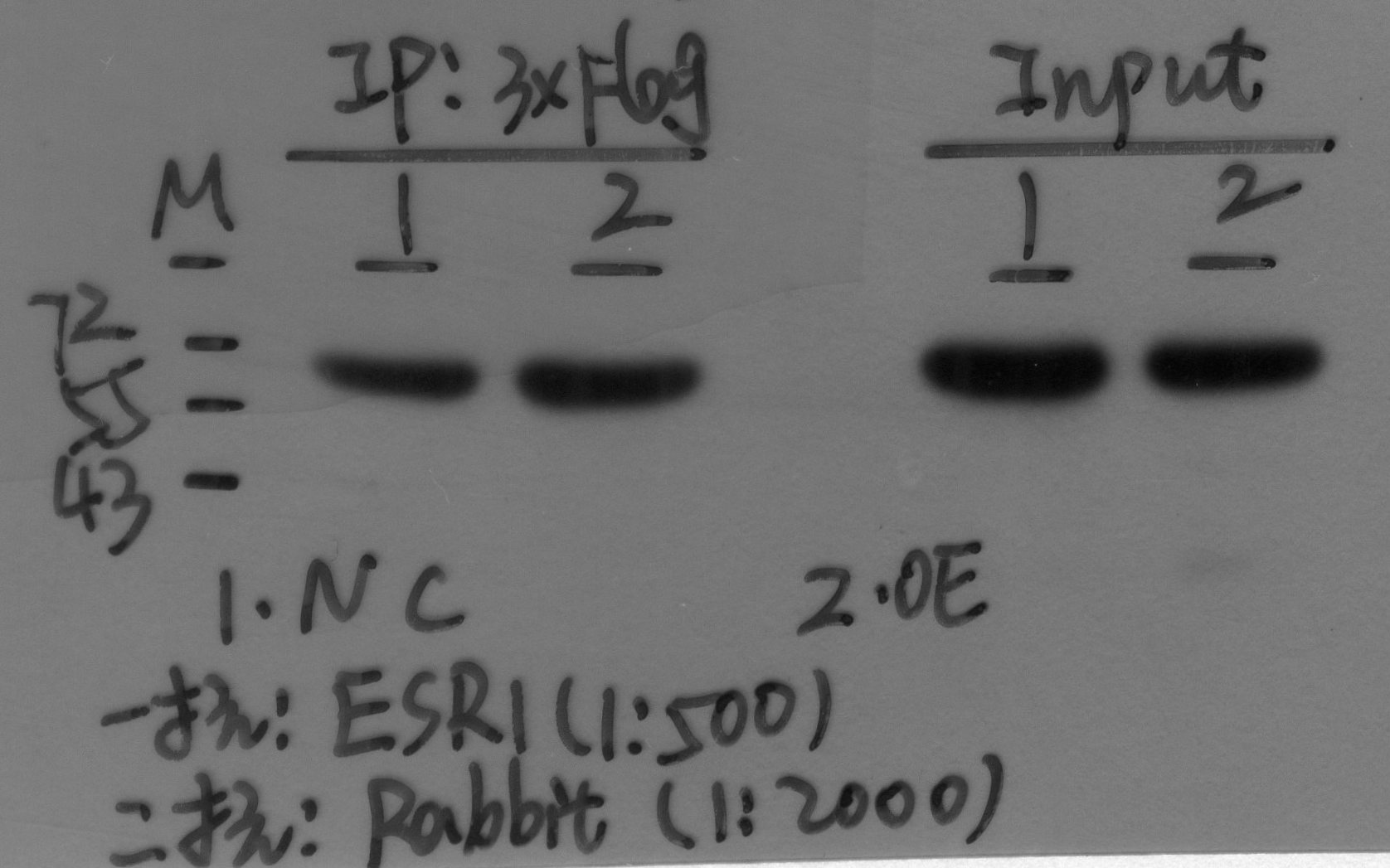

Supplement: Supplementary file 7 [file DataSheet1.ZIP › Figure 8-A-ESR1.tif]

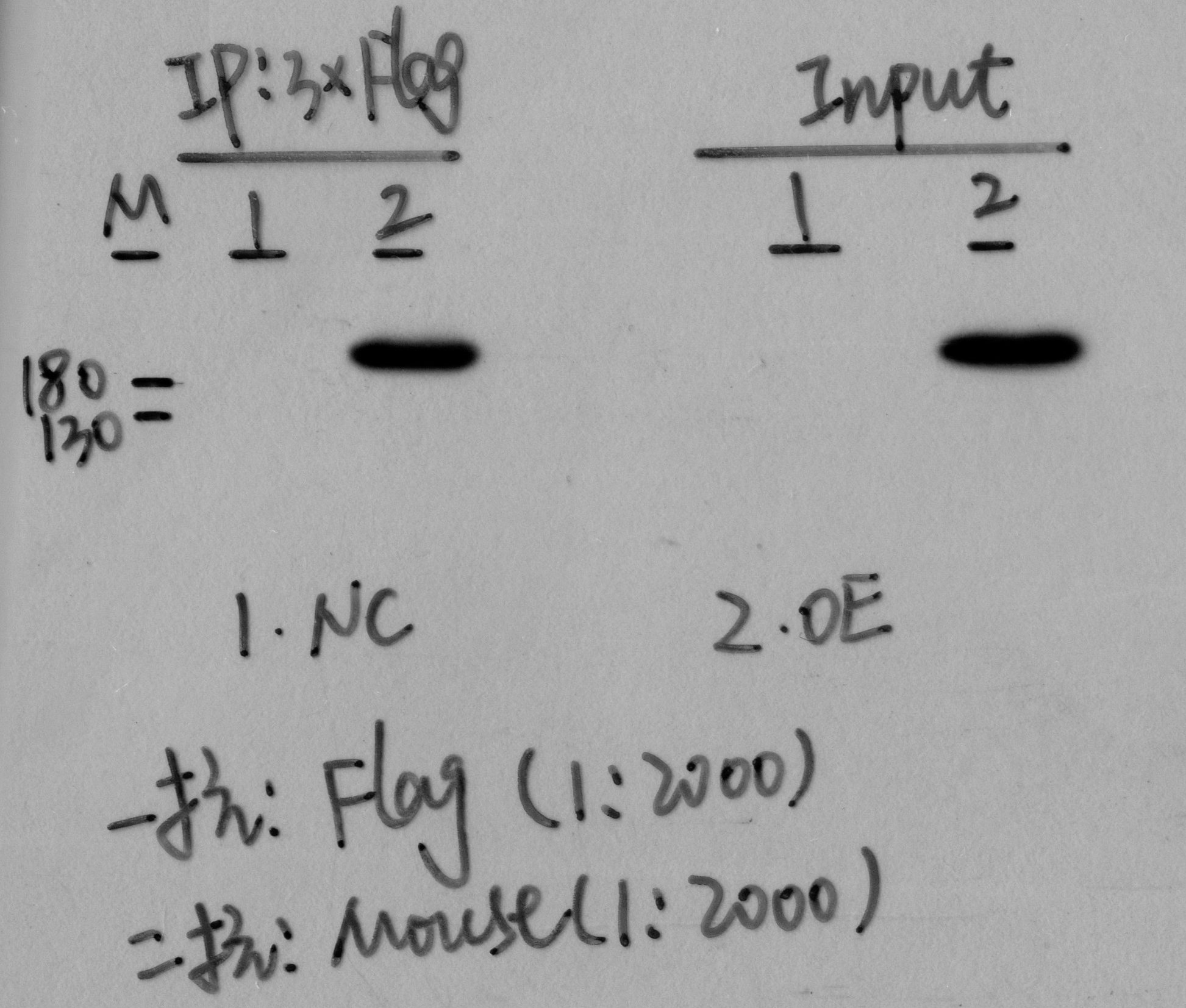

Supplement: Supplementary file 7 [file DataSheet1.ZIP › Figure 8-A-Flag(3X Flag Input).tif]

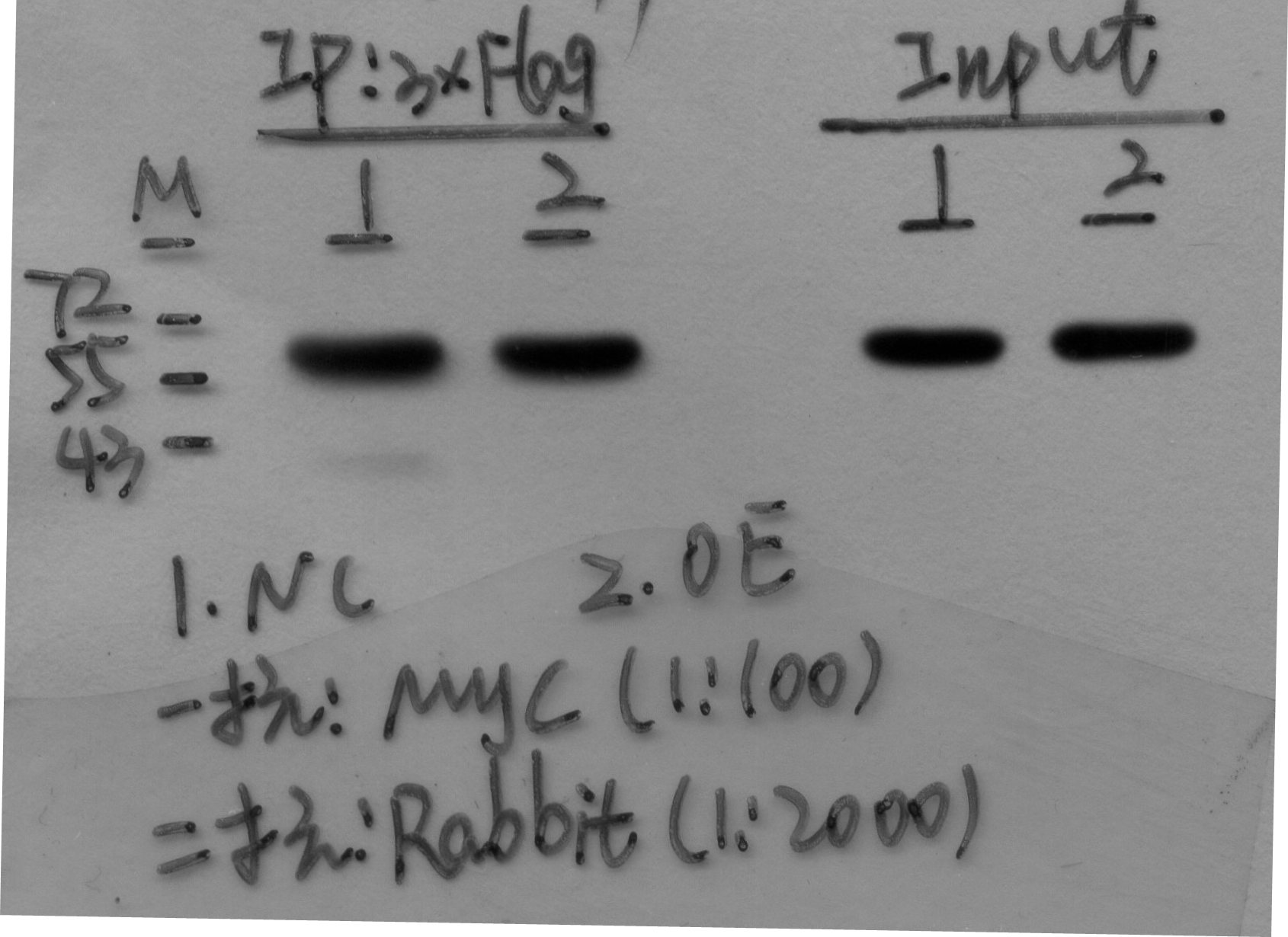

Supplement: Supplementary file 7 [file DataSheet1.ZIP › Figure 8-A-MYC.tif]

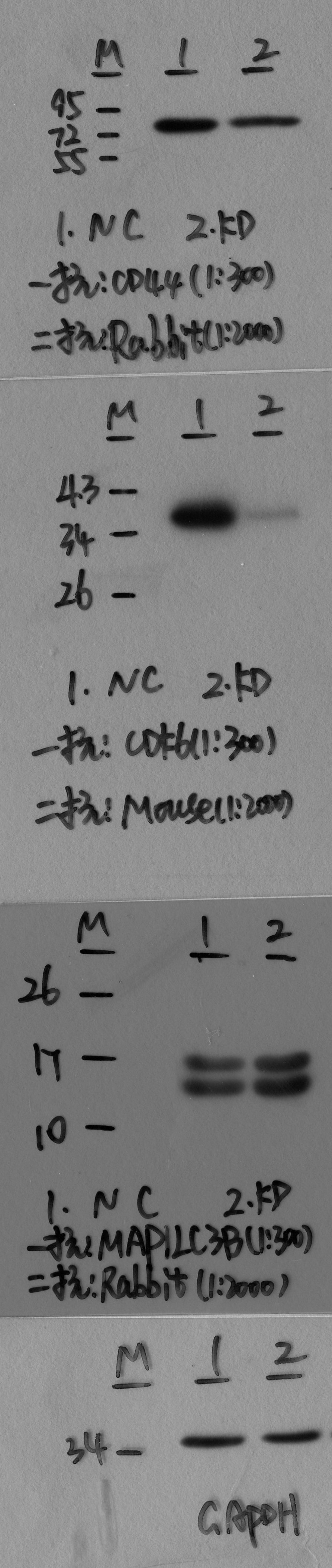

Supplement: Supplementary file 7 [file DataSheet1.ZIP › Figure 8-B-CD44 CDK6.tif]

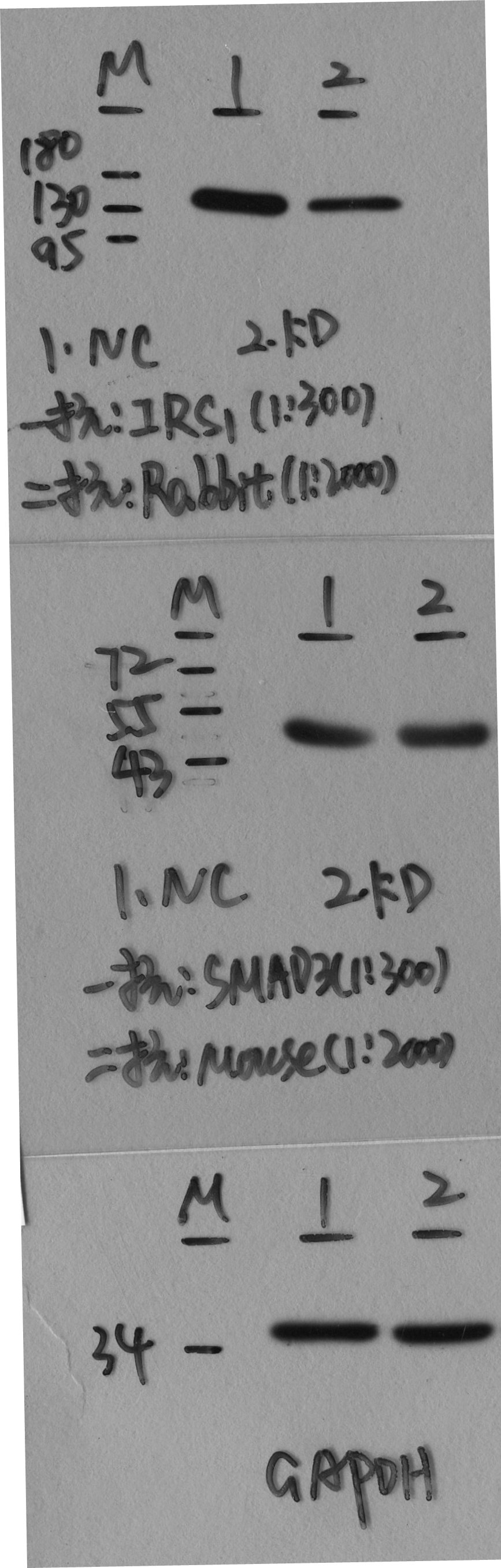

Supplement: Supplementary file 7 [file DataSheet1.ZIP › Figure 8-B-IRS1.tif]

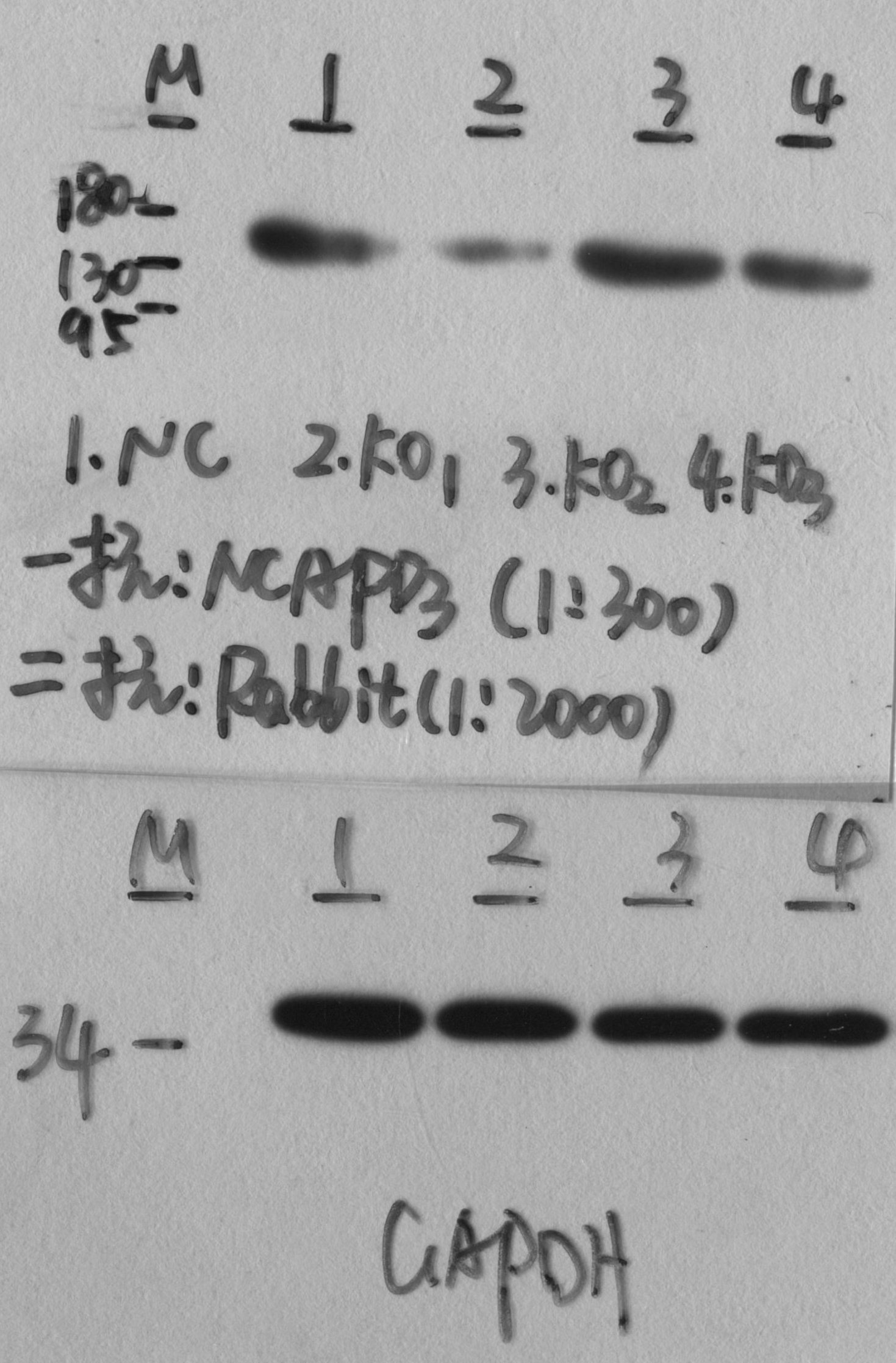

Supplement: Supplementary file 8 [file DataSheet10.ZIP › Raw image of Supplementary Figure 1.jpg]

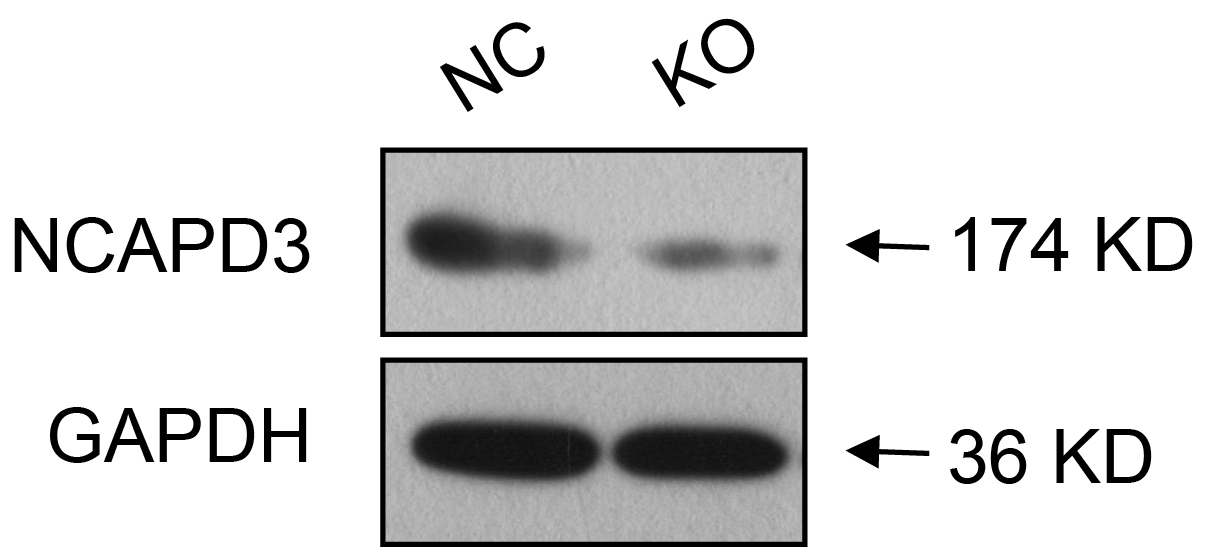

Supplement: Supplementary file 8 [file DataSheet10.ZIP › Supplementary Figure 1.jpg]

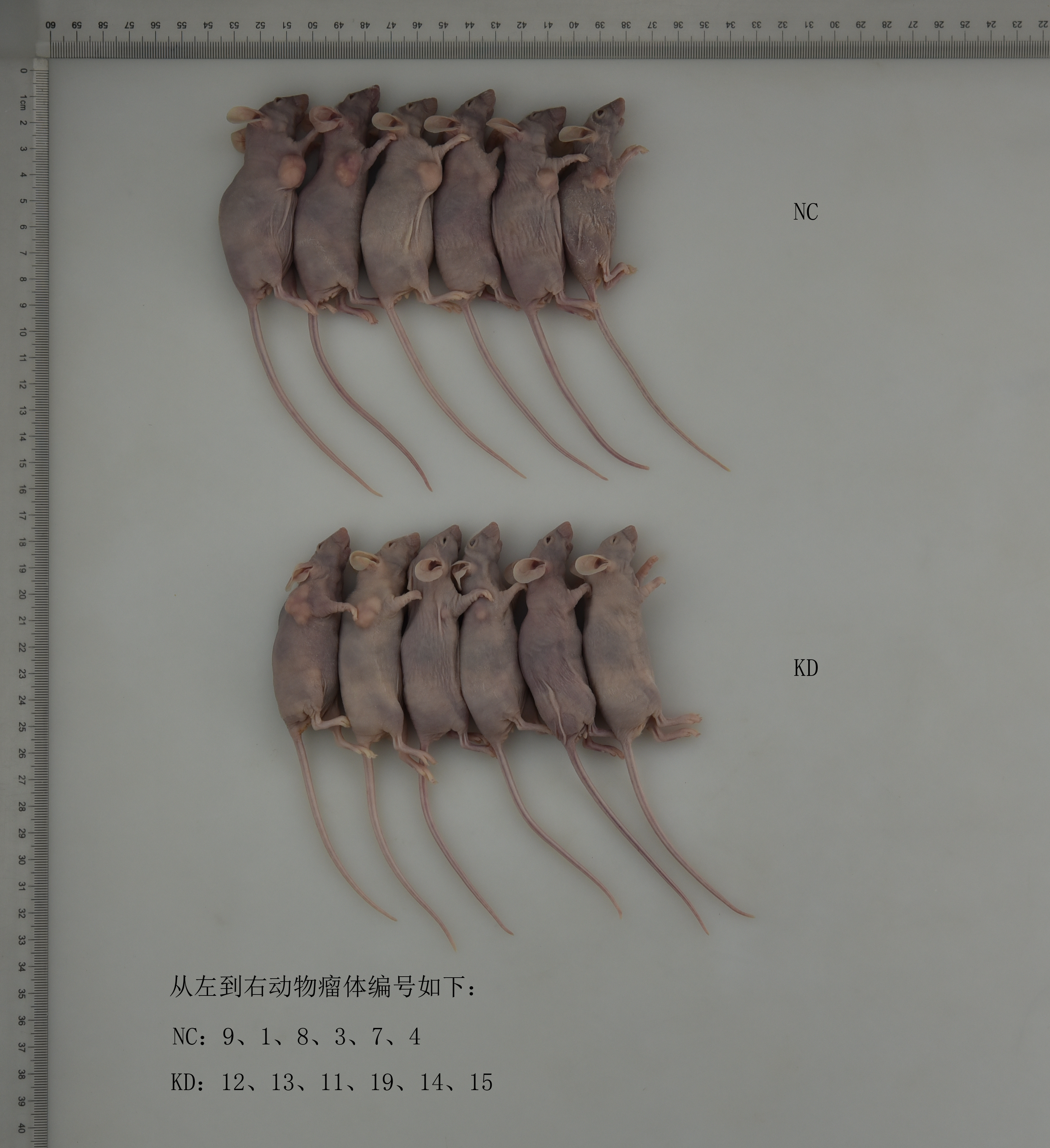

Supplement: Supplementary file 9 [file DataSheet6.ZIP › DSC_7712.JPG]

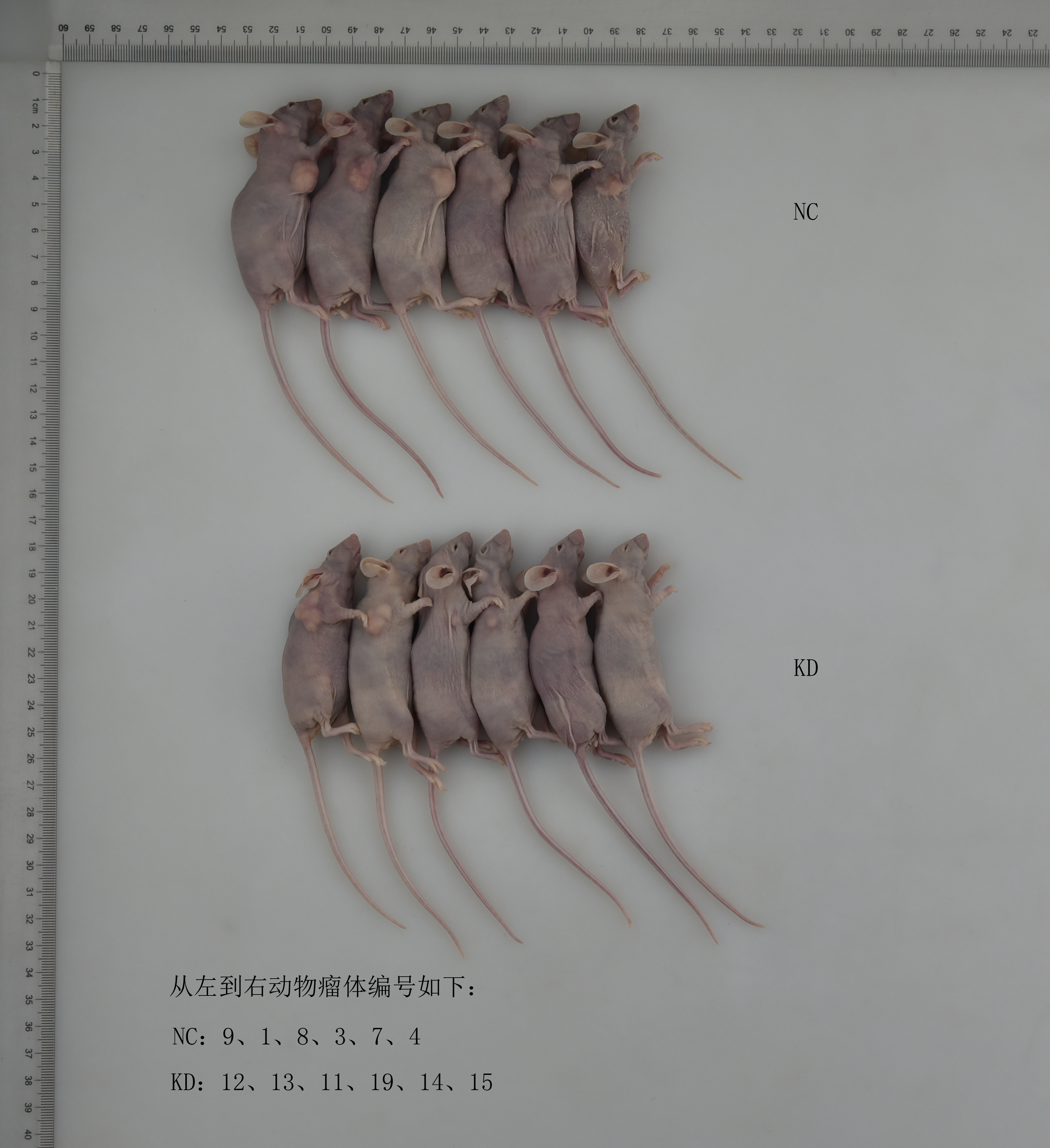

Supplement: Supplementary file 9 [file DataSheet6.ZIP › DSC_7713.JPG]

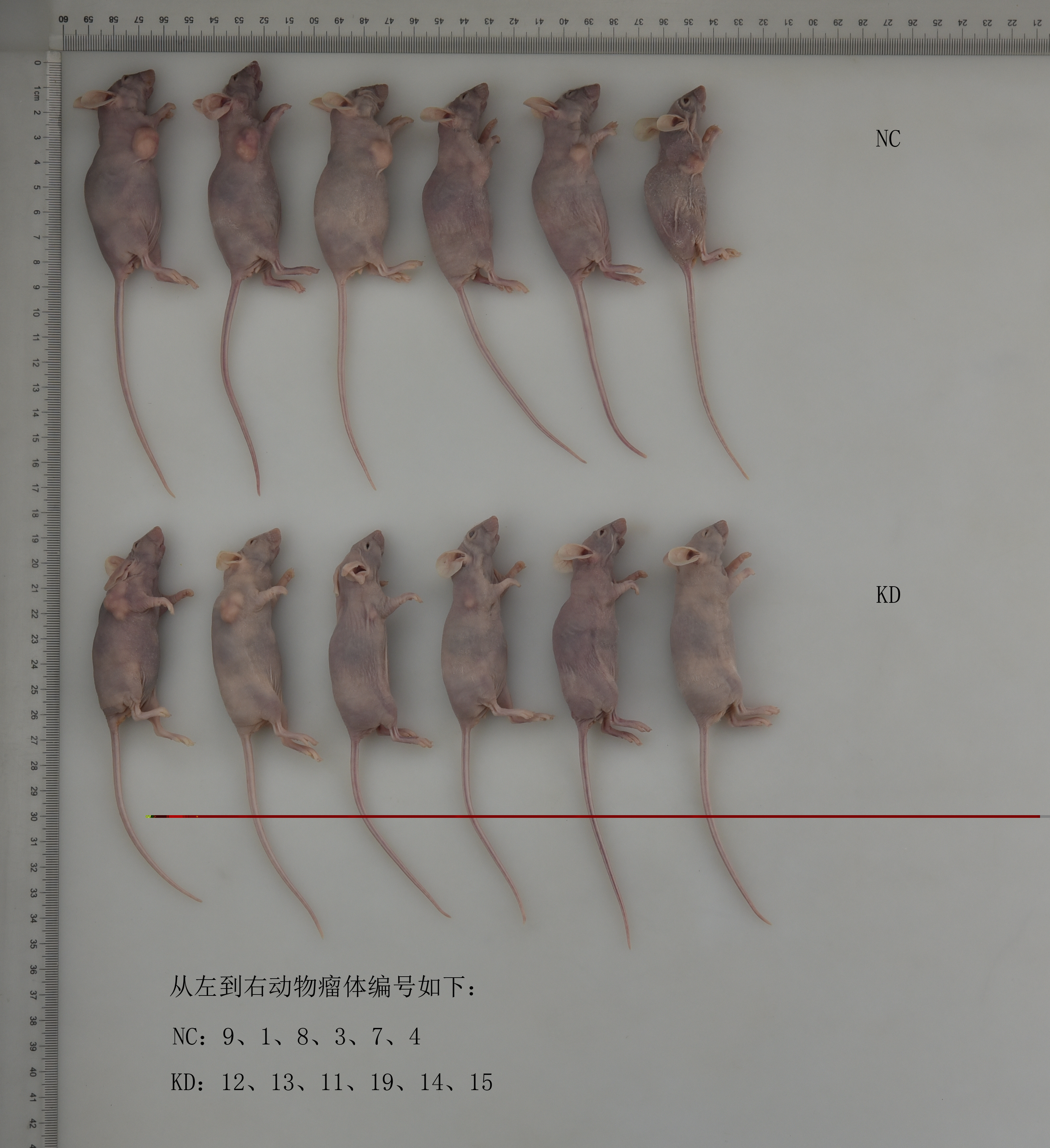

Supplement: Supplementary file 9 [file DataSheet6.ZIP › DSC_7716.JPG]

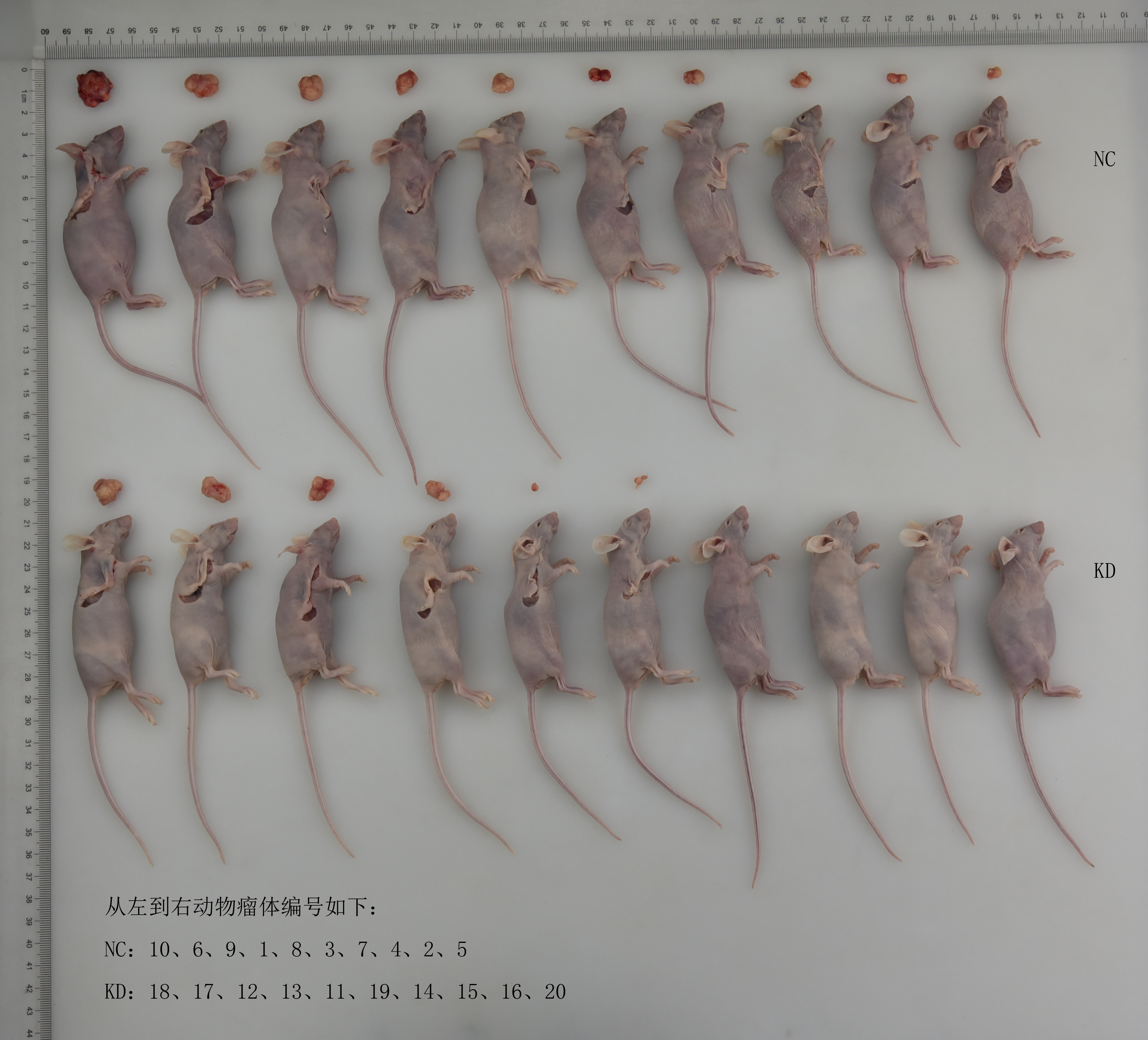

Supplement: Supplementary file 10 [file DataSheet12.ZIP › mice and tunor_10 mice per group.JPG]

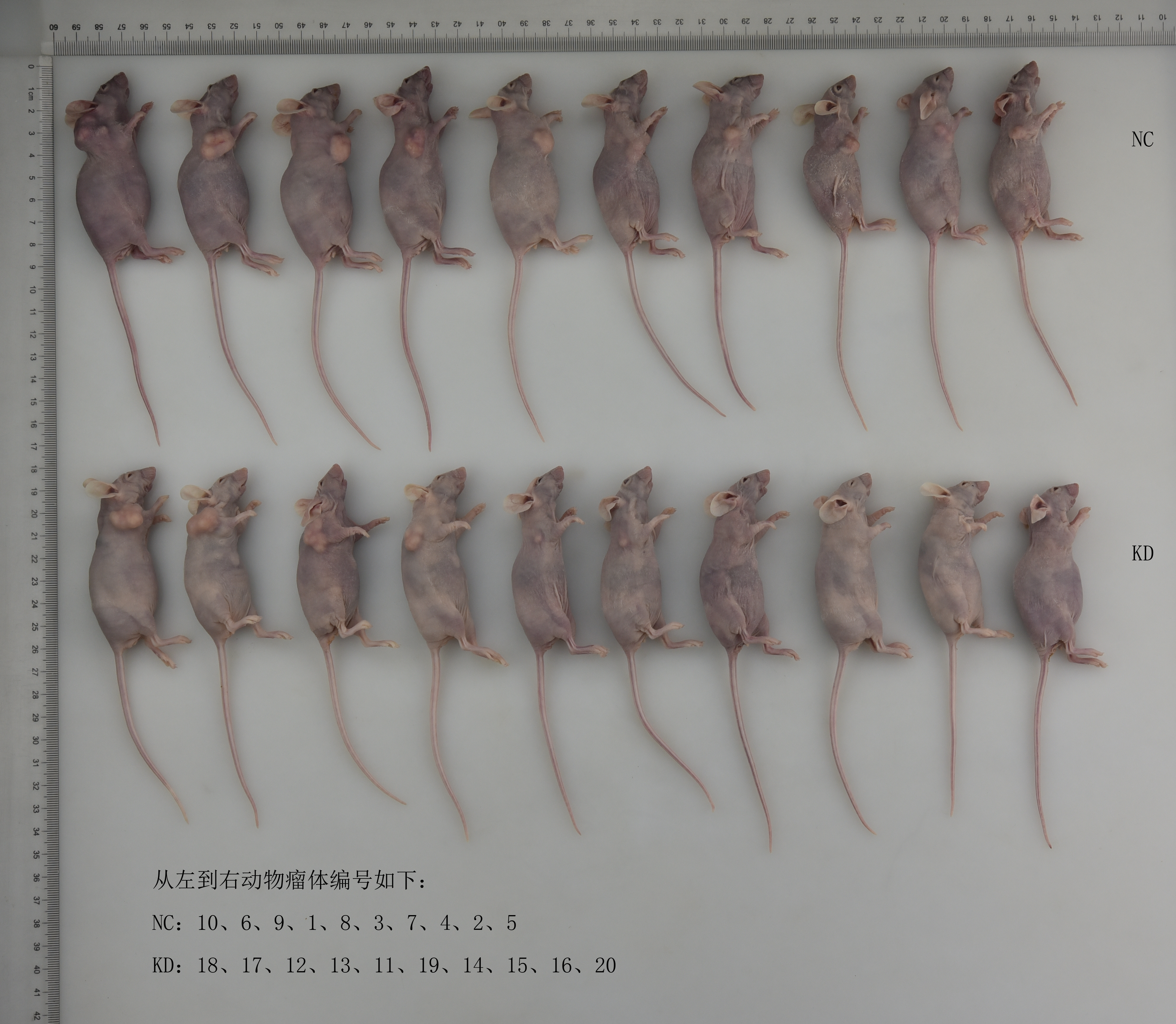

Supplement: Supplementary file 10 [file DataSheet12.ZIP › mice_10 mice per group.JPG]

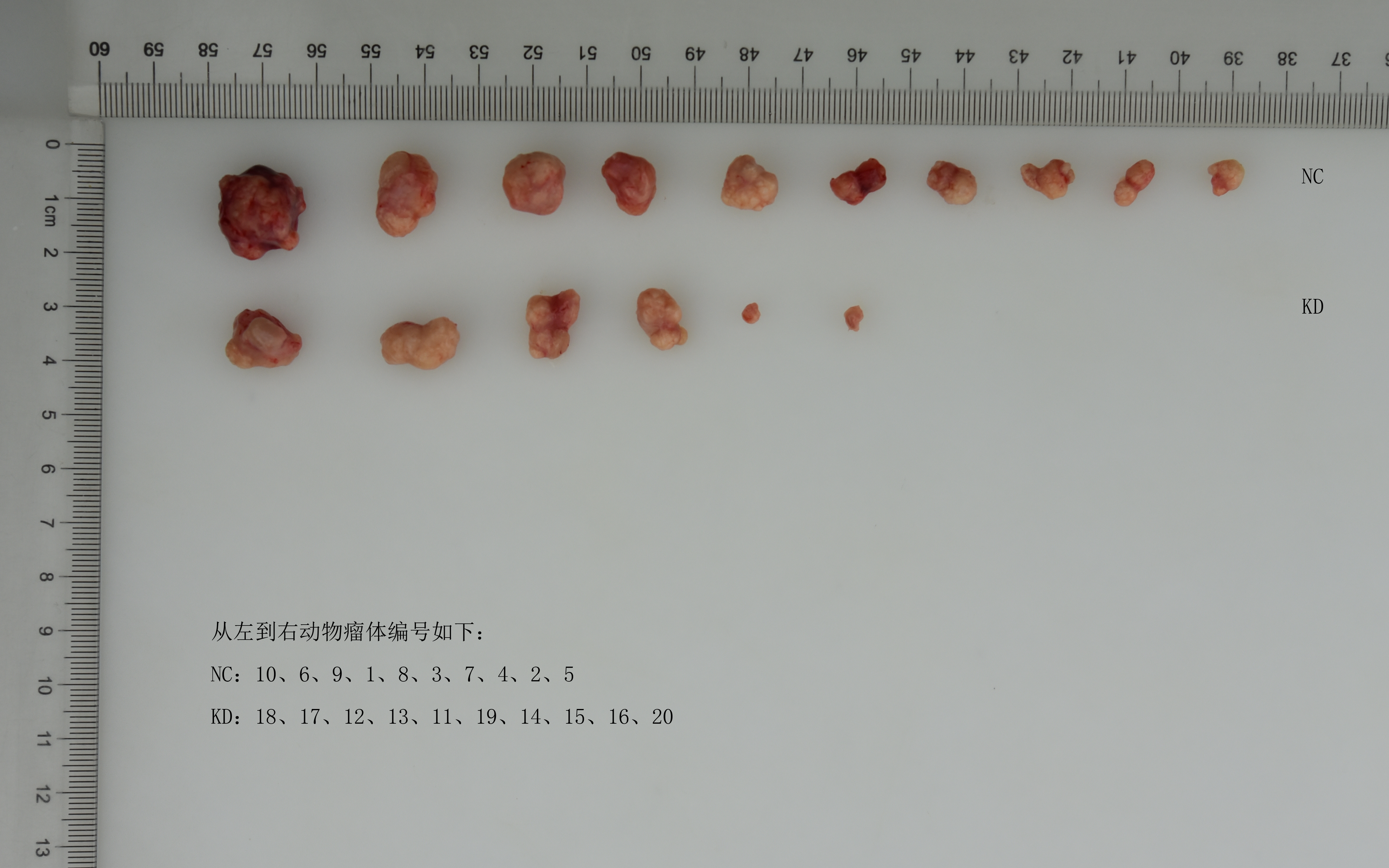

Supplement: Supplementary file 10 [file DataSheet12.ZIP › tumor_10 mice per group.JPG]

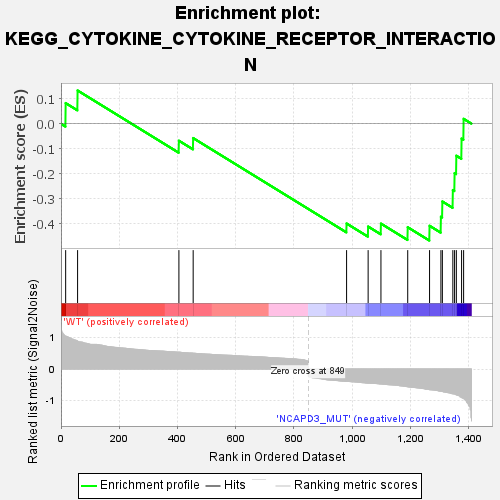

Supplement: Supplementary file 11 [file DataSheet2.ZIP › GSEA/Canonical pathways/my_analysis.Gsea.1599462267220/enplot_KEGG_CYTOKINE_CYTOKINE_RECEPTOR_INTERACTION_795.png]

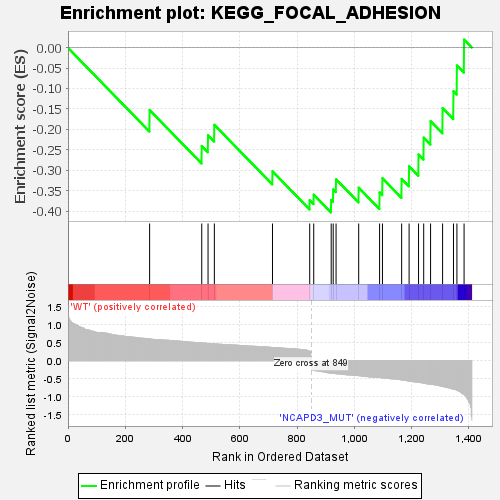

Supplement: Supplementary file 11 [file DataSheet2.ZIP › GSEA/Canonical pathways/my_analysis.Gsea.1599462267220/enplot_KEGG_FOCAL_ADHESION_801.png]

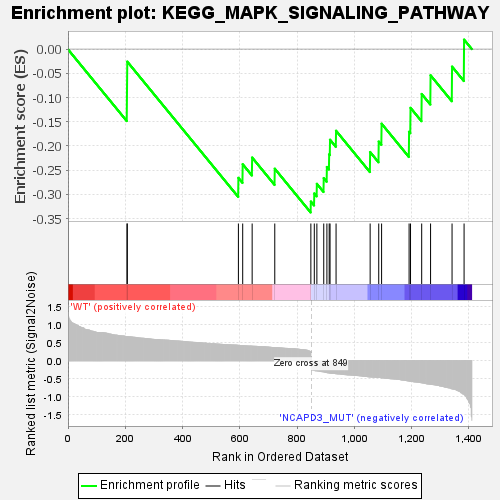

Supplement: Supplementary file 11 [file DataSheet2.ZIP › GSEA/Canonical pathways/my_analysis.Gsea.1599462267220/enplot_KEGG_MAPK_SIGNALING_PATHWAY_819.png]

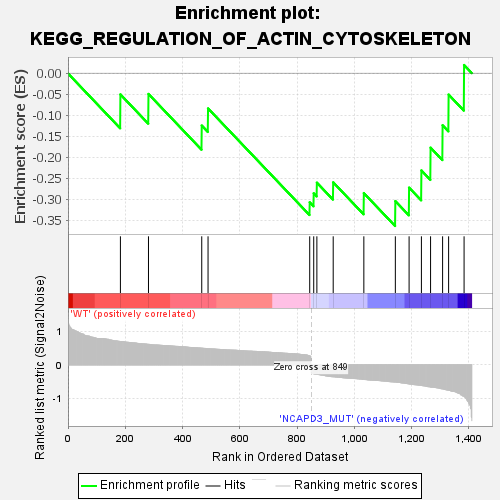

Supplement: Supplementary file 11 [file DataSheet2.ZIP › GSEA/Canonical pathways/my_analysis.Gsea.1599462267220/enplot_KEGG_REGULATION_OF_ACTIN_CYTOSKELETON_825.png]

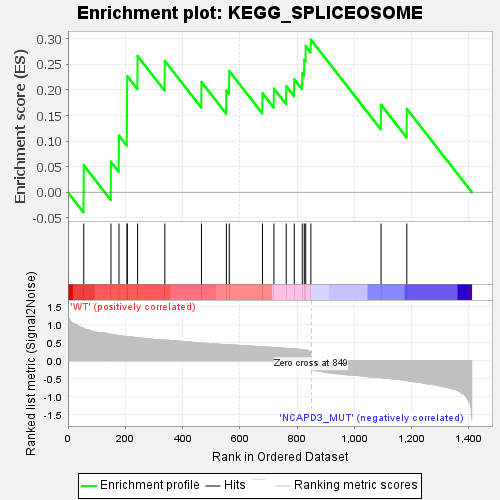

Supplement: Supplementary file 11 [file DataSheet2.ZIP › GSEA/Canonical pathways/my_analysis.Gsea.1599462267220/enplot_KEGG_SPLICEOSOME_741.png]

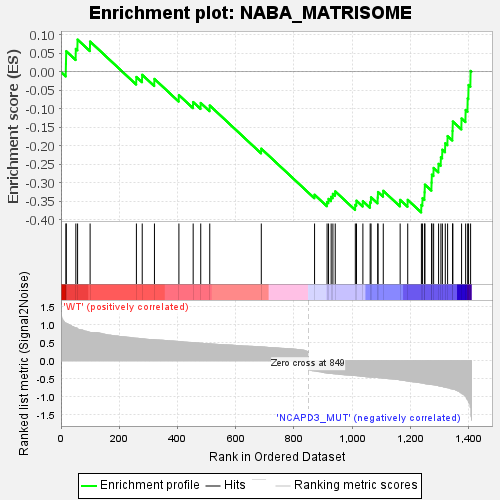

Supplement: Supplementary file 11 [file DataSheet2.ZIP › GSEA/Canonical pathways/my_analysis.Gsea.1599462267220/enplot_NABA_MATRISOME_777.png]

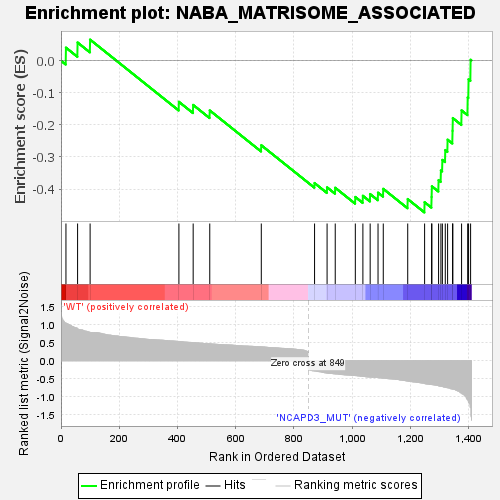

Supplement: Supplementary file 11 [file DataSheet2.ZIP › GSEA/Canonical pathways/my_analysis.Gsea.1599462267220/enplot_NABA_MATRISOME_ASSOCIATED_774.png]

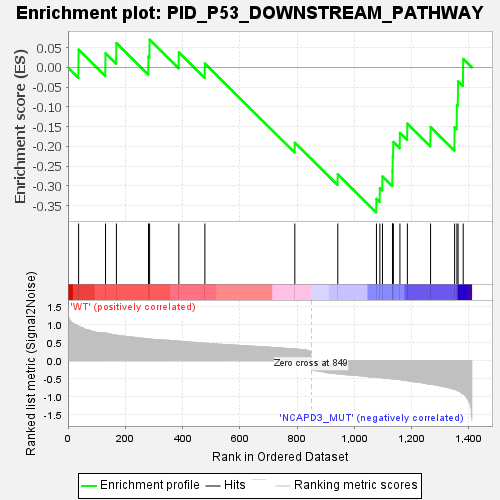

Supplement: Supplementary file 11 [file DataSheet2.ZIP › GSEA/Canonical pathways/my_analysis.Gsea.1599462267220/enplot_PID_P53_DOWNSTREAM_PATHWAY_807.png]

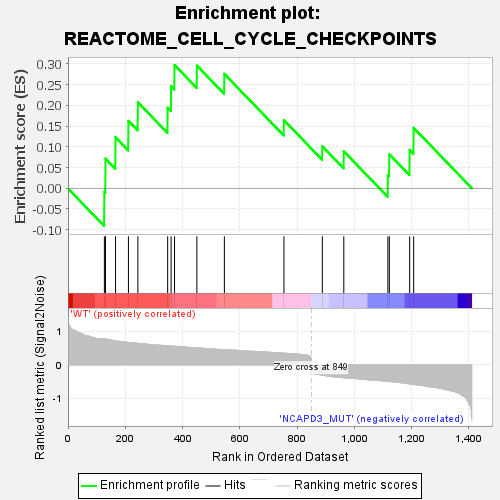

Supplement: Supplementary file 11 [file DataSheet2.ZIP › GSEA/Canonical pathways/my_analysis.Gsea.1599462267220/enplot_REACTOME_CELL_CYCLE_CHECKPOINTS_747.png]

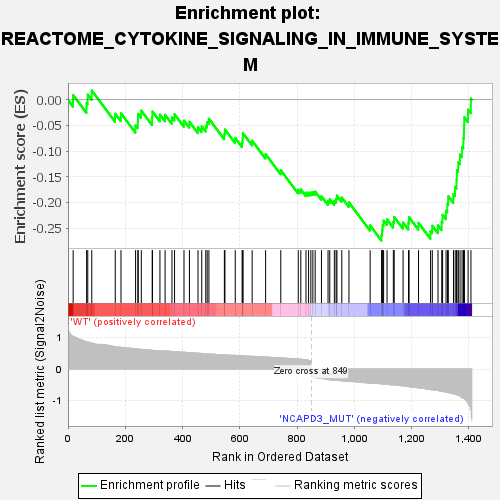

Supplement: Supplementary file 11 [file DataSheet2.ZIP › GSEA/Canonical pathways/my_analysis.Gsea.1599462267220/enplot_REACTOME_CYTOKINE_SIGNALING_IN_IMMUNE_SYSTEM_792.png]

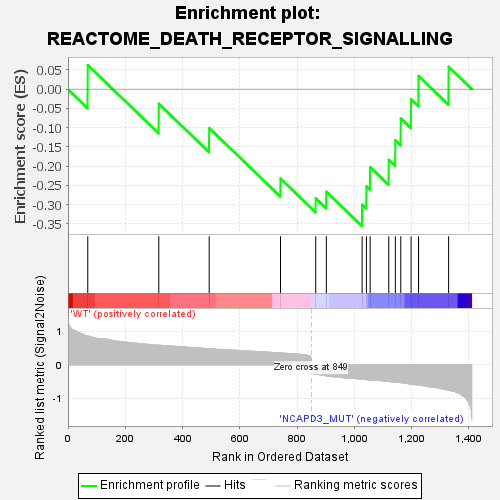

Supplement: Supplementary file 11 [file DataSheet2.ZIP › GSEA/Canonical pathways/my_analysis.Gsea.1599462267220/enplot_REACTOME_DEATH_RECEPTOR_SIGNALLING_831.png]

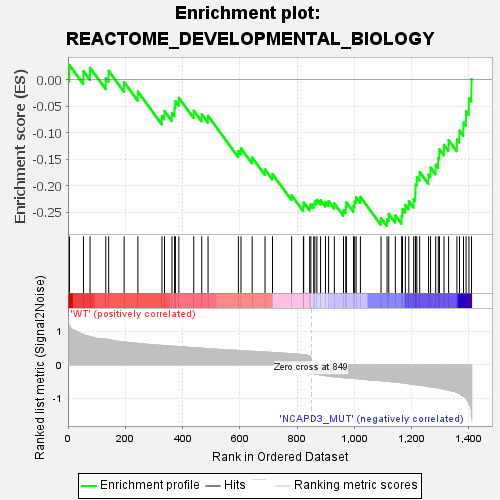

Supplement: Supplementary file 11 [file DataSheet2.ZIP › GSEA/Canonical pathways/my_analysis.Gsea.1599462267220/enplot_REACTOME_DEVELOPMENTAL_BIOLOGY_804.png]

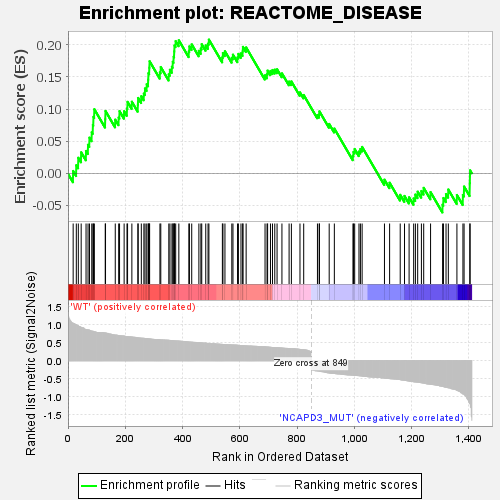

Supplement: Supplementary file 11 [file DataSheet2.ZIP › GSEA/Canonical pathways/my_analysis.Gsea.1599462267220/enplot_REACTOME_DISEASE_738.png]

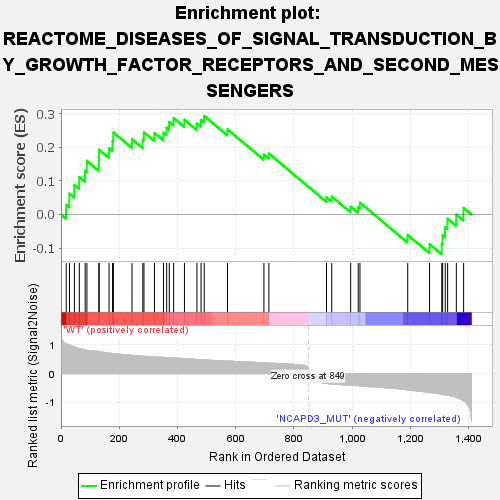

Supplement: Supplementary file 11 [file DataSheet2.ZIP › GSEA/Canonical pathways/my_analysis.Gsea.1599462267220/enplot_REACTOME_DISEASES_OF_SIGNAL_TRANSDUCTION_BY_GROWTH_FACTOR_RECEPTORS_AND_SECOND_MESSENGERS_723.png]

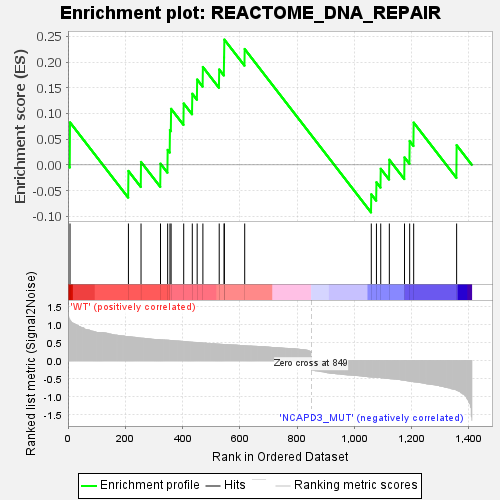

Supplement: Supplementary file 11 [file DataSheet2.ZIP › GSEA/Canonical pathways/my_analysis.Gsea.1599462267220/enplot_REACTOME_DNA_REPAIR_765.png]

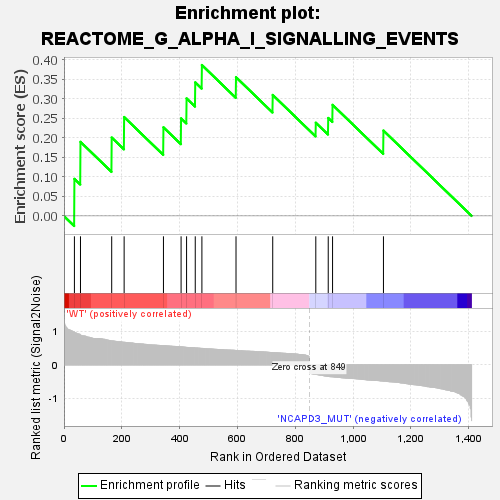

Supplement: Supplementary file 11 [file DataSheet2.ZIP › GSEA/Canonical pathways/my_analysis.Gsea.1599462267220/enplot_REACTOME_G_ALPHA_I_SIGNALLING_EVENTS_732.png]

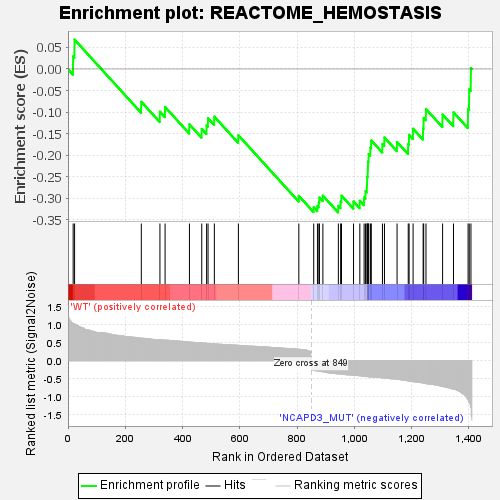

Supplement: Supplementary file 11 [file DataSheet2.ZIP › GSEA/Canonical pathways/my_analysis.Gsea.1599462267220/enplot_REACTOME_HEMOSTASIS_786.png]

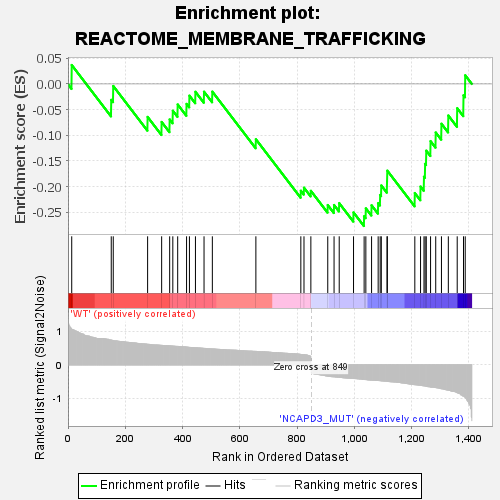

Supplement: Supplementary file 11 [file DataSheet2.ZIP › GSEA/Canonical pathways/my_analysis.Gsea.1599462267220/enplot_REACTOME_MEMBRANE_TRAFFICKING_813.png]

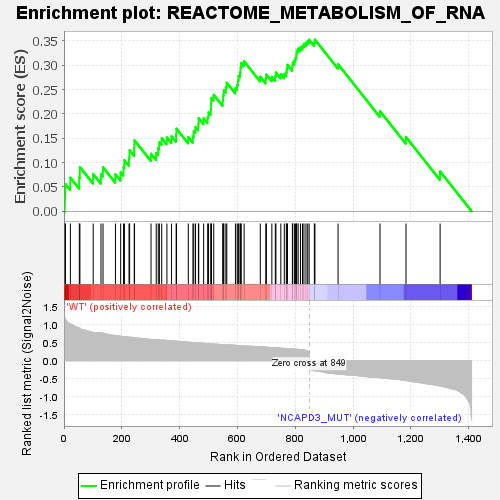

Supplement: Supplementary file 11 [file DataSheet2.ZIP › GSEA/Canonical pathways/my_analysis.Gsea.1599462267220/enplot_REACTOME_METABOLISM_OF_RNA_714.png]

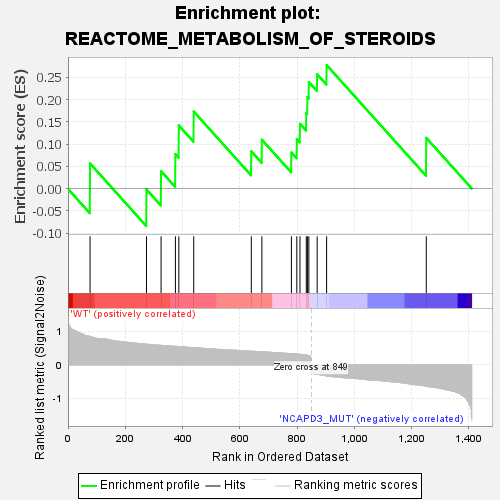

Supplement: Supplementary file 11 [file DataSheet2.ZIP › GSEA/Canonical pathways/my_analysis.Gsea.1599462267220/enplot_REACTOME_METABOLISM_OF_STEROIDS_759.png]

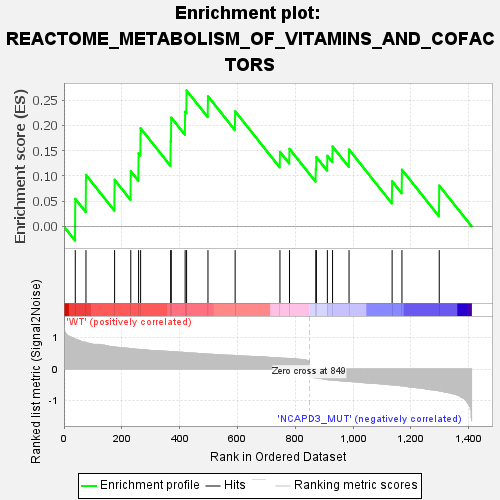

Supplement: Supplementary file 11 [file DataSheet2.ZIP › GSEA/Canonical pathways/my_analysis.Gsea.1599462267220/enplot_REACTOME_METABOLISM_OF_VITAMINS_AND_COFACTORS_750.png]

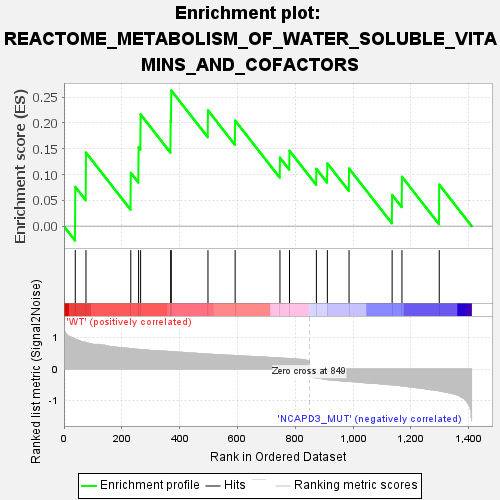

Supplement: Supplementary file 11 [file DataSheet2.ZIP › GSEA/Canonical pathways/my_analysis.Gsea.1599462267220/enplot_REACTOME_METABOLISM_OF_WATER_SOLUBLE_VITAMINS_AND_COFACTORS_768.png]

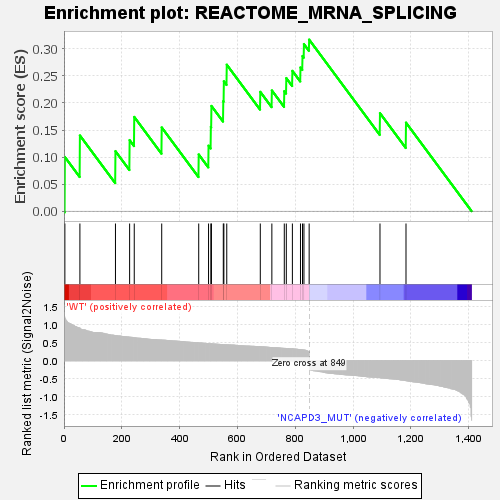

Supplement: Supplementary file 11 [file DataSheet2.ZIP › GSEA/Canonical pathways/my_analysis.Gsea.1599462267220/enplot_REACTOME_MRNA_SPLICING_735.png]

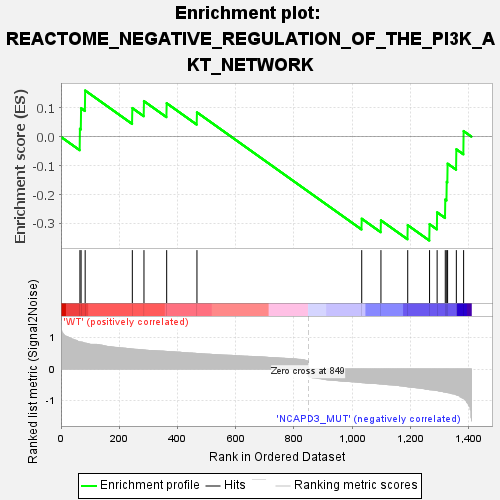

Supplement: Supplementary file 11 [file DataSheet2.ZIP › GSEA/Canonical pathways/my_analysis.Gsea.1599462267220/enplot_REACTOME_NEGATIVE_REGULATION_OF_THE_PI3K_AKT_NETWORK_822.png]

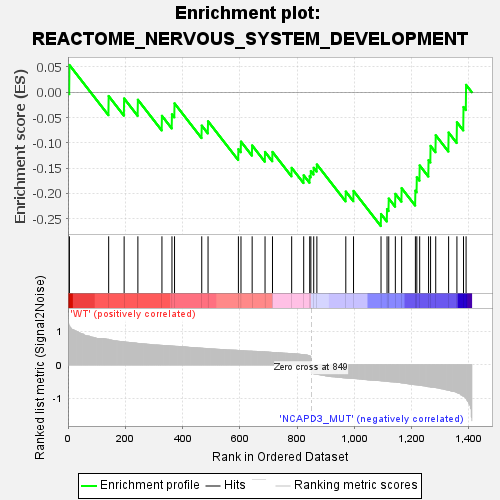

Supplement: Supplementary file 11 [file DataSheet2.ZIP › GSEA/Canonical pathways/my_analysis.Gsea.1599462267220/enplot_REACTOME_NERVOUS_SYSTEM_DEVELOPMENT_828.png]

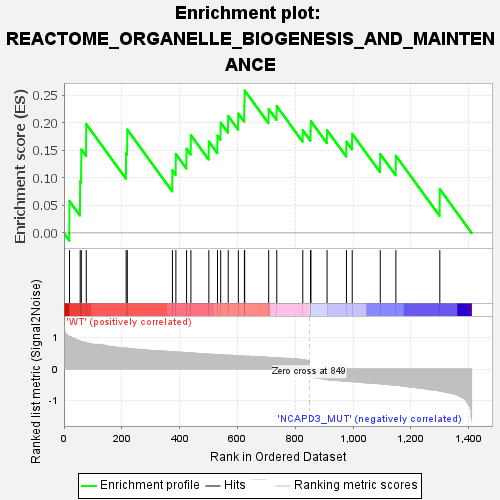

Supplement: Supplementary file 11 [file DataSheet2.ZIP › GSEA/Canonical pathways/my_analysis.Gsea.1599462267220/enplot_REACTOME_ORGANELLE_BIOGENESIS_AND_MAINTENANCE_744.png]

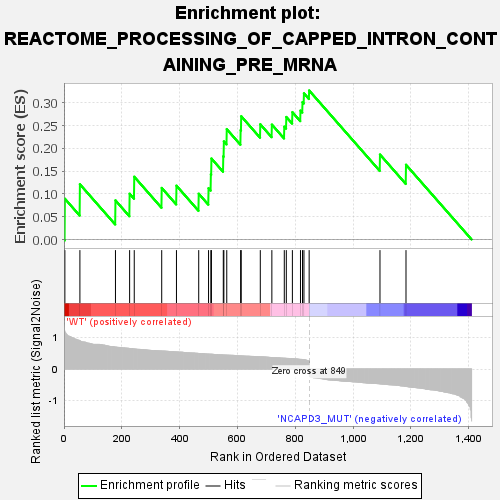

Supplement: Supplementary file 11 [file DataSheet2.ZIP › GSEA/Canonical pathways/my_analysis.Gsea.1599462267220/enplot_REACTOME_PROCESSING_OF_CAPPED_INTRON_CONTAINING_PRE_MRNA_726.png]

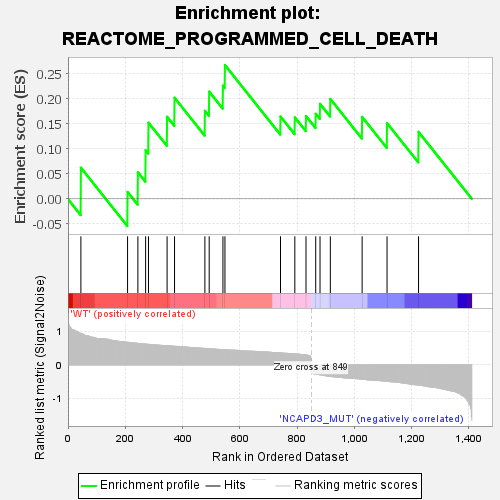

Supplement: Supplementary file 11 [file DataSheet2.ZIP › GSEA/Canonical pathways/my_analysis.Gsea.1599462267220/enplot_REACTOME_PROGRAMMED_CELL_DEATH_756.png]

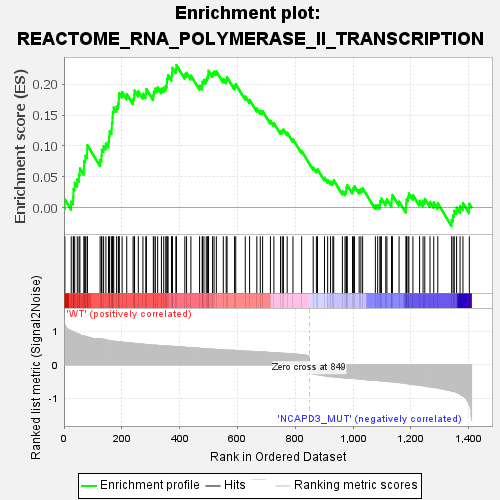

Supplement: Supplementary file 11 [file DataSheet2.ZIP › GSEA/Canonical pathways/my_analysis.Gsea.1599462267220/enplot_REACTOME_RNA_POLYMERASE_II_TRANSCRIPTION_720.png]

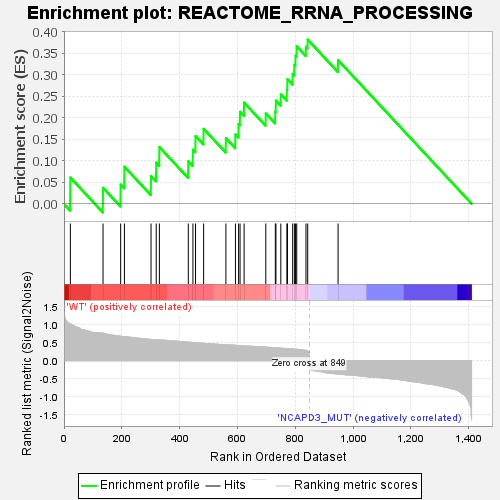

Supplement: Supplementary file 11 [file DataSheet2.ZIP › GSEA/Canonical pathways/my_analysis.Gsea.1599462267220/enplot_REACTOME_RRNA_PROCESSING_717.png]

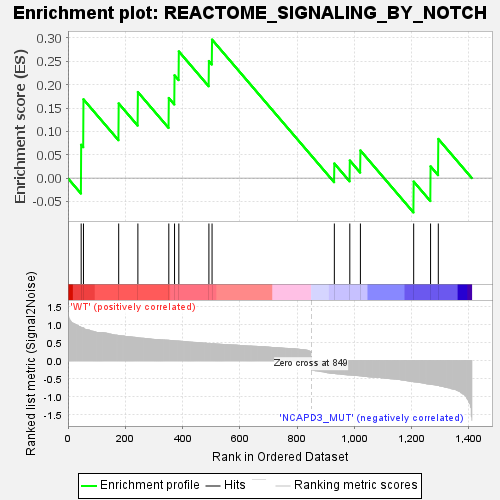

Supplement: Supplementary file 11 [file DataSheet2.ZIP › GSEA/Canonical pathways/my_analysis.Gsea.1599462267220/enplot_REACTOME_SIGNALING_BY_NOTCH_753.png]

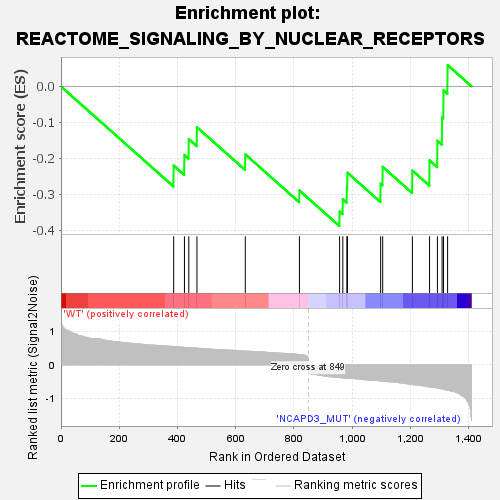

Supplement: Supplementary file 11 [file DataSheet2.ZIP › GSEA/Canonical pathways/my_analysis.Gsea.1599462267220/enplot_REACTOME_SIGNALING_BY_NUCLEAR_RECEPTORS_816.png]

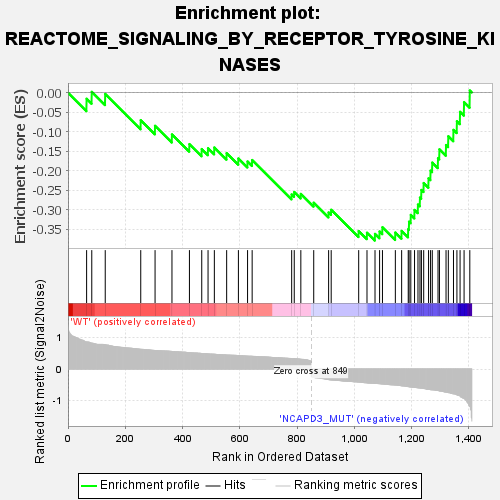

Supplement: Supplementary file 11 [file DataSheet2.ZIP › GSEA/Canonical pathways/my_analysis.Gsea.1599462267220/enplot_REACTOME_SIGNALING_BY_RECEPTOR_TYROSINE_KINASES_780.png]

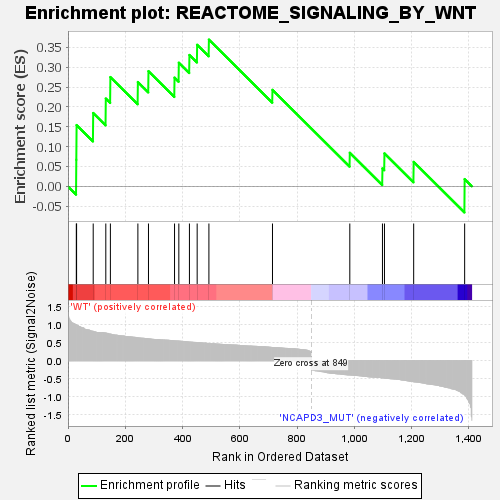

Supplement: Supplementary file 11 [file DataSheet2.ZIP › GSEA/Canonical pathways/my_analysis.Gsea.1599462267220/enplot_REACTOME_SIGNALING_BY_WNT_729.png]

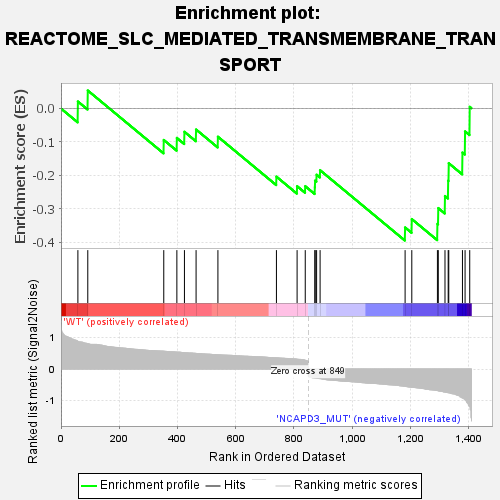

Supplement: Supplementary file 11 [file DataSheet2.ZIP › GSEA/Canonical pathways/my_analysis.Gsea.1599462267220/enplot_REACTOME_SLC_MEDIATED_TRANSMEMBRANE_TRANSPORT_798.png]

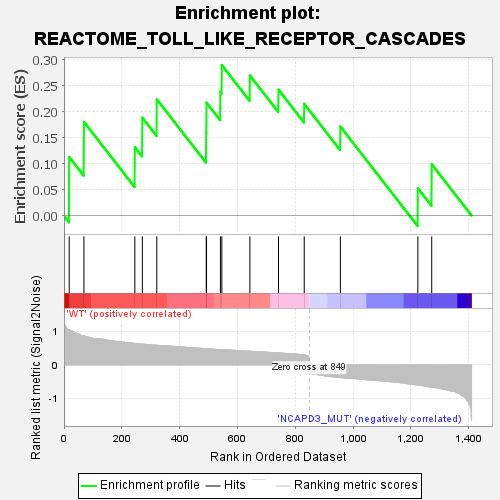

Supplement: Supplementary file 11 [file DataSheet2.ZIP › GSEA/Canonical pathways/my_analysis.Gsea.1599462267220/enplot_REACTOME_TOLL_LIKE_RECEPTOR_CASCADES_762.png]

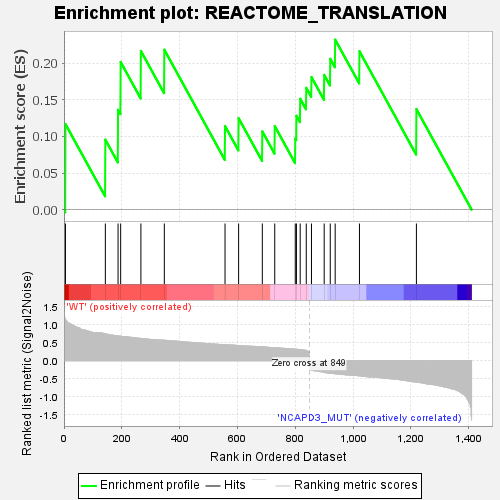

Supplement: Supplementary file 11 [file DataSheet2.ZIP › GSEA/Canonical pathways/my_analysis.Gsea.1599462267220/enplot_REACTOME_TRANSLATION_771.png]

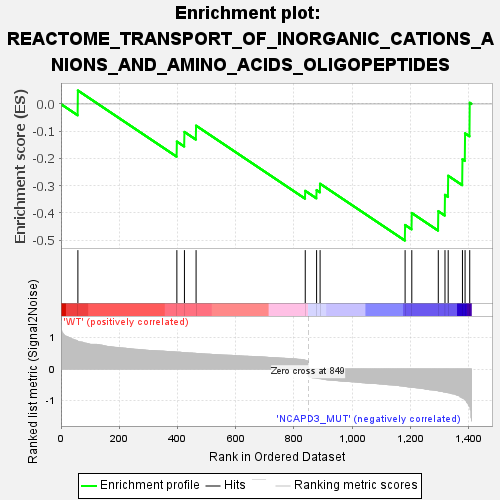

Supplement: Supplementary file 11 [file DataSheet2.ZIP › GSEA/Canonical pathways/my_analysis.Gsea.1599462267220/enplot_REACTOME_TRANSPORT_OF_INORGANIC_CATIONS_ANIONS_AND_AMINO_ACIDS_OLIGOPEPTIDES_789.png]

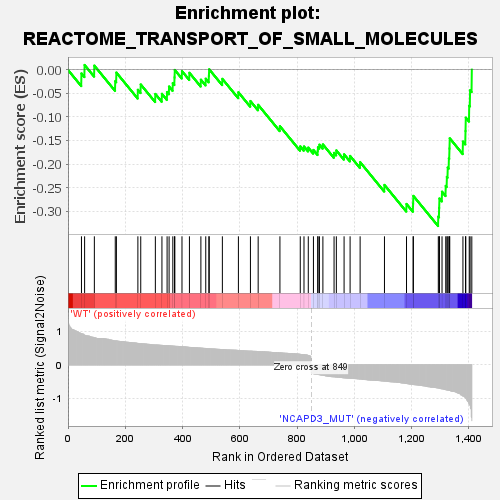

Supplement: Supplementary file 11 [file DataSheet2.ZIP › GSEA/Canonical pathways/my_analysis.Gsea.1599462267220/enplot_REACTOME_TRANSPORT_OF_SMALL_MOLECULES_783.png]

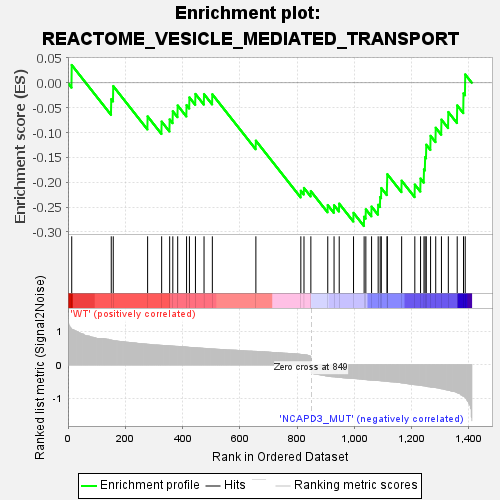

Supplement: Supplementary file 11 [file DataSheet2.ZIP › GSEA/Canonical pathways/my_analysis.Gsea.1599462267220/enplot_REACTOME_VESICLE_MEDIATED_TRANSPORT_810.png]

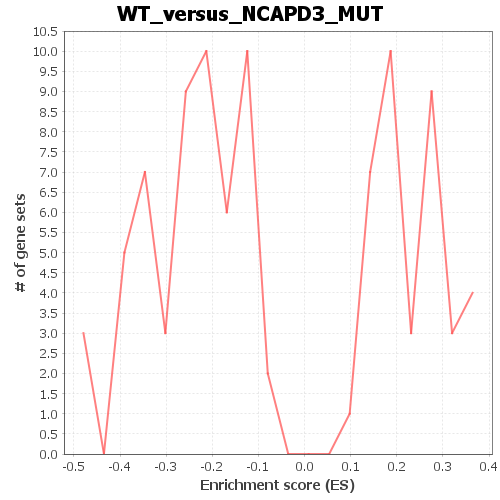

Supplement: Supplementary file 11 [file DataSheet2.ZIP › GSEA/Canonical pathways/my_analysis.Gsea.1599462267220/global_es_histogram.png]

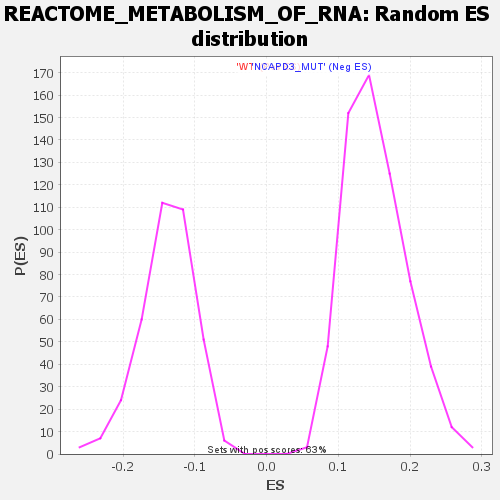

Supplement: Supplementary file 11 [file DataSheet2.ZIP › GSEA/Canonical pathways/my_analysis.Gsea.1599462267220/gset_rnd_es_dist_716.png]

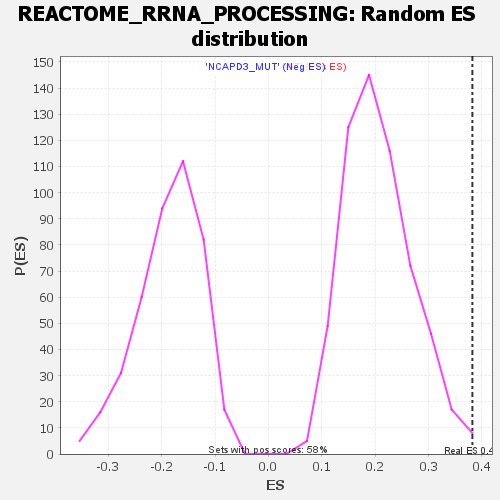

Supplement: Supplementary file 11 [file DataSheet2.ZIP › GSEA/Canonical pathways/my_analysis.Gsea.1599462267220/gset_rnd_es_dist_719.png]

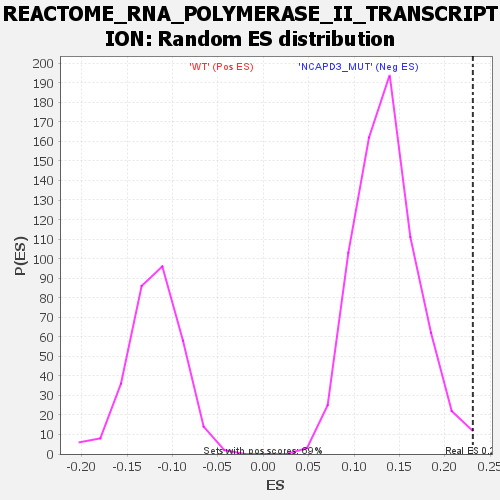

Supplement: Supplementary file 11 [file DataSheet2.ZIP › GSEA/Canonical pathways/my_analysis.Gsea.1599462267220/gset_rnd_es_dist_722.png]

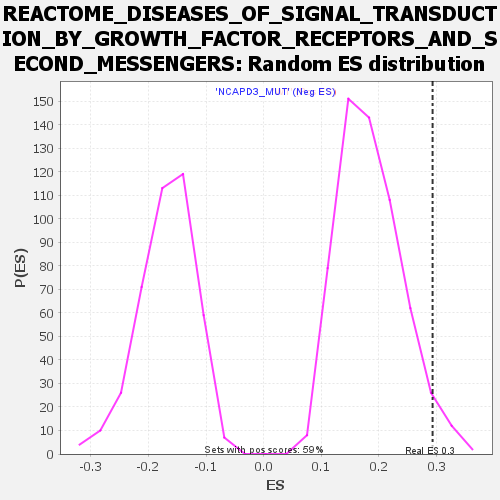

Supplement: Supplementary file 11 [file DataSheet2.ZIP › GSEA/Canonical pathways/my_analysis.Gsea.1599462267220/gset_rnd_es_dist_725.png]

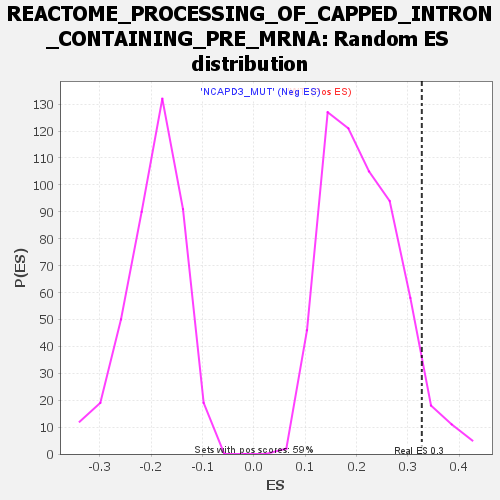

Supplement: Supplementary file 11 [file DataSheet2.ZIP › GSEA/Canonical pathways/my_analysis.Gsea.1599462267220/gset_rnd_es_dist_728.png]

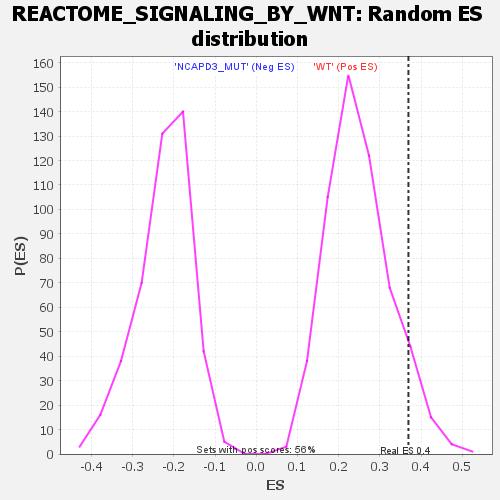

Supplement: Supplementary file 11 [file DataSheet2.ZIP › GSEA/Canonical pathways/my_analysis.Gsea.1599462267220/gset_rnd_es_dist_731.png]

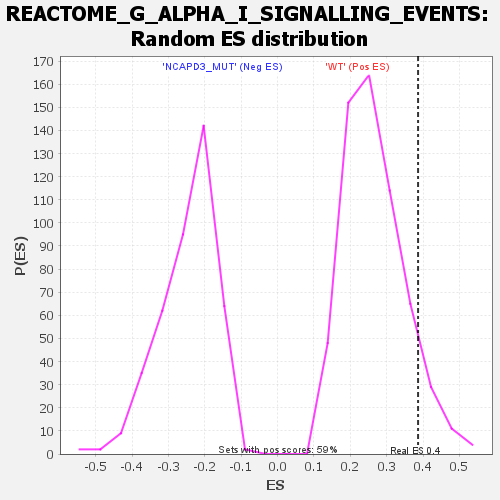

Supplement: Supplementary file 11 [file DataSheet2.ZIP › GSEA/Canonical pathways/my_analysis.Gsea.1599462267220/gset_rnd_es_dist_734.png]

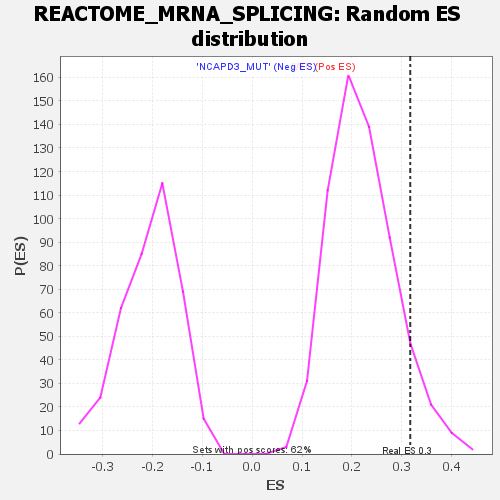

Supplement: Supplementary file 11 [file DataSheet2.ZIP › GSEA/Canonical pathways/my_analysis.Gsea.1599462267220/gset_rnd_es_dist_737.png]

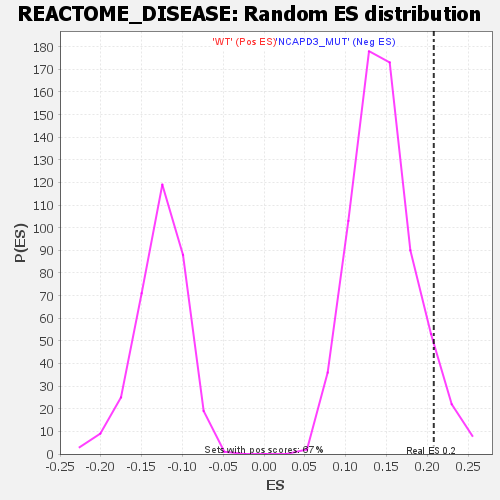

Supplement: Supplementary file 11 [file DataSheet2.ZIP › GSEA/Canonical pathways/my_analysis.Gsea.1599462267220/gset_rnd_es_dist_740.png]

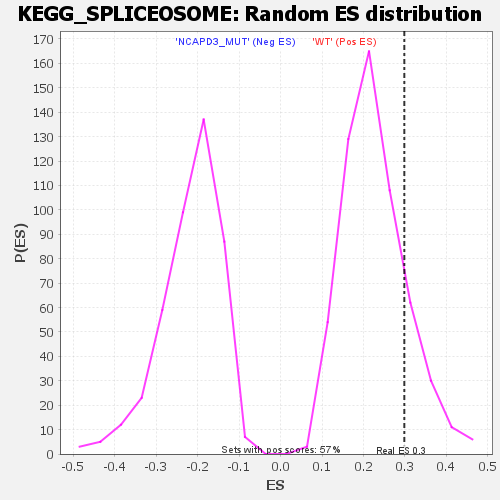

Supplement: Supplementary file 11 [file DataSheet2.ZIP › GSEA/Canonical pathways/my_analysis.Gsea.1599462267220/gset_rnd_es_dist_743.png]

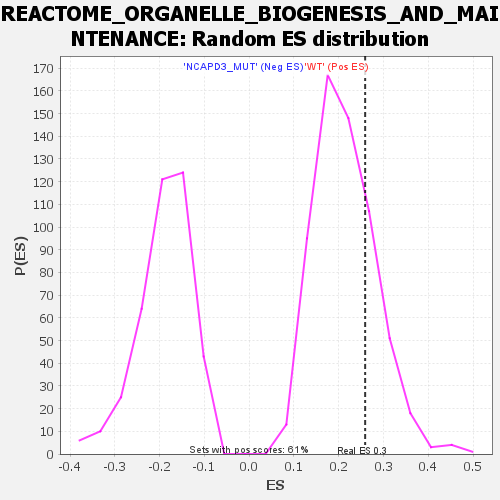

Supplement: Supplementary file 11 [file DataSheet2.ZIP › GSEA/Canonical pathways/my_analysis.Gsea.1599462267220/gset_rnd_es_dist_746.png]

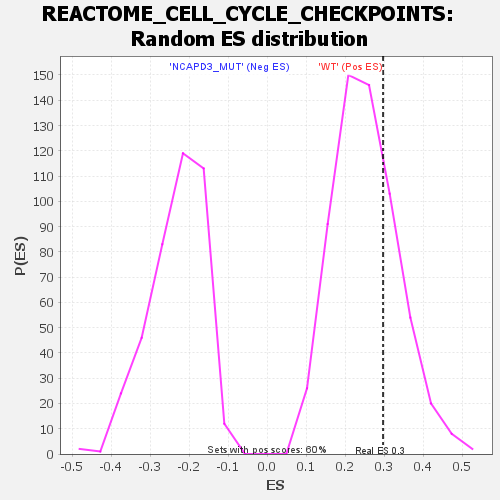

Supplement: Supplementary file 11 [file DataSheet2.ZIP › GSEA/Canonical pathways/my_analysis.Gsea.1599462267220/gset_rnd_es_dist_749.png]

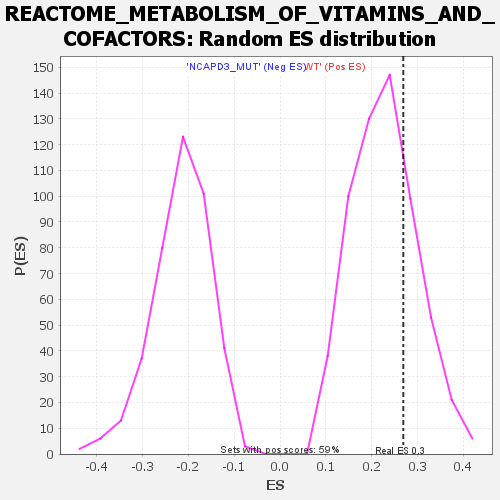

Supplement: Supplementary file 11 [file DataSheet2.ZIP › GSEA/Canonical pathways/my_analysis.Gsea.1599462267220/gset_rnd_es_dist_752.png]

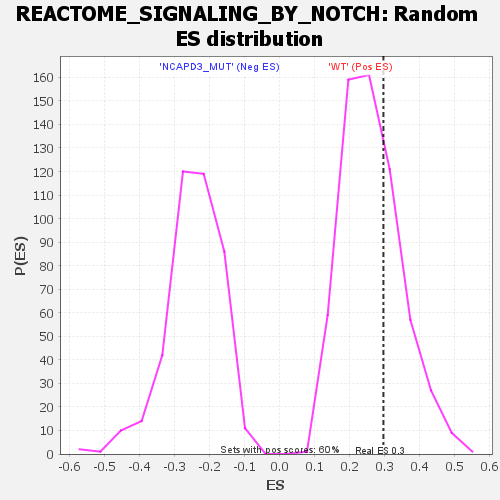

Supplement: Supplementary file 11 [file DataSheet2.ZIP › GSEA/Canonical pathways/my_analysis.Gsea.1599462267220/gset_rnd_es_dist_755.png]

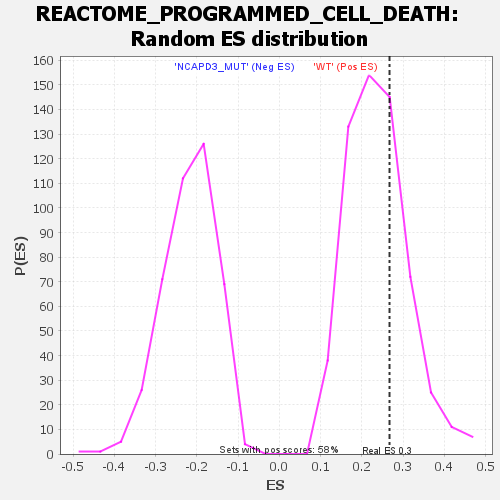

Supplement: Supplementary file 11 [file DataSheet2.ZIP › GSEA/Canonical pathways/my_analysis.Gsea.1599462267220/gset_rnd_es_dist_758.png]

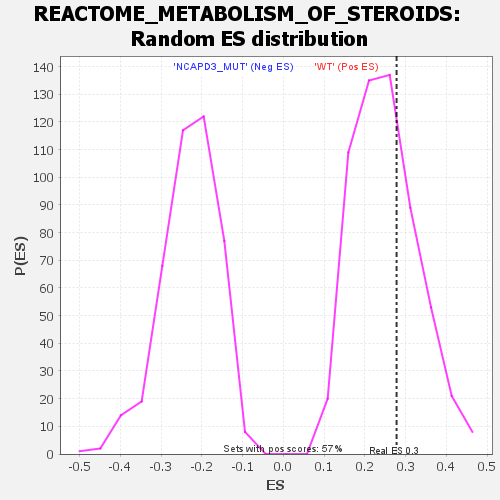

Supplement: Supplementary file 11 [file DataSheet2.ZIP › GSEA/Canonical pathways/my_analysis.Gsea.1599462267220/gset_rnd_es_dist_761.png]

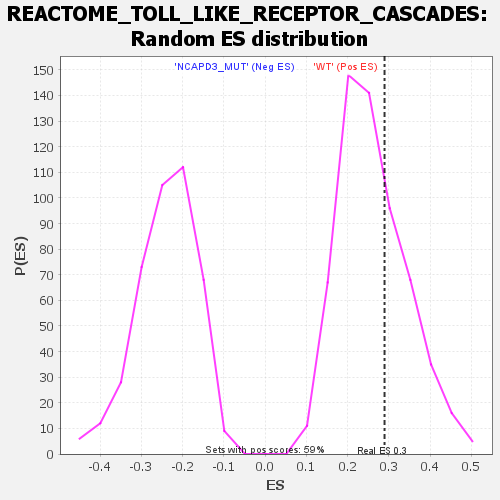

Supplement: Supplementary file 11 [file DataSheet2.ZIP › GSEA/Canonical pathways/my_analysis.Gsea.1599462267220/gset_rnd_es_dist_764.png]

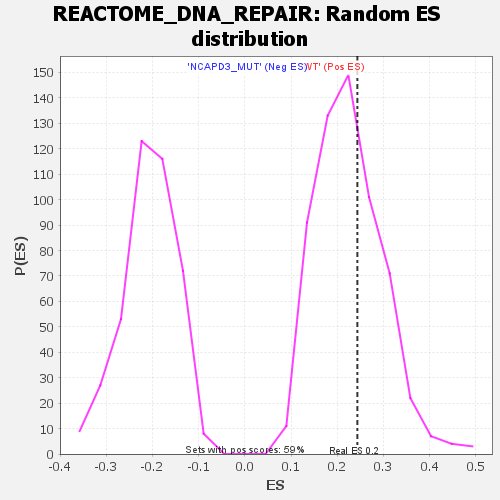

Supplement: Supplementary file 11 [file DataSheet2.ZIP › GSEA/Canonical pathways/my_analysis.Gsea.1599462267220/gset_rnd_es_dist_767.png]

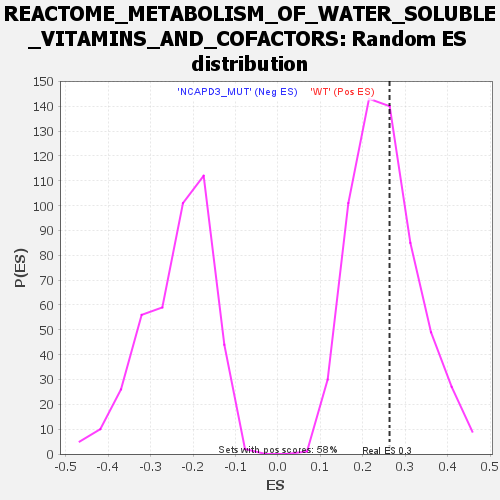

Supplement: Supplementary file 11 [file DataSheet2.ZIP › GSEA/Canonical pathways/my_analysis.Gsea.1599462267220/gset_rnd_es_dist_770.png]

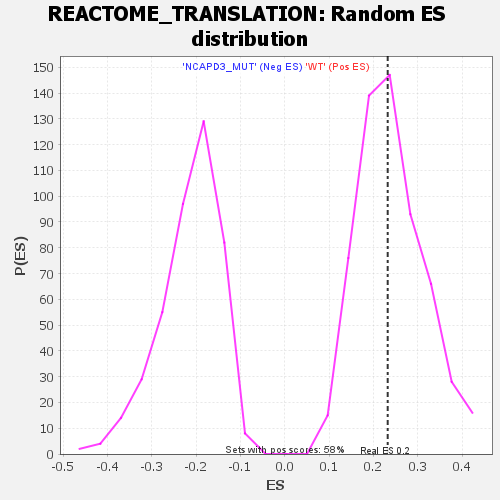

Supplement: Supplementary file 11 [file DataSheet2.ZIP › GSEA/Canonical pathways/my_analysis.Gsea.1599462267220/gset_rnd_es_dist_773.png]

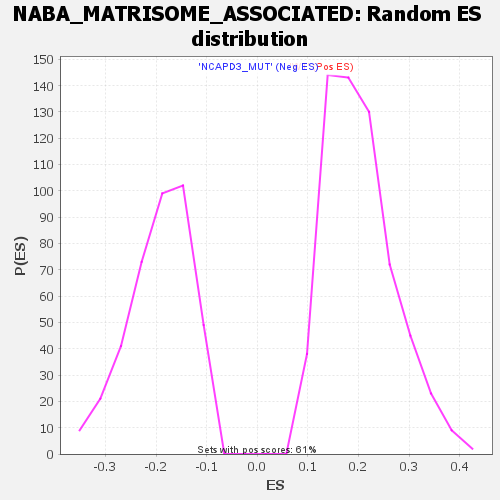

Supplement: Supplementary file 11 [file DataSheet2.ZIP › GSEA/Canonical pathways/my_analysis.Gsea.1599462267220/gset_rnd_es_dist_776.png]

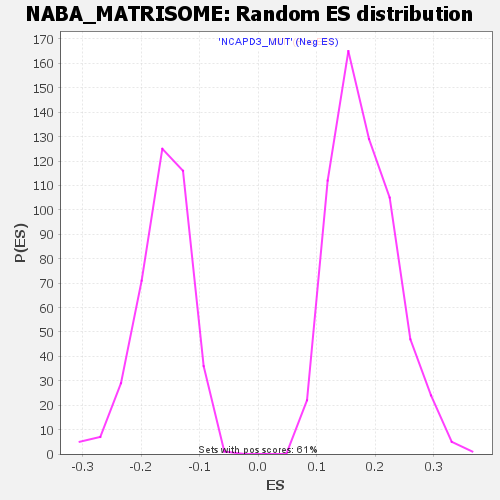

Supplement: Supplementary file 11 [file DataSheet2.ZIP › GSEA/Canonical pathways/my_analysis.Gsea.1599462267220/gset_rnd_es_dist_779.png]

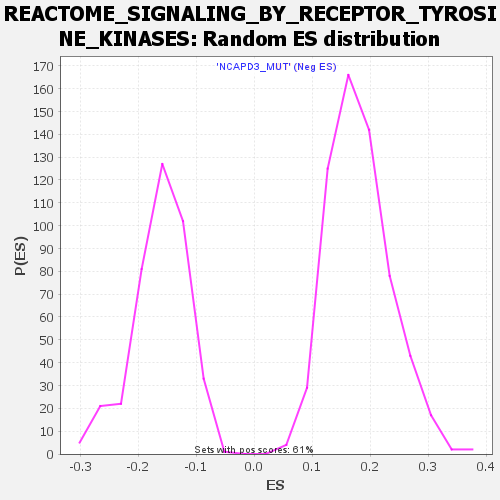

Supplement: Supplementary file 11 [file DataSheet2.ZIP › GSEA/Canonical pathways/my_analysis.Gsea.1599462267220/gset_rnd_es_dist_782.png]

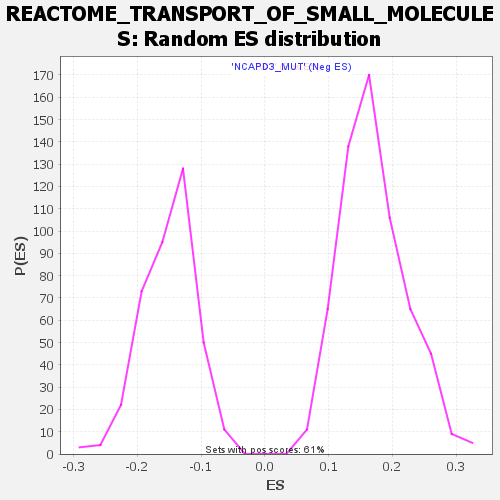

Supplement: Supplementary file 11 [file DataSheet2.ZIP › GSEA/Canonical pathways/my_analysis.Gsea.1599462267220/gset_rnd_es_dist_785.png]

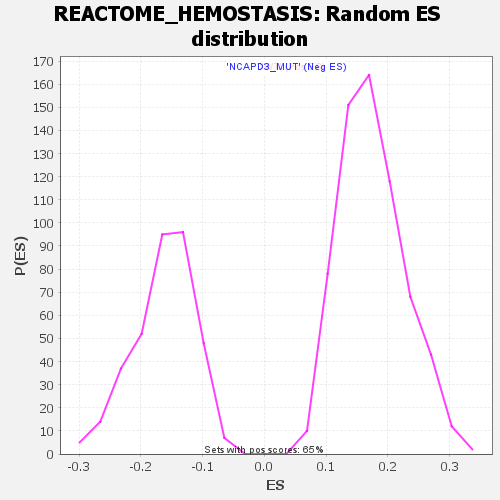

Supplement: Supplementary file 11 [file DataSheet2.ZIP › GSEA/Canonical pathways/my_analysis.Gsea.1599462267220/gset_rnd_es_dist_788.png]

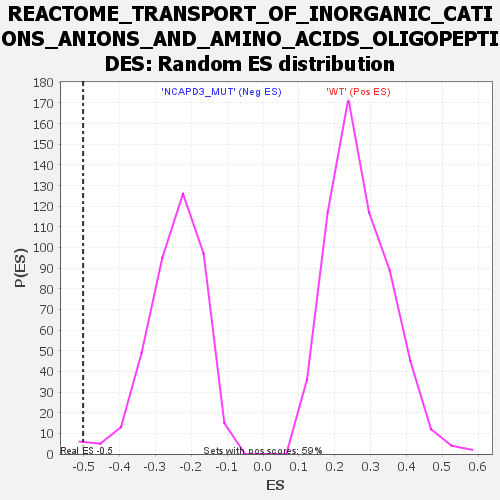

Supplement: Supplementary file 11 [file DataSheet2.ZIP › GSEA/Canonical pathways/my_analysis.Gsea.1599462267220/gset_rnd_es_dist_791.png]

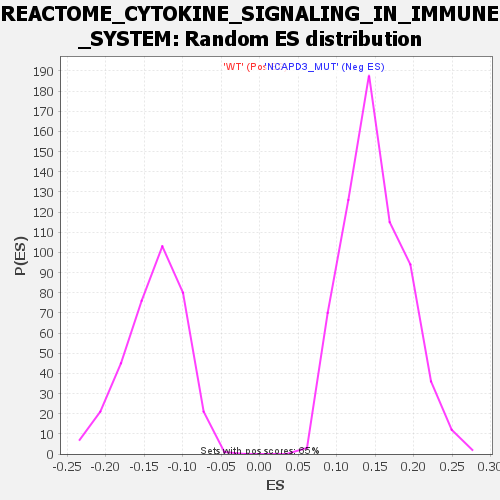

Supplement: Supplementary file 11 [file DataSheet2.ZIP › GSEA/Canonical pathways/my_analysis.Gsea.1599462267220/gset_rnd_es_dist_794.png]

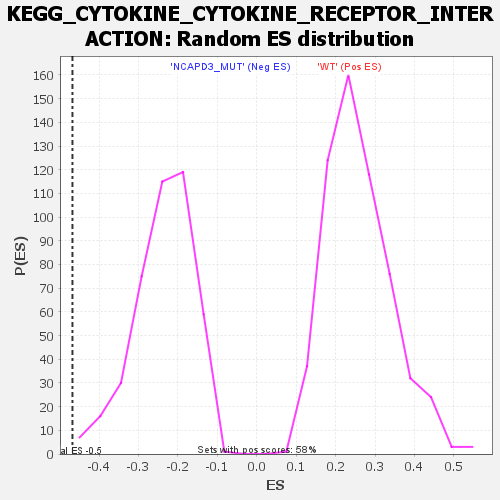

Supplement: Supplementary file 11 [file DataSheet2.ZIP › GSEA/Canonical pathways/my_analysis.Gsea.1599462267220/gset_rnd_es_dist_797.png]

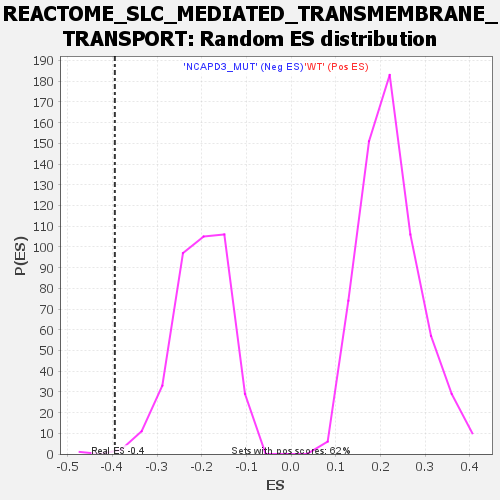

Supplement: Supplementary file 11 [file DataSheet2.ZIP › GSEA/Canonical pathways/my_analysis.Gsea.1599462267220/gset_rnd_es_dist_800.png]

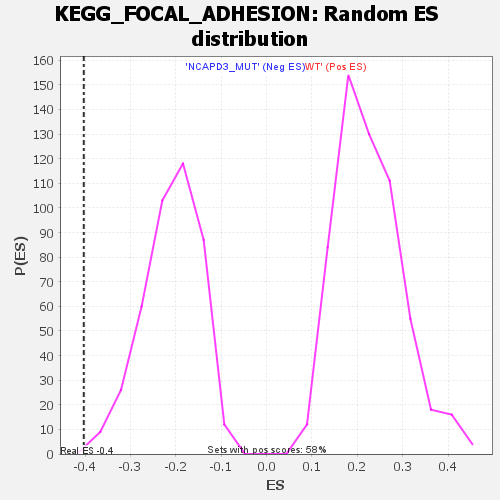

Supplement: Supplementary file 11 [file DataSheet2.ZIP › GSEA/Canonical pathways/my_analysis.Gsea.1599462267220/gset_rnd_es_dist_803.png]

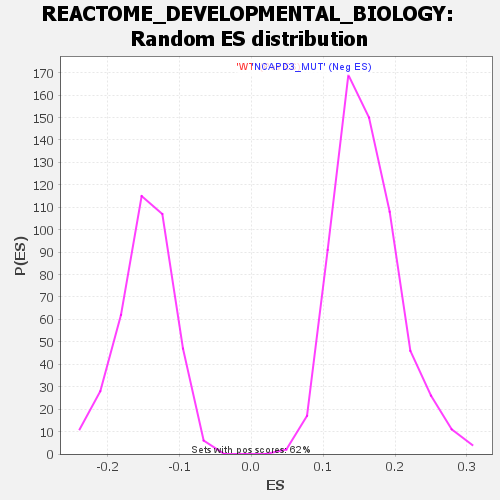

Supplement: Supplementary file 11 [file DataSheet2.ZIP › GSEA/Canonical pathways/my_analysis.Gsea.1599462267220/gset_rnd_es_dist_806.png]

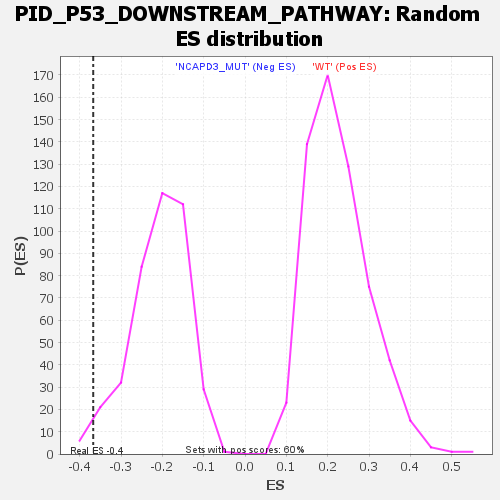

Supplement: Supplementary file 11 [file DataSheet2.ZIP › GSEA/Canonical pathways/my_analysis.Gsea.1599462267220/gset_rnd_es_dist_809.png]

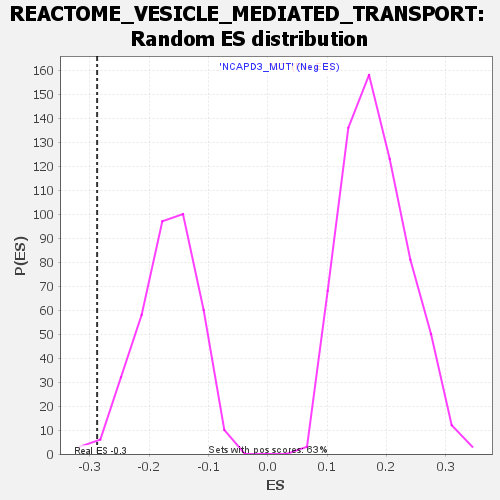

Supplement: Supplementary file 11 [file DataSheet2.ZIP › GSEA/Canonical pathways/my_analysis.Gsea.1599462267220/gset_rnd_es_dist_812.png]
