# Supplementary material for: Role and mechanism of NCAPD3 in promoting malignant behaviors in gastric cancer
Source: Front Pharmacol. 2024 Apr 22;15:1341039. doi: 10.3389/fphar.2024.1341039 (PMC11070777; doi:10.3389/fphar.2024.1341039)
Supplement: Supplementary file 5 [file Table1.docx]

| Supplementary Table 1. The sequences of shRNAs/sgRNAs | |
| --- | --- |
| shRNAs/sgRNAs | Target Sequences |
| Negative Control | CTCTGTGATTAGAGCACAT |
| *NCAPD3*-1 | TCCATGTTCTCCAGTAGCAA |
| *NCAPD3*-2 | AATCCATAATAAGCCAGTTC |
| *NCAPD3*-3 | AATGTCATGTTACACAAGCC |

| Supplementary Table 2. The primer sequences and enzyme digestion information needed for effective sgRNA screening |
| --- |
| \| **sgRNAs** \| **Primer sequence (5–3′)** \| **Amplified fragment (bp)** \| **Endonuclea fragement 1 (bp)** \| **Endonuclea fragement 2 (bp)** \| \| --- \| --- \| --- \| --- \| --- \| \| *NCAPD3*-sgRNA1 \| F:ATGTGAATCGTGACTAGTAAAC \| 389 \| 243 \| 146 \| \| R:TACTCATATTGTACTCCCTGTG \| \| *NCAPD3*-sgRNA2 \| F:CTTCAAAGGATTAGACTATGG \| 530 \| 361 \| 169 \| \| R:GAGCTAGTAAATTGAAATGGCA \| \| *NCAPD3*-sgRNA3 \| F:CTTCAAAGGATTAGACTATGG \| 530 \| 132 \| 398 \| \| R: GAGCTAGTAAATTGAAATGGCA \| |

| Supplementary Table 3. The primer sequences of *NCAPD*3 and GAPDH | | |
| --- | --- | --- |
| Gene | Forward primer sequence | Reward peimer sequence |
| *NCAPD3* | 5'- CCTATTTCCCATGATTCCTTCATA -3' | 5'- GTAATACGGTTATCCACGCG -3' |
| GAPDH | 5’-GCGGGGCTCCAGAACATCAT-3’ | 5’-CCAGCCCCAGCGTCAAGGTG-3’ |
