# Supplementary material for: Role and mechanism of NCAPD3 in promoting malignant behaviors in gastric cancer
Source: Front Pharmacol. 2024 Apr 22;15:1341039. doi: 10.3389/fphar.2024.1341039 (PMC11070777; doi:10.3389/fphar.2024.1341039)
Supplement: Supplementary file 11 [file DataSheet2.ZIP › GSEA/Canonical pathways/my_analysis.Gsea.1599462267220/gsea_report_for_NCAPD3_MUT_1599462267220.html]

Report for NCAPD3\_MUT 1599462267220 [GSEA]

| GS  follow link to MSigDB | GS DETAILS | SIZE | ES | NES | NOM p-val | FDR q-val | FWER p-val | RANK AT MAX | LEADING EDGE || 1 | NABA\_MATRISOME\_ASSOCIATED | Details ... | 30 | -0.47 | -2.45 | 0.000 | 0.006 | 0.004 | 163 | tags=47%, list=12%, signal=52% |
| 2 | NABA\_MATRISOME | Details ... | 48 | -0.38 | -2.34 | 0.000 | 0.006 | 0.009 | 174 | tags=40%, list=12%, signal=44% |
| 3 | REACTOME\_SIGNALING\_BY\_RECEPTOR\_TYROSINE\_KINASES | Details ... | 47 | -0.38 | -2.34 | 0.000 | 0.004 | 0.009 | 340 | tags=53%, list=24%, signal=68% |
| 4 | REACTOME\_TRANSPORT\_OF\_SMALL\_MOLECULES | Details ... | 58 | -0.33 | -2.19 | 0.000 | 0.012 | 0.031 | 119 | tags=28%, list=8%, signal=29% |
| 5 | REACTOME\_HEMOSTASIS | Details ... | 45 | -0.33 | -2.04 | 0.000 | 0.024 | 0.076 | 468 | tags=62%, list=33%, signal=90% |
| 6 | REACTOME\_TRANSPORT\_OF\_INORGANIC\_CATIONS\_ANIONS\_AND\_AMINO\_ACIDS\_OLIGOPEPTIDES | Details ... | 15 | -0.50 | -2.04 | 0.010 | 0.020 | 0.077 | 230 | tags=53%, list=16%, signal=63% |
| 7 | REACTOME\_CYTOKINE\_SIGNALING\_IN\_IMMUNE\_SYSTEM | Details ... | 79 | -0.27 | -2.01 | 0.000 | 0.021 | 0.092 | 317 | tags=39%, list=22%, signal=48% |
| 8 | KEGG\_CYTOKINE\_CYTOKINE\_RECEPTOR\_INTERACTION | Details ... | 16 | -0.47 | -1.98 | 0.002 | 0.024 | 0.121 | 146 | tags=50%, list=10%, signal=55% |
| 9 | REACTOME\_SLC\_MEDIATED\_TRANSMEMBRANE\_TRANSPORT | Details ... | 24 | -0.39 | -1.95 | 0.003 | 0.028 | 0.156 | 230 | tags=42%, list=16%, signal=49% |
| 10 | KEGG\_FOCAL\_ADHESION | Details ... | 22 | -0.40 | -1.91 | 0.002 | 0.032 | 0.197 | 493 | tags=68%, list=35%, signal=103% |
| 11 | REACTOME\_DEVELOPMENTAL\_BIOLOGY | Details ... | 66 | -0.28 | -1.87 | 0.000 | 0.039 | 0.257 | 298 | tags=38%, list=21%, signal=46% |
| 12 | PID\_P53\_DOWNSTREAM\_PATHWAY | Details ... | 22 | -0.37 | -1.77 | 0.020 | 0.065 | 0.409 | 335 | tags=59%, list=24%, signal=76% |
| 13 | REACTOME\_VESICLE\_MEDIATED\_TRANSPORT | Details ... | 42 | -0.29 | -1.70 | 0.016 | 0.090 | 0.552 | 378 | tags=50%, list=27%, signal=66% |
| 14 | REACTOME\_MEMBRANE\_TRAFFICKING | Details ... | 41 | -0.28 | -1.64 | 0.028 | 0.122 | 0.694 | 378 | tags=49%, list=27%, signal=65% |
| 15 | REACTOME\_SIGNALING\_BY\_NUCLEAR\_RECEPTORS | Details ... | 18 | -0.39 | -1.63 | 0.030 | 0.116 | 0.699 | 455 | tags=67%, list=32%, signal=97% |
| 16 | KEGG\_MAPK\_SIGNALING\_PATHWAY | Details ... | 23 | -0.34 | -1.63 | 0.042 | 0.109 | 0.700 | 564 | tags=74%, list=40%, signal=121% |
| 17 | REACTOME\_NEGATIVE\_REGULATION\_OF\_THE\_PI3K\_AKT\_NETWORK | Details ... | 17 | -0.36 | -1.56 | 0.060 | 0.152 | 0.828 | 146 | tags=41%, list=10%, signal=45% |
| 18 | KEGG\_REGULATION\_OF\_ACTIN\_CYTOSKELETON | Details ... | 16 | -0.36 | -1.55 | 0.053 | 0.153 | 0.848 | 269 | tags=44%, list=19%, signal=53% |
| 19 | REACTOME\_NERVOUS\_SYSTEM\_DEVELOPMENT | Details ... | 37 | -0.26 | -1.49 | 0.065 | 0.192 | 0.914 | 319 | tags=41%, list=23%, signal=51% |
| 20 | REACTOME\_DEATH\_RECEPTOR\_SIGNALLING | Details ... | 15 | -0.36 | -1.47 | 0.083 | 0.201 | 0.929 | 385 | tags=60%, list=27%, signal=82% |
| 21 | REACTOME\_EXTRACELLULAR\_MATRIX\_ORGANIZATION |  | 25 | -0.29 | -1.41 | 0.099 | 0.247 | 0.970 | 554 | tags=72%, list=39%, signal=116% |
| 22 | REACTOME\_FLT3\_SIGNALING |  | 26 | -0.27 | -1.38 | 0.106 | 0.267 | 0.985 | 146 | tags=31%, list=10%, signal=34% |
| 23 | REACTOME\_SIGNALING\_BY\_INTERLEUKINS |  | 34 | -0.23 | -1.31 | 0.149 | 0.349 | 0.996 | 87 | tags=18%, list=6%, signal=18% |
| 24 | REACTOME\_MAPK\_FAMILY\_SIGNALING\_CASCADES |  | 24 | -0.26 | -1.28 | 0.158 | 0.373 | 0.996 | 146 | tags=29%, list=10%, signal=32% |
| 25 | REACTOME\_PI3K\_AKT\_SIGNALING\_IN\_CANCER |  | 15 | -0.31 | -1.27 | 0.187 | 0.385 | 0.997 | 221 | tags=40%, list=16%, signal=47% |
| 26 | REACTOME\_INTRACELLULAR\_SIGNALING\_BY\_SECOND\_MESSENGERS |  | 39 | -0.21 | -1.20 | 0.225 | 0.488 | 0.999 | 146 | tags=23%, list=10%, signal=25% |
| 27 | NABA\_CORE\_MATRISOME |  | 18 | -0.26 | -1.16 | 0.277 | 0.533 | 1.000 | 493 | tags=67%, list=35%, signal=101% |
| 28 | REACTOME\_SIGNALING\_BY\_RHO\_GTPASES |  | 19 | -0.25 | -1.15 | 0.272 | 0.535 | 1.000 | 292 | tags=42%, list=21%, signal=52% |
| 29 | REACTOME\_NEUTROPHIL\_DEGRANULATION |  | 40 | -0.19 | -1.13 | 0.296 | 0.560 | 1.000 | 468 | tags=48%, list=33%, signal=69% |
| 30 | REACTOME\_CELL\_CELL\_COMMUNICATION |  | 16 | -0.27 | -1.12 | 0.284 | 0.559 | 1.000 | 397 | tags=56%, list=28%, signal=77% |
| 31 | KEGG\_PATHWAYS\_IN\_CANCER |  | 32 | -0.20 | -1.10 | 0.341 | 0.572 | 1.000 | 247 | tags=28%, list=18%, signal=33% |
| 32 | REACTOME\_POST\_TRANSLATIONAL\_PROTEIN\_MODIFICATION |  | 120 | -0.13 | -1.09 | 0.332 | 0.575 | 1.000 | 298 | tags=28%, list=21%, signal=32% |
| 33 | KEGG\_ENDOCYTOSIS |  | 19 | -0.23 | -1.04 | 0.383 | 0.663 | 1.000 | 191 | tags=26%, list=14%, signal=30% |
| 34 | REACTOME\_INNATE\_IMMUNE\_SYSTEM |  | 81 | -0.13 | -0.95 | 0.532 | 0.827 | 1.000 | 468 | tags=42%, list=33%, signal=59% |
| 35 | REACTOME\_RESPONSE\_TO\_ELEVATED\_PLATELET\_CYTOSOLIC\_CA2 |  | 15 | -0.23 | -0.95 | 0.534 | 0.824 | 1.000 | 415 | tags=53%, list=29%, signal=75% |
| 36 | REACTOME\_SIGNALING\_BY\_GPCR |  | 34 | -0.17 | -0.94 | 0.552 | 0.812 | 1.000 | 322 | tags=35%, list=23%, signal=45% |
| 37 | REACTOME\_CLASS\_I\_MHC\_MEDIATED\_ANTIGEN\_PROCESSING\_PRESENTATION |  | 39 | -0.16 | -0.94 | 0.531 | 0.799 | 1.000 | 164 | tags=21%, list=12%, signal=23% |
| 38 | REACTOME\_REGULATION\_OF\_TP53\_ACTIVITY |  | 16 | -0.22 | -0.92 | 0.561 | 0.803 | 1.000 | 379 | tags=50%, list=27%, signal=68% |
| 39 | REACTOME\_ADAPTIVE\_IMMUNE\_SYSTEM |  | 53 | -0.14 | -0.90 | 0.598 | 0.825 | 1.000 | 164 | tags=19%, list=12%, signal=21% |
| 40 | REACTOME\_ASPARAGINE\_N\_LINKED\_GLYCOSYLATION |  | 19 | -0.20 | -0.88 | 0.601 | 0.841 | 1.000 | 415 | tags=47%, list=29%, signal=66% |
| 41 | REACTOME\_ANTIGEN\_PROCESSING\_UBIQUITINATION\_PROTEASOME\_DEGRADATION |  | 34 | -0.16 | -0.88 | 0.618 | 0.833 | 1.000 | 164 | tags=21%, list=12%, signal=23% |
| 42 | REACTOME\_INTERFERON\_SIGNALING |  | 25 | -0.17 | -0.86 | 0.658 | 0.854 | 1.000 | 55 | tags=12%, list=4%, signal=12% |
| 43 | REACTOME\_PLATELET\_ACTIVATION\_SIGNALING\_AND\_AGGREGATION |  | 18 | -0.20 | -0.85 | 0.646 | 0.844 | 1.000 | 415 | tags=50%, list=29%, signal=70% |
| 44 | NABA\_ECM\_GLYCOPROTEINS |  | 15 | -0.20 | -0.81 | 0.689 | 0.900 | 1.000 | 170 | tags=27%, list=12%, signal=30% |
| 45 | REACTOME\_NEURONAL\_SYSTEM |  | 22 | -0.15 | -0.74 | 0.785 | 0.992 | 1.000 | 325 | tags=36%, list=23%, signal=47% |
| 46 | REACTOME\_METABOLISM\_OF\_LIPIDS |  | 59 | -0.10 | -0.68 | 0.869 | 1.000 | 1.000 | 1150 | tags=95%, list=82%, signal=492% |
| 47 | REACTOME\_CELLULAR\_SENESCENCE |  | 17 | -0.16 | -0.67 | 0.853 | 1.000 | 1.000 | 449 | tags=47%, list=32%, signal=68% |
| 48 | REACTOME\_PHOSPHOLIPID\_METABOLISM |  | 23 | -0.13 | -0.66 | 0.899 | 1.000 | 1.000 | 1127 | tags=96%, list=80%, signal=467% |
| 49 | REACTOME\_INFECTIOUS\_DISEASE |  | 34 | -0.12 | -0.64 | 0.913 | 1.000 | 1.000 | 237 | tags=24%, list=17%, signal=28% |
| 50 | REACTOME\_METABOLISM\_OF\_AMINO\_ACIDS\_AND\_DERIVATIVES |  | 28 | -0.12 | -0.62 | 0.941 | 1.000 | 1.000 | 562 | tags=57%, list=40%, signal=93% |
| 51 | REACTOME\_CELL\_CYCLE |  | 49 | -0.09 | -0.59 | 0.942 | 1.000 | 1.000 | 1285 | tags=100%, list=91%, signal=1081% |
| 52 | REACTOME\_GLYCEROPHOSPHOLIPID\_BIOSYNTHESIS |  | 15 | -0.14 | -0.56 | 0.941 | 1.000 | 1.000 | 742 | tags=73%, list=53%, signal=153% |
| 53 | KEGG\_APOPTOSIS |  | 16 | -0.14 | -0.56 | 0.956 | 0.999 | 1.000 | 945 | tags=88%, list=67%, signal=262% |
| 54 | REACTOME\_CELL\_CYCLE\_MITOTIC |  | 34 | -0.09 | -0.52 | 0.977 | 0.998 | 1.000 | 1285 | tags=100%, list=91%, signal=1093% |
| 55 | KEGG\_CELL\_CYCLE |  | 17 | -0.11 | -0.48 | 0.990 | 0.990 | 1.000 | 524 | tags=53%, list=37%, signal=83% |
Table: Gene sets enriched in phenotype **NCAPD3\_MUT (3 samples)**[plain text format]****

  
