# Supplementary material for: Role and mechanism of NCAPD3 in promoting malignant behaviors in gastric cancer
Source: Front Pharmacol. 2024 Apr 22;15:1341039. doi: 10.3389/fphar.2024.1341039 (PMC11070777; doi:10.3389/fphar.2024.1341039)
Supplement: Supplementary file 11 [file DataSheet2.ZIP › GSEA/Canonical pathways/my_analysis.Gsea.1599462267220/gsea_report_for_WT_1599462267220.html]

Report for WT 1599462267220 [GSEA]

| GS  follow link to MSigDB | GS DETAILS | SIZE | ES | NES | NOM p-val | FDR q-val | FWER p-val | RANK AT MAX | LEADING EDGE || 1 | REACTOME\_METABOLISM\_OF\_RNA | Details ... | 81 | 0.35 | 2.31 | 0.000 | 0.003 | 0.004 | 868 | tags=95%, list=62%, signal=233% |
| 2 | REACTOME\_RRNA\_PROCESSING | Details ... | 29 | 0.38 | 1.85 | 0.002 | 0.131 | 0.332 | 843 | tags=97%, list=60%, signal=235% |
| 3 | REACTOME\_RNA\_POLYMERASE\_II\_TRANSCRIPTION | Details ... | 135 | 0.23 | 1.70 | 0.004 | 0.254 | 0.686 | 389 | tags=40%, list=28%, signal=50% |
| 4 | REACTOME\_DISEASES\_OF\_SIGNAL\_TRANSDUCTION\_BY\_GROWTH\_FACTOR\_RECEPTORS\_AND\_SECOND\_MESSENGERS | Details ... | 39 | 0.29 | 1.58 | 0.041 | 0.383 | 0.920 | 492 | tags=59%, list=35%, signal=88% |
| 5 | REACTOME\_PROCESSING\_OF\_CAPPED\_INTRON\_CONTAINING\_PRE\_MRNA | Details ... | 27 | 0.33 | 1.54 | 0.058 | 0.374 | 0.943 | 848 | tags=93%, list=60%, signal=228% |
| 6 | REACTOME\_SIGNALING\_BY\_WNT | Details ... | 18 | 0.37 | 1.48 | 0.074 | 0.441 | 0.982 | 492 | tags=67%, list=35%, signal=101% |
| 7 | REACTOME\_G\_ALPHA\_I\_SIGNALLING\_EVENTS | Details ... | 15 | 0.39 | 1.46 | 0.082 | 0.419 | 0.987 | 477 | tags=60%, list=34%, signal=90% |
| 8 | REACTOME\_MRNA\_SPLICING | Details ... | 24 | 0.32 | 1.43 | 0.079 | 0.409 | 0.996 | 848 | tags=92%, list=60%, signal=226% |
| 9 | REACTOME\_DISEASE | Details ... | 104 | 0.21 | 1.42 | 0.060 | 0.381 | 0.997 | 492 | tags=48%, list=35%, signal=68% |
| 10 | KEGG\_SPLICEOSOME | Details ... | 20 | 0.30 | 1.31 | 0.155 | 0.563 | 1.000 | 848 | tags=90%, list=60%, signal=222% |
| 11 | REACTOME\_ORGANELLE\_BIOGENESIS\_AND\_MAINTENANCE | Details ... | 28 | 0.26 | 1.22 | 0.232 | 0.761 | 1.000 | 625 | tags=61%, list=44%, signal=107% |
| 12 | REACTOME\_CELL\_CYCLE\_CHECKPOINTS | Details ... | 17 | 0.30 | 1.18 | 0.265 | 0.800 | 1.000 | 372 | tags=47%, list=26%, signal=63% |
| 13 | REACTOME\_METABOLISM\_OF\_VITAMINS\_AND\_COFACTORS | Details ... | 22 | 0.27 | 1.18 | 0.276 | 0.739 | 1.000 | 424 | tags=45%, list=30%, signal=64% |
| 14 | REACTOME\_SIGNALING\_BY\_NOTCH | Details ... | 15 | 0.30 | 1.13 | 0.318 | 0.814 | 1.000 | 503 | tags=60%, list=36%, signal=92% |
| 15 | REACTOME\_PROGRAMMED\_CELL\_DEATH | Details ... | 20 | 0.27 | 1.12 | 0.315 | 0.784 | 1.000 | 548 | tags=55%, list=39%, signal=89% |
| 16 | REACTOME\_METABOLISM\_OF\_STEROIDS | Details ... | 17 | 0.28 | 1.11 | 0.337 | 0.754 | 1.000 | 903 | tags=94%, list=64%, signal=258% |
| 17 | REACTOME\_TOLL\_LIKE\_RECEPTOR\_CASCADES | Details ... | 15 | 0.29 | 1.11 | 0.337 | 0.717 | 1.000 | 546 | tags=60%, list=39%, signal=97% |
| 18 | REACTOME\_DNA\_REPAIR | Details ... | 23 | 0.24 | 1.08 | 0.360 | 0.739 | 1.000 | 546 | tags=61%, list=39%, signal=98% |
| 19 | REACTOME\_METABOLISM\_OF\_WATER\_SOLUBLE\_VITAMINS\_AND\_COFACTORS | Details ... | 17 | 0.26 | 1.05 | 0.385 | 0.774 | 1.000 | 371 | tags=41%, list=26%, signal=55% |
| 20 | REACTOME\_TRANSLATION | Details ... | 20 | 0.23 | 0.97 | 0.491 | 0.918 | 1.000 | 938 | tags=90%, list=66%, signal=265% |
| 21 | REACTOME\_DISEASES\_OF\_METABOLISM |  | 24 | 0.19 | 0.87 | 0.630 | 1.000 | 1.000 | 457 | tags=46%, list=32%, signal=67% |
| 22 | REACTOME\_METABOLISM\_OF\_CARBOHYDRATES |  | 21 | 0.20 | 0.87 | 0.638 | 1.000 | 1.000 | 740 | tags=71%, list=52%, signal=148% |
| 23 | KEGG\_UBIQUITIN\_MEDIATED\_PROTEOLYSIS |  | 20 | 0.20 | 0.86 | 0.640 | 1.000 | 1.000 | 546 | tags=55%, list=39%, signal=88% |
| 24 | REACTOME\_TRANSCRIPTIONAL\_REGULATION\_BY\_TP53 |  | 33 | 0.17 | 0.84 | 0.674 | 1.000 | 1.000 | 387 | tags=36%, list=27%, signal=49% |
| 25 | REACTOME\_DEUBIQUITINATION |  | 27 | 0.18 | 0.82 | 0.691 | 1.000 | 1.000 | 617 | tags=63%, list=44%, signal=110% |
| 26 | REACTOME\_NEDDYLATION |  | 24 | 0.17 | 0.79 | 0.742 | 1.000 | 1.000 | 909 | tags=83%, list=64%, signal=230% |
| 27 | REACTOME\_CHROMATIN\_MODIFYING\_ENZYMES |  | 31 | 0.16 | 0.79 | 0.742 | 1.000 | 1.000 | 468 | tags=42%, list=33%, signal=61% |
| 28 | REACTOME\_CELLULAR\_RESPONSES\_TO\_EXTERNAL\_STIMULI |  | 49 | 0.13 | 0.73 | 0.814 | 1.000 | 1.000 | 324 | tags=29%, list=23%, signal=36% |
| 29 | KEGG\_P53\_SIGNALING\_PATHWAY |  | 15 | 0.18 | 0.70 | 0.831 | 1.000 | 1.000 | 324 | tags=33%, list=23%, signal=43% |
| 30 | REACTOME\_MITOCHONDRIAL\_BIOGENESIS |  | 15 | 0.18 | 0.69 | 0.832 | 1.000 | 1.000 | 531 | tags=47%, list=38%, signal=74% |
| 31 | REACTOME\_TRANSCRIPTIONAL\_REGULATION\_BY\_RUNX1 |  | 16 | 0.17 | 0.66 | 0.863 | 1.000 | 1.000 | 387 | tags=38%, list=27%, signal=51% |
| 32 | KEGG\_TIGHT\_JUNCTION |  | 15 | 0.16 | 0.63 | 0.892 | 1.000 | 1.000 | 436 | tags=40%, list=31%, signal=57% |
| 33 | REACTOME\_M\_PHASE |  | 21 | 0.15 | 0.63 | 0.927 | 1.000 | 1.000 | 810 | tags=76%, list=57%, signal=176% |
| 34 | REACTOME\_TRANSMISSION\_ACROSS\_CHEMICAL\_SYNAPSES |  | 15 | 0.14 | 0.54 | 0.968 | 1.000 | 1.000 | 464 | tags=40%, list=33%, signal=59% |
| 35 | KEGG\_INSULIN\_SIGNALING\_PATHWAY |  | 19 | 0.13 | 0.53 | 0.967 | 1.000 | 1.000 | 467 | tags=37%, list=33%, signal=54% |
| 36 | REACTOME\_SUMOYLATION |  | 19 | 0.12 | 0.52 | 0.971 | 0.998 | 1.000 | 772 | tags=68%, list=55%, signal=149% |
| 37 | REACTOME\_AUTOPHAGY |  | 15 | 0.12 | 0.46 | 0.995 | 0.991 | 1.000 | 1243 | tags=100%, list=88%, signal=831% |
Table: Gene sets enriched in phenotype **WT (3 samples)**[plain text format]****

  
