# Supplementary material for: Role and mechanism of NCAPD3 in promoting malignant behaviors in gastric cancer
Source: Front Pharmacol. 2024 Apr 22;15:1341039. doi: 10.3389/fphar.2024.1341039 (PMC11070777; doi:10.3389/fphar.2024.1341039)
Supplement: Supplementary file 11 [file DataSheet2.ZIP › GSEA/Canonical pathways/my_analysis.Gsea.1599462267220/heat_map_corr_plot.html]

Heat map and correlation plot for filtered\_dataset  

Fig 1: heat\_map      
 Heat Map of the top 50 features for each phenotype in filtered\_dataset

  
  

Fig 2: Ranked Gene List Correlation Profile      
 Ranked list correlations for filtered\_dataset

  
  
    
