# Supplementary material for: Role and mechanism of NCAPD3 in promoting malignant behaviors in gastric cancer
Source: Front Pharmacol. 2024 Apr 22;15:1341039. doi: 10.3389/fphar.2024.1341039 (PMC11070777; doi:10.3389/fphar.2024.1341039)
Supplement: Supplementary file 11 [file DataSheet2.ZIP › GSEA/Canonical pathways/my_analysis.Gsea.1599462267220/KEGG_CYTOKINE_CYTOKINE_RECEPTOR_INTERACTION.html]

Details for gene set KEGG\_CYTOKINE\_CYTOKINE\_RECEPTOR\_INTERACTION[GSEA]

|  || Dataset | filtered\_dataset.sample\_info.cls#WT\_versus\_NCAPD3\_MUT |
| Phenotype | sample\_info.cls#WT\_versus\_NCAPD3\_MUT |
| Upregulated in class | NCAPD3\_MUT |
| GeneSet | KEGG\_CYTOKINE\_CYTOKINE\_RECEPTOR\_INTERACTION |
| Enrichment Score (ES) | -0.4673852 |
| Normalized Enrichment Score (NES) | -1.9822166 |
| Nominal p-value | 0.0023696683 |
| FDR q-value | 0.024106912 |
| FWER p-Value | 0.121 |
Table: GSEA Results Summary

  

Fig 1: Enrichment plot: KEGG\_CYTOKINE\_CYTOKINE\_RECEPTOR\_INTERACTION      
 Profile of the Running ES Score & Positions of GeneSet Members on the Rank Ordered List

  

| SYMBOL | TITLE | RANK IN GENE LIST | RANK METRIC SCORE | RUNNING ES | CORE ENRICHMENT || 1 | 659 | BMPR2 | 16 | 1.047 | 0.0823 | No |
| 2 | 2921 | CXCL3 | 57 | 0.884 | 0.1327 | No |
| 3 | 2919 | CXCL1 | 405 | 0.534 | -0.0682 | No |
| 4 | 2920 | CXCL2 | 454 | 0.501 | -0.0577 | No |
| 5 | 9180 | OSMR | 981 | -0.394 | -0.3995 | No |
| 6 | 7132 | TNFRSF1A | 1055 | -0.449 | -0.4116 | No |
| 7 | 3556 | IL1RAP | 1099 | -0.476 | -0.3998 | No |
| 8 | 5154 | PDGFA | 1191 | -0.566 | -0.4143 | Yes |
| 9 | 1956 | EGFR | 1266 | -0.657 | -0.4085 | Yes |
| 10 | 8744 | TNFSF9 | 1305 | -0.708 | -0.3724 | Yes |
| 11 | 10344 | CCL26 | 1310 | -0.714 | -0.3114 | Yes |
| 12 | 7422 | VEGFA | 1346 | -0.788 | -0.2659 | Yes |
| 13 | 53833 | IL20RB | 1352 | -0.796 | -0.1982 | Yes |
| 14 | 4233 | MET | 1358 | -0.821 | -0.1282 | Yes |
| 15 | 9966 | TNFSF15 | 1376 | -0.900 | -0.0598 | Yes |
| 16 | 5156 | PDGFRA | 1383 | -0.932 | 0.0194 | Yes |
Table: GSEA details [plain text format]

  

Fig 2: KEGG\_CYTOKINE\_CYTOKINE\_RECEPTOR\_INTERACTION      
 Blue-Pink O' Gram in the Space of the Analyzed GeneSet

  

Fig 3: KEGG\_CYTOKINE\_CYTOKINE\_RECEPTOR\_INTERACTION: Random ES distribution      
 Gene set null distribution of ES for **KEGG\_CYTOKINE\_CYTOKINE\_RECEPTOR\_INTERACTION**

  
