# Supplementary material for: Role and mechanism of NCAPD3 in promoting malignant behaviors in gastric cancer
Source: Front Pharmacol. 2024 Apr 22;15:1341039. doi: 10.3389/fphar.2024.1341039 (PMC11070777; doi:10.3389/fphar.2024.1341039)
Supplement: Supplementary file 11 [file DataSheet2.ZIP › GSEA/Canonical pathways/my_analysis.Gsea.1599462267220/KEGG_FOCAL_ADHESION.html]

Details for gene set KEGG\_FOCAL\_ADHESION[GSEA]

|  || Dataset | filtered\_dataset.sample\_info.cls#WT\_versus\_NCAPD3\_MUT |
| Phenotype | sample\_info.cls#WT\_versus\_NCAPD3\_MUT |
| Upregulated in class | NCAPD3\_MUT |
| GeneSet | KEGG\_FOCAL\_ADHESION |
| Enrichment Score (ES) | -0.40296257 |
| Normalized Enrichment Score (NES) | -1.9129444 |
| Nominal p-value | 0.0024038462 |
| FDR q-value | 0.031828754 |
| FWER p-Value | 0.197 |
Table: GSEA Results Summary

  

Fig 1: Enrichment plot: KEGG\_FOCAL\_ADHESION      
 Profile of the Running ES Score & Positions of GeneSet Members on the Rank Ordered List

  

| SYMBOL | TITLE | RANK IN GENE LIST | RANK METRIC SCORE | RUNNING ES | CORE ENRICHMENT || 1 | 5728 | PTEN | 285 | 0.606 | -0.1529 | No |
| 2 | 5295 | PIK3R1 | 467 | 0.493 | -0.2406 | No |
| 3 | 1793 | DOCK1 | 489 | 0.480 | -0.2143 | No |
| 4 | 3479 | IGF1 | 511 | 0.469 | -0.1889 | No |
| 5 | 2932 | GSK3B | 714 | 0.367 | -0.3027 | No |
| 6 | 2909 | ARHGAP35 | 844 | 0.259 | -0.3732 | Yes |
| 7 | 3673 | ITGA2 | 858 | -0.264 | -0.3598 | Yes |
| 8 | 3913 | LAMB2 | 919 | -0.348 | -0.3729 | Yes |
| 9 | 3691 | ITGB4 | 926 | -0.352 | -0.3468 | Yes |
| 10 | 2317 | FLNB | 936 | -0.360 | -0.3222 | Yes |
| 11 | 3914 | LAMB3 | 1015 | -0.414 | -0.3426 | Yes |
| 12 | 3918 | LAMC2 | 1088 | -0.466 | -0.3542 | Yes |
| 13 | 857 | CAV1 | 1098 | -0.475 | -0.3197 | Yes |
| 14 | 1282 | COL4A1 | 1165 | -0.534 | -0.3211 | Yes |
| 15 | 5154 | PDGFA | 1191 | -0.566 | -0.2902 | Yes |
| 16 | 330 | BIRC3 | 1224 | -0.600 | -0.2615 | Yes |
| 17 | 7057 | THBS1 | 1242 | -0.620 | -0.2202 | Yes |
| 18 | 1956 | EGFR | 1266 | -0.657 | -0.1800 | Yes |
| 19 | 7414 | VCL | 1308 | -0.712 | -0.1481 | Yes |
| 20 | 7422 | VEGFA | 1346 | -0.788 | -0.1067 | Yes |
| 21 | 4233 | MET | 1358 | -0.821 | -0.0438 | Yes |
| 22 | 5156 | PDGFRA | 1383 | -0.932 | 0.0194 | Yes |
Table: GSEA details [plain text format]

  

Fig 2: KEGG\_FOCAL\_ADHESION      
 Blue-Pink O' Gram in the Space of the Analyzed GeneSet

  

Fig 3: KEGG\_FOCAL\_ADHESION: Random ES distribution      
 Gene set null distribution of ES for **KEGG\_FOCAL\_ADHESION**

  
