# Supplementary material for: Role and mechanism of NCAPD3 in promoting malignant behaviors in gastric cancer
Source: Front Pharmacol. 2024 Apr 22;15:1341039. doi: 10.3389/fphar.2024.1341039 (PMC11070777; doi:10.3389/fphar.2024.1341039)
Supplement: Supplementary file 11 [file DataSheet2.ZIP › GSEA/Canonical pathways/my_analysis.Gsea.1599462267220/KEGG_MAPK_SIGNALING_PATHWAY.html]

Details for gene set KEGG\_MAPK\_SIGNALING\_PATHWAY[GSEA]

|  || Dataset | filtered\_dataset.sample\_info.cls#WT\_versus\_NCAPD3\_MUT |
| Phenotype | sample\_info.cls#WT\_versus\_NCAPD3\_MUT |
| Upregulated in class | NCAPD3\_MUT |
| GeneSet | KEGG\_MAPK\_SIGNALING\_PATHWAY |
| Enrichment Score (ES) | -0.33699316 |
| Normalized Enrichment Score (NES) | -1.6319553 |
| Nominal p-value | 0.04221636 |
| FDR q-value | 0.10925946 |
| FWER p-Value | 0.7 |
Table: GSEA Results Summary

  

Fig 1: Enrichment plot: KEGG\_MAPK\_SIGNALING\_PATHWAY      
 Profile of the Running ES Score & Positions of GeneSet Members on the Rank Ordered List

  

| SYMBOL | TITLE | RANK IN GENE LIST | RANK METRIC SCORE | RUNNING ES | CORE ENRICHMENT || 1 | 3303 | HSPA1A | 206 | 0.669 | -0.0872 | No |
| 2 | 3304 | HSPA1B | 207 | 0.669 | -0.0260 | No |
| 3 | 5567 | PRKACB | 595 | 0.426 | -0.2658 | No |
| 4 | 80824 | DUSP16 | 610 | 0.418 | -0.2377 | No |
| 5 | 6197 | RPS6KA3 | 643 | 0.403 | -0.2239 | No |
| 6 | 5613 | PRKX | 722 | 0.363 | -0.2469 | No |
| 7 | 3312 | HSPA8 | 848 | 0.244 | -0.3147 | Yes |
| 8 | 2872 | MKNK2 | 860 | -0.269 | -0.2980 | Yes |
| 9 | 6237 | RRAS | 869 | -0.280 | -0.2782 | Yes |
| 10 | 59285 | CACNG6 | 893 | -0.310 | -0.2664 | Yes |
| 11 | 4616 | GADD45B | 904 | -0.330 | -0.2434 | Yes |
| 12 | 3164 | NR4A1 | 912 | -0.341 | -0.2173 | Yes |
| 13 | 6789 | STK4 | 915 | -0.345 | -0.1872 | Yes |
| 14 | 2317 | FLNB | 936 | -0.360 | -0.1687 | Yes |
| 15 | 7132 | TNFRSF1A | 1055 | -0.449 | -0.2126 | Yes |
| 16 | 4773 | NFATC2 | 1085 | -0.465 | -0.1909 | Yes |
| 17 | 11221 | DUSP10 | 1095 | -0.475 | -0.1540 | Yes |
| 18 | 5154 | PDGFA | 1191 | -0.566 | -0.1707 | Yes |
| 19 | 51776 | ZAK | 1196 | -0.571 | -0.1213 | Yes |
| 20 | 2005 | ELK4 | 1235 | -0.612 | -0.0927 | Yes |
| 21 | 1956 | EGFR | 1266 | -0.657 | -0.0542 | Yes |
| 22 | 1649 | DDIT3 | 1341 | -0.779 | -0.0363 | Yes |
| 23 | 5156 | PDGFRA | 1383 | -0.932 | 0.0195 | Yes |
Table: GSEA details [plain text format]

  

Fig 2: KEGG\_MAPK\_SIGNALING\_PATHWAY      
 Blue-Pink O' Gram in the Space of the Analyzed GeneSet

  

Fig 3: KEGG\_MAPK\_SIGNALING\_PATHWAY: Random ES distribution      
 Gene set null distribution of ES for **KEGG\_MAPK\_SIGNALING\_PATHWAY**

  
