# Supplementary material for: Role and mechanism of NCAPD3 in promoting malignant behaviors in gastric cancer
Source: Front Pharmacol. 2024 Apr 22;15:1341039. doi: 10.3389/fphar.2024.1341039 (PMC11070777; doi:10.3389/fphar.2024.1341039)
Supplement: Supplementary file 11 [file DataSheet2.ZIP › GSEA/Canonical pathways/my_analysis.Gsea.1599462267220/KEGG_REGULATION_OF_ACTIN_CYTOSKELETON.html]

Details for gene set KEGG\_REGULATION\_OF\_ACTIN\_CYTOSKELETON[GSEA]

|  || Dataset | filtered\_dataset.sample\_info.cls#WT\_versus\_NCAPD3\_MUT |
| Phenotype | sample\_info.cls#WT\_versus\_NCAPD3\_MUT |
| Upregulated in class | NCAPD3\_MUT |
| GeneSet | KEGG\_REGULATION\_OF\_ACTIN\_CYTOSKELETON |
| Enrichment Score (ES) | -0.3640096 |
| Normalized Enrichment Score (NES) | -1.5450261 |
| Nominal p-value | 0.052757792 |
| FDR q-value | 0.15326862 |
| FWER p-Value | 0.848 |
Table: GSEA Results Summary

  

Fig 1: Enrichment plot: KEGG\_REGULATION\_OF\_ACTIN\_CYTOSKELETON      
 Profile of the Running ES Score & Positions of GeneSet Members on the Rank Ordered List

  

| SYMBOL | TITLE | RANK IN GENE LIST | RANK METRIC SCORE | RUNNING ES | CORE ENRICHMENT || 1 | 54434 | SSH1 | 183 | 0.692 | -0.0507 | No |
| 2 | 324 | APC | 281 | 0.607 | -0.0495 | No |
| 3 | 5295 | PIK3R1 | 467 | 0.493 | -0.1248 | No |
| 4 | 1793 | DOCK1 | 489 | 0.480 | -0.0840 | No |
| 5 | 2909 | ARHGAP35 | 844 | 0.259 | -0.3077 | Yes |
| 6 | 3673 | ITGA2 | 858 | -0.264 | -0.2863 | Yes |
| 7 | 6237 | RRAS | 869 | -0.280 | -0.2609 | Yes |
| 8 | 3691 | ITGB4 | 926 | -0.352 | -0.2601 | Yes |
| 9 | 5305 | PIP4K2A | 1033 | -0.432 | -0.2859 | Yes |
| 10 | 7074 | TIAM1 | 1143 | -0.508 | -0.3049 | Yes |
| 11 | 5154 | PDGFA | 1191 | -0.566 | -0.2727 | Yes |
| 12 | 26999 | CYFIP2 | 1234 | -0.611 | -0.2318 | Yes |
| 13 | 1956 | EGFR | 1266 | -0.657 | -0.1776 | Yes |
| 14 | 7414 | VCL | 1308 | -0.712 | -0.1242 | Yes |
| 15 | 23365 | ARHGEF12 | 1329 | -0.751 | -0.0511 | Yes |
| 16 | 5156 | PDGFRA | 1383 | -0.932 | 0.0194 | Yes |
Table: GSEA details [plain text format]

  

Fig 2: KEGG\_REGULATION\_OF\_ACTIN\_CYTOSKELETON      
 Blue-Pink O' Gram in the Space of the Analyzed GeneSet

  

Fig 3: KEGG\_REGULATION\_OF\_ACTIN\_CYTOSKELETON: Random ES distribution      
 Gene set null distribution of ES for **KEGG\_REGULATION\_OF\_ACTIN\_CYTOSKELETON**

  
