# Supplementary material for: Role and mechanism of NCAPD3 in promoting malignant behaviors in gastric cancer
Source: Front Pharmacol. 2024 Apr 22;15:1341039. doi: 10.3389/fphar.2024.1341039 (PMC11070777; doi:10.3389/fphar.2024.1341039)
Supplement: Supplementary file 11 [file DataSheet2.ZIP › GSEA/Canonical pathways/my_analysis.Gsea.1599462267220/NABA_MATRISOME.html]

Details for gene set NABA\_MATRISOME[GSEA]

|  || Dataset | filtered\_dataset.sample\_info.cls#WT\_versus\_NCAPD3\_MUT |
| Phenotype | sample\_info.cls#WT\_versus\_NCAPD3\_MUT |
| Upregulated in class | NCAPD3\_MUT |
| GeneSet | NABA\_MATRISOME |
| Enrichment Score (ES) | -0.37949854 |
| Normalized Enrichment Score (NES) | -2.342604 |
| Nominal p-value | 0.0 |
| FDR q-value | 0.0064973654 |
| FWER p-Value | 0.009 |
Table: GSEA Results Summary

  

Fig 1: Enrichment plot: NABA\_MATRISOME      
 Profile of the Running ES Score & Positions of GeneSet Members on the Rank Ordered List

  

| SYMBOL | TITLE | RANK IN GENE LIST | RANK METRIC SCORE | RUNNING ES | CORE ENRICHMENT || 1 | 7052 | TGM2 | 17 | 1.045 | 0.0215 | No |
| 2 | 2243 | FGA | 18 | 1.039 | 0.0553 | No |
| 3 | 4811 | NID1 | 51 | 0.913 | 0.0616 | No |
| 4 | 2921 | CXCL3 | 57 | 0.884 | 0.0867 | No |
| 5 | 5967 | REG1A | 100 | 0.788 | 0.0815 | No |
| 6 | 64856 | VWA1 | 259 | 0.623 | -0.0141 | No |
| 7 | 4238 | MFAP3 | 279 | 0.609 | -0.0082 | No |
| 8 | 2244 | FGB | 321 | 0.582 | -0.0194 | No |
| 9 | 2919 | CXCL1 | 405 | 0.534 | -0.0629 | No |
| 10 | 2920 | CXCL2 | 454 | 0.501 | -0.0818 | No |
| 11 | 1755 | DMBT1 | 480 | 0.487 | -0.0843 | No |
| 12 | 3479 | IGF1 | 511 | 0.469 | -0.0910 | No |
| 13 | 9037 | SEMA5A | 688 | 0.385 | -0.2076 | No |
| 14 | 6385 | SDC4 | 871 | -0.282 | -0.3320 | No |
| 15 | 301 | ANXA1 | 914 | -0.342 | -0.3517 | No |
| 16 | 3913 | LAMB2 | 919 | -0.348 | -0.3432 | No |
| 17 | 4052 | LTBP1 | 928 | -0.353 | -0.3376 | No |
| 18 | 3489 | IGFBP6 | 934 | -0.359 | -0.3296 | No |
| 19 | 6273 | S100A2 | 942 | -0.365 | -0.3229 | No |
| 20 | 6286 | S100P | 1011 | -0.412 | -0.3594 | No |
| 21 | 3914 | LAMB3 | 1015 | -0.414 | -0.3481 | No |
| 22 | 83998 | REG4 | 1037 | -0.435 | -0.3493 | No |
| 23 | 6590 | SLPI | 1062 | -0.453 | -0.3522 | No |
| 24 | 1308 | COL17A1 | 1065 | -0.455 | -0.3389 | No |
| 25 | 3918 | LAMC2 | 1088 | -0.466 | -0.3398 | No |
| 26 | 5268 | SERPINB5 | 1089 | -0.467 | -0.3246 | No |
| 27 | 3963 | LGALS7 | 1107 | -0.483 | -0.3214 | No |
| 28 | 1282 | COL4A1 | 1165 | -0.534 | -0.3458 | No |
| 29 | 5154 | PDGFA | 1191 | -0.566 | -0.3457 | No |
| 30 | 145864 | HAPLN3 | 1238 | -0.616 | -0.3595 | Yes |
| 31 | 7057 | THBS1 | 1242 | -0.620 | -0.3415 | Yes |
| 32 | 50856 | CLEC4A | 1249 | -0.630 | -0.3254 | Yes |
| 33 | 1893 | ECM1 | 1250 | -0.637 | -0.3046 | Yes |
| 34 | 23436 | CELA3B | 1273 | -0.661 | -0.2993 | Yes |
| 35 | 10136 | CELA3A | 1274 | -0.661 | -0.2777 | Yes |
| 36 | 4240 | MFGE8 | 1280 | -0.668 | -0.2596 | Yes |
| 37 | 5046 | PCSK6 | 1297 | -0.699 | -0.2487 | Yes |
| 38 | 8744 | TNFSF9 | 1305 | -0.708 | -0.2308 | Yes |
| 39 | 10344 | CCL26 | 1310 | -0.714 | -0.2105 | Yes |
| 40 | 3084 | NRG1 | 1320 | -0.739 | -0.1930 | Yes |
| 41 | 1839 | HBEGF | 1328 | -0.751 | -0.1737 | Yes |
| 42 | 113791 | PIK3IP1 | 1345 | -0.787 | -0.1599 | Yes |
| 43 | 7422 | VEGFA | 1346 | -0.788 | -0.1342 | Yes |
| 44 | 9966 | TNFSF15 | 1376 | -0.900 | -0.1262 | Yes |
| 45 | 59277 | NTN4 | 1390 | -0.999 | -0.1032 | Yes |
| 46 | 5055 | SERPINB2 | 1397 | -1.088 | -0.0722 | Yes |
| 47 | 1475 | CSTA | 1400 | -1.162 | -0.0358 | Yes |
| 48 | 4312 | MMP1 | 1407 | -1.304 | 0.0022 | Yes |
Table: GSEA details [plain text format]

  

Fig 2: NABA\_MATRISOME      
 Blue-Pink O' Gram in the Space of the Analyzed GeneSet

  

Fig 3: NABA\_MATRISOME: Random ES distribution      
 Gene set null distribution of ES for **NABA\_MATRISOME**

  
