# Supplementary material for: Role and mechanism of NCAPD3 in promoting malignant behaviors in gastric cancer
Source: Front Pharmacol. 2024 Apr 22;15:1341039. doi: 10.3389/fphar.2024.1341039 (PMC11070777; doi:10.3389/fphar.2024.1341039)
Supplement: Supplementary file 11 [file DataSheet2.ZIP › GSEA/Canonical pathways/my_analysis.Gsea.1599462267220/PID_P53_DOWNSTREAM_PATHWAY.html]

Details for gene set PID\_P53\_DOWNSTREAM\_PATHWAY[GSEA]

|  || Dataset | filtered\_dataset.sample\_info.cls#WT\_versus\_NCAPD3\_MUT |
| Phenotype | sample\_info.cls#WT\_versus\_NCAPD3\_MUT |
| Upregulated in class | NCAPD3\_MUT |
| GeneSet | PID\_P53\_DOWNSTREAM\_PATHWAY |
| Enrichment Score (ES) | -0.3673813 |
| Normalized Enrichment Score (NES) | -1.7674958 |
| Nominal p-value | 0.019900497 |
| FDR q-value | 0.06530372 |
| FWER p-Value | 0.409 |
Table: GSEA Results Summary

  

Fig 1: Enrichment plot: PID\_P53\_DOWNSTREAM\_PATHWAY      
 Profile of the Running ES Score & Positions of GeneSet Members on the Rank Ordered List

  

| SYMBOL | TITLE | RANK IN GENE LIST | RANK METRIC SCORE | RUNNING ES | CORE ENRICHMENT || 1 | 57103 | TIGAR | 37 | 0.963 | 0.0452 | No |
| 2 | 4193 | MDM2 | 131 | 0.769 | 0.0356 | No |
| 3 | 55240 | STEAP3 | 169 | 0.710 | 0.0620 | No |
| 4 | 324 | APC | 281 | 0.607 | 0.0274 | No |
| 5 | 5728 | PTEN | 285 | 0.606 | 0.0704 | No |
| 6 | 1387 | CREBBP | 387 | 0.544 | 0.0383 | No |
| 7 | 637 | BID | 478 | 0.487 | 0.0098 | No |
| 8 | 839 | CASP6 | 792 | 0.330 | -0.1909 | No |
| 9 | 6273 | S100A2 | 942 | -0.365 | -0.2709 | No |
| 10 | 5111 | PCNA | 1077 | -0.460 | -0.3331 | Yes |
| 11 | 5268 | SERPINB5 | 1089 | -0.467 | -0.3061 | Yes |
| 12 | 857 | CAV1 | 1098 | -0.475 | -0.2764 | Yes |
| 13 | 54541 | DDIT4 | 1133 | -0.502 | -0.2634 | Yes |
| 14 | 1263 | PLK3 | 1134 | -0.502 | -0.2260 | Yes |
| 15 | 604 | BCL6 | 1136 | -0.504 | -0.1890 | Yes |
| 16 | 639 | PRDM1 | 1159 | -0.523 | -0.1658 | Yes |
| 17 | 665 | BNIP3L | 1185 | -0.556 | -0.1424 | Yes |
| 18 | 1956 | EGFR | 1266 | -0.657 | -0.1509 | Yes |
| 19 | 133746 | JMY | 1350 | -0.793 | -0.1515 | Yes |
| 20 | 4233 | MET | 1358 | -0.821 | -0.0953 | Yes |
| 21 | 255488 | RNF144B | 1362 | -0.834 | -0.0351 | Yes |
| 22 | 10397 | NDRG1 | 1380 | -0.925 | 0.0216 | Yes |
Table: GSEA details [plain text format]

  

Fig 2: PID\_P53\_DOWNSTREAM\_PATHWAY      
 Blue-Pink O' Gram in the Space of the Analyzed GeneSet

  

Fig 3: PID\_P53\_DOWNSTREAM\_PATHWAY: Random ES distribution      
 Gene set null distribution of ES for **PID\_P53\_DOWNSTREAM\_PATHWAY**

  
