# Supplementary material for: Role and mechanism of NCAPD3 in promoting malignant behaviors in gastric cancer
Source: Front Pharmacol. 2024 Apr 22;15:1341039. doi: 10.3389/fphar.2024.1341039 (PMC11070777; doi:10.3389/fphar.2024.1341039)
Supplement: Supplementary file 11 [file DataSheet2.ZIP › GSEA/Canonical pathways/my_analysis.Gsea.1599462267220/REACTOME_CELL_CYCLE_CHECKPOINTS.html]

Details for gene set REACTOME\_CELL\_CYCLE\_CHECKPOINTS[GSEA]

|  || Dataset | filtered\_dataset.sample\_info.cls#WT\_versus\_NCAPD3\_MUT |
| Phenotype | sample\_info.cls#WT\_versus\_NCAPD3\_MUT |
| Upregulated in class | WT |
| GeneSet | REACTOME\_CELL\_CYCLE\_CHECKPOINTS |
| Enrichment Score (ES) | 0.2972653 |
| Normalized Enrichment Score (NES) | 1.1789604 |
| Nominal p-value | 0.265 |
| FDR q-value | 0.79957163 |
| FWER p-Value | 1.0 |
Table: GSEA Results Summary

  

Fig 1: Enrichment plot: REACTOME\_CELL\_CYCLE\_CHECKPOINTS      
 Profile of the Running ES Score & Positions of GeneSet Members on the Rank Ordered List

  

| SYMBOL | TITLE | RANK IN GENE LIST | RANK METRIC SCORE | RUNNING ES | CORE ENRICHMENT || 1 | 51512 | GTSE1 | 127 | 0.774 | -0.0085 | Yes |
| 2 | 4193 | MDM2 | 131 | 0.769 | 0.0715 | Yes |
| 3 | 51434 | ANAPC7 | 166 | 0.710 | 0.1229 | Yes |
| 4 | 84142 | FAM175A | 211 | 0.667 | 0.1626 | Yes |
| 5 | 5701 | PSMC2 | 244 | 0.636 | 0.2075 | Yes |
| 6 | 79184 | BRCC3 | 348 | 0.569 | 0.1944 | Yes |
| 7 | 5810 | RAD1 | 360 | 0.560 | 0.2462 | Yes |
| 8 | 5718 | PSMD12 | 372 | 0.552 | 0.2973 | Yes |
| 9 | 5001 | ORC5 | 450 | 0.506 | 0.2960 | No |
| 10 | 7334 | UBE2N | 546 | 0.449 | 0.2758 | No |
| 11 | 10726 | NUDC | 754 | 0.346 | 0.1643 | No |
| 12 | 990 | CDC6 | 888 | -0.304 | 0.1013 | No |
| 13 | 8881 | CDC16 | 963 | -0.383 | 0.0891 | No |
| 14 | 4194 | MDM4 | 1117 | -0.492 | 0.0319 | No |
| 15 | 7468 | WHSC1 | 1122 | -0.496 | 0.0820 | No |
| 16 | 472 | ATM | 1193 | -0.568 | 0.0925 | No |
| 17 | 8345 | HIST1H2BH | 1207 | -0.585 | 0.1456 | No |
Table: GSEA details [plain text format]

  

Fig 2: REACTOME\_CELL\_CYCLE\_CHECKPOINTS      
 Blue-Pink O' Gram in the Space of the Analyzed GeneSet

  

Fig 3: REACTOME\_CELL\_CYCLE\_CHECKPOINTS: Random ES distribution      
 Gene set null distribution of ES for **REACTOME\_CELL\_CYCLE\_CHECKPOINTS**

  
