# Supplementary material for: Role and mechanism of NCAPD3 in promoting malignant behaviors in gastric cancer
Source: Front Pharmacol. 2024 Apr 22;15:1341039. doi: 10.3389/fphar.2024.1341039 (PMC11070777; doi:10.3389/fphar.2024.1341039)
Supplement: Supplementary file 11 [file DataSheet2.ZIP › GSEA/Canonical pathways/my_analysis.Gsea.1599462267220/REACTOME_CYTOKINE_SIGNALING_IN_IMMUNE_SYSTEM.html]

Details for gene set REACTOME\_CYTOKINE\_SIGNALING\_IN\_IMMUNE\_SYSTEM[GSEA]

|  || Dataset | filtered\_dataset.sample\_info.cls#WT\_versus\_NCAPD3\_MUT |
| Phenotype | sample\_info.cls#WT\_versus\_NCAPD3\_MUT |
| Upregulated in class | NCAPD3\_MUT |
| GeneSet | REACTOME\_CYTOKINE\_SIGNALING\_IN\_IMMUNE\_SYSTEM |
| Enrichment Score (ES) | -0.27413562 |
| Normalized Enrichment Score (NES) | -2.0145838 |
| Nominal p-value | 0.0 |
| FDR q-value | 0.02084979 |
| FWER p-Value | 0.092 |
Table: GSEA Results Summary

  

Fig 1: Enrichment plot: REACTOME\_CYTOKINE\_SIGNALING\_IN\_IMMUNE\_SYSTEM      
 Profile of the Running ES Score & Positions of GeneSet Members on the Rank Ordered List

  

| SYMBOL | TITLE | RANK IN GENE LIST | RANK METRIC SCORE | RUNNING ES | CORE ENRICHMENT || 1 | 2243 | FGA | 18 | 1.039 | 0.0092 | No |
| 2 | 117145 | THEM4 | 65 | 0.860 | -0.0066 | No |
| 3 | 3654 | IRAK1 | 69 | 0.856 | 0.0099 | No |
| 4 | 3667 | IRS1 | 83 | 0.821 | 0.0180 | No |
| 5 | 817 | CAMK2D | 165 | 0.710 | -0.0272 | No |
| 6 | 6672 | SP100 | 185 | 0.689 | -0.0265 | No |
| 7 | 8831 | SYNGAP1 | 236 | 0.647 | -0.0499 | No |
| 8 | 5701 | PSMC2 | 244 | 0.636 | -0.0412 | No |
| 9 | 51135 | IRAK4 | 245 | 0.635 | -0.0274 | No |
| 10 | 6850 | SYK | 256 | 0.625 | -0.0212 | No |
| 11 | 4281 | MID1 | 294 | 0.595 | -0.0360 | No |
| 12 | 84078 | KBTBD7 | 295 | 0.594 | -0.0230 | No |
| 13 | 2244 | FGB | 321 | 0.582 | -0.0291 | No |
| 14 | 960 | CD44 | 339 | 0.573 | -0.0293 | No |
| 15 | 10818 | FRS2 | 363 | 0.558 | -0.0344 | No |
| 16 | 5718 | PSMD12 | 372 | 0.552 | -0.0284 | No |
| 17 | 2919 | CXCL1 | 405 | 0.534 | -0.0407 | No |
| 18 | 801 | CALM1 | 424 | 0.518 | -0.0429 | No |
| 19 | 2920 | CXCL2 | 454 | 0.501 | -0.0537 | No |
| 20 | 5295 | PIK3R1 | 467 | 0.493 | -0.0520 | No |
| 21 | 5245 | PHB | 481 | 0.486 | -0.0511 | No |
| 22 | 9830 | TRIM14 | 486 | 0.480 | -0.0436 | No |
| 23 | 8454 | CUL1 | 492 | 0.477 | -0.0369 | No |
| 24 | 7334 | UBE2N | 546 | 0.449 | -0.0669 | No |
| 25 | 3836 | KPNA1 | 548 | 0.448 | -0.0579 | No |
| 26 | 23586 | DDX58 | 584 | 0.433 | -0.0747 | No |
| 27 | 3433 | IFIT2 | 608 | 0.418 | -0.0828 | No |
| 28 | 80824 | DUSP16 | 610 | 0.418 | -0.0745 | No |
| 29 | 8021 | NUP214 | 611 | 0.417 | -0.0653 | No |
| 30 | 6197 | RPS6KA3 | 643 | 0.403 | -0.0798 | No |
| 31 | 4507 | MTAP | 690 | 0.384 | -0.1060 | No |
| 32 | 3434 | IFIT1 | 743 | 0.354 | -0.1373 | No |
| 33 | 1973 | EIF4A1 | 804 | 0.319 | -0.1753 | No |
| 34 | 5898 | RALA | 813 | 0.309 | -0.1746 | No |
| 35 | 3146 | HMGB1 | 831 | 0.295 | -0.1809 | No |
| 36 | 8835 | SOCS2 | 840 | 0.275 | -0.1809 | No |
| 37 | 3312 | HSPA8 | 848 | 0.244 | -0.1808 | No |
| 38 | 3106 | HLA-B | 855 | -0.258 | -0.1797 | No |
| 39 | 4502 | MT2A | 863 | -0.277 | -0.1789 | No |
| 40 | 5292 | PIM1 | 885 | -0.300 | -0.1881 | No |
| 41 | 3664 | IRF6 | 908 | -0.334 | -0.1973 | No |
| 42 | 301 | ANXA1 | 914 | -0.342 | -0.1936 | No |
| 43 | 4088 | SMAD3 | 930 | -0.353 | -0.1972 | No |
| 44 | 2317 | FLNB | 936 | -0.360 | -0.1930 | No |
| 45 | 3716 | JAK1 | 939 | -0.363 | -0.1866 | No |
| 46 | 3665 | IRF7 | 956 | -0.375 | -0.1905 | No |
| 47 | 9180 | OSMR | 981 | -0.394 | -0.1999 | No |
| 48 | 7132 | TNFRSF1A | 1055 | -0.449 | -0.2449 | No |
| 49 | 11221 | DUSP10 | 1095 | -0.475 | -0.2638 | Yes |
| 50 | 3669 | ISG20 | 1097 | -0.475 | -0.2541 | Yes |
| 51 | 3556 | IL1RAP | 1099 | -0.476 | -0.2445 | Yes |
| 52 | 317649 | EIF4E3 | 1102 | -0.479 | -0.2355 | Yes |
| 53 | 6709 | SPTAN1 | 1114 | -0.487 | -0.2331 | Yes |
| 54 | 604 | BCL6 | 1136 | -0.504 | -0.2379 | Yes |
| 55 | 3134 | HLA-F | 1139 | -0.506 | -0.2283 | Yes |
| 56 | 27250 | PDCD4 | 1170 | -0.537 | -0.2391 | Yes |
| 57 | 58480 | RHOU | 1189 | -0.560 | -0.2404 | Yes |
| 58 | 5154 | PDGFA | 1191 | -0.566 | -0.2288 | Yes |
| 59 | 330 | BIRC3 | 1224 | -0.600 | -0.2397 | Yes |
| 60 | 1956 | EGFR | 1266 | -0.657 | -0.2561 | Yes |
| 61 | 8651 | SOCS1 | 1272 | -0.661 | -0.2454 | Yes |
| 62 | 57761 | TRIB3 | 1292 | -0.685 | -0.2447 | Yes |
| 63 | 8744 | TNFSF9 | 1305 | -0.708 | -0.2383 | Yes |
| 64 | 7414 | VCL | 1308 | -0.712 | -0.2242 | Yes |
| 65 | 3084 | NRG1 | 1320 | -0.739 | -0.2164 | Yes |
| 66 | 90865 | IL33 | 1325 | -0.745 | -0.2031 | Yes |
| 67 | 1839 | HBEGF | 1328 | -0.751 | -0.1882 | Yes |
| 68 | 7422 | VEGFA | 1346 | -0.788 | -0.1837 | Yes |
| 69 | 53833 | IL20RB | 1352 | -0.796 | -0.1701 | Yes |
| 70 | 7706 | TRIM25 | 1357 | -0.817 | -0.1552 | Yes |
| 71 | 4233 | MET | 1358 | -0.821 | -0.1373 | Yes |
| 72 | 5784 | PTPN14 | 1363 | -0.840 | -0.1220 | Yes |
| 73 | 4734 | NEDD4 | 1369 | -0.856 | -0.1070 | Yes |
| 74 | 9966 | TNFSF15 | 1376 | -0.900 | -0.0918 | Yes |
| 75 | 6711 | SPTBN1 | 1381 | -0.925 | -0.0746 | Yes |
| 76 | 5156 | PDGFRA | 1383 | -0.932 | -0.0550 | Yes |
| 77 | 23650 | TRIM29 | 1384 | -0.951 | -0.0342 | Yes |
| 78 | 5055 | SERPINB2 | 1397 | -1.088 | -0.0195 | Yes |
| 79 | 4312 | MMP1 | 1407 | -1.304 | 0.0023 | Yes |
Table: GSEA details [plain text format]

  

Fig 2: REACTOME\_CYTOKINE\_SIGNALING\_IN\_IMMUNE\_SYSTEM      
 Blue-Pink O' Gram in the Space of the Analyzed GeneSet

  

Fig 3: REACTOME\_CYTOKINE\_SIGNALING\_IN\_IMMUNE\_SYSTEM: Random ES distribution      
 Gene set null distribution of ES for **REACTOME\_CYTOKINE\_SIGNALING\_IN\_IMMUNE\_SYSTEM**

  
