# Supplementary material for: Role and mechanism of NCAPD3 in promoting malignant behaviors in gastric cancer
Source: Front Pharmacol. 2024 Apr 22;15:1341039. doi: 10.3389/fphar.2024.1341039 (PMC11070777; doi:10.3389/fphar.2024.1341039)
Supplement: Supplementary file 11 [file DataSheet2.ZIP › GSEA/Canonical pathways/my_analysis.Gsea.1599462267220/REACTOME_DEATH_RECEPTOR_SIGNALLING.html]

Details for gene set REACTOME\_DEATH\_RECEPTOR\_SIGNALLING[GSEA]

|  || Dataset | filtered\_dataset.sample\_info.cls#WT\_versus\_NCAPD3\_MUT |
| Phenotype | sample\_info.cls#WT\_versus\_NCAPD3\_MUT |
| Upregulated in class | NCAPD3\_MUT |
| GeneSet | REACTOME\_DEATH\_RECEPTOR\_SIGNALLING |
| Enrichment Score (ES) | -0.35519075 |
| Normalized Enrichment Score (NES) | -1.4683789 |
| Nominal p-value | 0.08254717 |
| FDR q-value | 0.20132229 |
| FWER p-Value | 0.929 |
Table: GSEA Results Summary

  

Fig 1: Enrichment plot: REACTOME\_DEATH\_RECEPTOR\_SIGNALLING      
 Profile of the Running ES Score & Positions of GeneSet Members on the Rank Ordered List

  

| SYMBOL | TITLE | RANK IN GENE LIST | RANK METRIC SCORE | RUNNING ES | CORE ENRICHMENT || 1 | 3654 | IRAK1 | 69 | 0.856 | 0.0625 | No |
| 2 | 23229 | ARHGEF9 | 317 | 0.584 | -0.0380 | No |
| 3 | 8737 | RIPK1 | 493 | 0.477 | -0.1009 | No |
| 4 | 8772 | FADD | 742 | 0.355 | -0.2322 | No |
| 5 | 8837 | CFLAR | 865 | -0.278 | -0.2832 | No |
| 6 | 9181 | ARHGEF2 | 902 | -0.326 | -0.2664 | No |
| 7 | 10018 | BCL2L11 | 1027 | -0.425 | -0.2996 | Yes |
| 8 | 10616 | RBCK1 | 1042 | -0.438 | -0.2523 | Yes |
| 9 | 7132 | TNFRSF1A | 1055 | -0.449 | -0.2022 | Yes |
| 10 | 7204 | TRIO | 1120 | -0.494 | -0.1834 | Yes |
| 11 | 7074 | TIAM1 | 1143 | -0.508 | -0.1326 | Yes |
| 12 | 1540 | CYLD | 1162 | -0.530 | -0.0761 | Yes |
| 13 | 7128 | TNFAIP3 | 1198 | -0.573 | -0.0263 | Yes |
| 14 | 330 | BIRC3 | 1224 | -0.600 | 0.0342 | Yes |
| 15 | 23365 | ARHGEF12 | 1329 | -0.751 | 0.0580 | Yes |
Table: GSEA details [plain text format]

  

Fig 2: REACTOME\_DEATH\_RECEPTOR\_SIGNALLING      
 Blue-Pink O' Gram in the Space of the Analyzed GeneSet

  

Fig 3: REACTOME\_DEATH\_RECEPTOR\_SIGNALLING: Random ES distribution      
 Gene set null distribution of ES for **REACTOME\_DEATH\_RECEPTOR\_SIGNALLING**

  
