# Supplementary material for: Role and mechanism of NCAPD3 in promoting malignant behaviors in gastric cancer
Source: Front Pharmacol. 2024 Apr 22;15:1341039. doi: 10.3389/fphar.2024.1341039 (PMC11070777; doi:10.3389/fphar.2024.1341039)
Supplement: Supplementary file 11 [file DataSheet2.ZIP › GSEA/Canonical pathways/my_analysis.Gsea.1599462267220/REACTOME_DEVELOPMENTAL_BIOLOGY.html]

Details for gene set REACTOME\_DEVELOPMENTAL\_BIOLOGY[GSEA]

|  || Dataset | filtered\_dataset.sample\_info.cls#WT\_versus\_NCAPD3\_MUT |
| Phenotype | sample\_info.cls#WT\_versus\_NCAPD3\_MUT |
| Upregulated in class | NCAPD3\_MUT |
| GeneSet | REACTOME\_DEVELOPMENTAL\_BIOLOGY |
| Enrichment Score (ES) | -0.27560836 |
| Normalized Enrichment Score (NES) | -1.8664271 |
| Nominal p-value | 0.0 |
| FDR q-value | 0.03891414 |
| FWER p-Value | 0.257 |
Table: GSEA Results Summary

  

Fig 1: Enrichment plot: REACTOME\_DEVELOPMENTAL\_BIOLOGY      
 Profile of the Running ES Score & Positions of GeneSet Members on the Rank Ordered List

  

| SYMBOL | TITLE | RANK IN GENE LIST | RANK METRIC SCORE | RUNNING ES | CORE ENRICHMENT || 1 | 2935 | GSPT1 | 5 | 1.156 | 0.0273 | No |
| 2 | 5087 | PBX1 | 54 | 0.908 | 0.0161 | No |
| 3 | 23054 | NCOA6 | 77 | 0.838 | 0.0222 | No |
| 4 | 6925 | TCF4 | 132 | 0.768 | 0.0027 | No |
| 5 | 23380 | SRGAP2 | 142 | 0.757 | 0.0164 | No |
| 6 | 6191 | RPS4X | 196 | 0.682 | -0.0047 | No |
| 7 | 5701 | PSMC2 | 244 | 0.636 | -0.0225 | No |
| 8 | 7220 | TRPC1 | 328 | 0.576 | -0.0688 | No |
| 9 | 4297 | KMT2A | 337 | 0.573 | -0.0593 | No |
| 10 | 10818 | FRS2 | 363 | 0.558 | -0.0629 | No |
| 11 | 5718 | PSMD12 | 372 | 0.552 | -0.0540 | No |
| 12 | 5465 | PPARA | 375 | 0.550 | -0.0407 | No |
| 13 | 1387 | CREBBP | 387 | 0.544 | -0.0343 | No |
| 14 | 6256 | RXRA | 439 | 0.509 | -0.0585 | No |
| 15 | 5295 | PIK3R1 | 467 | 0.493 | -0.0653 | No |
| 16 | 1793 | DOCK1 | 489 | 0.480 | -0.0680 | No |
| 17 | 5567 | PRKACB | 595 | 0.426 | -0.1346 | No |
| 18 | 6208 | RPS14 | 604 | 0.421 | -0.1293 | No |
| 19 | 6197 | RPS6KA3 | 643 | 0.403 | -0.1467 | No |
| 20 | 9037 | SEMA5A | 688 | 0.385 | -0.1691 | No |
| 21 | 2932 | GSK3B | 714 | 0.367 | -0.1778 | No |
| 22 | 23767 | FLRT3 | 781 | 0.333 | -0.2179 | No |
| 23 | 11091 | WDR5 | 822 | 0.304 | -0.2395 | No |
| 24 | 1605 | DAG1 | 823 | 0.304 | -0.2313 | No |
| 25 | 2909 | ARHGAP35 | 844 | 0.259 | -0.2392 | No |
| 26 | 3312 | HSPA8 | 848 | 0.244 | -0.2349 | No |
| 27 | 3673 | ITGA2 | 858 | -0.264 | -0.2345 | No |
| 28 | 1051 | CEBPB | 862 | -0.274 | -0.2293 | No |
| 29 | 6237 | RRAS | 869 | -0.280 | -0.2263 | No |
| 30 | 10589 | DRAP1 | 882 | -0.300 | -0.2271 | No |
| 31 | 8848 | TSC22D1 | 899 | -0.324 | -0.2303 | No |
| 32 | 4209 | MEF2D | 910 | -0.337 | -0.2287 | No |
| 33 | 4088 | SMAD3 | 930 | -0.353 | -0.2333 | No |
| 34 | 10974 | ADIRF | 962 | -0.382 | -0.2461 | No |
| 35 | 5621 | PRNP | 970 | -0.387 | -0.2409 | No |
| 36 | 9314 | KLF4 | 972 | -0.389 | -0.2312 | No |
| 37 | 7277 | TUBA4A | 997 | -0.404 | -0.2382 | No |
| 38 | 2146 | EZH2 | 1001 | -0.406 | -0.2295 | No |
| 39 | 83896 | KRTAP3-1 | 1006 | -0.408 | -0.2215 | No |
| 40 | 3516 | RBPJ | 1021 | -0.419 | -0.2207 | No |
| 41 | 9939 | RBM8A | 1093 | -0.474 | -0.2607 | No |
| 42 | 6709 | SPTAN1 | 1114 | -0.487 | -0.2625 | Yes |
| 43 | 7204 | TRIO | 1120 | -0.494 | -0.2529 | Yes |
| 44 | 7074 | TIAM1 | 1143 | -0.508 | -0.2556 | Yes |
| 45 | 1282 | COL4A1 | 1165 | -0.534 | -0.2569 | Yes |
| 46 | 3887 | KRT81 | 1168 | -0.536 | -0.2440 | Yes |
| 47 | 6698 | SPRR1A | 1178 | -0.548 | -0.2359 | Yes |
| 48 | 10001 | MED6 | 1190 | -0.561 | -0.2290 | Yes |
| 49 | 8345 | HIST1H2BH | 1207 | -0.585 | -0.2252 | Yes |
| 50 | 4651 | MYO10 | 1213 | -0.588 | -0.2131 | Yes |
| 51 | 3866 | KRT15 | 1214 | -0.589 | -0.1973 | Yes |
| 52 | 27 | ABL2 | 1218 | -0.592 | -0.1836 | Yes |
| 53 | 2043 | EPHA4 | 1228 | -0.602 | -0.1741 | Yes |
| 54 | 2887 | GRB10 | 1259 | -0.646 | -0.1790 | Yes |
| 55 | 1956 | EGFR | 1266 | -0.657 | -0.1658 | Yes |
| 56 | 286 | ANK1 | 1284 | -0.672 | -0.1604 | Yes |
| 57 | 861 | RUNX1 | 1293 | -0.687 | -0.1479 | Yes |
| 58 | 5046 | PCSK6 | 1297 | -0.699 | -0.1313 | Yes |
| 59 | 3892 | KRT86 | 1313 | -0.724 | -0.1230 | Yes |
| 60 | 23365 | ARHGEF12 | 1329 | -0.751 | -0.1140 | Yes |
| 61 | 4233 | MET | 1358 | -0.821 | -0.1127 | Yes |
| 62 | 3860 | KRT13 | 1367 | -0.847 | -0.0959 | Yes |
| 63 | 6711 | SPTBN1 | 1381 | -0.925 | -0.0807 | Yes |
| 64 | 59277 | NTN4 | 1390 | -0.999 | -0.0598 | Yes |
| 65 | 1475 | CSTA | 1400 | -1.162 | -0.0352 | Yes |
| 66 | 6703 | SPRR2D | 1409 | -1.560 | 0.0007 | Yes |
Table: GSEA details [plain text format]

  

Fig 2: REACTOME\_DEVELOPMENTAL\_BIOLOGY      
 Blue-Pink O' Gram in the Space of the Analyzed GeneSet

  

Fig 3: REACTOME\_DEVELOPMENTAL\_BIOLOGY: Random ES distribution      
 Gene set null distribution of ES for **REACTOME\_DEVELOPMENTAL\_BIOLOGY**

  
