# Supplementary material for: Role and mechanism of NCAPD3 in promoting malignant behaviors in gastric cancer
Source: Front Pharmacol. 2024 Apr 22;15:1341039. doi: 10.3389/fphar.2024.1341039 (PMC11070777; doi:10.3389/fphar.2024.1341039)
Supplement: Supplementary file 11 [file DataSheet2.ZIP › GSEA/Canonical pathways/my_analysis.Gsea.1599462267220/REACTOME_DISEASE.html]

Details for gene set REACTOME\_DISEASE[GSEA]

|  || Dataset | filtered\_dataset.sample\_info.cls#WT\_versus\_NCAPD3\_MUT |
| Phenotype | sample\_info.cls#WT\_versus\_NCAPD3\_MUT |
| Upregulated in class | WT |
| GeneSet | REACTOME\_DISEASE |
| Enrichment Score (ES) | 0.20791183 |
| Normalized Enrichment Score (NES) | 1.4241841 |
| Nominal p-value | 0.060150377 |
| FDR q-value | 0.38109875 |
| FWER p-Value | 0.997 |
Table: GSEA Results Summary

  

Fig 1: Enrichment plot: REACTOME\_DISEASE      
 Profile of the Running ES Score & Positions of GeneSet Members on the Rank Ordered List

  

| SYMBOL | TITLE | RANK IN GENE LIST | RANK METRIC SCORE | RUNNING ES | CORE ENRICHMENT || 1 | 2243 | FGA | 18 | 1.039 | 0.0035 | Yes |
| 2 | 54894 | RNF43 | 29 | 1.000 | 0.0125 | Yes |
| 3 | 222236 | NAPEPLD | 36 | 0.977 | 0.0241 | Yes |
| 4 | 9759 | HDAC4 | 46 | 0.925 | 0.0326 | Yes |
| 5 | 2120 | ETV6 | 63 | 0.862 | 0.0347 | Yes |
| 6 | 4204 | MECP2 | 70 | 0.856 | 0.0443 | Yes |
| 7 | 2926 | GRSF1 | 75 | 0.849 | 0.0554 | Yes |
| 8 | 3667 | IRS1 | 83 | 0.821 | 0.0637 | Yes |
| 9 | 6421 | SFPQ | 87 | 0.818 | 0.0750 | Yes |
| 10 | 11052 | CPSF6 | 89 | 0.814 | 0.0877 | Yes |
| 11 | 55343 | SLC35C1 | 92 | 0.804 | 0.0996 | Yes |
| 12 | 79109 | MAPKAP1 | 130 | 0.770 | 0.0841 | Yes |
| 13 | 4193 | MDM2 | 131 | 0.769 | 0.0968 | Yes |
| 14 | 817 | CAMK2D | 165 | 0.710 | 0.0834 | Yes |
| 15 | 54492 | NEURL1B | 177 | 0.697 | 0.0866 | Yes |
| 16 | 613 | BCR | 180 | 0.695 | 0.0966 | Yes |
| 17 | 6191 | RPS4X | 196 | 0.682 | 0.0965 | Yes |
| 18 | 3303 | HSPA1A | 206 | 0.669 | 0.1007 | Yes |
| 19 | 4836 | NMT1 | 208 | 0.669 | 0.1111 | Yes |
| 20 | 126792 | B3GALT6 | 223 | 0.658 | 0.1113 | Yes |
| 21 | 5701 | PSMC2 | 244 | 0.636 | 0.1066 | Yes |
| 22 | 51135 | IRAK4 | 245 | 0.635 | 0.1171 | Yes |
| 23 | 6850 | SYK | 256 | 0.625 | 0.1198 | Yes |
| 24 | 25974 | MMACHC | 265 | 0.621 | 0.1241 | Yes |
| 25 | 22845 | DOLK | 269 | 0.618 | 0.1320 | Yes |
| 26 | 199857 | ALG14 | 275 | 0.615 | 0.1384 | Yes |
| 27 | 2632 | GBE1 | 280 | 0.609 | 0.1455 | Yes |
| 28 | 324 | APC | 281 | 0.607 | 0.1556 | Yes |
| 29 | 10585 | POMT1 | 284 | 0.606 | 0.1641 | Yes |
| 30 | 5728 | PTEN | 285 | 0.606 | 0.1742 | Yes |
| 31 | 2244 | FGB | 321 | 0.582 | 0.1571 | Yes |
| 32 | 1021 | CDK6 | 324 | 0.579 | 0.1652 | Yes |
| 33 | 55869 | HDAC8 | 352 | 0.566 | 0.1539 | Yes |
| 34 | 2965 | GTF2H1 | 356 | 0.563 | 0.1610 | Yes |
| 35 | 10818 | FRS2 | 363 | 0.558 | 0.1657 | Yes |
| 36 | 1080 | CFTR | 366 | 0.557 | 0.1734 | Yes |
| 37 | 166785 | MMAA | 369 | 0.553 | 0.1811 | Yes |
| 38 | 326625 | MMAB | 371 | 0.552 | 0.1895 | Yes |
| 39 | 5718 | PSMD12 | 372 | 0.552 | 0.1987 | Yes |
| 40 | 2997 | GYS1 | 376 | 0.548 | 0.2055 | Yes |
| 41 | 1387 | CREBBP | 387 | 0.544 | 0.2069 | Yes |
| 42 | 79651 | RHBDF2 | 422 | 0.519 | 0.1895 | Yes |
| 43 | 801 | CALM1 | 424 | 0.518 | 0.1973 | Yes |
| 44 | 4247 | MGAT2 | 432 | 0.513 | 0.2005 | Yes |
| 45 | 29071 | C1GALT1C1 | 457 | 0.500 | 0.1904 | Yes |
| 46 | 6507 | SLC1A3 | 464 | 0.496 | 0.1941 | Yes |
| 47 | 5295 | PIK3R1 | 467 | 0.493 | 0.2008 | Yes |
| 48 | 5245 | PHB | 481 | 0.486 | 0.1989 | Yes |
| 49 | 1793 | DOCK1 | 489 | 0.480 | 0.2015 | Yes |
| 50 | 8454 | CUL1 | 492 | 0.477 | 0.2079 | Yes |
| 51 | 81031 | SLC2A10 | 539 | 0.453 | 0.1802 | No |
| 52 | 7099 | TLR4 | 541 | 0.451 | 0.1870 | No |
| 53 | 3836 | KPNA1 | 548 | 0.448 | 0.1898 | No |
| 54 | 84335 | AKT1S1 | 572 | 0.437 | 0.1795 | No |
| 55 | 79053 | ALG8 | 576 | 0.436 | 0.1844 | No |
| 56 | 4548 | MTR | 592 | 0.428 | 0.1801 | No |
| 57 | 5567 | PRKACB | 595 | 0.426 | 0.1856 | No |
| 58 | 6208 | RPS14 | 604 | 0.421 | 0.1865 | No |
| 59 | 80824 | DUSP16 | 610 | 0.418 | 0.1896 | No |
| 60 | 8021 | NUP214 | 611 | 0.417 | 0.1966 | No |
| 61 | 113189 | CHST14 | 622 | 0.411 | 0.1958 | No |
| 62 | 9037 | SEMA5A | 688 | 0.385 | 0.1524 | No |
| 63 | 5987 | TRIM27 | 695 | 0.380 | 0.1541 | No |
| 64 | 55750 | AGK | 697 | 0.380 | 0.1597 | No |
| 65 | 4669 | NAGLU | 707 | 0.373 | 0.1590 | No |
| 66 | 2932 | GSK3B | 714 | 0.367 | 0.1605 | No |
| 67 | 5613 | PRKX | 722 | 0.363 | 0.1612 | No |
| 68 | 57674 | RNF213 | 730 | 0.359 | 0.1618 | No |
| 69 | 64087 | MCCC2 | 747 | 0.352 | 0.1554 | No |
| 70 | 4869 | NPM1 | 772 | 0.336 | 0.1426 | No |
| 71 | 31 | ACACA | 780 | 0.334 | 0.1428 | No |
| 72 | 5901 | RAN | 810 | 0.313 | 0.1258 | No |
| 73 | 1605 | DAG1 | 823 | 0.304 | 0.1217 | No |
| 74 | 6385 | SDC4 | 871 | -0.282 | 0.0904 | No |
| 75 | 7296 | TXNRD1 | 877 | -0.289 | 0.0914 | No |
| 76 | 6520 | SLC3A2 | 878 | -0.290 | 0.0962 | No |
| 77 | 3164 | NR4A1 | 912 | -0.341 | 0.0767 | No |
| 78 | 4088 | SMAD3 | 930 | -0.353 | 0.0695 | No |
| 79 | 9208 | LRRFIP1 | 995 | -0.402 | 0.0272 | No |
| 80 | 7277 | TUBA4A | 997 | -0.404 | 0.0332 | No |
| 81 | 2146 | EZH2 | 1001 | -0.406 | 0.0376 | No |
| 82 | 2673 | GFPT1 | 1016 | -0.414 | 0.0338 | No |
| 83 | 3516 | RBPJ | 1021 | -0.419 | 0.0377 | No |
| 84 | 10018 | BCL2L11 | 1027 | -0.425 | 0.0409 | No |
| 85 | 10681 | GNB5 | 1105 | -0.481 | -0.0100 | No |
| 86 | 2729 | GCLC | 1123 | -0.497 | -0.0147 | No |
| 87 | 831 | CAST | 1160 | -0.524 | -0.0335 | No |
| 88 | 7518 | XRCC4 | 1175 | -0.540 | -0.0353 | No |
| 89 | 5154 | PDGFA | 1191 | -0.566 | -0.0374 | No |
| 90 | 8345 | HIST1H2BH | 1207 | -0.585 | -0.0391 | No |
| 91 | 4651 | MYO10 | 1213 | -0.588 | -0.0332 | No |
| 92 | 153 | ADRB1 | 1221 | -0.595 | -0.0286 | No |
| 93 | 26999 | CYFIP2 | 1234 | -0.611 | -0.0277 | No |
| 94 | 7057 | THBS1 | 1242 | -0.620 | -0.0227 | No |
| 95 | 1956 | EGFR | 1266 | -0.657 | -0.0294 | No |
| 96 | 7414 | VCL | 1308 | -0.712 | -0.0489 | No |
| 97 | 80853 | KDM7A | 1311 | -0.714 | -0.0386 | No |
| 98 | 3084 | NRG1 | 1320 | -0.739 | -0.0324 | No |
| 99 | 1839 | HBEGF | 1328 | -0.751 | -0.0253 | No |
| 100 | 4233 | MET | 1358 | -0.821 | -0.0338 | No |
| 101 | 9990 | SLC12A6 | 1379 | -0.922 | -0.0338 | No |
| 102 | 5156 | PDGFRA | 1383 | -0.932 | -0.0206 | No |
| 103 | 2591 | GALNT3 | 1403 | -1.195 | -0.0153 | No |
| 104 | 6519 | SLC3A1 | 1404 | -1.195 | 0.0046 | No |
Table: GSEA details [plain text format]

  

Fig 2: REACTOME\_DISEASE      
 Blue-Pink O' Gram in the Space of the Analyzed GeneSet

  

Fig 3: REACTOME\_DISEASE: Random ES distribution      
 Gene set null distribution of ES for **REACTOME\_DISEASE**

  
