# Supplementary material for: Role and mechanism of NCAPD3 in promoting malignant behaviors in gastric cancer
Source: Front Pharmacol. 2024 Apr 22;15:1341039. doi: 10.3389/fphar.2024.1341039 (PMC11070777; doi:10.3389/fphar.2024.1341039)
Supplement: Supplementary file 11 [file DataSheet2.ZIP › GSEA/Canonical pathways/my_analysis.Gsea.1599462267220/REACTOME_DISEASES_OF_SIGNAL_TRANSDUCTION_BY_GROWTH_FACTOR_RECEPTORS_AND_SECOND_MESSENGERS.html]

Details for gene set REACTOME\_DISEASES\_OF\_SIGNAL\_TRANSDUCTION\_BY\_GROWTH\_FACTOR\_RECEPTORS\_AND\_SECOND\_MESSENGERS[GSEA]

|  || Dataset | filtered\_dataset.sample\_info.cls#WT\_versus\_NCAPD3\_MUT |
| Phenotype | sample\_info.cls#WT\_versus\_NCAPD3\_MUT |
| Upregulated in class | WT |
| GeneSet | REACTOME\_DISEASES\_OF\_SIGNAL\_TRANSDUCTION\_BY\_GROWTH\_FACTOR\_RECEPTORS\_AND\_SECOND\_MESSENGERS |
| Enrichment Score (ES) | 0.29309416 |
| Normalized Enrichment Score (NES) | 1.5832915 |
| Nominal p-value | 0.040609136 |
| FDR q-value | 0.3830722 |
| FWER p-Value | 0.92 |
Table: GSEA Results Summary

  

Fig 1: Enrichment plot: REACTOME\_DISEASES\_OF\_SIGNAL\_TRANSDUCTION\_BY\_GROWTH\_FACTOR\_RECEPTORS\_AND\_SECOND\_MESSENGERS      
 Profile of the Running ES Score & Positions of GeneSet Members on the Rank Ordered List

  

| SYMBOL | TITLE | RANK IN GENE LIST | RANK METRIC SCORE | RUNNING ES | CORE ENRICHMENT || 1 | 2243 | FGA | 18 | 1.039 | 0.0289 | Yes |
| 2 | 54894 | RNF43 | 29 | 1.000 | 0.0620 | Yes |
| 3 | 9759 | HDAC4 | 46 | 0.925 | 0.0877 | Yes |
| 4 | 2120 | ETV6 | 63 | 0.862 | 0.1109 | Yes |
| 5 | 3667 | IRS1 | 83 | 0.821 | 0.1302 | Yes |
| 6 | 11052 | CPSF6 | 89 | 0.814 | 0.1595 | Yes |
| 7 | 79109 | MAPKAP1 | 130 | 0.770 | 0.1614 | Yes |
| 8 | 4193 | MDM2 | 131 | 0.769 | 0.1925 | Yes |
| 9 | 817 | CAMK2D | 165 | 0.710 | 0.1972 | Yes |
| 10 | 54492 | NEURL1B | 177 | 0.697 | 0.2173 | Yes |
| 11 | 613 | BCR | 180 | 0.695 | 0.2440 | Yes |
| 12 | 5701 | PSMC2 | 244 | 0.636 | 0.2238 | Yes |
| 13 | 324 | APC | 281 | 0.607 | 0.2221 | Yes |
| 14 | 5728 | PTEN | 285 | 0.606 | 0.2444 | Yes |
| 15 | 2244 | FGB | 321 | 0.582 | 0.2424 | Yes |
| 16 | 55869 | HDAC8 | 352 | 0.566 | 0.2434 | Yes |
| 17 | 10818 | FRS2 | 363 | 0.558 | 0.2587 | Yes |
| 18 | 5718 | PSMD12 | 372 | 0.552 | 0.2751 | Yes |
| 19 | 1387 | CREBBP | 387 | 0.544 | 0.2869 | Yes |
| 20 | 801 | CALM1 | 424 | 0.518 | 0.2816 | Yes |
| 21 | 5295 | PIK3R1 | 467 | 0.493 | 0.2709 | Yes |
| 22 | 5245 | PHB | 481 | 0.486 | 0.2811 | Yes |
| 23 | 8454 | CUL1 | 492 | 0.477 | 0.2931 | Yes |
| 24 | 84335 | AKT1S1 | 572 | 0.437 | 0.2532 | No |
| 25 | 55750 | AGK | 697 | 0.380 | 0.1781 | No |
| 26 | 2932 | GSK3B | 714 | 0.367 | 0.1813 | No |
| 27 | 3164 | NR4A1 | 912 | -0.341 | 0.0515 | No |
| 28 | 4088 | SMAD3 | 930 | -0.353 | 0.0534 | No |
| 29 | 9208 | LRRFIP1 | 995 | -0.402 | 0.0230 | No |
| 30 | 3516 | RBPJ | 1021 | -0.419 | 0.0217 | No |
| 31 | 10018 | BCL2L11 | 1027 | -0.425 | 0.0352 | No |
| 32 | 5154 | PDGFA | 1191 | -0.566 | -0.0607 | No |
| 33 | 1956 | EGFR | 1266 | -0.657 | -0.0881 | No |
| 34 | 7414 | VCL | 1308 | -0.712 | -0.0892 | No |
| 35 | 80853 | KDM7A | 1311 | -0.714 | -0.0618 | No |
| 36 | 3084 | NRG1 | 1320 | -0.739 | -0.0378 | No |
| 37 | 1839 | HBEGF | 1328 | -0.751 | -0.0125 | No |
| 38 | 4233 | MET | 1358 | -0.821 | -0.0005 | No |
| 39 | 5156 | PDGFRA | 1383 | -0.932 | 0.0197 | No |
Table: GSEA details [plain text format]

  

Fig 2: REACTOME\_DISEASES\_OF\_SIGNAL\_TRANSDUCTION\_BY\_GROWTH\_FACTOR\_RECEPTORS\_AND\_SECOND\_MESSENGERS      
 Blue-Pink O' Gram in the Space of the Analyzed GeneSet

  

Fig 3: REACTOME\_DISEASES\_OF\_SIGNAL\_TRANSDUCTION\_BY\_GROWTH\_FACTOR\_RECEPTORS\_AND\_SECOND\_MESSENGERS: Random ES distribution      
 Gene set null distribution of ES for **REACTOME\_DISEASES\_OF\_SIGNAL\_TRANSDUCTION\_BY\_GROWTH\_FACTOR\_RECEPTORS\_AND\_SECOND\_MESSENGERS**

  
