# Supplementary material for: Role and mechanism of NCAPD3 in promoting malignant behaviors in gastric cancer
Source: Front Pharmacol. 2024 Apr 22;15:1341039. doi: 10.3389/fphar.2024.1341039 (PMC11070777; doi:10.3389/fphar.2024.1341039)
Supplement: Supplementary file 11 [file DataSheet2.ZIP › GSEA/Canonical pathways/my_analysis.Gsea.1599462267220/REACTOME_DNA_REPAIR.html]

Details for gene set REACTOME\_DNA\_REPAIR[GSEA]

|  || Dataset | filtered\_dataset.sample\_info.cls#WT\_versus\_NCAPD3\_MUT |
| Phenotype | sample\_info.cls#WT\_versus\_NCAPD3\_MUT |
| Upregulated in class | WT |
| GeneSet | REACTOME\_DNA\_REPAIR |
| Enrichment Score (ES) | 0.24363984 |
| Normalized Enrichment Score (NES) | 1.0794839 |
| Nominal p-value | 0.3597973 |
| FDR q-value | 0.73870045 |
| FWER p-Value | 1.0 |
Table: GSEA Results Summary

  

Fig 1: Enrichment plot: REACTOME\_DNA\_REPAIR      
 Profile of the Running ES Score & Positions of GeneSet Members on the Rank Ordered List

  

| SYMBOL | TITLE | RANK IN GENE LIST | RANK METRIC SCORE | RUNNING ES | CORE ENRICHMENT || 1 | 2072 | ERCC4 | 7 | 1.130 | 0.0826 | Yes |
| 2 | 84142 | FAM175A | 211 | 0.667 | -0.0119 | Yes |
| 3 | 1161 | ERCC8 | 255 | 0.625 | 0.0056 | Yes |
| 4 | 7014 | TERF2 | 323 | 0.579 | 0.0022 | Yes |
| 5 | 79184 | BRCC3 | 348 | 0.569 | 0.0290 | Yes |
| 6 | 2965 | GTF2H1 | 356 | 0.563 | 0.0677 | Yes |
| 7 | 5810 | RAD1 | 360 | 0.560 | 0.1089 | Yes |
| 8 | 83932 | SPRTN | 404 | 0.535 | 0.1194 | Yes |
| 9 | 85015 | USP45 | 434 | 0.513 | 0.1383 | Yes |
| 10 | 8607 | RUVBL1 | 451 | 0.506 | 0.1660 | Yes |
| 11 | 5429 | POLH | 471 | 0.490 | 0.1903 | Yes |
| 12 | 2140 | EYA3 | 528 | 0.458 | 0.1855 | Yes |
| 13 | 2188 | FANCF | 545 | 0.449 | 0.2088 | Yes |
| 14 | 7334 | UBE2N | 546 | 0.449 | 0.2436 | Yes |
| 15 | 10445 | MCRS1 | 617 | 0.414 | 0.2253 | No |
| 16 | 5889 | RAD51C | 1059 | -0.451 | -0.0574 | No |
| 17 | 5111 | PCNA | 1077 | -0.460 | -0.0340 | No |
| 18 | 2175 | FANCA | 1092 | -0.469 | -0.0077 | No |
| 19 | 7468 | WHSC1 | 1122 | -0.496 | 0.0099 | No |
| 20 | 7518 | XRCC4 | 1175 | -0.540 | 0.0144 | No |
| 21 | 472 | ATM | 1193 | -0.568 | 0.0462 | No |
| 22 | 8345 | HIST1H2BH | 1207 | -0.585 | 0.0822 | No |
| 23 | 7706 | TRIM25 | 1357 | -0.817 | 0.0382 | No |
Table: GSEA details [plain text format]

  

Fig 2: REACTOME\_DNA\_REPAIR      
 Blue-Pink O' Gram in the Space of the Analyzed GeneSet

  

Fig 3: REACTOME\_DNA\_REPAIR: Random ES distribution      
 Gene set null distribution of ES for **REACTOME\_DNA\_REPAIR**

  
