# Supplementary material for: Role and mechanism of NCAPD3 in promoting malignant behaviors in gastric cancer
Source: Front Pharmacol. 2024 Apr 22;15:1341039. doi: 10.3389/fphar.2024.1341039 (PMC11070777; doi:10.3389/fphar.2024.1341039)
Supplement: Supplementary file 11 [file DataSheet2.ZIP › GSEA/Canonical pathways/my_analysis.Gsea.1599462267220/REACTOME_G_ALPHA_I_SIGNALLING_EVENTS.html]

Details for gene set REACTOME\_G\_ALPHA\_I\_SIGNALLING\_EVENTS[GSEA]

|  || Dataset | filtered\_dataset.sample\_info.cls#WT\_versus\_NCAPD3\_MUT |
| Phenotype | sample\_info.cls#WT\_versus\_NCAPD3\_MUT |
| Upregulated in class | WT |
| GeneSet | REACTOME\_G\_ALPHA\_I\_SIGNALLING\_EVENTS |
| Enrichment Score (ES) | 0.3864249 |
| Normalized Enrichment Score (NES) | 1.4569799 |
| Nominal p-value | 0.081771724 |
| FDR q-value | 0.418524 |
| FWER p-Value | 0.987 |
Table: GSEA Results Summary

  

Fig 1: Enrichment plot: REACTOME\_G\_ALPHA\_I\_SIGNALLING\_EVENTS      
 Profile of the Running ES Score & Positions of GeneSet Members on the Rank Ordered List

  

| SYMBOL | TITLE | RANK IN GENE LIST | RANK METRIC SCORE | RUNNING ES | CORE ENRICHMENT || 1 | 222236 | NAPEPLD | 36 | 0.977 | 0.0948 | Yes |
| 2 | 2921 | CXCL3 | 57 | 0.884 | 0.1896 | Yes |
| 3 | 817 | CAMK2D | 165 | 0.710 | 0.2007 | Yes |
| 4 | 4836 | NMT1 | 208 | 0.669 | 0.2532 | Yes |
| 5 | 2342 | FNTB | 344 | 0.570 | 0.2268 | Yes |
| 6 | 2919 | CXCL1 | 405 | 0.534 | 0.2498 | Yes |
| 7 | 801 | CALM1 | 424 | 0.518 | 0.3009 | Yes |
| 8 | 2920 | CXCL2 | 454 | 0.501 | 0.3420 | Yes |
| 9 | 4987 | OPRL1 | 477 | 0.487 | 0.3864 | Yes |
| 10 | 5567 | PRKACB | 595 | 0.426 | 0.3553 | No |
| 11 | 5613 | PRKX | 722 | 0.363 | 0.3098 | No |
| 12 | 6385 | SDC4 | 871 | -0.282 | 0.2386 | No |
| 13 | 301 | ANXA1 | 914 | -0.342 | 0.2508 | No |
| 14 | 3949 | LDLR | 929 | -0.353 | 0.2844 | No |
| 15 | 10681 | GNB5 | 1105 | -0.481 | 0.2185 | No |
Table: GSEA details [plain text format]

  

Fig 2: REACTOME\_G\_ALPHA\_I\_SIGNALLING\_EVENTS      
 Blue-Pink O' Gram in the Space of the Analyzed GeneSet

  

Fig 3: REACTOME\_G\_ALPHA\_I\_SIGNALLING\_EVENTS: Random ES distribution      
 Gene set null distribution of ES for **REACTOME\_G\_ALPHA\_I\_SIGNALLING\_EVENTS**

  
