# Supplementary material for: Role and mechanism of NCAPD3 in promoting malignant behaviors in gastric cancer
Source: Front Pharmacol. 2024 Apr 22;15:1341039. doi: 10.3389/fphar.2024.1341039 (PMC11070777; doi:10.3389/fphar.2024.1341039)
Supplement: Supplementary file 11 [file DataSheet2.ZIP › GSEA/Canonical pathways/my_analysis.Gsea.1599462267220/REACTOME_HEMOSTASIS.html]

Details for gene set REACTOME\_HEMOSTASIS[GSEA]

|  || Dataset | filtered\_dataset.sample\_info.cls#WT\_versus\_NCAPD3\_MUT |
| Phenotype | sample\_info.cls#WT\_versus\_NCAPD3\_MUT |
| Upregulated in class | NCAPD3\_MUT |
| GeneSet | REACTOME\_HEMOSTASIS |
| Enrichment Score (ES) | -0.33307794 |
| Normalized Enrichment Score (NES) | -2.044452 |
| Nominal p-value | 0.0 |
| FDR q-value | 0.024228873 |
| FWER p-Value | 0.076 |
Table: GSEA Results Summary

  

Fig 1: Enrichment plot: REACTOME\_HEMOSTASIS      
 Profile of the Running ES Score & Positions of GeneSet Members on the Rank Ordered List

  

| SYMBOL | TITLE | RANK IN GENE LIST | RANK METRIC SCORE | RUNNING ES | CORE ENRICHMENT || 1 | 2243 | FGA | 18 | 1.039 | 0.0292 | No |
| 2 | 5099 | PCDH7 | 23 | 1.026 | 0.0682 | No |
| 3 | 6850 | SYK | 256 | 0.625 | -0.0761 | No |
| 4 | 2244 | FGB | 321 | 0.582 | -0.0992 | No |
| 5 | 960 | CD44 | 339 | 0.573 | -0.0883 | No |
| 6 | 801 | CALM1 | 424 | 0.518 | -0.1287 | No |
| 7 | 5295 | PIK3R1 | 467 | 0.493 | -0.1393 | No |
| 8 | 23468 | CBX5 | 484 | 0.485 | -0.1312 | No |
| 9 | 1793 | DOCK1 | 489 | 0.480 | -0.1145 | No |
| 10 | 3479 | IGF1 | 511 | 0.469 | -0.1107 | No |
| 11 | 5567 | PRKACB | 595 | 0.426 | -0.1541 | No |
| 12 | 9749 | PHACTR2 | 806 | 0.319 | -0.2948 | No |
| 13 | 3673 | ITGA2 | 858 | -0.264 | -0.3214 | Yes |
| 14 | 6385 | SDC4 | 871 | -0.282 | -0.3186 | Yes |
| 15 | 3309 | HSPA5 | 876 | -0.288 | -0.3098 | Yes |
| 16 | 6520 | SLC3A2 | 878 | -0.290 | -0.2987 | Yes |
| 17 | 8140 | SLC7A5 | 890 | -0.305 | -0.2943 | Yes |
| 18 | 4973 | OLR1 | 944 | -0.366 | -0.3182 | Yes |
| 19 | 5329 | PLAUR | 952 | -0.373 | -0.3081 | Yes |
| 20 | 4680 | CEACAM6 | 955 | -0.374 | -0.2943 | Yes |
| 21 | 7277 | TUBA4A | 997 | -0.404 | -0.3078 | Yes |
| 22 | 10938 | EHD1 | 1019 | -0.419 | -0.3061 | Yes |
| 23 | 23046 | KIF21B | 1034 | -0.433 | -0.2986 | Yes |
| 24 | 23764 | MAFF | 1039 | -0.436 | -0.2838 | Yes |
| 25 | 10019 | SH2B3 | 1044 | -0.440 | -0.2687 | Yes |
| 26 | 80005 | DOCK5 | 1045 | -0.441 | -0.2508 | Yes |
| 27 | 5673 | PSG5 | 1047 | -0.444 | -0.2334 | Yes |
| 28 | 5671 | PSG3 | 1048 | -0.444 | -0.2152 | Yes |
| 29 | 54676 | GTPBP2 | 1050 | -0.447 | -0.1977 | Yes |
| 30 | 1191 | CLU | 1056 | -0.450 | -0.1830 | Yes |
| 31 | 5889 | RAD51C | 1059 | -0.451 | -0.1660 | Yes |
| 32 | 857 | CAV1 | 1098 | -0.475 | -0.1745 | Yes |
| 33 | 10681 | GNB5 | 1105 | -0.481 | -0.1592 | Yes |
| 34 | 90952 | ESAM | 1149 | -0.514 | -0.1697 | Yes |
| 35 | 2152 | F3 | 1188 | -0.559 | -0.1747 | Yes |
| 36 | 5154 | PDGFA | 1191 | -0.566 | -0.1530 | Yes |
| 37 | 23657 | SLC7A11 | 1205 | -0.581 | -0.1388 | Yes |
| 38 | 2734 | GLG1 | 1240 | -0.618 | -0.1385 | Yes |
| 39 | 7057 | THBS1 | 1242 | -0.620 | -0.1139 | Yes |
| 40 | 1893 | ECM1 | 1250 | -0.637 | -0.0930 | Yes |
| 41 | 7414 | VCL | 1308 | -0.712 | -0.1057 | Yes |
| 42 | 7422 | VEGFA | 1346 | -0.788 | -0.1006 | Yes |
| 43 | 5055 | SERPINB2 | 1397 | -1.088 | -0.0928 | Yes |
| 44 | 493 | ATP2B4 | 1401 | -1.166 | -0.0474 | Yes |
| 45 | 4312 | MMP1 | 1407 | -1.304 | 0.0022 | Yes |
Table: GSEA details [plain text format]

  

Fig 2: REACTOME\_HEMOSTASIS      
 Blue-Pink O' Gram in the Space of the Analyzed GeneSet

  

Fig 3: REACTOME\_HEMOSTASIS: Random ES distribution      
 Gene set null distribution of ES for **REACTOME\_HEMOSTASIS**

  
