# Supplementary material for: Role and mechanism of NCAPD3 in promoting malignant behaviors in gastric cancer
Source: Front Pharmacol. 2024 Apr 22;15:1341039. doi: 10.3389/fphar.2024.1341039 (PMC11070777; doi:10.3389/fphar.2024.1341039)
Supplement: Supplementary file 11 [file DataSheet2.ZIP › GSEA/Canonical pathways/my_analysis.Gsea.1599462267220/REACTOME_METABOLISM_OF_RNA.html]

Details for gene set REACTOME\_METABOLISM\_OF\_RNA[GSEA]

|  || Dataset | filtered\_dataset.sample\_info.cls#WT\_versus\_NCAPD3\_MUT |
| Phenotype | sample\_info.cls#WT\_versus\_NCAPD3\_MUT |
| Upregulated in class | WT |
| GeneSet | REACTOME\_METABOLISM\_OF\_RNA |
| Enrichment Score (ES) | 0.35255572 |
| Normalized Enrichment Score (NES) | 2.314195 |
| Nominal p-value | 0.0 |
| FDR q-value | 0.0026635504 |
| FWER p-Value | 0.004 |
Table: GSEA Results Summary

  

Fig 1: Enrichment plot: REACTOME\_METABOLISM\_OF\_RNA      
 Profile of the Running ES Score & Positions of GeneSet Members on the Rank Ordered List

  

| SYMBOL | TITLE | RANK IN GENE LIST | RANK METRIC SCORE | RUNNING ES | CORE ENRICHMENT || 1 | 51585 | PCF11 | 3 | 1.212 | 0.0278 | Yes |
| 2 | 2935 | GSPT1 | 5 | 1.156 | 0.0557 | Yes |
| 3 | 8568 | RRP1 | 22 | 1.027 | 0.0691 | Yes |
| 4 | 80746 | TSEN2 | 53 | 0.909 | 0.0690 | Yes |
| 5 | 6428 | SRSF3 | 55 | 0.907 | 0.0908 | Yes |
| 6 | 55006 | TRMT61B | 101 | 0.782 | 0.0763 | Yes |
| 7 | 55687 | TRMU | 128 | 0.774 | 0.0759 | Yes |
| 8 | 55781 | RIOK2 | 135 | 0.763 | 0.0903 | Yes |
| 9 | 23450 | SF3B3 | 178 | 0.697 | 0.0760 | Yes |
| 10 | 6191 | RPS4X | 196 | 0.682 | 0.0801 | Yes |
| 11 | 3303 | HSPA1A | 206 | 0.669 | 0.0899 | Yes |
| 12 | 79922 | MRM1 | 209 | 0.669 | 0.1050 | Yes |
| 13 | 127253 | TYW3 | 225 | 0.654 | 0.1099 | Yes |
| 14 | 55599 | RNPC3 | 227 | 0.652 | 0.1253 | Yes |
| 15 | 6426 | SRSF1 | 243 | 0.637 | 0.1298 | Yes |
| 16 | 5701 | PSMC2 | 244 | 0.636 | 0.1456 | Yes |
| 17 | 9724 | UTP14C | 301 | 0.591 | 0.1181 | Yes |
| 18 | 84946 | LTV1 | 319 | 0.583 | 0.1198 | Yes |
| 19 | 8125 | ANP32A | 326 | 0.578 | 0.1296 | Yes |
| 20 | 84135 | UTP15 | 330 | 0.576 | 0.1416 | Yes |
| 21 | 5356 | PLRG1 | 338 | 0.573 | 0.1505 | Yes |
| 22 | 2965 | GTF2H1 | 356 | 0.563 | 0.1517 | Yes |
| 23 | 5718 | PSMD12 | 372 | 0.552 | 0.1541 | Yes |
| 24 | 54517 | PUS7 | 388 | 0.544 | 0.1563 | Yes |
| 25 | 10212 | DDX39A | 389 | 0.543 | 0.1697 | Yes |
| 26 | 10885 | WDR3 | 430 | 0.515 | 0.1524 | Yes |
| 27 | 54931 | TRMT10C | 446 | 0.507 | 0.1537 | Yes |
| 28 | 54482 | TRMT13 | 449 | 0.506 | 0.1647 | Yes |
| 29 | 27340 | UTP20 | 455 | 0.501 | 0.1734 | Yes |
| 30 | 10785 | WDR4 | 465 | 0.495 | 0.1789 | Yes |
| 31 | 10291 | SF3A1 | 466 | 0.493 | 0.1911 | Yes |
| 32 | 10969 | EBNA1BP2 | 483 | 0.485 | 0.1911 | Yes |
| 33 | 10940 | POP1 | 497 | 0.474 | 0.1930 | Yes |
| 34 | 1479 | CSTF3 | 500 | 0.474 | 0.2033 | Yes |
| 35 | 3184 | HNRNPD | 508 | 0.470 | 0.2096 | Yes |
| 36 | 54920 | DUS2 | 509 | 0.470 | 0.2213 | Yes |
| 37 | 1660 | DHX9 | 510 | 0.469 | 0.2329 | Yes |
| 38 | 104 | ADARB1 | 518 | 0.463 | 0.2391 | Yes |
| 39 | 9836 | LCMT2 | 549 | 0.448 | 0.2276 | Yes |
| 40 | 55339 | WDR33 | 551 | 0.445 | 0.2379 | Yes |
| 41 | 9416 | DDX23 | 553 | 0.445 | 0.2482 | Yes |
| 42 | 79707 | NOL9 | 560 | 0.441 | 0.2546 | Yes |
| 43 | 6632 | SNRPD1 | 563 | 0.440 | 0.2640 | Yes |
| 44 | 51388 | NIP7 | 593 | 0.428 | 0.2528 | Yes |
| 45 | 55039 | TRMT12 | 599 | 0.424 | 0.2596 | Yes |
| 46 | 55181 | SMG8 | 602 | 0.422 | 0.2685 | Yes |
| 47 | 6208 | RPS14 | 604 | 0.421 | 0.2782 | Yes |
| 48 | 23404 | EXOSC2 | 609 | 0.418 | 0.2855 | Yes |
| 49 | 8021 | NUP214 | 611 | 0.417 | 0.2951 | Yes |
| 50 | 56339 | METTL3 | 613 | 0.417 | 0.3047 | Yes |
| 51 | 54512 | EXOSC4 | 623 | 0.411 | 0.3081 | Yes |
| 52 | 6427 | SRSF2 | 679 | 0.388 | 0.2763 | Yes |
| 53 | 10607 | TBL3 | 698 | 0.380 | 0.2722 | Yes |
| 54 | 79670 | ZCCHC6 | 700 | 0.378 | 0.2808 | Yes |
| 55 | 23350 | U2SURP | 719 | 0.364 | 0.2763 | Yes |
| 56 | 55759 | WDR12 | 731 | 0.359 | 0.2769 | Yes |
| 57 | 134430 | WDR36 | 733 | 0.359 | 0.2851 | Yes |
| 58 | 51602 | NOP58 | 750 | 0.348 | 0.2817 | Yes |
| 59 | 27316 | RBMX | 762 | 0.342 | 0.2819 | Yes |
| 60 | 3188 | HNRNPH2 | 769 | 0.338 | 0.2857 | Yes |
| 61 | 55178 | RNMTL1 | 771 | 0.337 | 0.2933 | Yes |
| 62 | 51118 | UTP11L | 773 | 0.336 | 0.3009 | Yes |
| 63 | 4670 | HNRNPM | 790 | 0.330 | 0.2970 | Yes |
| 64 | 1736 | DKC1 | 791 | 0.330 | 0.3052 | Yes |
| 65 | 23246 | BOP1 | 797 | 0.325 | 0.3095 | Yes |
| 66 | 27341 | RRP7A | 801 | 0.320 | 0.3152 | Yes |
| 67 | 1973 | EIF4A1 | 804 | 0.319 | 0.3216 | Yes |
| 68 | 29960 | FTSJ2 | 805 | 0.319 | 0.3295 | Yes |
| 69 | 5901 | RAN | 810 | 0.313 | 0.3342 | Yes |
| 70 | 9343 | EFTUD2 | 818 | 0.305 | 0.3365 | Yes |
| 71 | 26121 | PRPF31 | 825 | 0.301 | 0.3394 | Yes |
| 72 | 11157 | LSM6 | 830 | 0.296 | 0.3438 | Yes |
| 73 | 10528 | NOP56 | 837 | 0.285 | 0.3463 | Yes |
| 74 | 4691 | NCL | 843 | 0.270 | 0.3493 | Yes |
| 75 | 3312 | HSPA8 | 848 | 0.244 | 0.3523 | Yes |
| 76 | 200315 | APOBEC3A | 866 | -0.278 | 0.3464 | Yes |
| 77 | 9582 | APOBEC3B | 868 | -0.278 | 0.3526 | Yes |
| 78 | 8780 | RIOK3 | 948 | -0.367 | 0.3023 | No |
| 79 | 9939 | RBM8A | 1093 | -0.474 | 0.2057 | No |
| 80 | 6430 | SRSF5 | 1183 | -0.553 | 0.1525 | No |
| 81 | 54464 | XRN1 | 1301 | -0.703 | 0.0820 | No |
Table: GSEA details [plain text format]

  

Fig 2: REACTOME\_METABOLISM\_OF\_RNA      
 Blue-Pink O' Gram in the Space of the Analyzed GeneSet

  

Fig 3: REACTOME\_METABOLISM\_OF\_RNA: Random ES distribution      
 Gene set null distribution of ES for **REACTOME\_METABOLISM\_OF\_RNA**

  
