# Supplementary material for: Role and mechanism of NCAPD3 in promoting malignant behaviors in gastric cancer
Source: Front Pharmacol. 2024 Apr 22;15:1341039. doi: 10.3389/fphar.2024.1341039 (PMC11070777; doi:10.3389/fphar.2024.1341039)
Supplement: Supplementary file 11 [file DataSheet2.ZIP › GSEA/Canonical pathways/my_analysis.Gsea.1599462267220/REACTOME_METABOLISM_OF_STEROIDS.html]

Details for gene set REACTOME\_METABOLISM\_OF\_STEROIDS[GSEA]

|  || Dataset | filtered\_dataset.sample\_info.cls#WT\_versus\_NCAPD3\_MUT |
| Phenotype | sample\_info.cls#WT\_versus\_NCAPD3\_MUT |
| Upregulated in class | WT |
| GeneSet | REACTOME\_METABOLISM\_OF\_STEROIDS |
| Enrichment Score (ES) | 0.27781415 |
| Normalized Enrichment Score (NES) | 1.1103362 |
| Nominal p-value | 0.3374126 |
| FDR q-value | 0.7535217 |
| FWER p-Value | 1.0 |
Table: GSEA Results Summary

  

Fig 1: Enrichment plot: REACTOME\_METABOLISM\_OF\_STEROIDS      
 Profile of the Running ES Score & Positions of GeneSet Members on the Rank Ordered List

  

| SYMBOL | TITLE | RANK IN GENE LIST | RANK METRIC SCORE | RUNNING ES | CORE ENRICHMENT || 1 | 23054 | NCOA6 | 77 | 0.838 | 0.0565 | Yes |
| 2 | 403313 | PLPP6 | 274 | 0.615 | -0.0020 | Yes |
| 3 | 6715 | SRD5A1 | 325 | 0.578 | 0.0392 | Yes |
| 4 | 5465 | PPARA | 375 | 0.550 | 0.0774 | Yes |
| 5 | 1387 | CREBBP | 387 | 0.544 | 0.1421 | Yes |
| 6 | 6256 | RXRA | 439 | 0.509 | 0.1734 | Yes |
| 7 | 4047 | LSS | 640 | 0.405 | 0.0839 | Yes |
| 8 | 9453 | GGPS1 | 677 | 0.389 | 0.1099 | Yes |
| 9 | 31 | ACACA | 780 | 0.334 | 0.0812 | Yes |
| 10 | 39 | ACAT2 | 799 | 0.322 | 0.1112 | Yes |
| 11 | 5901 | RAN | 810 | 0.313 | 0.1457 | Yes |
| 12 | 1718 | DHCR24 | 832 | 0.293 | 0.1698 | Yes |
| 13 | 1717 | DHCR7 | 836 | 0.288 | 0.2060 | Yes |
| 14 | 2224 | FDPS | 841 | 0.273 | 0.2396 | Yes |
| 15 | 26031 | OSBPL3 | 870 | -0.281 | 0.2570 | Yes |
| 16 | 51141 | INSIG2 | 903 | -0.328 | 0.2778 | Yes |
| 17 | 10802 | SEC24A | 1251 | -0.639 | 0.1141 | No |
Table: GSEA details [plain text format]

  

Fig 2: REACTOME\_METABOLISM\_OF\_STEROIDS      
 Blue-Pink O' Gram in the Space of the Analyzed GeneSet

  

Fig 3: REACTOME\_METABOLISM\_OF\_STEROIDS: Random ES distribution      
 Gene set null distribution of ES for **REACTOME\_METABOLISM\_OF\_STEROIDS**

  
