# Supplementary material for: Role and mechanism of NCAPD3 in promoting malignant behaviors in gastric cancer
Source: Front Pharmacol. 2024 Apr 22;15:1341039. doi: 10.3389/fphar.2024.1341039 (PMC11070777; doi:10.3389/fphar.2024.1341039)
Supplement: Supplementary file 11 [file DataSheet2.ZIP › GSEA/Canonical pathways/my_analysis.Gsea.1599462267220/REACTOME_METABOLISM_OF_WATER_SOLUBLE_VITAMINS_AND_COFACTORS.html]

Details for gene set REACTOME\_METABOLISM\_OF\_WATER\_SOLUBLE\_VITAMINS\_AND\_COFACTORS[GSEA]

|  || Dataset | filtered\_dataset.sample\_info.cls#WT\_versus\_NCAPD3\_MUT |
| Phenotype | sample\_info.cls#WT\_versus\_NCAPD3\_MUT |
| Upregulated in class | WT |
| GeneSet | REACTOME\_METABOLISM\_OF\_WATER\_SOLUBLE\_VITAMINS\_AND\_COFACTORS |
| Enrichment Score (ES) | 0.2629014 |
| Normalized Enrichment Score (NES) | 1.0474937 |
| Nominal p-value | 0.3846154 |
| FDR q-value | 0.7740616 |
| FWER p-Value | 1.0 |
Table: GSEA Results Summary

  

Fig 1: Enrichment plot: REACTOME\_METABOLISM\_OF\_WATER\_SOLUBLE\_VITAMINS\_AND\_COFACTORS      
 Profile of the Running ES Score & Positions of GeneSet Members on the Rank Ordered List

  

| SYMBOL | TITLE | RANK IN GENE LIST | RANK METRIC SCORE | RUNNING ES | CORE ENRICHMENT || 1 | 53354 | PANK1 | 39 | 0.949 | 0.0759 | Yes |
| 2 | 27304 | MOCS3 | 76 | 0.844 | 0.1425 | Yes |
| 3 | 6573 | SLC19A1 | 231 | 0.650 | 0.1031 | Yes |
| 4 | 83666 | PARP9 | 258 | 0.624 | 0.1528 | Yes |
| 5 | 25974 | MMACHC | 265 | 0.621 | 0.2165 | Yes |
| 6 | 166785 | MMAA | 369 | 0.553 | 0.2032 | Yes |
| 7 | 326625 | MMAB | 371 | 0.552 | 0.2629 | Yes |
| 8 | 6470 | SHMT1 | 498 | 0.474 | 0.2244 | No |
| 9 | 4548 | MTR | 592 | 0.428 | 0.2046 | No |
| 10 | 64087 | MCCC2 | 747 | 0.352 | 0.1326 | No |
| 11 | 31 | ACACA | 780 | 0.334 | 0.1461 | No |
| 12 | 6515 | SLC2A3 | 873 | -0.283 | 0.1111 | No |
| 13 | 10797 | MTHFD2 | 911 | -0.338 | 0.1215 | No |
| 14 | 25902 | MTHFD1L | 986 | -0.395 | 0.1116 | No |
| 15 | 6947 | TCN1 | 1135 | -0.503 | 0.0605 | No |
| 16 | 9962 | SLC23A2 | 1169 | -0.537 | 0.0956 | No |
| 17 | 1719 | DHFR | 1298 | -0.700 | 0.0803 | No |
Table: GSEA details [plain text format]

  

Fig 2: REACTOME\_METABOLISM\_OF\_WATER\_SOLUBLE\_VITAMINS\_AND\_COFACTORS      
 Blue-Pink O' Gram in the Space of the Analyzed GeneSet

  

Fig 3: REACTOME\_METABOLISM\_OF\_WATER\_SOLUBLE\_VITAMINS\_AND\_COFACTORS: Random ES distribution      
 Gene set null distribution of ES for **REACTOME\_METABOLISM\_OF\_WATER\_SOLUBLE\_VITAMINS\_AND\_COFACTORS**

  
