# Supplementary material for: Role and mechanism of NCAPD3 in promoting malignant behaviors in gastric cancer
Source: Front Pharmacol. 2024 Apr 22;15:1341039. doi: 10.3389/fphar.2024.1341039 (PMC11070777; doi:10.3389/fphar.2024.1341039)
Supplement: Supplementary file 11 [file DataSheet2.ZIP › GSEA/Canonical pathways/my_analysis.Gsea.1599462267220/REACTOME_NEGATIVE_REGULATION_OF_THE_PI3K_AKT_NETWORK.html]

Details for gene set REACTOME\_NEGATIVE\_REGULATION\_OF\_THE\_PI3K\_AKT\_NETWORK[GSEA]

|  || Dataset | filtered\_dataset.sample\_info.cls#WT\_versus\_NCAPD3\_MUT |
| Phenotype | sample\_info.cls#WT\_versus\_NCAPD3\_MUT |
| Upregulated in class | NCAPD3\_MUT |
| GeneSet | REACTOME\_NEGATIVE\_REGULATION\_OF\_THE\_PI3K\_AKT\_NETWORK |
| Enrichment Score (ES) | -0.35922146 |
| Normalized Enrichment Score (NES) | -1.5585004 |
| Nominal p-value | 0.05955335 |
| FDR q-value | 0.1521877 |
| FWER p-Value | 0.828 |
Table: GSEA Results Summary

  

Fig 1: Enrichment plot: REACTOME\_NEGATIVE\_REGULATION\_OF\_THE\_PI3K\_AKT\_NETWORK      
 Profile of the Running ES Score & Positions of GeneSet Members on the Rank Ordered List

  

| SYMBOL | TITLE | RANK IN GENE LIST | RANK METRIC SCORE | RUNNING ES | CORE ENRICHMENT || 1 | 117145 | THEM4 | 65 | 0.860 | 0.0273 | No |
| 2 | 3654 | IRAK1 | 69 | 0.856 | 0.0987 | No |
| 3 | 3667 | IRS1 | 83 | 0.821 | 0.1599 | No |
| 4 | 51135 | IRAK4 | 245 | 0.635 | 0.0990 | No |
| 5 | 5728 | PTEN | 285 | 0.606 | 0.1231 | No |
| 6 | 10818 | FRS2 | 363 | 0.558 | 0.1159 | No |
| 7 | 5295 | PIK3R1 | 467 | 0.493 | 0.0844 | No |
| 8 | 5305 | PIP4K2A | 1033 | -0.432 | -0.2838 | No |
| 9 | 3556 | IL1RAP | 1099 | -0.476 | -0.2895 | No |
| 10 | 5154 | PDGFA | 1191 | -0.566 | -0.3061 | Yes |
| 11 | 1956 | EGFR | 1266 | -0.657 | -0.3027 | Yes |
| 12 | 57761 | TRIB3 | 1292 | -0.685 | -0.2617 | Yes |
| 13 | 3084 | NRG1 | 1320 | -0.739 | -0.2176 | Yes |
| 14 | 90865 | IL33 | 1325 | -0.745 | -0.1564 | Yes |
| 15 | 1839 | HBEGF | 1328 | -0.751 | -0.0933 | Yes |
| 16 | 4233 | MET | 1358 | -0.821 | -0.0435 | Yes |
| 17 | 5156 | PDGFRA | 1383 | -0.932 | 0.0194 | Yes |
Table: GSEA details [plain text format]

  

Fig 2: REACTOME\_NEGATIVE\_REGULATION\_OF\_THE\_PI3K\_AKT\_NETWORK      
 Blue-Pink O' Gram in the Space of the Analyzed GeneSet

  

Fig 3: REACTOME\_NEGATIVE\_REGULATION\_OF\_THE\_PI3K\_AKT\_NETWORK: Random ES distribution      
 Gene set null distribution of ES for **REACTOME\_NEGATIVE\_REGULATION\_OF\_THE\_PI3K\_AKT\_NETWORK**

  
