# Supplementary material for: Role and mechanism of NCAPD3 in promoting malignant behaviors in gastric cancer
Source: Front Pharmacol. 2024 Apr 22;15:1341039. doi: 10.3389/fphar.2024.1341039 (PMC11070777; doi:10.3389/fphar.2024.1341039)
Supplement: Supplementary file 11 [file DataSheet2.ZIP › GSEA/Canonical pathways/my_analysis.Gsea.1599462267220/REACTOME_NERVOUS_SYSTEM_DEVELOPMENT.html]

Details for gene set REACTOME\_NERVOUS\_SYSTEM\_DEVELOPMENT[GSEA]

|  || Dataset | filtered\_dataset.sample\_info.cls#WT\_versus\_NCAPD3\_MUT |
| Phenotype | sample\_info.cls#WT\_versus\_NCAPD3\_MUT |
| Upregulated in class | NCAPD3\_MUT |
| GeneSet | REACTOME\_NERVOUS\_SYSTEM\_DEVELOPMENT |
| Enrichment Score (ES) | -0.26425654 |
| Normalized Enrichment Score (NES) | -1.4889591 |
| Nominal p-value | 0.065162905 |
| FDR q-value | 0.19228567 |
| FWER p-Value | 0.914 |
Table: GSEA Results Summary

  

Fig 1: Enrichment plot: REACTOME\_NERVOUS\_SYSTEM\_DEVELOPMENT      
 Profile of the Running ES Score & Positions of GeneSet Members on the Rank Ordered List

  

| SYMBOL | TITLE | RANK IN GENE LIST | RANK METRIC SCORE | RUNNING ES | CORE ENRICHMENT || 1 | 2935 | GSPT1 | 5 | 1.156 | 0.0538 | No |
| 2 | 23380 | SRGAP2 | 142 | 0.757 | -0.0076 | No |
| 3 | 6191 | RPS4X | 196 | 0.682 | -0.0123 | No |
| 4 | 5701 | PSMC2 | 244 | 0.636 | -0.0149 | No |
| 5 | 7220 | TRPC1 | 328 | 0.576 | -0.0466 | No |
| 6 | 10818 | FRS2 | 363 | 0.558 | -0.0436 | No |
| 7 | 5718 | PSMD12 | 372 | 0.552 | -0.0220 | No |
| 8 | 5295 | PIK3R1 | 467 | 0.493 | -0.0659 | No |
| 9 | 1793 | DOCK1 | 489 | 0.480 | -0.0574 | No |
| 10 | 5567 | PRKACB | 595 | 0.426 | -0.1126 | No |
| 11 | 6208 | RPS14 | 604 | 0.421 | -0.0975 | No |
| 12 | 6197 | RPS6KA3 | 643 | 0.403 | -0.1051 | No |
| 13 | 9037 | SEMA5A | 688 | 0.385 | -0.1180 | No |
| 14 | 2932 | GSK3B | 714 | 0.367 | -0.1180 | No |
| 15 | 23767 | FLRT3 | 781 | 0.333 | -0.1495 | No |
| 16 | 1605 | DAG1 | 823 | 0.304 | -0.1642 | No |
| 17 | 2909 | ARHGAP35 | 844 | 0.259 | -0.1659 | No |
| 18 | 3312 | HSPA8 | 848 | 0.244 | -0.1559 | No |
| 19 | 3673 | ITGA2 | 858 | -0.264 | -0.1494 | No |
| 20 | 6237 | RRAS | 869 | -0.280 | -0.1427 | No |
| 21 | 5621 | PRNP | 970 | -0.387 | -0.1963 | No |
| 22 | 7277 | TUBA4A | 997 | -0.404 | -0.1951 | No |
| 23 | 9939 | RBM8A | 1093 | -0.474 | -0.2407 | Yes |
| 24 | 6709 | SPTAN1 | 1114 | -0.487 | -0.2311 | Yes |
| 25 | 7204 | TRIO | 1120 | -0.494 | -0.2101 | Yes |
| 26 | 7074 | TIAM1 | 1143 | -0.508 | -0.2009 | Yes |
| 27 | 1282 | COL4A1 | 1165 | -0.534 | -0.1896 | Yes |
| 28 | 4651 | MYO10 | 1213 | -0.588 | -0.1946 | Yes |
| 29 | 27 | ABL2 | 1218 | -0.592 | -0.1680 | Yes |
| 30 | 2043 | EPHA4 | 1228 | -0.602 | -0.1446 | Yes |
| 31 | 2887 | GRB10 | 1259 | -0.646 | -0.1344 | Yes |
| 32 | 1956 | EGFR | 1266 | -0.657 | -0.1060 | Yes |
| 33 | 286 | ANK1 | 1284 | -0.672 | -0.0850 | Yes |
| 34 | 23365 | ARHGEF12 | 1329 | -0.751 | -0.0797 | Yes |
| 35 | 4233 | MET | 1358 | -0.821 | -0.0593 | Yes |
| 36 | 6711 | SPTBN1 | 1381 | -0.925 | -0.0293 | Yes |
| 37 | 59277 | NTN4 | 1390 | -0.999 | 0.0146 | Yes |
Table: GSEA details [plain text format]

  

Fig 2: REACTOME\_NERVOUS\_SYSTEM\_DEVELOPMENT      
 Blue-Pink O' Gram in the Space of the Analyzed GeneSet

  

Fig 3: REACTOME\_NERVOUS\_SYSTEM\_DEVELOPMENT: Random ES distribution      
 Gene set null distribution of ES for **REACTOME\_NERVOUS\_SYSTEM\_DEVELOPMENT**

  
