# Supplementary material for: Role and mechanism of NCAPD3 in promoting malignant behaviors in gastric cancer
Source: Front Pharmacol. 2024 Apr 22;15:1341039. doi: 10.3389/fphar.2024.1341039 (PMC11070777; doi:10.3389/fphar.2024.1341039)
Supplement: Supplementary file 11 [file DataSheet2.ZIP › GSEA/Canonical pathways/my_analysis.Gsea.1599462267220/REACTOME_ORGANELLE_BIOGENESIS_AND_MAINTENANCE.html]

Details for gene set REACTOME\_ORGANELLE\_BIOGENESIS\_AND\_MAINTENANCE[GSEA]

|  || Dataset | filtered\_dataset.sample\_info.cls#WT\_versus\_NCAPD3\_MUT |
| Phenotype | sample\_info.cls#WT\_versus\_NCAPD3\_MUT |
| Upregulated in class | WT |
| GeneSet | REACTOME\_ORGANELLE\_BIOGENESIS\_AND\_MAINTENANCE |
| Enrichment Score (ES) | 0.2589974 |
| Normalized Enrichment Score (NES) | 1.2154381 |
| Nominal p-value | 0.23228996 |
| FDR q-value | 0.76119894 |
| FWER p-Value | 1.0 |
Table: GSEA Results Summary

  

Fig 1: Enrichment plot: REACTOME\_ORGANELLE\_BIOGENESIS\_AND\_MAINTENANCE      
 Profile of the Running ES Score & Positions of GeneSet Members on the Rank Ordered List

  

| SYMBOL | TITLE | RANK IN GENE LIST | RANK METRIC SCORE | RUNNING ES | CORE ENRICHMENT || 1 | 51134 | CEP83 | 19 | 1.038 | 0.0578 | Yes |
| 2 | 132320 | SCLT1 | 56 | 0.902 | 0.0939 | Yes |
| 3 | 23322 | RPGRIP1L | 60 | 0.871 | 0.1518 | Yes |
| 4 | 23054 | NCOA6 | 77 | 0.838 | 0.1979 | Yes |
| 5 | 22948 | CCT5 | 215 | 0.663 | 0.1445 | Yes |
| 6 | 54801 | HAUS6 | 219 | 0.660 | 0.1879 | Yes |
| 7 | 5465 | PPARA | 375 | 0.550 | 0.1137 | Yes |
| 8 | 1387 | CREBBP | 387 | 0.544 | 0.1432 | Yes |
| 9 | 801 | CALM1 | 424 | 0.518 | 0.1529 | Yes |
| 10 | 6256 | RXRA | 439 | 0.509 | 0.1778 | Yes |
| 11 | 6742 | SSBP1 | 501 | 0.472 | 0.1662 | Yes |
| 12 | 56652 | C10orf2 | 531 | 0.457 | 0.1767 | Yes |
| 13 | 8195 | MKKS | 542 | 0.449 | 0.2005 | Yes |
| 14 | 55125 | CEP192 | 568 | 0.438 | 0.2126 | Yes |
| 15 | 26123 | TCTN3 | 603 | 0.421 | 0.2170 | Yes |
| 16 | 5147 | PDE6D | 624 | 0.410 | 0.2308 | Yes |
| 17 | 55735 | DNAJC11 | 625 | 0.409 | 0.2590 | Yes |
| 18 | 5311 | PKD2 | 708 | 0.372 | 0.2253 | No |
| 19 | 203068 | TUBB | 736 | 0.358 | 0.2305 | No |
| 20 | 516 | ATP5G1 | 826 | 0.301 | 0.1869 | No |
| 21 | 2747 | GLUD2 | 853 | -0.255 | 0.1857 | No |
| 22 | 2746 | GLUD1 | 854 | -0.255 | 0.2032 | No |
| 23 | 4209 | MEF2D | 910 | -0.337 | 0.1867 | No |
| 24 | 9572 | NR1D1 | 977 | -0.392 | 0.1660 | No |
| 25 | 7277 | TUBA4A | 997 | -0.404 | 0.1801 | No |
| 26 | 5565 | PRKAB2 | 1094 | -0.474 | 0.1433 | No |
| 27 | 146057 | TTBK2 | 1148 | -0.513 | 0.1403 | No |
| 28 | 64854 | USP46 | 1300 | -0.702 | 0.0795 | No |
Table: GSEA details [plain text format]

  

Fig 2: REACTOME\_ORGANELLE\_BIOGENESIS\_AND\_MAINTENANCE      
 Blue-Pink O' Gram in the Space of the Analyzed GeneSet

  

Fig 3: REACTOME\_ORGANELLE\_BIOGENESIS\_AND\_MAINTENANCE: Random ES distribution      
 Gene set null distribution of ES for **REACTOME\_ORGANELLE\_BIOGENESIS\_AND\_MAINTENANCE**

  
