# Supplementary material for: Role and mechanism of NCAPD3 in promoting malignant behaviors in gastric cancer
Source: Front Pharmacol. 2024 Apr 22;15:1341039. doi: 10.3389/fphar.2024.1341039 (PMC11070777; doi:10.3389/fphar.2024.1341039)
Supplement: Supplementary file 11 [file DataSheet2.ZIP › GSEA/Canonical pathways/my_analysis.Gsea.1599462267220/REACTOME_PROCESSING_OF_CAPPED_INTRON_CONTAINING_PRE_MRNA.html]

Details for gene set REACTOME\_PROCESSING\_OF\_CAPPED\_INTRON\_CONTAINING\_PRE\_MRNA[GSEA]

|  || Dataset | filtered\_dataset.sample\_info.cls#WT\_versus\_NCAPD3\_MUT |
| Phenotype | sample\_info.cls#WT\_versus\_NCAPD3\_MUT |
| Upregulated in class | WT |
| GeneSet | REACTOME\_PROCESSING\_OF\_CAPPED\_INTRON\_CONTAINING\_PRE\_MRNA |
| Enrichment Score (ES) | 0.32698134 |
| Normalized Enrichment Score (NES) | 1.5444375 |
| Nominal p-value | 0.057921637 |
| FDR q-value | 0.37443072 |
| FWER p-Value | 0.943 |
Table: GSEA Results Summary

  

Fig 1: Enrichment plot: REACTOME\_PROCESSING\_OF\_CAPPED\_INTRON\_CONTAINING\_PRE\_MRNA      
 Profile of the Running ES Score & Positions of GeneSet Members on the Rank Ordered List

  

| SYMBOL | TITLE | RANK IN GENE LIST | RANK METRIC SCORE | RUNNING ES | CORE ENRICHMENT || 1 | 51585 | PCF11 | 3 | 1.212 | 0.0895 | Yes |
| 2 | 6428 | SRSF3 | 55 | 0.907 | 0.1212 | Yes |
| 3 | 23450 | SF3B3 | 178 | 0.697 | 0.0858 | Yes |
| 4 | 55599 | RNPC3 | 227 | 0.652 | 0.1004 | Yes |
| 5 | 6426 | SRSF1 | 243 | 0.637 | 0.1377 | Yes |
| 6 | 5356 | PLRG1 | 338 | 0.573 | 0.1131 | Yes |
| 7 | 10212 | DDX39A | 389 | 0.543 | 0.1181 | Yes |
| 8 | 10291 | SF3A1 | 466 | 0.493 | 0.1005 | Yes |
| 9 | 1479 | CSTF3 | 500 | 0.474 | 0.1124 | Yes |
| 10 | 3184 | HNRNPD | 508 | 0.470 | 0.1429 | Yes |
| 11 | 1660 | DHX9 | 510 | 0.469 | 0.1776 | Yes |
| 12 | 55339 | WDR33 | 551 | 0.445 | 0.1824 | Yes |
| 13 | 9416 | DDX23 | 553 | 0.445 | 0.2153 | Yes |
| 14 | 6632 | SNRPD1 | 563 | 0.440 | 0.2421 | Yes |
| 15 | 8021 | NUP214 | 611 | 0.417 | 0.2397 | Yes |
| 16 | 56339 | METTL3 | 613 | 0.417 | 0.2705 | Yes |
| 17 | 6427 | SRSF2 | 679 | 0.388 | 0.2528 | Yes |
| 18 | 23350 | U2SURP | 719 | 0.364 | 0.2522 | Yes |
| 19 | 27316 | RBMX | 762 | 0.342 | 0.2477 | Yes |
| 20 | 3188 | HNRNPH2 | 769 | 0.338 | 0.2688 | Yes |
| 21 | 4670 | HNRNPM | 790 | 0.330 | 0.2794 | Yes |
| 22 | 9343 | EFTUD2 | 818 | 0.305 | 0.2829 | Yes |
| 23 | 26121 | PRPF31 | 825 | 0.301 | 0.3013 | Yes |
| 24 | 11157 | LSM6 | 830 | 0.296 | 0.3208 | Yes |
| 25 | 3312 | HSPA8 | 848 | 0.244 | 0.3270 | Yes |
| 26 | 9939 | RBM8A | 1093 | -0.474 | 0.1865 | No |
| 27 | 6430 | SRSF5 | 1183 | -0.553 | 0.1640 | No |
Table: GSEA details [plain text format]

  

Fig 2: REACTOME\_PROCESSING\_OF\_CAPPED\_INTRON\_CONTAINING\_PRE\_MRNA      
 Blue-Pink O' Gram in the Space of the Analyzed GeneSet

  

Fig 3: REACTOME\_PROCESSING\_OF\_CAPPED\_INTRON\_CONTAINING\_PRE\_MRNA: Random ES distribution      
 Gene set null distribution of ES for **REACTOME\_PROCESSING\_OF\_CAPPED\_INTRON\_CONTAINING\_PRE\_MRNA**

  
