# Supplementary material for: Role and mechanism of NCAPD3 in promoting malignant behaviors in gastric cancer
Source: Front Pharmacol. 2024 Apr 22;15:1341039. doi: 10.3389/fphar.2024.1341039 (PMC11070777; doi:10.3389/fphar.2024.1341039)
Supplement: Supplementary file 11 [file DataSheet2.ZIP › GSEA/Canonical pathways/my_analysis.Gsea.1599462267220/REACTOME_PROGRAMMED_CELL_DEATH.html]

Details for gene set REACTOME\_PROGRAMMED\_CELL\_DEATH[GSEA]

|  || Dataset | filtered\_dataset.sample\_info.cls#WT\_versus\_NCAPD3\_MUT |
| Phenotype | sample\_info.cls#WT\_versus\_NCAPD3\_MUT |
| Upregulated in class | WT |
| GeneSet | REACTOME\_PROGRAMMED\_CELL\_DEATH |
| Enrichment Score (ES) | 0.2672559 |
| Normalized Enrichment Score (NES) | 1.118382 |
| Nominal p-value | 0.31452993 |
| FDR q-value | 0.7836479 |
| FWER p-Value | 1.0 |
Table: GSEA Results Summary

  

Fig 1: Enrichment plot: REACTOME\_PROGRAMMED\_CELL\_DEATH      
 Profile of the Running ES Score & Positions of GeneSet Members on the Rank Ordered List

  

| SYMBOL | TITLE | RANK IN GENE LIST | RANK METRIC SCORE | RUNNING ES | CORE ENRICHMENT || 1 | 115209 | OMA1 | 45 | 0.932 | 0.0623 | Yes |
| 2 | 4836 | NMT1 | 208 | 0.669 | 0.0137 | Yes |
| 3 | 5701 | PSMC2 | 244 | 0.636 | 0.0530 | Yes |
| 4 | 353376 | TICAM2 | 271 | 0.617 | 0.0969 | Yes |
| 5 | 324 | APC | 281 | 0.607 | 0.1521 | Yes |
| 6 | 197259 | MLKL | 346 | 0.569 | 0.1639 | Yes |
| 7 | 5718 | PSMD12 | 372 | 0.552 | 0.2019 | Yes |
| 8 | 637 | BID | 478 | 0.487 | 0.1758 | Yes |
| 9 | 8737 | RIPK1 | 493 | 0.477 | 0.2141 | Yes |
| 10 | 7099 | TLR4 | 541 | 0.451 | 0.2261 | Yes |
| 11 | 3836 | KPNA1 | 548 | 0.448 | 0.2673 | Yes |
| 12 | 8772 | FADD | 742 | 0.355 | 0.1645 | No |
| 13 | 839 | CASP6 | 792 | 0.330 | 0.1627 | No |
| 14 | 3146 | HMGB1 | 831 | 0.295 | 0.1654 | No |
| 15 | 8837 | CFLAR | 865 | -0.278 | 0.1699 | No |
| 16 | 3005 | H1F0 | 880 | -0.294 | 0.1897 | No |
| 17 | 9414 | TJP2 | 916 | -0.345 | 0.1996 | No |
| 18 | 10018 | BCL2L11 | 1027 | -0.425 | 0.1636 | No |
| 19 | 6709 | SPTAN1 | 1114 | -0.487 | 0.1512 | No |
| 20 | 330 | BIRC3 | 1224 | -0.600 | 0.1337 | No |
Table: GSEA details [plain text format]

  

Fig 2: REACTOME\_PROGRAMMED\_CELL\_DEATH      
 Blue-Pink O' Gram in the Space of the Analyzed GeneSet

  

Fig 3: REACTOME\_PROGRAMMED\_CELL\_DEATH: Random ES distribution      
 Gene set null distribution of ES for **REACTOME\_PROGRAMMED\_CELL\_DEATH**

  
