# Supplementary material for: Role and mechanism of NCAPD3 in promoting malignant behaviors in gastric cancer
Source: Front Pharmacol. 2024 Apr 22;15:1341039. doi: 10.3389/fphar.2024.1341039 (PMC11070777; doi:10.3389/fphar.2024.1341039)
Supplement: Supplementary file 11 [file DataSheet2.ZIP › GSEA/Canonical pathways/my_analysis.Gsea.1599462267220/REACTOME_RNA_POLYMERASE_II_TRANSCRIPTION.html]

Details for gene set REACTOME\_RNA\_POLYMERASE\_II\_TRANSCRIPTION[GSEA]

|  || Dataset | filtered\_dataset.sample\_info.cls#WT\_versus\_NCAPD3\_MUT |
| Phenotype | sample\_info.cls#WT\_versus\_NCAPD3\_MUT |
| Upregulated in class | WT |
| GeneSet | REACTOME\_RNA\_POLYMERASE\_II\_TRANSCRIPTION |
| Enrichment Score (ES) | 0.2309288 |
| Normalized Enrichment Score (NES) | 1.7018334 |
| Nominal p-value | 0.004322767 |
| FDR q-value | 0.25392768 |
| FWER p-Value | 0.686 |
Table: GSEA Results Summary

  

Fig 1: Enrichment plot: REACTOME\_RNA\_POLYMERASE\_II\_TRANSCRIPTION      
 Profile of the Running ES Score & Positions of GeneSet Members on the Rank Ordered List

  

| SYMBOL | TITLE | RANK IN GENE LIST | RANK METRIC SCORE | RUNNING ES | CORE ENRICHMENT || 1 | 51585 | PCF11 | 3 | 1.212 | 0.0132 | Yes |
| 2 | 7571 | ZNF23 | 25 | 1.018 | 0.0098 | Yes |
| 3 | 55170 | PRMT6 | 32 | 0.981 | 0.0176 | Yes |
| 4 | 23248 | RPRD2 | 33 | 0.977 | 0.0302 | Yes |
| 5 | 57103 | TIGAR | 37 | 0.963 | 0.0402 | Yes |
| 6 | 9759 | HDAC4 | 46 | 0.925 | 0.0457 | Yes |
| 7 | 388536 | ZNF790 | 52 | 0.913 | 0.0535 | Yes |
| 8 | 6428 | SRSF3 | 55 | 0.907 | 0.0636 | Yes |
| 9 | 3654 | IRAK1 | 69 | 0.856 | 0.0644 | Yes |
| 10 | 4204 | MECP2 | 70 | 0.856 | 0.0753 | Yes |
| 11 | 7743 | ZNF189 | 74 | 0.851 | 0.0839 | Yes |
| 12 | 7697 | ZNF138 | 80 | 0.827 | 0.0905 | Yes |
| 13 | 353088 | ZNF429 | 81 | 0.824 | 0.1011 | Yes |
| 14 | 7769 | ZNF226 | 125 | 0.777 | 0.0774 | Yes |
| 15 | 79109 | MAPKAP1 | 130 | 0.770 | 0.0841 | Yes |
| 16 | 4193 | MDM2 | 131 | 0.769 | 0.0939 | Yes |
| 17 | 390927 | ZNF793 | 137 | 0.758 | 0.0997 | Yes |
| 18 | 55900 | ZNF302 | 145 | 0.740 | 0.1037 | Yes |
| 19 | 7733 | ZNF180 | 154 | 0.728 | 0.1068 | Yes |
| 20 | 162967 | ZNF320 | 156 | 0.726 | 0.1153 | Yes |
| 21 | 7248 | TSC1 | 158 | 0.718 | 0.1237 | Yes |
| 22 | 817 | CAMK2D | 165 | 0.710 | 0.1281 | Yes |
| 23 | 51434 | ANAPC7 | 166 | 0.710 | 0.1372 | Yes |
| 24 | 10795 | ZNF268 | 168 | 0.710 | 0.1455 | Yes |
| 25 | 55240 | STEAP3 | 169 | 0.710 | 0.1546 | Yes |
| 26 | 54971 | BANP | 172 | 0.707 | 0.1621 | Yes |
| 27 | 84911 | ZNF382 | 182 | 0.692 | 0.1639 | Yes |
| 28 | 339559 | ZFP69 | 188 | 0.686 | 0.1688 | Yes |
| 29 | 7562 | ZNF708 | 190 | 0.686 | 0.1768 | Yes |
| 30 | 54815 | GATAD2A | 191 | 0.685 | 0.1856 | Yes |
| 31 | 79230 | ZNF557 | 201 | 0.675 | 0.1872 | Yes |
| 32 | 79664 | ICE2 | 217 | 0.662 | 0.1839 | Yes |
| 33 | 57521 | RPTOR | 239 | 0.645 | 0.1757 | Yes |
| 34 | 6426 | SRSF1 | 243 | 0.637 | 0.1815 | Yes |
| 35 | 5701 | PSMC2 | 244 | 0.636 | 0.1897 | Yes |
| 36 | 55756 | INTS9 | 257 | 0.624 | 0.1883 | Yes |
| 37 | 92595 | ZNF764 | 273 | 0.616 | 0.1844 | Yes |
| 38 | 84874 | ZNF514 | 283 | 0.606 | 0.1851 | Yes |
| 39 | 5728 | PTEN | 285 | 0.606 | 0.1921 | Yes |
| 40 | 7626 | ZNF75D | 309 | 0.588 | 0.1816 | Yes |
| 41 | 147929 | ZNF565 | 311 | 0.587 | 0.1884 | Yes |
| 42 | 7565 | ZNF17 | 316 | 0.585 | 0.1927 | Yes |
| 43 | 1021 | CDK6 | 324 | 0.579 | 0.1946 | Yes |
| 44 | 4297 | KMT2A | 337 | 0.573 | 0.1926 | Yes |
| 45 | 23660 | ZKSCAN5 | 345 | 0.570 | 0.1944 | Yes |
| 46 | 55869 | HDAC8 | 352 | 0.566 | 0.1970 | Yes |
| 47 | 2965 | GTF2H1 | 356 | 0.563 | 0.2018 | Yes |
| 48 | 168374 | ZNF92 | 357 | 0.562 | 0.2090 | Yes |
| 49 | 5810 | RAD1 | 360 | 0.560 | 0.2146 | Yes |
| 50 | 5718 | PSMD12 | 372 | 0.552 | 0.2131 | Yes |
| 51 | 115509 | ZNF689 | 374 | 0.551 | 0.2194 | Yes |
| 52 | 5465 | PPARA | 375 | 0.550 | 0.2264 | Yes |
| 53 | 1387 | CREBBP | 387 | 0.544 | 0.2248 | Yes |
| 54 | 10212 | DDX39A | 389 | 0.543 | 0.2309 | Yes |
| 55 | 126231 | ZNF573 | 418 | 0.523 | 0.2157 | No |
| 56 | 801 | CALM1 | 424 | 0.518 | 0.2184 | No |
| 57 | 6256 | RXRA | 439 | 0.509 | 0.2140 | No |
| 58 | 79047 | KCTD15 | 469 | 0.493 | 0.1975 | No |
| 59 | 637 | BID | 478 | 0.487 | 0.1975 | No |
| 60 | 80095 | ZNF606 | 479 | 0.487 | 0.2038 | No |
| 61 | 23468 | CBX5 | 484 | 0.485 | 0.2068 | No |
| 62 | 8454 | CUL1 | 492 | 0.477 | 0.2075 | No |
| 63 | 29101 | SSU72 | 496 | 0.475 | 0.2112 | No |
| 64 | 58500 | ZNF250 | 499 | 0.474 | 0.2157 | No |
| 65 | 1479 | CSTF3 | 500 | 0.474 | 0.2218 | No |
| 66 | 7753 | ZNF202 | 514 | 0.466 | 0.2176 | No |
| 67 | 4091 | SMAD6 | 519 | 0.462 | 0.2203 | No |
| 68 | 7627 | ZNF75A | 527 | 0.459 | 0.2207 | No |
| 69 | 55339 | WDR33 | 551 | 0.445 | 0.2084 | No |
| 70 | 53615 | MBD3 | 561 | 0.441 | 0.2070 | No |
| 71 | 84775 | ZNF607 | 564 | 0.440 | 0.2111 | No |
| 72 | 93134 | ZNF561 | 590 | 0.431 | 0.1970 | No |
| 73 | 79027 | ZNF655 | 594 | 0.427 | 0.2001 | No |
| 74 | 1108 | CHD4 | 627 | 0.408 | 0.1803 | No |
| 75 | 80110 | ZNF614 | 642 | 0.404 | 0.1745 | No |
| 76 | 7587 | ZNF37A | 667 | 0.394 | 0.1607 | No |
| 77 | 6427 | SRSF2 | 679 | 0.388 | 0.1571 | No |
| 78 | 28996 | HIPK2 | 687 | 0.385 | 0.1565 | No |
| 79 | 2932 | GSK3B | 714 | 0.367 | 0.1409 | No |
| 80 | 122809 | SOCS4 | 726 | 0.360 | 0.1369 | No |
| 81 | 6605 | SMARCE1 | 749 | 0.349 | 0.1241 | No |
| 82 | 6241 | RRM2 | 755 | 0.346 | 0.1246 | No |
| 83 | 27300 | ZNF544 | 759 | 0.343 | 0.1266 | No |
| 84 | 4869 | NPM1 | 772 | 0.336 | 0.1215 | No |
| 85 | 839 | CASP6 | 792 | 0.330 | 0.1109 | No |
| 86 | 11091 | WDR5 | 822 | 0.304 | 0.0920 | No |
| 87 | 1051 | CEBPB | 862 | -0.274 | 0.0650 | No |
| 88 | 6515 | SLC2A3 | 873 | -0.283 | 0.0608 | No |
| 89 | 7296 | TXNRD1 | 877 | -0.289 | 0.0621 | No |
| 90 | 694 | BTG1 | 901 | -0.326 | 0.0483 | No |
| 91 | 3164 | NR4A1 | 912 | -0.341 | 0.0448 | No |
| 92 | 8738 | CRADD | 922 | -0.349 | 0.0422 | No |
| 93 | 4088 | SMAD3 | 930 | -0.353 | 0.0413 | No |
| 94 | 90874 | ZNF697 | 933 | -0.357 | 0.0443 | No |
| 95 | 8881 | CDC16 | 963 | -0.383 | 0.0265 | No |
| 96 | 9314 | KLF4 | 972 | -0.389 | 0.0252 | No |
| 97 | 7067 | THRA | 976 | -0.392 | 0.0278 | No |
| 98 | 9572 | NR1D1 | 977 | -0.392 | 0.0329 | No |
| 99 | 10793 | ZNF273 | 980 | -0.394 | 0.0363 | No |
| 100 | 901 | CCNG2 | 998 | -0.405 | 0.0282 | No |
| 101 | 2146 | EZH2 | 1001 | -0.406 | 0.0318 | No |
| 102 | 51343 | FZR1 | 1005 | -0.407 | 0.0347 | No |
| 103 | 3516 | RBPJ | 1021 | -0.419 | 0.0283 | No |
| 104 | 10018 | BCL2L11 | 1027 | -0.425 | 0.0299 | No |
| 105 | 5305 | PIP4K2A | 1033 | -0.432 | 0.0315 | No |
| 106 | 5111 | PCNA | 1077 | -0.460 | 0.0037 | No |
| 107 | 4773 | NFATC2 | 1085 | -0.465 | 0.0041 | No |
| 108 | 9939 | RBM8A | 1093 | -0.474 | 0.0047 | No |
| 109 | 5565 | PRKAB2 | 1094 | -0.474 | 0.0108 | No |
| 110 | 857 | CAV1 | 1098 | -0.475 | 0.0145 | No |
| 111 | 26053 | AUTS2 | 1113 | -0.485 | 0.0098 | No |
| 112 | 4194 | MDM4 | 1117 | -0.492 | 0.0137 | No |
| 113 | 54541 | DDIT4 | 1133 | -0.502 | 0.0084 | No |
| 114 | 1263 | PLK3 | 1134 | -0.502 | 0.0148 | No |
| 115 | 604 | BCL6 | 1136 | -0.504 | 0.0205 | No |
| 116 | 639 | PRDM1 | 1159 | -0.523 | 0.0100 | No |
| 117 | 6430 | SRSF5 | 1183 | -0.553 | -0.0009 | No |
| 118 | 8535 | CBX4 | 1184 | -0.555 | 0.0062 | No |
| 119 | 665 | BNIP3L | 1185 | -0.556 | 0.0133 | No |
| 120 | 10001 | MED6 | 1190 | -0.561 | 0.0173 | No |
| 121 | 472 | ATM | 1193 | -0.568 | 0.0231 | No |
| 122 | 8345 | HIST1H2BH | 1207 | -0.585 | 0.0204 | No |
| 123 | 9975 | NR1D2 | 1230 | -0.605 | 0.0109 | No |
| 124 | 7057 | THBS1 | 1242 | -0.620 | 0.0102 | No |
| 125 | 114907 | FBXO32 | 1248 | -0.625 | 0.0143 | No |
| 126 | 1956 | EGFR | 1266 | -0.657 | 0.0094 | No |
| 127 | 7994 | KAT6A | 1279 | -0.666 | 0.0085 | No |
| 128 | 861 | RUNX1 | 1293 | -0.687 | 0.0071 | No |
| 129 | 1649 | DDIT3 | 1341 | -0.779 | -0.0197 | No |
| 130 | 7422 | VEGFA | 1346 | -0.788 | -0.0128 | No |
| 131 | 133746 | JMY | 1350 | -0.793 | -0.0049 | No |
| 132 | 4233 | MET | 1358 | -0.821 | 0.0001 | No |
| 133 | 22936 | ELL2 | 1370 | -0.882 | 0.0028 | No |
| 134 | 10397 | NDRG1 | 1380 | -0.925 | 0.0076 | No |
| 135 | 3329 | HSPD1 | 1402 | -1.183 | 0.0063 | No |
Table: GSEA details [plain text format]

  

Fig 2: REACTOME\_RNA\_POLYMERASE\_II\_TRANSCRIPTION      
 Blue-Pink O' Gram in the Space of the Analyzed GeneSet

  

Fig 3: REACTOME\_RNA\_POLYMERASE\_II\_TRANSCRIPTION: Random ES distribution      
 Gene set null distribution of ES for **REACTOME\_RNA\_POLYMERASE\_II\_TRANSCRIPTION**

  
