# Supplementary material for: Role and mechanism of NCAPD3 in promoting malignant behaviors in gastric cancer
Source: Front Pharmacol. 2024 Apr 22;15:1341039. doi: 10.3389/fphar.2024.1341039 (PMC11070777; doi:10.3389/fphar.2024.1341039)
Supplement: Supplementary file 11 [file DataSheet2.ZIP › GSEA/Canonical pathways/my_analysis.Gsea.1599462267220/REACTOME_RRNA_PROCESSING.html]

Details for gene set REACTOME\_RRNA\_PROCESSING[GSEA]

|  || Dataset | filtered\_dataset.sample\_info.cls#WT\_versus\_NCAPD3\_MUT |
| Phenotype | sample\_info.cls#WT\_versus\_NCAPD3\_MUT |
| Upregulated in class | WT |
| GeneSet | REACTOME\_RRNA\_PROCESSING |
| Enrichment Score (ES) | 0.3820613 |
| Normalized Enrichment Score (NES) | 1.8541355 |
| Nominal p-value | 0.0017152659 |
| FDR q-value | 0.1314948 |
| FWER p-Value | 0.332 |
Table: GSEA Results Summary

  

Fig 1: Enrichment plot: REACTOME\_RRNA\_PROCESSING      
 Profile of the Running ES Score & Positions of GeneSet Members on the Rank Ordered List

  

| SYMBOL | TITLE | RANK IN GENE LIST | RANK METRIC SCORE | RUNNING ES | CORE ENRICHMENT || 1 | 8568 | RRP1 | 22 | 1.027 | 0.0610 | Yes |
| 2 | 55781 | RIOK2 | 135 | 0.763 | 0.0371 | Yes |
| 3 | 6191 | RPS4X | 196 | 0.682 | 0.0448 | Yes |
| 4 | 79922 | MRM1 | 209 | 0.669 | 0.0861 | Yes |
| 5 | 9724 | UTP14C | 301 | 0.591 | 0.0646 | Yes |
| 6 | 84946 | LTV1 | 319 | 0.583 | 0.0959 | Yes |
| 7 | 84135 | UTP15 | 330 | 0.576 | 0.1318 | Yes |
| 8 | 10885 | WDR3 | 430 | 0.515 | 0.0988 | Yes |
| 9 | 54931 | TRMT10C | 446 | 0.507 | 0.1259 | Yes |
| 10 | 27340 | UTP20 | 455 | 0.501 | 0.1576 | Yes |
| 11 | 10969 | EBNA1BP2 | 483 | 0.485 | 0.1744 | Yes |
| 12 | 79707 | NOL9 | 560 | 0.441 | 0.1525 | Yes |
| 13 | 51388 | NIP7 | 593 | 0.428 | 0.1614 | Yes |
| 14 | 6208 | RPS14 | 604 | 0.421 | 0.1856 | Yes |
| 15 | 23404 | EXOSC2 | 609 | 0.418 | 0.2141 | Yes |
| 16 | 54512 | EXOSC4 | 623 | 0.411 | 0.2354 | Yes |
| 17 | 10607 | TBL3 | 698 | 0.380 | 0.2103 | Yes |
| 18 | 55759 | WDR12 | 731 | 0.359 | 0.2140 | Yes |
| 19 | 134430 | WDR36 | 733 | 0.359 | 0.2402 | Yes |
| 20 | 51602 | NOP58 | 750 | 0.348 | 0.2547 | Yes |
| 21 | 55178 | RNMTL1 | 771 | 0.337 | 0.2654 | Yes |
| 22 | 51118 | UTP11L | 773 | 0.336 | 0.2899 | Yes |
| 23 | 1736 | DKC1 | 791 | 0.330 | 0.3023 | Yes |
| 24 | 23246 | BOP1 | 797 | 0.325 | 0.3230 | Yes |
| 25 | 27341 | RRP7A | 801 | 0.320 | 0.3448 | Yes |
| 26 | 29960 | FTSJ2 | 805 | 0.319 | 0.3665 | Yes |
| 27 | 10528 | NOP56 | 837 | 0.285 | 0.3655 | Yes |
| 28 | 4691 | NCL | 843 | 0.270 | 0.3821 | Yes |
| 29 | 8780 | RIOK3 | 948 | -0.367 | 0.3343 | No |
Table: GSEA details [plain text format]

  

Fig 2: REACTOME\_RRNA\_PROCESSING      
 Blue-Pink O' Gram in the Space of the Analyzed GeneSet

  

Fig 3: REACTOME\_RRNA\_PROCESSING: Random ES distribution      
 Gene set null distribution of ES for **REACTOME\_RRNA\_PROCESSING**

  
