# Supplementary material for: Role and mechanism of NCAPD3 in promoting malignant behaviors in gastric cancer
Source: Front Pharmacol. 2024 Apr 22;15:1341039. doi: 10.3389/fphar.2024.1341039 (PMC11070777; doi:10.3389/fphar.2024.1341039)
Supplement: Supplementary file 11 [file DataSheet2.ZIP › GSEA/Canonical pathways/my_analysis.Gsea.1599462267220/REACTOME_SIGNALING_BY_NOTCH.html]

Details for gene set REACTOME\_SIGNALING\_BY\_NOTCH[GSEA]

|  || Dataset | filtered\_dataset.sample\_info.cls#WT\_versus\_NCAPD3\_MUT |
| Phenotype | sample\_info.cls#WT\_versus\_NCAPD3\_MUT |
| Upregulated in class | WT |
| GeneSet | REACTOME\_SIGNALING\_BY\_NOTCH |
| Enrichment Score (ES) | 0.29649282 |
| Normalized Enrichment Score (NES) | 1.1281629 |
| Nominal p-value | 0.31764707 |
| FDR q-value | 0.81407315 |
| FWER p-Value | 1.0 |
Table: GSEA Results Summary

  

Fig 1: Enrichment plot: REACTOME\_SIGNALING\_BY\_NOTCH      
 Profile of the Running ES Score & Positions of GeneSet Members on the Rank Ordered List

  

| SYMBOL | TITLE | RANK IN GENE LIST | RANK METRIC SCORE | RUNNING ES | CORE ENRICHMENT || 1 | 9759 | HDAC4 | 46 | 0.925 | 0.0713 | Yes |
| 2 | 5087 | PBX1 | 54 | 0.908 | 0.1686 | Yes |
| 3 | 54492 | NEURL1B | 177 | 0.697 | 0.1598 | Yes |
| 4 | 5701 | PSMC2 | 244 | 0.636 | 0.1842 | Yes |
| 5 | 55869 | HDAC8 | 352 | 0.566 | 0.1714 | Yes |
| 6 | 5718 | PSMD12 | 372 | 0.552 | 0.2200 | Yes |
| 7 | 1387 | CREBBP | 387 | 0.544 | 0.2713 | Yes |
| 8 | 8454 | CUL1 | 492 | 0.477 | 0.2505 | Yes |
| 9 | 11060 | WWP2 | 503 | 0.471 | 0.2965 | Yes |
| 10 | 4088 | SMAD3 | 930 | -0.353 | 0.0312 | No |
| 11 | 7090 | TLE3 | 984 | -0.395 | 0.0377 | No |
| 12 | 3516 | RBPJ | 1021 | -0.419 | 0.0591 | No |
| 13 | 8345 | HIST1H2BH | 1207 | -0.585 | -0.0075 | No |
| 14 | 1956 | EGFR | 1266 | -0.657 | 0.0250 | No |
| 15 | 861 | RUNX1 | 1293 | -0.687 | 0.0838 | No |
Table: GSEA details [plain text format]

  

Fig 2: REACTOME\_SIGNALING\_BY\_NOTCH      
 Blue-Pink O' Gram in the Space of the Analyzed GeneSet

  

Fig 3: REACTOME\_SIGNALING\_BY\_NOTCH: Random ES distribution      
 Gene set null distribution of ES for **REACTOME\_SIGNALING\_BY\_NOTCH**

  
