# Supplementary material for: Role and mechanism of NCAPD3 in promoting malignant behaviors in gastric cancer
Source: Front Pharmacol. 2024 Apr 22;15:1341039. doi: 10.3389/fphar.2024.1341039 (PMC11070777; doi:10.3389/fphar.2024.1341039)
Supplement: Supplementary file 11 [file DataSheet2.ZIP › GSEA/Canonical pathways/my_analysis.Gsea.1599462267220/REACTOME_SIGNALING_BY_NUCLEAR_RECEPTORS.html]

Details for gene set REACTOME\_SIGNALING\_BY\_NUCLEAR\_RECEPTORS[GSEA]

|  || Dataset | filtered\_dataset.sample\_info.cls#WT\_versus\_NCAPD3\_MUT |
| Phenotype | sample\_info.cls#WT\_versus\_NCAPD3\_MUT |
| Upregulated in class | NCAPD3\_MUT |
| GeneSet | REACTOME\_SIGNALING\_BY\_NUCLEAR\_RECEPTORS |
| Enrichment Score (ES) | -0.38766026 |
| Normalized Enrichment Score (NES) | -1.6332577 |
| Nominal p-value | 0.0302267 |
| FDR q-value | 0.11586055 |
| FWER p-Value | 0.699 |
Table: GSEA Results Summary

  

Fig 1: Enrichment plot: REACTOME\_SIGNALING\_BY\_NUCLEAR\_RECEPTORS      
 Profile of the Running ES Score & Positions of GeneSet Members on the Rank Ordered List

  

| SYMBOL | TITLE | RANK IN GENE LIST | RANK METRIC SCORE | RUNNING ES | CORE ENRICHMENT || 1 | 1387 | CREBBP | 387 | 0.544 | -0.2199 | No |
| 2 | 801 | CALM1 | 424 | 0.518 | -0.1907 | No |
| 3 | 6256 | RXRA | 439 | 0.509 | -0.1466 | No |
| 4 | 5295 | PIK3R1 | 467 | 0.493 | -0.1136 | No |
| 5 | 10274 | STAG1 | 633 | 0.406 | -0.1889 | No |
| 6 | 57665 | RDH14 | 819 | 0.304 | -0.2893 | No |
| 7 | 10123 | ARL4C | 957 | -0.377 | -0.3476 | Yes |
| 8 | 221656 | KDM1B | 968 | -0.386 | -0.3138 | Yes |
| 9 | 5163 | PDK1 | 982 | -0.394 | -0.2812 | Yes |
| 10 | 7090 | TLE3 | 984 | -0.395 | -0.2399 | Yes |
| 11 | 857 | CAV1 | 1098 | -0.475 | -0.2705 | Yes |
| 12 | 10681 | GNB5 | 1105 | -0.481 | -0.2236 | Yes |
| 13 | 8345 | HIST1H2BH | 1207 | -0.585 | -0.2339 | Yes |
| 14 | 1956 | EGFR | 1266 | -0.657 | -0.2056 | Yes |
| 15 | 861 | RUNX1 | 1293 | -0.687 | -0.1513 | Yes |
| 16 | 7031 | TFF1 | 1309 | -0.713 | -0.0862 | Yes |
| 17 | 195814 | SDR16C5 | 1314 | -0.728 | -0.0116 | Yes |
| 18 | 1839 | HBEGF | 1328 | -0.751 | 0.0589 | Yes |
Table: GSEA details [plain text format]

  

Fig 2: REACTOME\_SIGNALING\_BY\_NUCLEAR\_RECEPTORS      
 Blue-Pink O' Gram in the Space of the Analyzed GeneSet

  

Fig 3: REACTOME\_SIGNALING\_BY\_NUCLEAR\_RECEPTORS: Random ES distribution      
 Gene set null distribution of ES for **REACTOME\_SIGNALING\_BY\_NUCLEAR\_RECEPTORS**

  
