# Supplementary material for: Role and mechanism of NCAPD3 in promoting malignant behaviors in gastric cancer
Source: Front Pharmacol. 2024 Apr 22;15:1341039. doi: 10.3389/fphar.2024.1341039 (PMC11070777; doi:10.3389/fphar.2024.1341039)
Supplement: Supplementary file 11 [file DataSheet2.ZIP › GSEA/Canonical pathways/my_analysis.Gsea.1599462267220/REACTOME_SIGNALING_BY_RECEPTOR_TYROSINE_KINASES.html]

Details for gene set REACTOME\_SIGNALING\_BY\_RECEPTOR\_TYROSINE\_KINASES[GSEA]

|  || Dataset | filtered\_dataset.sample\_info.cls#WT\_versus\_NCAPD3\_MUT |
| Phenotype | sample\_info.cls#WT\_versus\_NCAPD3\_MUT |
| Upregulated in class | NCAPD3\_MUT |
| GeneSet | REACTOME\_SIGNALING\_BY\_RECEPTOR\_TYROSINE\_KINASES |
| Enrichment Score (ES) | -0.37898067 |
| Normalized Enrichment Score (NES) | -2.342423 |
| Nominal p-value | 0.0 |
| FDR q-value | 0.0043315766 |
| FWER p-Value | 0.009 |
Table: GSEA Results Summary

  

Fig 1: Enrichment plot: REACTOME\_SIGNALING\_BY\_RECEPTOR\_TYROSINE\_KINASES      
 Profile of the Running ES Score & Positions of GeneSet Members on the Rank Ordered List

  

| SYMBOL | TITLE | RANK IN GENE LIST | RANK METRIC SCORE | RUNNING ES | CORE ENRICHMENT || 1 | 117145 | THEM4 | 65 | 0.860 | -0.0161 | No |
| 2 | 3667 | IRS1 | 83 | 0.821 | 0.0016 | No |
| 3 | 79109 | MAPKAP1 | 130 | 0.770 | -0.0038 | No |
| 4 | 528 | ATP6V1C1 | 254 | 0.625 | -0.0710 | No |
| 5 | 2241 | FER | 304 | 0.588 | -0.0854 | No |
| 6 | 10818 | FRS2 | 363 | 0.558 | -0.1074 | No |
| 7 | 801 | CALM1 | 424 | 0.518 | -0.1323 | No |
| 8 | 5295 | PIK3R1 | 467 | 0.493 | -0.1450 | No |
| 9 | 1793 | DOCK1 | 489 | 0.480 | -0.1428 | No |
| 10 | 3479 | IGF1 | 511 | 0.469 | -0.1410 | No |
| 11 | 84951 | TNS4 | 554 | 0.445 | -0.1554 | No |
| 12 | 5567 | PRKACB | 595 | 0.426 | -0.1691 | No |
| 13 | 1108 | CHD4 | 627 | 0.408 | -0.1768 | No |
| 14 | 6197 | RPS6KA3 | 643 | 0.403 | -0.1730 | No |
| 15 | 23767 | FLRT3 | 781 | 0.333 | -0.2612 | No |
| 16 | 4670 | HNRNPM | 790 | 0.330 | -0.2549 | No |
| 17 | 5898 | RALA | 813 | 0.309 | -0.2597 | No |
| 18 | 3673 | ITGA2 | 858 | -0.264 | -0.2823 | No |
| 19 | 4209 | MEF2D | 910 | -0.337 | -0.3073 | No |
| 20 | 3913 | LAMB2 | 919 | -0.348 | -0.3004 | No |
| 21 | 3914 | LAMB3 | 1015 | -0.414 | -0.3548 | No |
| 22 | 10019 | SH2B3 | 1044 | -0.440 | -0.3592 | No |
| 23 | 6016 | RIT1 | 1072 | -0.458 | -0.3622 | Yes |
| 24 | 3918 | LAMC2 | 1088 | -0.466 | -0.3560 | Yes |
| 25 | 857 | CAV1 | 1098 | -0.475 | -0.3452 | Yes |
| 26 | 7074 | TIAM1 | 1143 | -0.508 | -0.3588 | Yes |
| 27 | 1282 | COL4A1 | 1165 | -0.534 | -0.3546 | Yes |
| 28 | 2152 | F3 | 1188 | -0.559 | -0.3501 | Yes |
| 29 | 5154 | PDGFA | 1191 | -0.566 | -0.3308 | Yes |
| 30 | 64759 | TNS3 | 1197 | -0.572 | -0.3135 | Yes |
| 31 | 5795 | PTPRJ | 1210 | -0.586 | -0.3008 | Yes |
| 32 | 8061 | FOSL1 | 1222 | -0.597 | -0.2869 | Yes |
| 33 | 10221 | TRIB1 | 1229 | -0.604 | -0.2691 | Yes |
| 34 | 26999 | CYFIP2 | 1234 | -0.611 | -0.2496 | Yes |
| 35 | 7057 | THBS1 | 1242 | -0.620 | -0.2320 | Yes |
| 36 | 2887 | GRB10 | 1259 | -0.646 | -0.2200 | Yes |
| 37 | 1956 | EGFR | 1266 | -0.657 | -0.2002 | Yes |
| 38 | 8651 | SOCS1 | 1272 | -0.661 | -0.1796 | Yes |
| 39 | 57761 | TRIB3 | 1292 | -0.685 | -0.1684 | Yes |
| 40 | 5046 | PCSK6 | 1297 | -0.699 | -0.1456 | Yes |
| 41 | 3084 | NRG1 | 1320 | -0.739 | -0.1346 | Yes |
| 42 | 1839 | HBEGF | 1328 | -0.751 | -0.1122 | Yes |
| 43 | 7422 | VEGFA | 1346 | -0.788 | -0.0957 | Yes |
| 44 | 4233 | MET | 1358 | -0.821 | -0.0736 | Yes |
| 45 | 4734 | NEDD4 | 1369 | -0.856 | -0.0495 | Yes |
| 46 | 5156 | PDGFRA | 1383 | -0.932 | -0.0248 | Yes |
| 47 | 2591 | GALNT3 | 1403 | -1.195 | 0.0051 | Yes |
Table: GSEA details [plain text format]

  

Fig 2: REACTOME\_SIGNALING\_BY\_RECEPTOR\_TYROSINE\_KINASES      
 Blue-Pink O' Gram in the Space of the Analyzed GeneSet

  

Fig 3: REACTOME\_SIGNALING\_BY\_RECEPTOR\_TYROSINE\_KINASES: Random ES distribution      
 Gene set null distribution of ES for **REACTOME\_SIGNALING\_BY\_RECEPTOR\_TYROSINE\_KINASES**

  
