# Supplementary material for: Role and mechanism of NCAPD3 in promoting malignant behaviors in gastric cancer
Source: Front Pharmacol. 2024 Apr 22;15:1341039. doi: 10.3389/fphar.2024.1341039 (PMC11070777; doi:10.3389/fphar.2024.1341039)
Supplement: Supplementary file 11 [file DataSheet2.ZIP › GSEA/Canonical pathways/my_analysis.Gsea.1599462267220/REACTOME_SIGNALING_BY_WNT.html]

Details for gene set REACTOME\_SIGNALING\_BY\_WNT[GSEA]

|  || Dataset | filtered\_dataset.sample\_info.cls#WT\_versus\_NCAPD3\_MUT |
| Phenotype | sample\_info.cls#WT\_versus\_NCAPD3\_MUT |
| Upregulated in class | WT |
| GeneSet | REACTOME\_SIGNALING\_BY\_WNT |
| Enrichment Score (ES) | 0.36942413 |
| Normalized Enrichment Score (NES) | 1.476214 |
| Nominal p-value | 0.07387388 |
| FDR q-value | 0.44060424 |
| FWER p-Value | 0.982 |
Table: GSEA Results Summary

  

Fig 1: Enrichment plot: REACTOME\_SIGNALING\_BY\_WNT      
 Profile of the Running ES Score & Positions of GeneSet Members on the Rank Ordered List

  

| SYMBOL | TITLE | RANK IN GENE LIST | RANK METRIC SCORE | RUNNING ES | CORE ENRICHMENT || 1 | 54894 | RNF43 | 29 | 1.000 | 0.0668 | Yes |
| 2 | 84133 | ZNRF3 | 30 | 0.996 | 0.1541 | Yes |
| 3 | 7976 | FZD3 | 88 | 0.815 | 0.1846 | Yes |
| 4 | 6925 | TCF4 | 132 | 0.768 | 0.2210 | Yes |
| 5 | 8549 | LGR5 | 148 | 0.739 | 0.2750 | Yes |
| 6 | 5701 | PSMC2 | 244 | 0.636 | 0.2625 | Yes |
| 7 | 324 | APC | 281 | 0.607 | 0.2899 | Yes |
| 8 | 5718 | PSMD12 | 372 | 0.552 | 0.2736 | Yes |
| 9 | 1387 | CREBBP | 387 | 0.544 | 0.3112 | Yes |
| 10 | 801 | CALM1 | 424 | 0.518 | 0.3307 | Yes |
| 11 | 8607 | RUVBL1 | 451 | 0.506 | 0.3564 | Yes |
| 12 | 8454 | CUL1 | 492 | 0.477 | 0.3694 | Yes |
| 13 | 2932 | GSK3B | 714 | 0.367 | 0.2429 | No |
| 14 | 7090 | TLE3 | 984 | -0.395 | 0.0844 | No |
| 15 | 857 | CAV1 | 1098 | -0.475 | 0.0449 | No |
| 16 | 10681 | GNB5 | 1105 | -0.481 | 0.0828 | No |
| 17 | 8345 | HIST1H2BH | 1207 | -0.585 | 0.0615 | No |
| 18 | 4919 | ROR1 | 1385 | -0.954 | 0.0179 | No |
Table: GSEA details [plain text format]

  

Fig 2: REACTOME\_SIGNALING\_BY\_WNT      
 Blue-Pink O' Gram in the Space of the Analyzed GeneSet

  

Fig 3: REACTOME\_SIGNALING\_BY\_WNT: Random ES distribution      
 Gene set null distribution of ES for **REACTOME\_SIGNALING\_BY\_WNT**

  
