# Supplementary figures and images for: Role and mechanism of NCAPD3 in promoting malignant behaviors in gastric cancer (part 2 of 2)
Source: Front Pharmacol. 2024 Apr 22;15:1341039. doi: 10.3389/fphar.2024.1341039 (PMC11070777; doi:10.3389/fphar.2024.1341039)

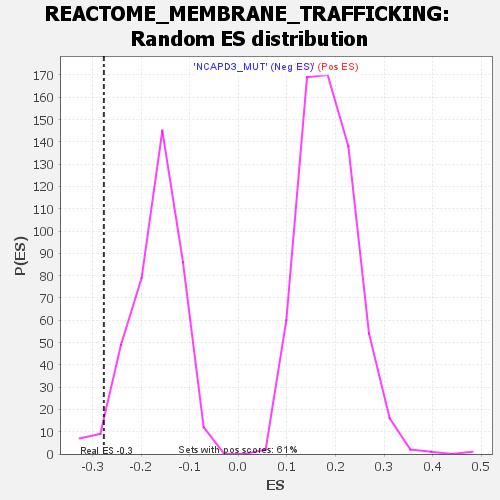

Supplement: Supplementary file 11 [file DataSheet2.ZIP › GSEA/Canonical pathways/my_analysis.Gsea.1599462267220/gset_rnd_es_dist_815.png]

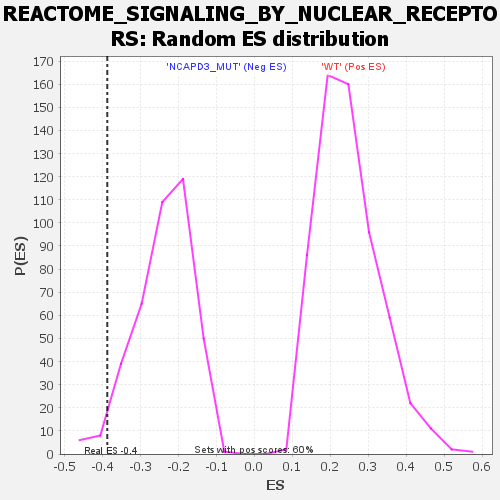

Supplement: Supplementary file 11 [file DataSheet2.ZIP › GSEA/Canonical pathways/my_analysis.Gsea.1599462267220/gset_rnd_es_dist_818.png]

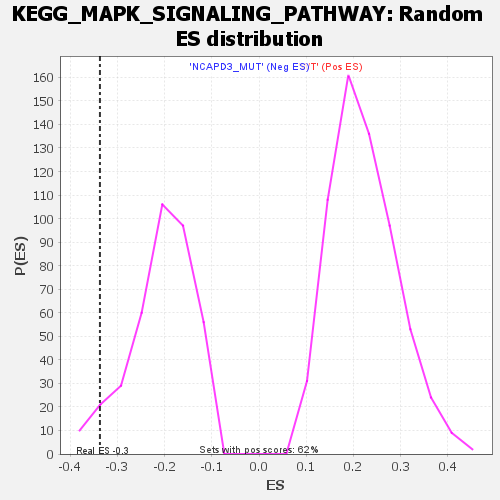

Supplement: Supplementary file 11 [file DataSheet2.ZIP › GSEA/Canonical pathways/my_analysis.Gsea.1599462267220/gset_rnd_es_dist_821.png]

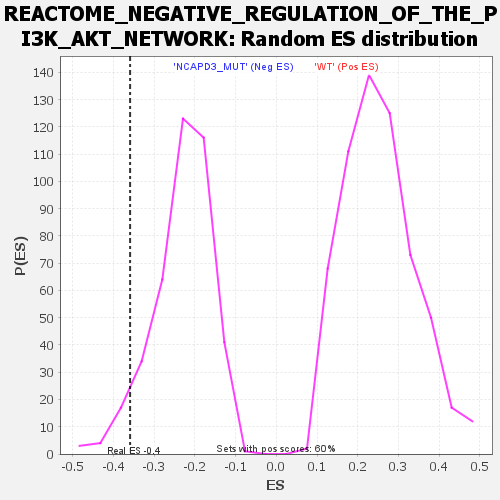

Supplement: Supplementary file 11 [file DataSheet2.ZIP › GSEA/Canonical pathways/my_analysis.Gsea.1599462267220/gset_rnd_es_dist_824.png]

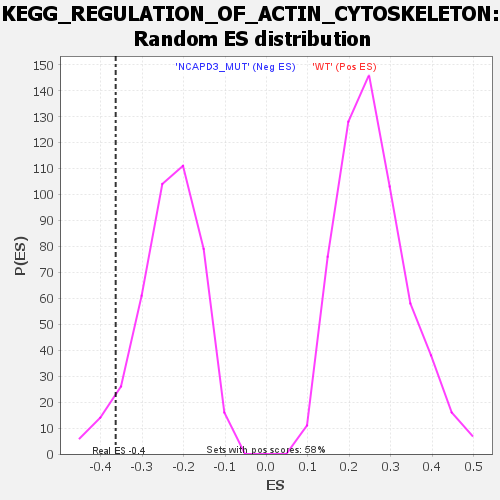

Supplement: Supplementary file 11 [file DataSheet2.ZIP › GSEA/Canonical pathways/my_analysis.Gsea.1599462267220/gset_rnd_es_dist_827.png]

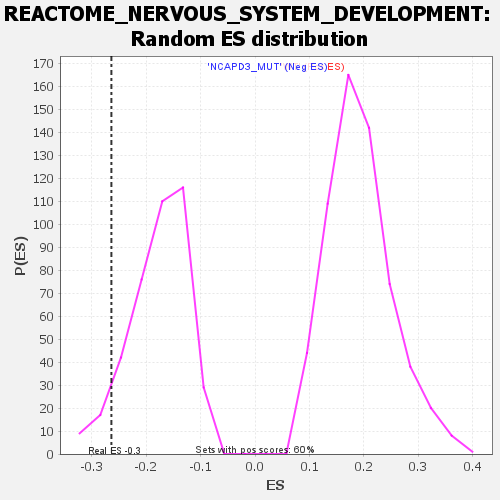

Supplement: Supplementary file 11 [file DataSheet2.ZIP › GSEA/Canonical pathways/my_analysis.Gsea.1599462267220/gset_rnd_es_dist_830.png]

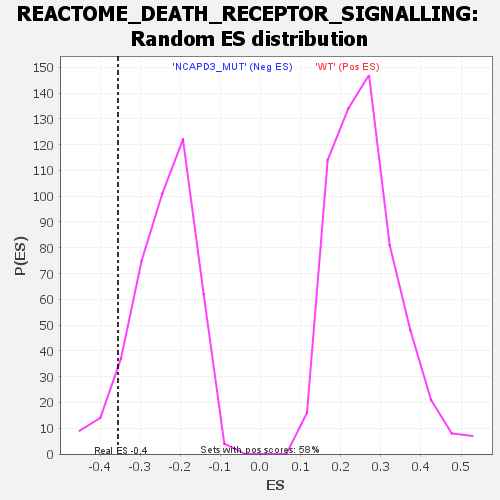

Supplement: Supplementary file 11 [file DataSheet2.ZIP › GSEA/Canonical pathways/my_analysis.Gsea.1599462267220/gset_rnd_es_dist_833.png]

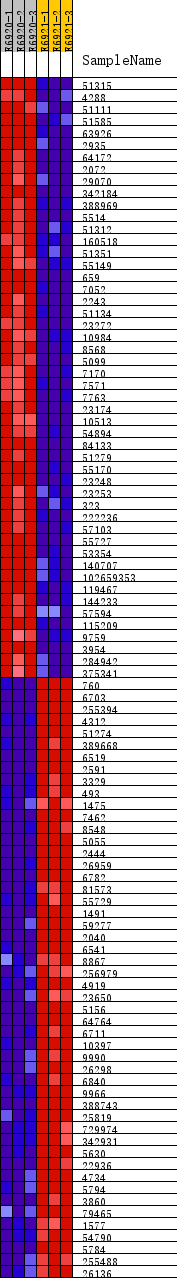

Supplement: Supplementary file 11 [file DataSheet2.ZIP › GSEA/Canonical pathways/my_analysis.Gsea.1599462267220/heat_map_712.png]

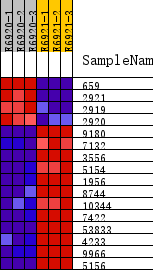

Supplement: Supplementary file 11 [file DataSheet2.ZIP › GSEA/Canonical pathways/my_analysis.Gsea.1599462267220/KEGG_CYTOKINE_CYTOKINE_RECEPTOR_INTERACTION_796.png]

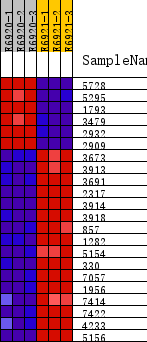

Supplement: Supplementary file 11 [file DataSheet2.ZIP › GSEA/Canonical pathways/my_analysis.Gsea.1599462267220/KEGG_FOCAL_ADHESION_802.png]

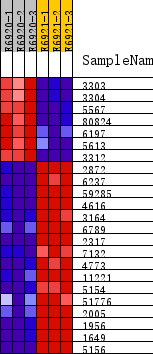

Supplement: Supplementary file 11 [file DataSheet2.ZIP › GSEA/Canonical pathways/my_analysis.Gsea.1599462267220/KEGG_MAPK_SIGNALING_PATHWAY_820.png]

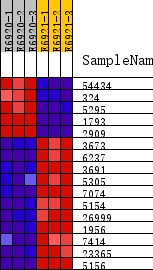

Supplement: Supplementary file 11 [file DataSheet2.ZIP › GSEA/Canonical pathways/my_analysis.Gsea.1599462267220/KEGG_REGULATION_OF_ACTIN_CYTOSKELETON_826.png]

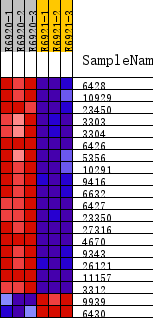

Supplement: Supplementary file 11 [file DataSheet2.ZIP › GSEA/Canonical pathways/my_analysis.Gsea.1599462267220/KEGG_SPLICEOSOME_742.png]

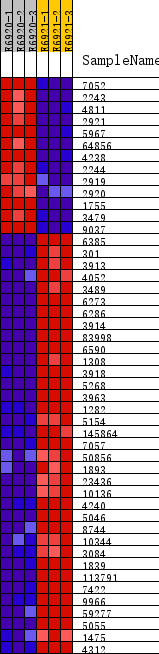

Supplement: Supplementary file 11 [file DataSheet2.ZIP › GSEA/Canonical pathways/my_analysis.Gsea.1599462267220/NABA_MATRISOME_778.png]

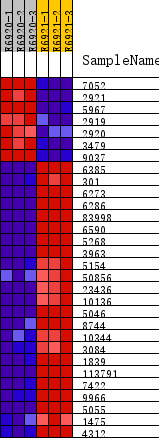

Supplement: Supplementary file 11 [file DataSheet2.ZIP › GSEA/Canonical pathways/my_analysis.Gsea.1599462267220/NABA_MATRISOME_ASSOCIATED_775.png]

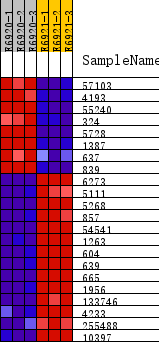

Supplement: Supplementary file 11 [file DataSheet2.ZIP › GSEA/Canonical pathways/my_analysis.Gsea.1599462267220/PID_P53_DOWNSTREAM_PATHWAY_808.png]

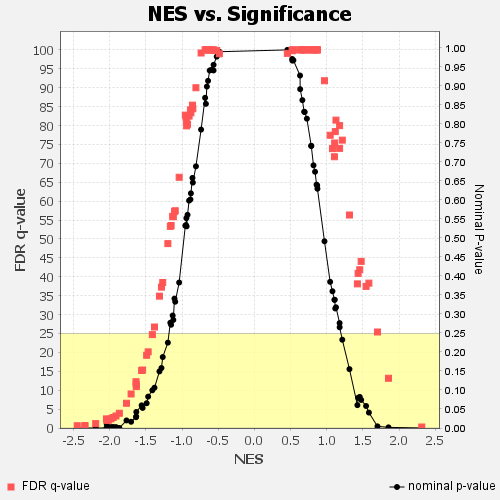

Supplement: Supplementary file 11 [file DataSheet2.ZIP › GSEA/Canonical pathways/my_analysis.Gsea.1599462267220/pvalues_vs_nes_plot.png]

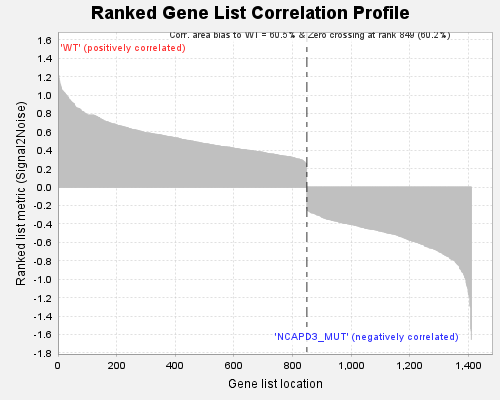

Supplement: Supplementary file 11 [file DataSheet2.ZIP › GSEA/Canonical pathways/my_analysis.Gsea.1599462267220/ranked_list_corr_713.png]

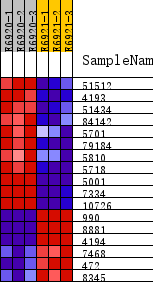

Supplement: Supplementary file 11 [file DataSheet2.ZIP › GSEA/Canonical pathways/my_analysis.Gsea.1599462267220/REACTOME_CELL_CYCLE_CHECKPOINTS_748.png]

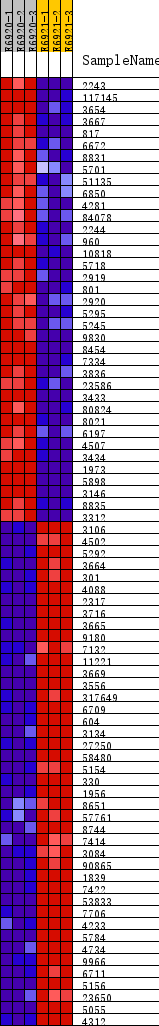

Supplement: Supplementary file 11 [file DataSheet2.ZIP › GSEA/Canonical pathways/my_analysis.Gsea.1599462267220/REACTOME_CYTOKINE_SIGNALING_IN_IMMUNE_SYSTEM_793.png]

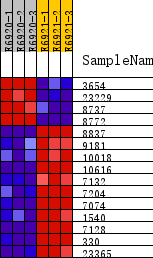

Supplement: Supplementary file 11 [file DataSheet2.ZIP › GSEA/Canonical pathways/my_analysis.Gsea.1599462267220/REACTOME_DEATH_RECEPTOR_SIGNALLING_832.png]

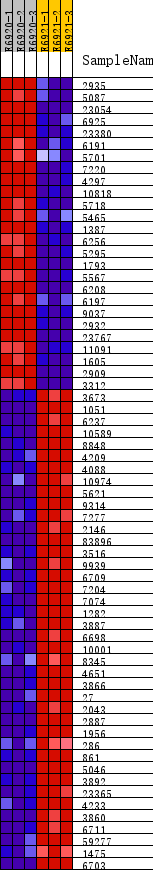

Supplement: Supplementary file 11 [file DataSheet2.ZIP › GSEA/Canonical pathways/my_analysis.Gsea.1599462267220/REACTOME_DEVELOPMENTAL_BIOLOGY_805.png]

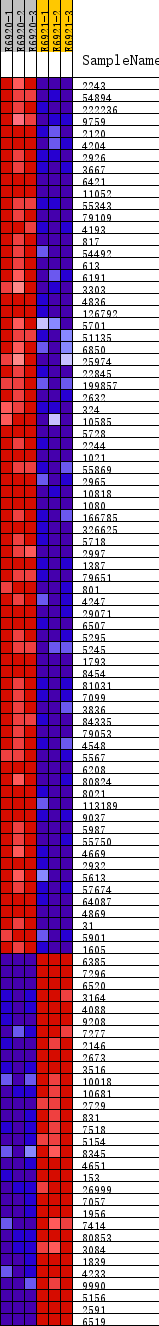

Supplement: Supplementary file 11 [file DataSheet2.ZIP › GSEA/Canonical pathways/my_analysis.Gsea.1599462267220/REACTOME_DISEASE_739.png]

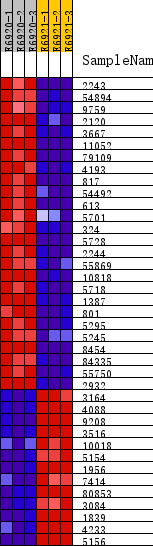

Supplement: Supplementary file 11 [file DataSheet2.ZIP › GSEA/Canonical pathways/my_analysis.Gsea.1599462267220/REACTOME_DISEASES_OF_SIGNAL_TRANSDUCTION_BY_GROWTH_FACTOR_RECEPTORS_AND_SECOND_MESSENGERS_724.png]

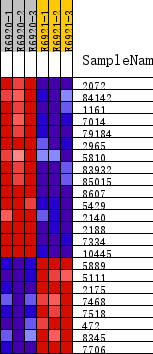

Supplement: Supplementary file 11 [file DataSheet2.ZIP › GSEA/Canonical pathways/my_analysis.Gsea.1599462267220/REACTOME_DNA_REPAIR_766.png]

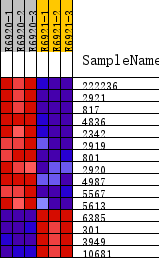

Supplement: Supplementary file 11 [file DataSheet2.ZIP › GSEA/Canonical pathways/my_analysis.Gsea.1599462267220/REACTOME_G_ALPHA_I_SIGNALLING_EVENTS_733.png]

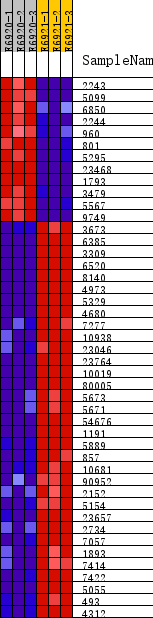

Supplement: Supplementary file 11 [file DataSheet2.ZIP › GSEA/Canonical pathways/my_analysis.Gsea.1599462267220/REACTOME_HEMOSTASIS_787.png]

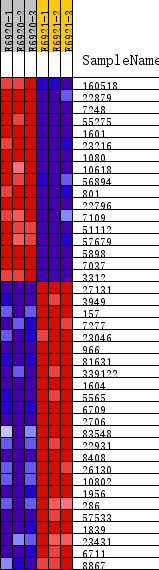

Supplement: Supplementary file 11 [file DataSheet2.ZIP › GSEA/Canonical pathways/my_analysis.Gsea.1599462267220/REACTOME_MEMBRANE_TRAFFICKING_814.png]

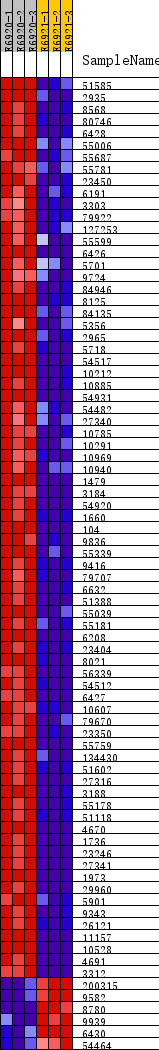

Supplement: Supplementary file 11 [file DataSheet2.ZIP › GSEA/Canonical pathways/my_analysis.Gsea.1599462267220/REACTOME_METABOLISM_OF_RNA_715.png]

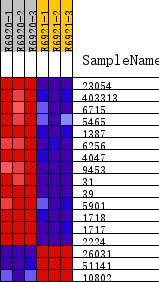

Supplement: Supplementary file 11 [file DataSheet2.ZIP › GSEA/Canonical pathways/my_analysis.Gsea.1599462267220/REACTOME_METABOLISM_OF_STEROIDS_760.png]

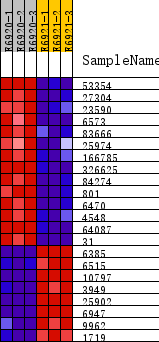

Supplement: Supplementary file 11 [file DataSheet2.ZIP › GSEA/Canonical pathways/my_analysis.Gsea.1599462267220/REACTOME_METABOLISM_OF_VITAMINS_AND_COFACTORS_751.png]

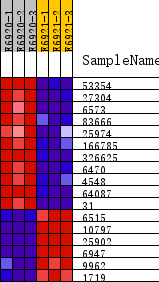

Supplement: Supplementary file 11 [file DataSheet2.ZIP › GSEA/Canonical pathways/my_analysis.Gsea.1599462267220/REACTOME_METABOLISM_OF_WATER_SOLUBLE_VITAMINS_AND_COFACTORS_769.png]

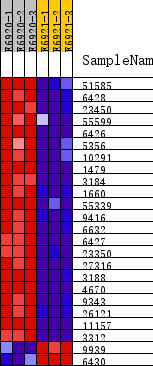

Supplement: Supplementary file 11 [file DataSheet2.ZIP › GSEA/Canonical pathways/my_analysis.Gsea.1599462267220/REACTOME_MRNA_SPLICING_736.png]

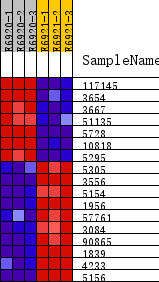

Supplement: Supplementary file 11 [file DataSheet2.ZIP › GSEA/Canonical pathways/my_analysis.Gsea.1599462267220/REACTOME_NEGATIVE_REGULATION_OF_THE_PI3K_AKT_NETWORK_823.png]

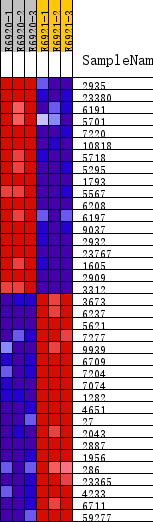

Supplement: Supplementary file 11 [file DataSheet2.ZIP › GSEA/Canonical pathways/my_analysis.Gsea.1599462267220/REACTOME_NERVOUS_SYSTEM_DEVELOPMENT_829.png]

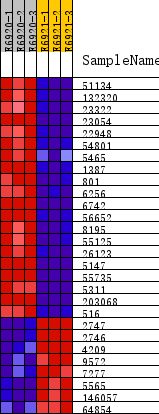

Supplement: Supplementary file 11 [file DataSheet2.ZIP › GSEA/Canonical pathways/my_analysis.Gsea.1599462267220/REACTOME_ORGANELLE_BIOGENESIS_AND_MAINTENANCE_745.png]

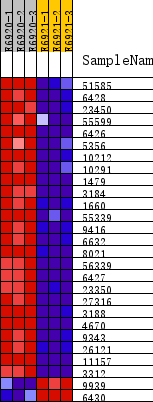

Supplement: Supplementary file 11 [file DataSheet2.ZIP › GSEA/Canonical pathways/my_analysis.Gsea.1599462267220/REACTOME_PROCESSING_OF_CAPPED_INTRON_CONTAINING_PRE_MRNA_727.png]

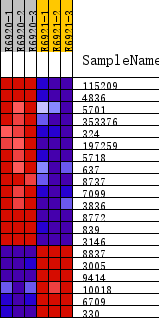

Supplement: Supplementary file 11 [file DataSheet2.ZIP › GSEA/Canonical pathways/my_analysis.Gsea.1599462267220/REACTOME_PROGRAMMED_CELL_DEATH_757.png]

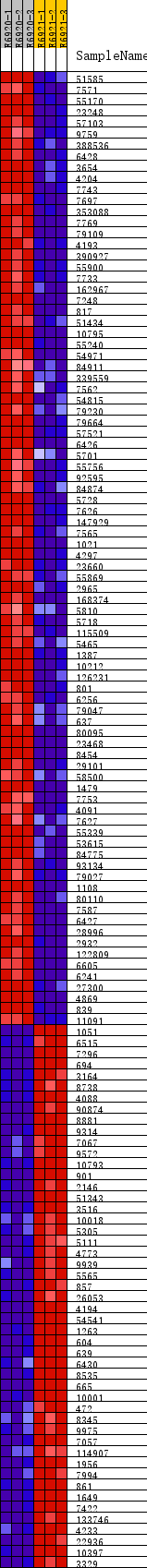

Supplement: Supplementary file 11 [file DataSheet2.ZIP › GSEA/Canonical pathways/my_analysis.Gsea.1599462267220/REACTOME_RNA_POLYMERASE_II_TRANSCRIPTION_721.png]

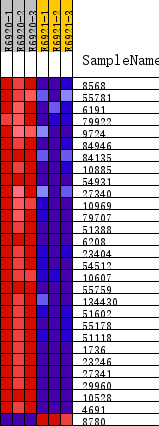

Supplement: Supplementary file 11 [file DataSheet2.ZIP › GSEA/Canonical pathways/my_analysis.Gsea.1599462267220/REACTOME_RRNA_PROCESSING_718.png]

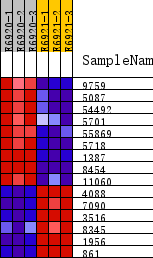

Supplement: Supplementary file 11 [file DataSheet2.ZIP › GSEA/Canonical pathways/my_analysis.Gsea.1599462267220/REACTOME_SIGNALING_BY_NOTCH_754.png]

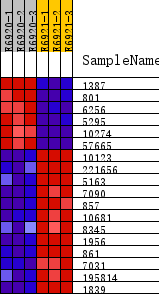

Supplement: Supplementary file 11 [file DataSheet2.ZIP › GSEA/Canonical pathways/my_analysis.Gsea.1599462267220/REACTOME_SIGNALING_BY_NUCLEAR_RECEPTORS_817.png]

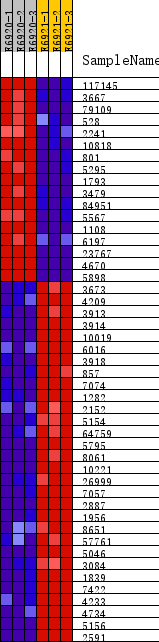

Supplement: Supplementary file 11 [file DataSheet2.ZIP › GSEA/Canonical pathways/my_analysis.Gsea.1599462267220/REACTOME_SIGNALING_BY_RECEPTOR_TYROSINE_KINASES_781.png]

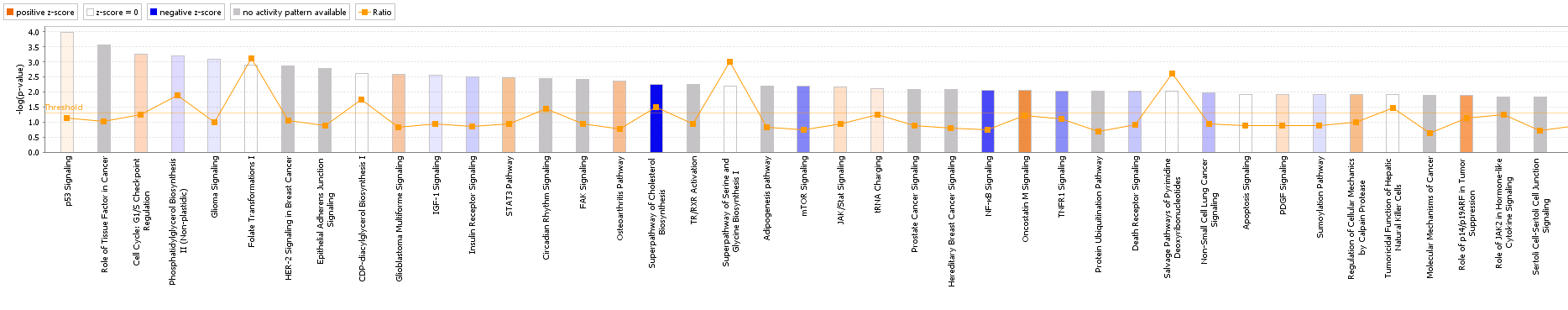

Supplement: Supplementary file 12 [file DataSheet5.ZIP › data of IPA/canonical pathway analysis/Canonical_Pathway_Histogram.png]

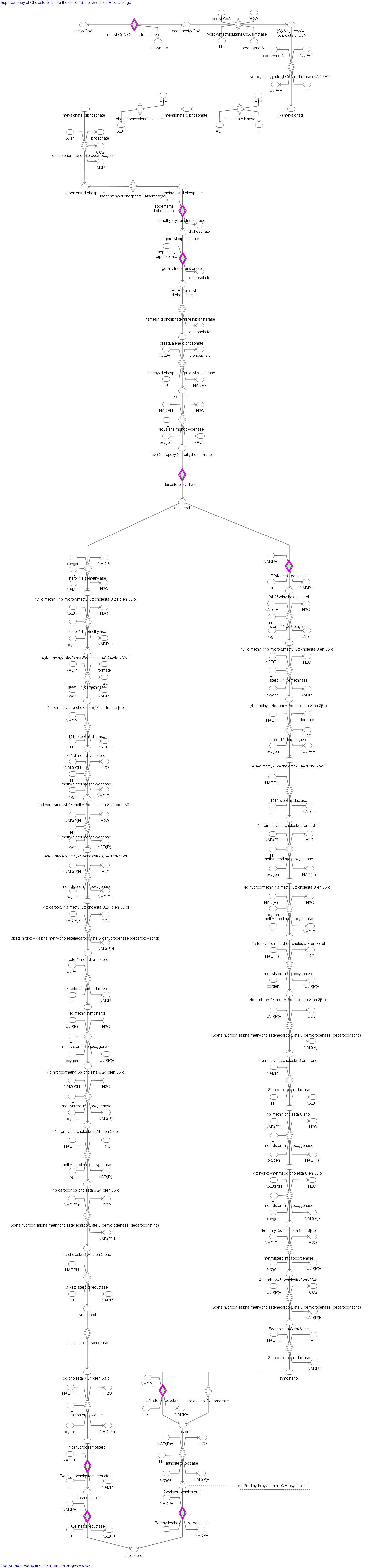

Supplement: Supplementary file 12 [file DataSheet5.ZIP › data of IPA/canonical pathway analysis/Canonical_Pathway_MAP.png]

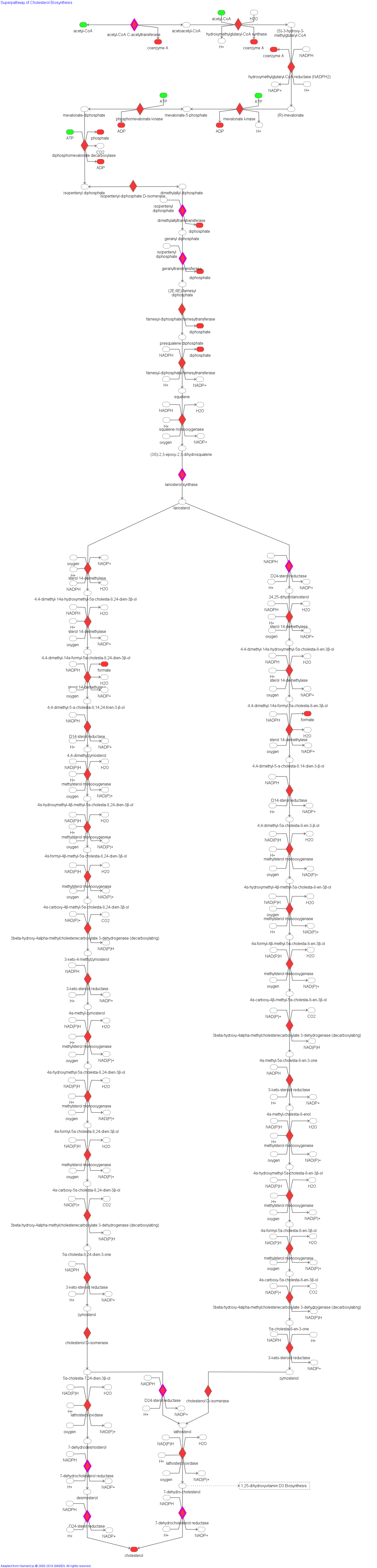

Supplement: Supplementary file 12 [file DataSheet5.ZIP › data of IPA/canonical pathway analysis/Canonical_Pathway_MAP_Supported_By_Literature.png]

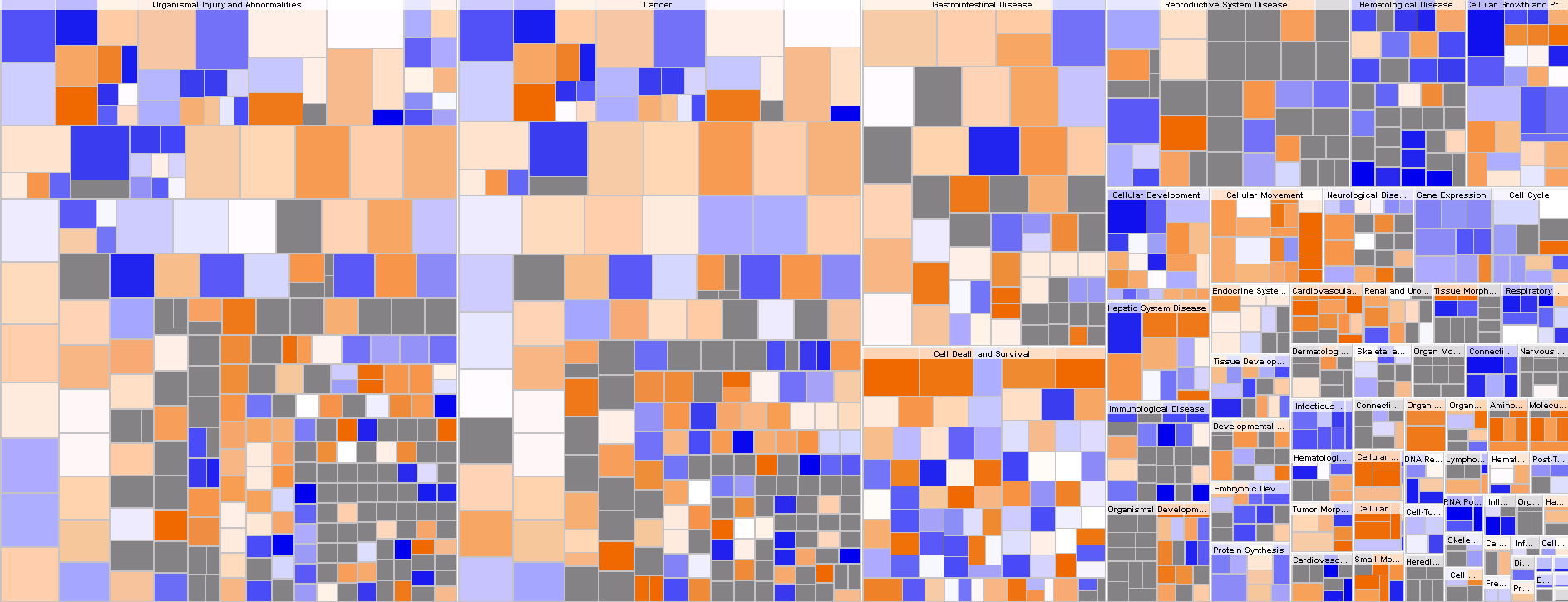

Supplement: Supplementary file 12 [file DataSheet5.ZIP › data of IPA/disease and function/Diseases_And_Functions_Heatmap.png]

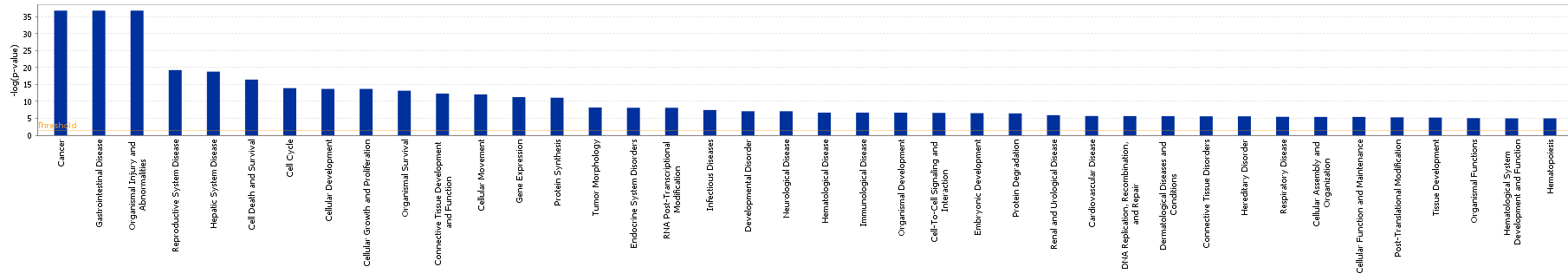

Supplement: Supplementary file 12 [file DataSheet5.ZIP › data of IPA/disease and function/Diseases_And_Functions_Histogram.png]

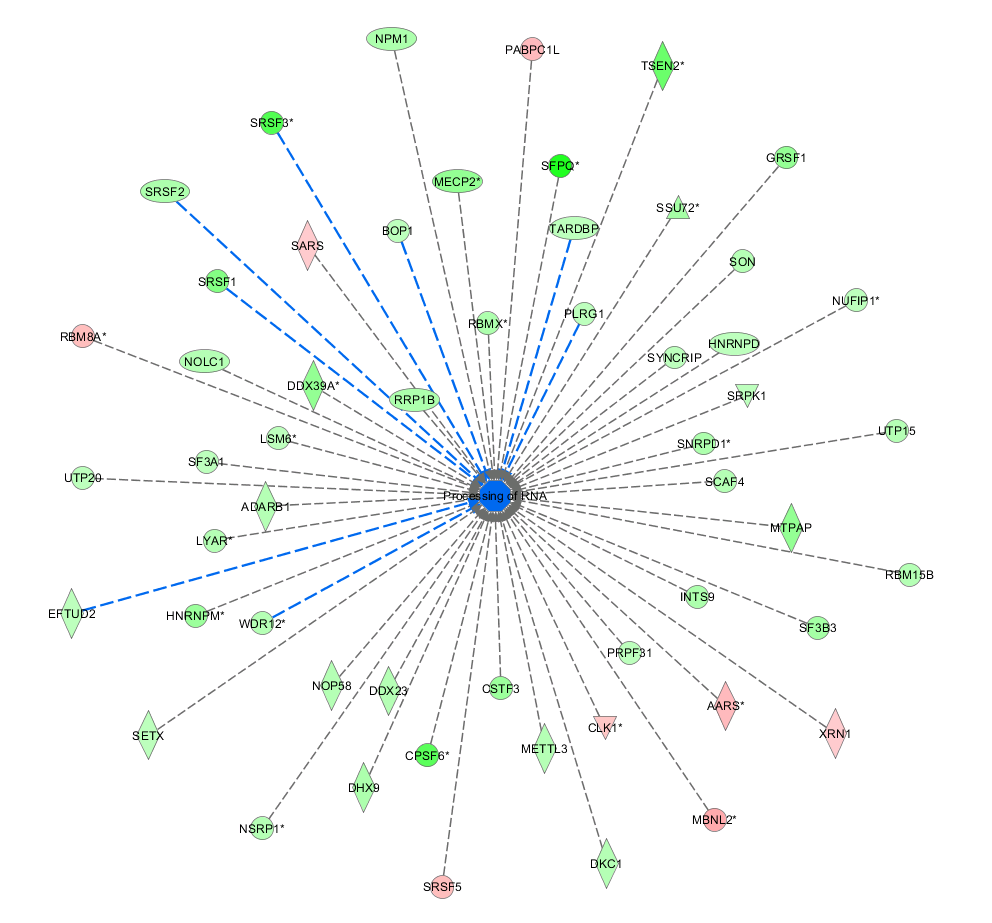

Supplement: Supplementary file 12 [file DataSheet5.ZIP › data of IPA/disease and function/Diseases_And_Functions_Network.png]

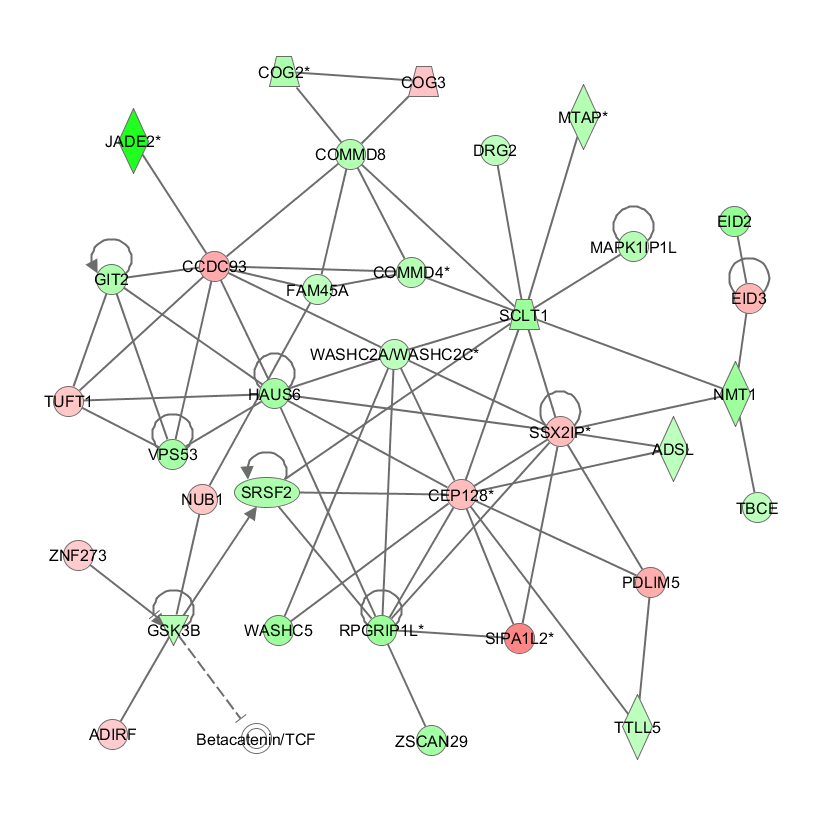

Supplement: Supplementary file 12 [file DataSheet5.ZIP › data of IPA/molecular regulatory network/Molecular_Network.png]

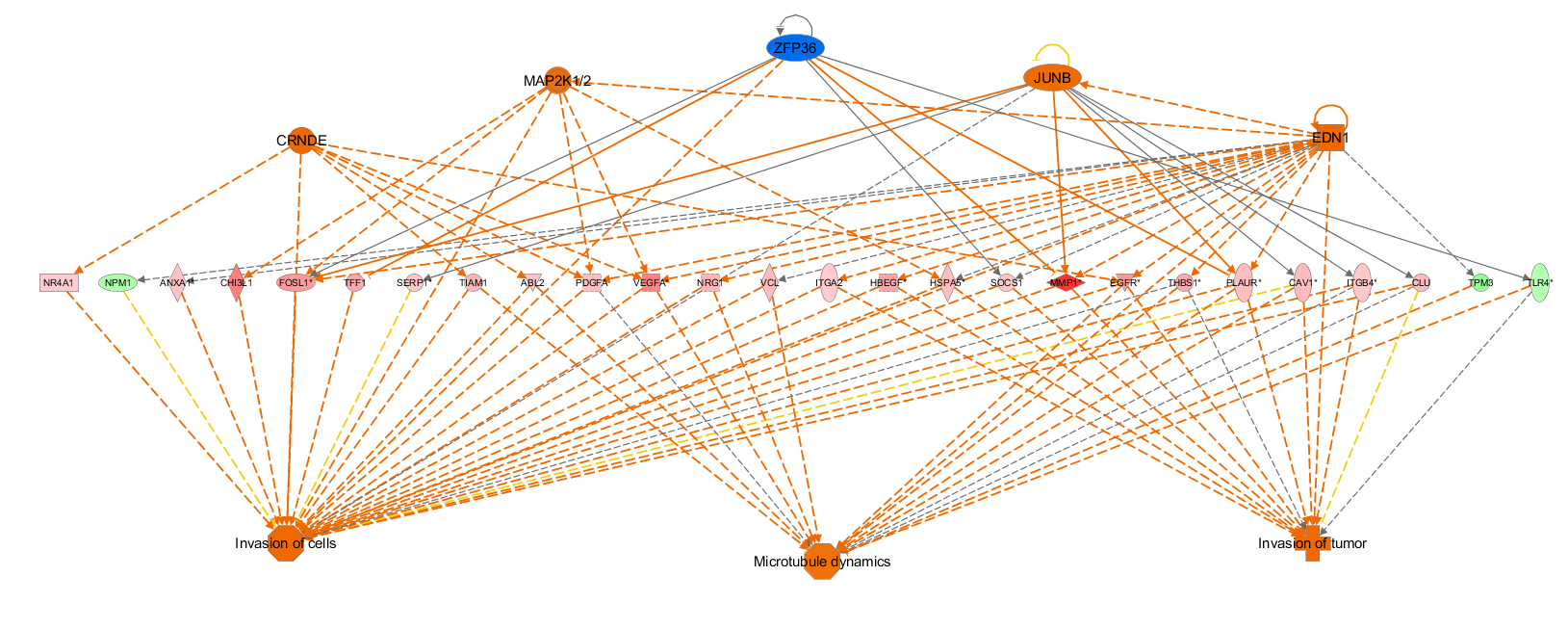

Supplement: Supplementary file 12 [file DataSheet5.ZIP › data of IPA/regulatory effect/Regulator_Effects_Network.png]

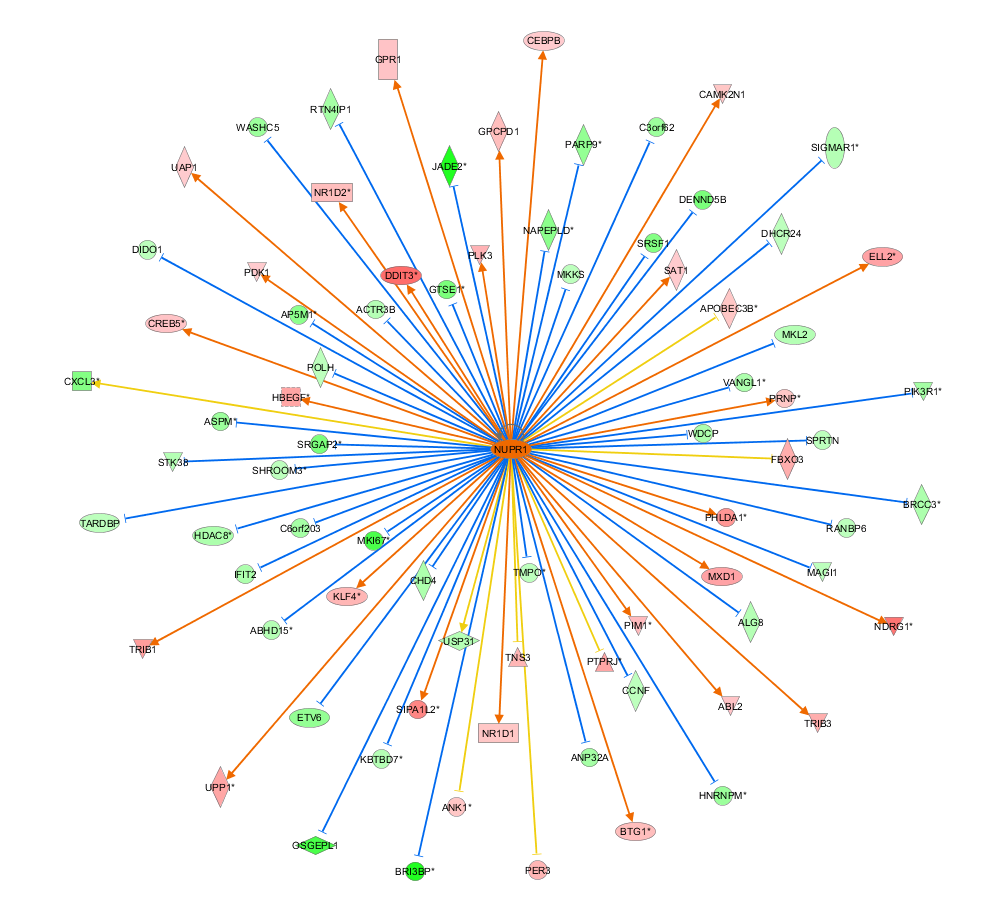

Supplement: Supplementary file 12 [file DataSheet5.ZIP › data of IPA/upstream regulatory factors/Upstream_Analysis_Network.png]

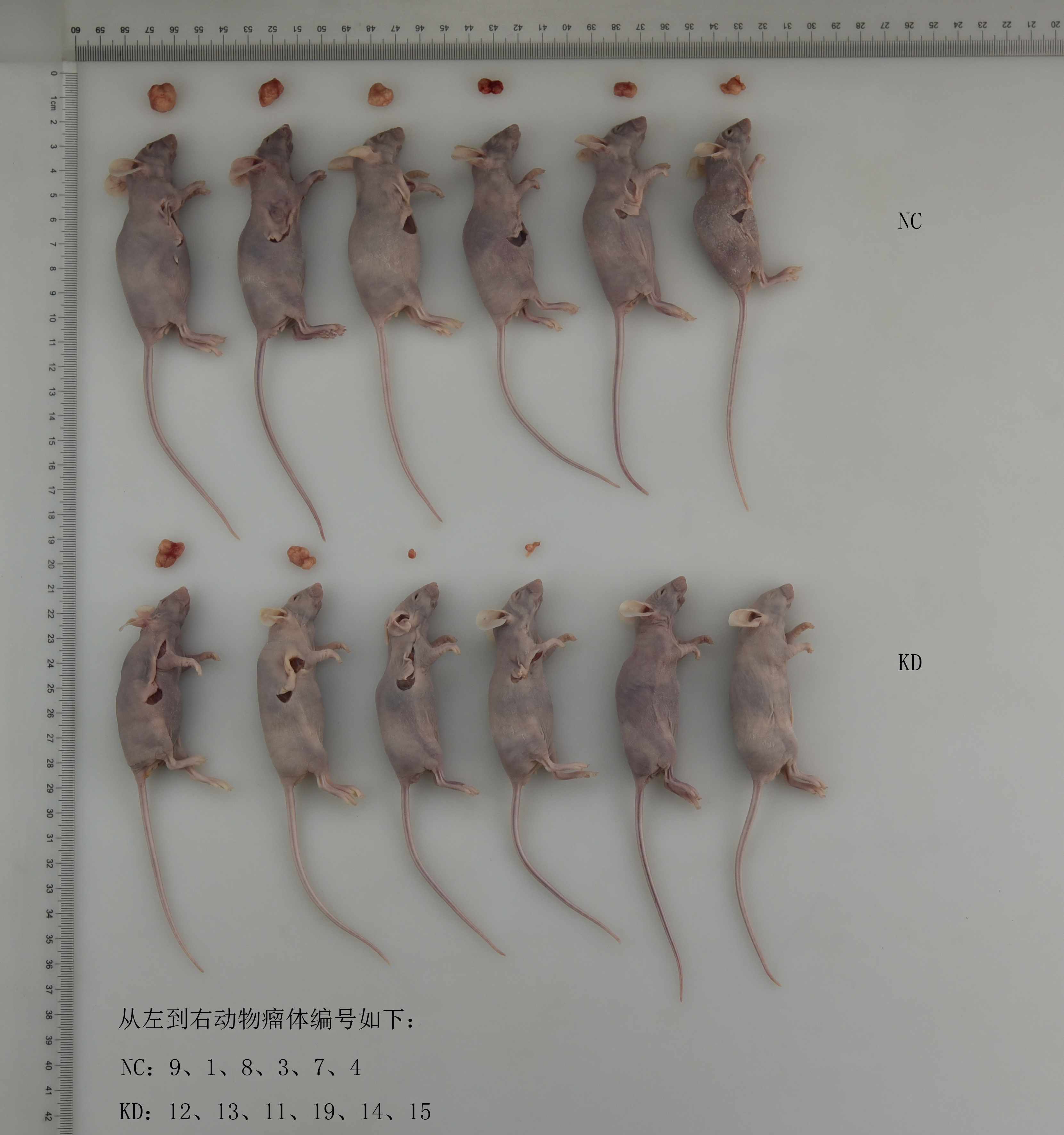

Supplement: Supplementary file 14 [file DataSheet7.ZIP › DSC_7724.JPG]
